# Corrigendum: Vaccine safety surveillance using routinely collected healthcare data—An empirical evaluation of epidemiological designs

**DOI:** 10.3389/fphar.2022.1088973

**Published:** 2022-11-24

**Authors:** Martijn J. Schuemie, Faaizah Arshad, Nicole Pratt, Fredrik Nyberg, Thamir M Alshammari, George Hripcsak, Patrick Ryan, Daniel Prieto-Alhambra, Lana Y. H. Lai, Xintong Li, Stephen Fortin, Evan Minty, Marc A. Suchard

**Affiliations:** ^1^ Observational Health Data Sciences and Informatics, New York, NY, United States; ^2^ Observational Health Data Analytics, Janssen R&D, Titusville, NJ, United States; ^3^ Department of Biostatistics, University of California, Los Angeles, Los Angeles, CA, United States; ^4^ Quality Use of Medicines and Pharmacy Research Centre, Clinical and Health Sciences, University of South Australia, Adelaide, SA, Australia; ^5^ School of Public Health and Community Medicine, Institute of Medicine, Sahlgrenska Academy, University of Gothenburg, Gothenburg, Sweden; ^6^ College of Pharmacy, Riyadh Elm University, Riyadh, Saudi Arabia; ^7^ Department of Biomedical Informatics, Columbia University, New York, NY, United States; ^8^ Centre for Statistics in Medicine, NDORMS, University of Oxford, Oxford, United Kingdom; ^9^ Department of Medical Informatics, Erasmus University Medical Center, Rotterdam, Netherlands; ^10^ O’Brien Institute for Public Health, Faculty of Medicine, University of Calgary, Calgary, AB, Canada; ^11^ Division of Medical Sciences, University of Manchester, Manchester, United Kingdom; ^12^ Department of Human Genetics, University of California, Los Angeles, Los Angeles, CA, United States

**Keywords:** vaccine safely, routinely collected data, adverse event, surveillance, methods

In the published article, there was an error in Figure 4 and Figure 5 as published. After publication, the authors found that the positive control imputation multiplication was accidentally applied twice, meaning the intended multiplication of 1.25, 2, and 4 actually were 1.25^2^ = 1.5625, 2^2^ = 4, and 4^2^ = 16, respectively. This means the type 2 error in Figure 4 and the time to 50% sensitivity in Figure 5 were underestimated. The corrected Figure 4 and Figure 5 appear below.

**FIGURE 4:**
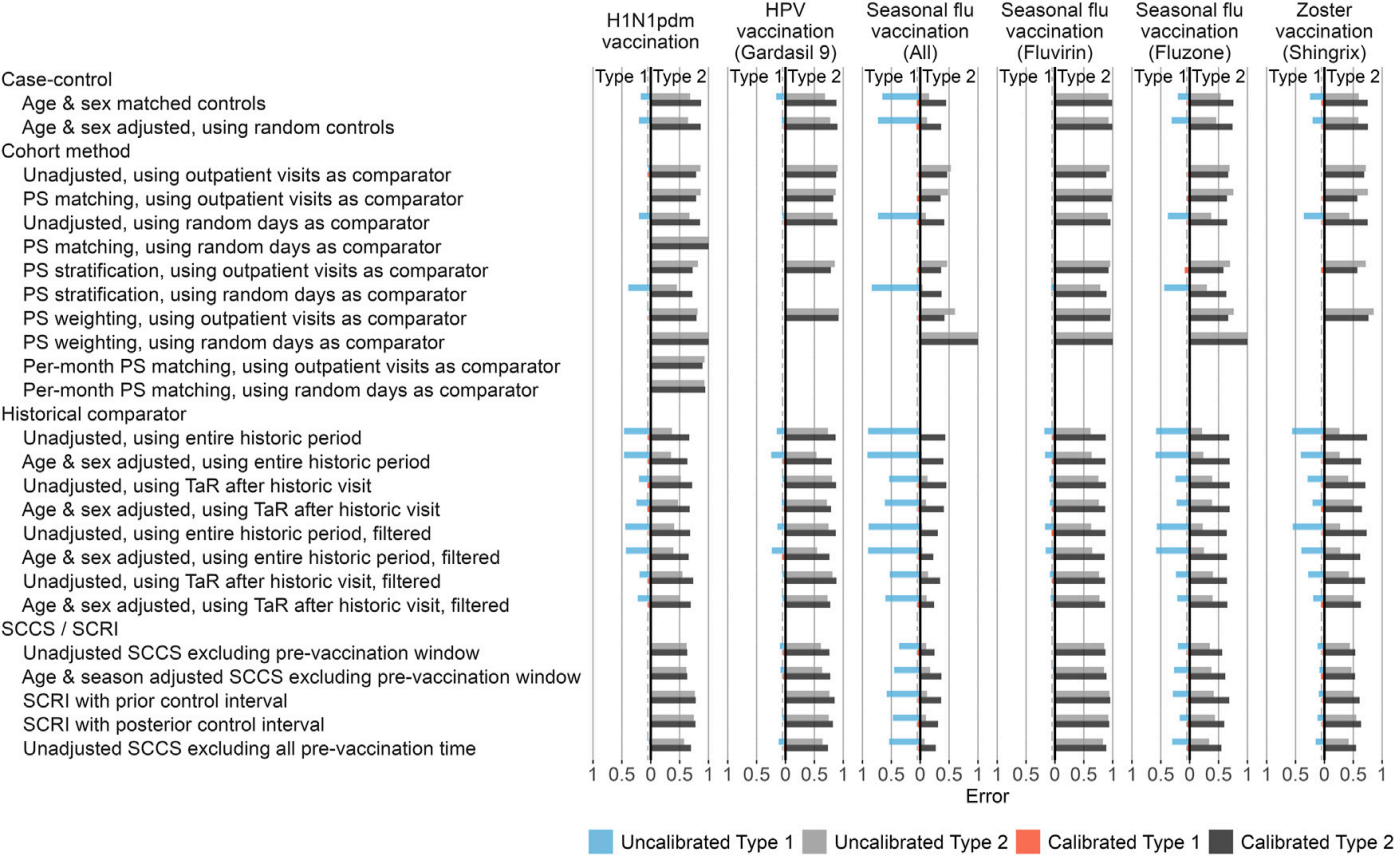
Type 1 and 2 error before and after empirical calibration. For each method variation and vaccine group, the type 1 and 2 error before and after empirical calibration in the Optum EHR database are shown. The *x*-axis indicates the type 1 error (higher values to the left) and type 2 error (higher values to the right), based on the (calibrated) one-sided *p*-value. The dashed line indicates nominal type 1 error of 5%. HPV = Human papillomavirus, PS = Propensity Score, SCCS = Self-Controlled Case Series, SCRI = Self-Controlled Risk Interval, TaR = Time-at-Risk.

**FIGURE 5:**
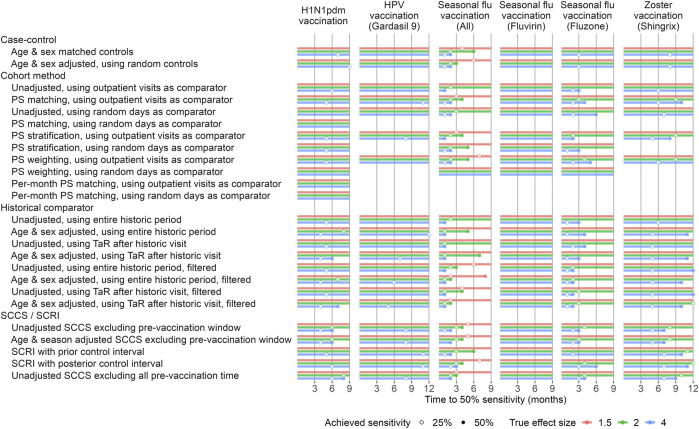
Time to 50% sensitivity after calibration. For each method variation and vaccine group, the number of months of data needed to achieve 50% sensitivity based on the calibrated MaxSPRT in the Optum EHR database are shown, stratified by true effect size of the positive controls. HPV = Human papillomavirus, PS = Propensity Score, SCCS = Self-Controlled Case Series, SCRI = Self-Controlled Risk Interval, TaR = Time-at-Risk.

In the published article the same error was present in the Supplementary Material. This means the type 2 error and the time to 50% sensitivity were underestimated. The correct Supplementary Data Sheet S1 can be found in the Supplementary Materials.

The authors apologize for these errors and state that they does not change the scientific conclusions of the article in any way. The original article has been updated.

