# Supplementary material for: Corrigendum: Vaccine safety surveillance using routinely collected healthcare data—An empirical evaluation of epidemiological designs
Source: Front Pharmacol. 2022 Nov 24;13:1088973. doi: 10.3389/fphar.2022.1088973 (PMC9731373; doi:10.3389/fphar.2022.1088973)
Supplement: Supplementary file 1 [file DataSheet1.PDF]

Supplementary Materials for

Vaccine safety surveillance using routinely collected healthcare data -Empirical evaluation of statistical methods

Contents

1 List of Abbreviations **4**

2 Exposures **5**

2.1 H1N1pdm Vaccines 5
2.2 Seasonal Flu Vaccines (Fluvirin) 6
2.3 Seasonal Flu Vaccines (Fluzone) 7
2.4 Seasonal Flu Vaccines (All) 8
2.5 HPV Vaccines 9
2.6 Zoster Vaccines 10

3 Negative control outcomes **12**

4 Data sources **14**

4.1 Database characteristics 15

5 Evaluated Methods **16**

5.1 Counterfactual construction 16
5.2 Time-at-risk 18
5.3 Statistic 18
5.4 Decision rule 19

6 Fitted systematic error distribution 20
6.1 Systematic error distributions in the Optum EHR database 20
6.2 Systematic error distributions in the MDCD database 21
6.3 Systematic error distributions in the MDCR database 22
6.4 Systematic error distributions in the CCAE database 23

7 Type 1 and 2 error before and after calibration 24
7.1 Type 1 and 2 error before and after calibration in the Optum EHR database 24
7.2 Type 1 and 2 error before and after calibration in the MDCD database . . 25
7.3 Type 1 and 2 error before and after calibration in the MDCR database . . 26
7.4 Type 1 and 2 error before and after calibration in the CCAE database . . 27

8 Time to 50% sensitivity 28
8.1 Time to 50% sensitivity in the Optum EHR database 28
8.2 Time to 50% sensitivity in the MDCD database 29
8.3 Time to 50% sensitivity in the MDCR database 30
8.4 Time to 50% sensitivity in the CCAE database 31

9 Time to 80% sensitivity 32
9.1 Time to 80% sensitivity in the Optum EHR database 32
9.2 Time to 80% sensitivity in the MDCD database 33
9.3 Time to 80% sensitivity in the MDCR database 34
9.4 Time to 80% sensitivity in the CCAE database 35

10 Type 1 and 2 error by true effect size based on p-value 36
10.1 Type 1 and 2 error for H1N1 vaccination 36
10.2 Type 1 and 2 error for Seasonal flu vaccination (Fluvirin) 40
10.3 Type 1 and 2 error for Seasonal flu vaccination (Fluzone) 44
10.4 Type 1 and 2 error for Seasonal flu vaccination (All) 48
10.5 Type 1 and 2 error for Zoster vaccination (Shingrix) 52
10.6 Type 1 and 2 error for HPV vaccination (Gardasil 9) 56

11 Type 1 and 2 error by true effect size based on MaxSPRT 60
11.1 Type 1 and 2 error for H1N1 vaccination 60
11.2 Type 1 and 2 error for Seasonal flu vaccination (Fluvirin) 64
11.3 Type 1 and 2 error for Seasonal flu vaccination (Fluzone) 68
11.4 Type 1 and 2 error for Seasonal flu vaccination (All) 72
11.5 Type 1 and 2 error for Zoster vaccination (Shingrix) 76
11.6 Type 1 and 2 error for HPV vaccination (Gardasil 9) 80

12 AUC based on effect-size estimate 84
12.1 AUC for H1N1 vaccination 84
12.2 AUC for Seasonal flu vaccination (Fluvirin) 88
12.3 AUC for Seasonal flu vaccination (Fluzone) 92
12.4 AUC for Seasonal flu vaccination (All) 96
12.5 AUC for Zoster vaccination (Shingrix) 100
12.6 AUC for HPV vaccination (Gardasil 9) 104

13 Confidence-interval-based metrics 108
13.1 Confidence-interval-based metrics for H1N1 vaccination 108
13.2 Confidence-interval-based metrics for Seasonal flu vaccination (Fluvirin) . 112
13.3 Confidence-interval-based metrics for Seasonal flu vaccination (Fluzone) . 116
13.4 Confidence-interval-based metrics for Seasonal flu vaccination (All) . . . 120
13.5 Confidence-interval-based metrics for Zoster vaccination (Shingrix) 124
13.6 Confidence-interval-based metrics for HPV vaccination (Gardasil 9) . . . 128

14 Negative controls effect size estimates 132
14.1 Negative controls effect size estimates in Optum EHR 132
14.2 Negative controls effect size estimates in MDCD 141
14.3 Negative controls effect size estimates in MDCR 150
14.4 Negative controls effect size estimates in CCAE 159

AUC	Area Under the receiver-operator Curve
CCAE	IBM MarketScan Commercial Claims and Encounters
H1N1pdm	Hemagglutinin Type 1 and Neuraminidase Type 1 (2009 pandemic influenza)
HPV	Human PapillomaVirus
LLR	Log Likelihood Ratio
MDCR	IBM MarketScan Medicare Supplemental Database
MDCD	IBM MarketScan Multi-State Medicaid Database
MSE	Mean Squared Error
OHDSI	Observational Health Data Science and Informatics
OMOP	Observational Medical Outcomes Partnership
MaxSPRT	MAXimized Sequential Probability Ratio Test
PS	Propensity score
SCCS	Self-Controlled Case Series
SCRI	Self-Controlled Risk Interval

15 Negative controls effect log likelihood ratios 168

15.1 Negative controls log likelihood ratios in Optum EHR 168

15.2 Negative controls log likelihood ratios in MDCD 181

15.3 Negative controls log likelihood ratios in MDCR 194

15.4 Negative controls log likelihood ratios in CCAE 207

References 220

1 List of Abbreviations

```
## Rows: 14 Columns: 2
## -- Column specification --------------------------------------------------------
## Delimiter: ";"
## chr (2): X1, X2
##
## i Use `spec()` to retrieve the full column specification for this data.
## i Specify the column types or set `show_col_types = FALSE` to quiet this message.
```

Table 1: Exposures of interest.

Exposure Name	Start Date	End Date	History Start Date	History End Date
H1N1pdm vaccination	01-09-2009	31-05-2010	01-09-2008	31-05-2009
Seasonal flu vaccination (Fluvirin)	01-09-2017	31-05-2018	01-09-2016	31-05-2017
Seasonal flu vaccination (Fluzone)	01-09-2017	31-05-2018	01-09-2016	31-05-2017
Seasonal flu vaccination (All)	01-09-2017	31-05-2018	01-09-2016	31-05-2017
Zoster vaccination (Shingrix)	01-01-2018	31-12-2018	01-01-2017	31-12-2017
HPV vaccination (Gardasil 9)	01-01-2018	31-12-2018	01-01-2017	31-12-2017

2 Exposures

The evaluation centers on six existing (groups of) vaccines, for specific time periods (start date to end date), as shown in Table 1.

For some methods the period between historic start and historic end date is used to estimate the historic incidence rate. The formal cohort definitions of each exposure can be found in the next sections.

2.1 H1N1pdm Vaccines

2.1.1 Cohort Entry Events

People enter the cohort when observing any of the following:

1. drug exposures of ‘H1N1 vaccine’, starting between September 1, 2009 and May 31, 2010.

Limit cohort entry events to the earliest event per person.

2.1.2 Cohort Exit

The cohort end date will be offset from index event’s start date plus 0 days.

2.1.3 Cohort Eras

Entry events will be combined into cohort eras if they are within 0 days of each other.

2.1.4 Concept set: H1N1 vaccine

Concept ID	Concept Name	Code	Vocabulary	Excluded	Descendants	Mapped
40213187	Novel influenza-H1N1-09, all formulations	128	CVX	NO	YES	NO|
40166607	influenza A-California-7-2009-(H1N1)v-like virus vaccine 0.03 MG/ML Injectable Suspension	864704	RxNorm	NO	YES	NO|
40166130	0.25 ML influenza A-California-7-2009-(H1N1)v-like virus vaccine 0.03 MG/ML Prefilled Syringe	864781	RxNorm	NO	YES	NO|
40166144	0.5 ML influenza A-California-7-2009-(H1N1)v-like virus vaccine 0.03 MG/ML Prefilled Syringe	864797	RxNorm	NO	YES	NO|
42902936	influenza A-California-7-2009-(H1N1)v-like virus vaccine 0.03 MG/ML Prefilled Syringe	1360049	RxNorm	NO	YES	NO|
40240135	influenza A-California-7-2009-(H1N1)v-like virus vaccine 0.09 MG/ML	1111367	RxNorm	NO	YES	NO|
40225009	influenza A-California-7-2009-(H1N1)v-like virus vaccine 0.12 MG/ML	1005949	RxNorm	NO	YES	NO|
40166608	influenza A-California-7-2009-(H1N1)v-like virus vaccine 158000000 UNT/ML	864812	RxNorm	NO	YES	NO|
45776785	influenza A-California-7-2009-(H1N1)v-like virus vaccine 50000000 MG/ML	1543758	RxNorm	NO	YES	NO|
40166609	influenza A-California-7-2009-(H1N1)v-like virus vaccine Injectable Suspension	864703	RxNorm	NO	YES	NO|
40166611	influenza A-California-7-2009-(H1N1)v-like virus vaccine Prefilled Syringe	864780	RxNorm	NO	YES	NO|

2.2 Seasonal Flu Vaccines (Fluvirin)

2.2.1 Cohort Entry Events

People enter the cohort when observing any of the following:

1. drug exposures of ‘Fluvirin’, starting between September 1, 2017 and May 31, 2018.

Limit cohort entry events to the earliest event per person.

2.2.2 Cohort Exit

The cohort end date will be offset from index event’s start date plus 0 days.

2.2.3 Cohort Eras

Entry events will be combined into cohort eras if they are within 0 days of each other.

2.2.4 Concept set: Fluvirin

Concept ID	Concept Name	Code	Vocabulary	Excluded	Descendants	Mapped
1593906	influenza A virus A/Hong Kong/4801/2014 (H3N2) antigen 0.03 MG/ML / influenza A virus A/Singapore/GP1908/2015 (H1N1) antigen 0.03 MG/ML / influenza B virus B/Brisbane/60/2008 antigen 0.03 MG/ML [Fluvirin 2017-2018]	1928971	RxNorm	NO	YES	NO|

2.3 Seasonal Flu Vaccines (Fluzone)

2.3.1 Cohort Entry Events

People enter the cohort when observing any of the following:

1. drug exposures of 'Fluzone', starting between September 1, 2017 and May 31, 2018.

Limit cohort entry events to the earliest event per person.

2.3.2 Cohort Exit

The cohort end date will be offset from index event's start date plus 0 days.

2.3.3 Cohort Eras

Entry events will be combined into cohort eras if they are within 0 days of each other.

2.3.4 Concept set: Fluzone

Concept ID	Concept Name	Code	Vocabulary	Excluded	Descendants	Mapped
1593354	influenza A virus A/Hong Kong/4801/2014 (H3N2) antigen 0.12 MG/ML / influenza A virus A/Michigan/45/2015 (H1N1) antigen 0.12 MG/ML / influenza B virus B/Brisbane/60/2008 antigen 0.12 MG/ML [Fluzone 2017-2018]	1928341	RxNorm	NO	YES	NO|

2.4 Seasonal Flu Vaccines (All)

2.4.1 Cohort Entry Events

People enter the cohort when observing any of the following:

1. drug exposures of 'Seasonal flu vaccine', starting between September 1, 2017 and May 31, 2018.

Limit cohort entry events to the earliest event per person.

2.4.2 Cohort Exit

The cohort end date will be offset from index event's start date plus 0 days.

2.4.3 Cohort Eras

Entry events will be combined into cohort eras if they are within 0 days of each other.

2.4.4 Concept set: Seasonal flu vaccine

Concept ID	Concept Name	Code	Vocabulary	Excluded	Descendants	Mapped
40213145	influenza, injectable, quadrivalent, contains preservative	158	CVX	NO	YES	NO|
42903442	influenza B virus	1312376	RxNorm	NO	YES	NO|
40213150	influenza, live, intranasal, quadrivalent	149	CVX	NO	YES	NO|
40213159	influenza virus vaccine, whole virus	16	CVX	NO	YES	NO|
40225028	influenza virus vaccine, inactivated A-Victoria-210-2009 X-187 (H3N2) (A-Perth-16-2009) strain	1005931	RxNorm	NO	YES	NO|
40213156	influenza virus vaccine, split virus (incl. purified surface antigen)-retired CODE	15	CVX	NO	YES	NO|
40213151	Seasonal, trivalent, recombinant, injectable influenza vaccine, preservative free	155	CVX	NO	YES	NO|
40213327	influenza nasal, unspecified formulation	151	CVX	NO	YES	NO|
40213148	influenza, intradermal, quadrivalent, preservative free, injectable	166	CVX	NO	YES	NO|
40213158	influenza virus vaccine, unspecified formulation	88	CVX	NO	YES	NO|
36878713	Influenza Virus Fragmented, Inactivated, Strain B / Phuket / 3073/2013	OMOP98957	RxNorm Extension	NO	YES	NO|
42873961	influenza B virus vaccine, B-Wisconsin-1-2010-like virus	1303855	RxNorm	NO	YES	NO|
40225038	influenza virus vaccine, live attenuated, A-Perth-16-2009 (H3N2) strain	1005911	RxNorm	NO	YES	NO|
40213146	Influenza, injectable, quadrivalent, preservative free	150	CVX	NO	YES	NO|

40213143	Influenza, injectable, Madin Darby Canine Kidney, preservative free, quadrivalent	171	CVX	NO	YES	NO|
36879025	Influenza Virus Surface Antigens, strain A / Switzerland / 9715293/2013 H3N2 - Analogue Strain Nib-88	OMOP991645	RxNorm Extension	NO	YES	NO|
40213157	Seasonal trivalent influenza vaccine, adjuvanted, preservative free	168	CVX	NO	YES	NO|
45776076	influenza A virus vaccine, A-Texas-50-2012 (H3N2)-like virus	1541617	RxNorm	NO	YES	NO|
40213149	influenza virus vaccine, live, attenuated, for intranasal use	111	CVX	NO	YES	NO|
40213147	Influenza, injectable,quadrivalent, preservative free, pediatric	161	CVX	NO	YES	NO|
40213152	Seasonal, quadrivalent, recombinant, injectable influenza vaccine, preservative free	185	CVX	NO	YES	NO|
42903441	influenza A virus	1312375	RxNorm	NO	YES	NO|
40213141	influenza, high dose seasonal, preservative-free	135	CVX	NO	YES	NO|
40213153	Influenza, seasonal, injectable	141	CVX	NO	YES	NO|
40213144	Influenza, injectable, Madin Darby Canine Kidney, quadrivalent with preservative	186	CVX	NO	YES	NO|
40213142	Influenza, injectable, Madin Darby Canine Kidney, preservative free	153	CVX	NO	YES	NO|
40213155	seasonal influenza, intradermal, preservative free	144	CVX	NO	YES	NO|
40164828	influenza B virus vaccine B/Brisbane/60/2008 antigen	857921	RxNorm	NO	YES	NO|

2.5 HPV Vaccines

2.5.1 Cohort Entry Events

People enter the cohort when observing any of the following:

1. drug exposures of ‘Gardasil 9’, starting between January 1, 2018 and December 31, 2018.

2.5.2 Cohort Exit

The cohort end date will be offset from index event’s start date plus 0 days.

2.5.3 Cohort Eras

Entry events will be combined into cohort eras if they are within 0 days of each other.

2.5.4 Concept set: Gardasil 9

Concept ID	Concept Name	Code	Vocabulary	Excluded	Descendants	Mapped
36248866	Gardasil 9 Injectable Product	1597098	RxNorm	NO	YES	NO|
45892513	L1 protein, human papillomavirus type 11 vaccine / L1 protein, human papillomavirus type 16 vaccine / L1 protein, human papillomavirus type 18 vaccine / L1 protein, human papillomavirus type 31 vaccine / L1 protein, human papillomavirus type 33 vaccine /	1597102	RxNorm	NO	YES	NO|
45892514	0.5 ML L1 protein, human papillomavirus type 11 vaccine 0.08 MG/ML / L1 protein, human papillomavirus type 16 vaccine 0.12 MG/ML / L1 protein, human papillomavirus type 18 vaccine 0.08 MG/ML / L1 protein, human papillomavirus type 31 vaccine 0.04 MG/ML /	1597103	RxNorm	NO	YES	NO|
45892510	0.5 ML L1 protein, human papillomavirus type 11 vaccine 0.08 MG/ML / L1 protein, human papillomavirus type 16 vaccine 0.12 MG/ML / L1 protein, human papillomavirus type 18 vaccine 0.08 MG/ML / L1 protein, human papillomavirus type 31 vaccine 0.04 MG/ML /	1597099	RxNorm	NO	YES	NO|
40213322	Human Papillomavirus 9-valent vaccine	165	CVX	NO	YES	NO|

2.6 Zoster Vaccines

2.6.1 Cohort Entry Events

People enter the cohort when observing any of the following:

1. drug exposures of ‘Shingrix’, starting between January 1, 2018 and December 31, 2018.

2.6.2 Cohort Exit

The cohort end date will be offset from index event’s start date plus 0 days.

2.6.3 Cohort Eras

Entry events will be combined into cohort eras if they are within 0 days of each other.

2.6.4 Concept set: Shingrix

Concept ID	Concept Name	Code	Vocabulary	Excluded	Descendants	Mapped
792784	varicella zoster virus glycoprotein E Injection [Shingrix]	1986828	RxNorm	NO	YES	NO|
792783	varicella zoster virus glycoprotein E, recombinant 0.1 MG/ML [Shingrix]	1986827	RxNorm	NO	YES	NO|
792788	varicella zoster virus glycoprotein E, recombinant 0.1 MG/ML Injection [Shingrix]	1986832	RxNorm	NO	YES	NO|
36421491	Varicella-Zoster Virus Vaccine Live (Oka-Merck) strain Injectable Solution [Shingrix]	OMOP4763774	RxNorm Extension	NO	YES	NO|
792785	Shingrix Injectable Product	1986829	RxNorm	NO	YES	NO|
706103	zoster vaccine recombinant	187	CVX	NO	YES	NO|

3 Negative control outcomes

Negative controls are outcomes believed not to be caused by any of the vaccines, and therefore ideally would not be flagged as a signal by a safety surveillance system. Any effect size estimates for negative control ideally should be close to the null.

A single set of negative control outcomes is defined for all four vaccine groups. To identify negative control outcomes that match the severity and prevalence of suspected vaccine adverse effects, a candidate list of negative controls was generated based on similarity of prevalence and percent of diagnoses that were recorded in an inpatient setting (as a proxy for severity). Manual review of this list by clinical experts created the final list of 93 negative control outcomes. The full list of negative control outcomes can be found in Table 8. The outcome ID is the concept ID in the OMOP Vocabulary, which can be mapped to the specific code systems used in the various databases.

Table 8: Negative control outcomes.

Outcome Id	Outcome Name
438945	Accidental poisoning by benzodiazepine-based tranquilizer
434455	Acquired claw toes
316211	Acquired spondylolisthesis
201612	Alcoholic liver damage
438730	Alkalosis
441258	Anemia in neoplastic disease
432513	Animal bite wound
4171556	Ankle ulcer
4098292	Antiphospholipid syndrome
77650	Aseptic necrosis of bone
4239873	Benign neoplasm of ciliary body
23731	Benign neoplasm of larynx
199764	Benign neoplasm of ovary
195500	Benign neoplasm of uterus
4145627	Biliary calculus
4108471	Burn of digit of hand
75121	Burn of lower leg
4284982	Calculus of bile duct without obstruction
434327	Cannabis abuse
78497	Cellulitis and abscess of toe
4001454	Cervical spine ankylosis
4068241	Chronic instability of knee
195596	Chronic pancreatitis
4206338	Chronic salpingitis
4058397	Claustrophobia
74816	Contusion of toe
73302	Curvature of spine
4151134	Cyst of pancreas
77638	Displacement of intervertebral disc without myelopathy
195864	Diverticulum of bladder
201346	Edema of penis
200461	Endometriosis of uterus
377877	Esotropia
193530	Follicular cyst of ovary
4094822	Foreign body in respiratory tract
443421	Gallbladder and bile duct calculi

4299408	Gouty tophus
135215	Hashimoto thyroiditis
442190	Hemorrhage of colon
43020475	High risk heterosexual behavior
194149	Hirschsprung's disease
443204	Human ehrlichiosis
4226238	Hyperosmolar coma due to diabetes mellitus
4032787	Hyperosmolarity
197032	Hyperplasia of prostate
140362	Hypoparathyroidism
435371	Hypothermia
138690	Infestation by Pediculus
4152376	Intentional self poisoning
192953	Intestinal adhesions with obstruction
196347	Intestinal parasitism
137977	Jaundice
317510	Leukemia
765053	Lump in right breast
378165	Nystagmus
434085	Obstruction of duodenum
4147016	Open wound of buttock
4129404	Open wound of upper arm
438120	Opioid dependence
75924	Osteodystrophy
432594	Osteomalacia
30365	Panhypopituitarism
4108371	Peripheral gangrene
440367	Plasmacytosis
439233	Poisoning by antidiabetic agent
442149	Poisoning by bee sting
4314086	Poisoning due to sting of ant
4147660	Postural kyphosis
434319	Premature ejaculation
199754	Primary malignant neoplasm of pancreas
4311499	Primary malignant neoplasm of respiratory tract
436635	Primary malignant neoplasm of sigmoid colon
196044	Primary malignant neoplasm of stomach
433716	Primary malignant neoplasm of testis
133424	Primary malignant neoplasm of thyroid gland
194997	Prostatitis
80286	Prosthetic joint loosening
443274	Psychostimulant dependence
314962	Raynaud's disease
37018294	Residual osteitis
4288241	Salmonella enterica subspecies arizonae infection
45757269	Sclerosing mesenteritis
74722	Secondary localized osteoarthrosis of pelvic region
200348	Secondary malignant neoplasm of large intestine
43020446	Sedative withdrawal
74194	Sprain of spinal ligament
4194207	Tailor's bunion
193521	Tropical sprue
40482801	Type II diabetes mellitus uncontrolled
74719	Ulcer of foot
196625	Viral hepatitis A without hepatic coma
197494	Viral hepatitis C
4284533	Vitamin D-dependent rickets

Negative control outcomes are defined as the first occurrence of the negative control concept or any of its descendants.

Table 9: Committed EUMAEUS data sources and the populations they cover.

Data source	Population	Data capture process and short description
**Administrative claims**		
IBM MarketScan Commercial Claims and Encounters (CCAE)	Commercially insured, younger than 65 years	Adjudicated health insurance claims (e.g. inpatient, outpatient, and outpatient pharmacy) from large employers and health plans who provide private healthcare coverage to employees, their spouses and dependents.
IBM MarketScan Medicare Supplemental Database (MDCR)	Commercially insured, 65 years and older	Adjudicated health insurance claims of retirees with primary or Medicare supplemental coverage through privately insured fee-for-service, point-of-service or capitated health plans.
IBM MarketScan Multi-State Medicaid Database (MDCD)	Medicaid enrollees, racially diverse	Adjudicated health insurance claims for Medicaid enrollees from multiple states and includes hospital discharge diagnoses, outpatient diagnoses and procedures, and outpatient pharmacy claims.
**Electronic health records (EHRs)**		
Optum Electronic Health Records (OptumEHR)	US, general	Clinical information, prescriptions, lab results, vital signs, body measurements, diagnoses and procedures derived from clinical notes using natural language processing.

4 Data sources

Table 9 lists the five data sources included in EUMAEUS; these sources encompass a large variety of practice types and populations. For each data source, we report a brief description and size of the population it represents. All data sources have received institutional review board approval or exemption for their participation before executing EUMAEUS.

```
## Rows: 4 Columns: 3
## -- Column specification --------------------------------------------------------
## Delimiter: ";"
## chr (3): Data source, Population, Data capture process and short description
##
## i Use `spec()` to retrieve the full column specification for this data.
## i Specify the column types or set `show_col_types = FALSE` to quiet this message.
```

4.1 Database characteristics

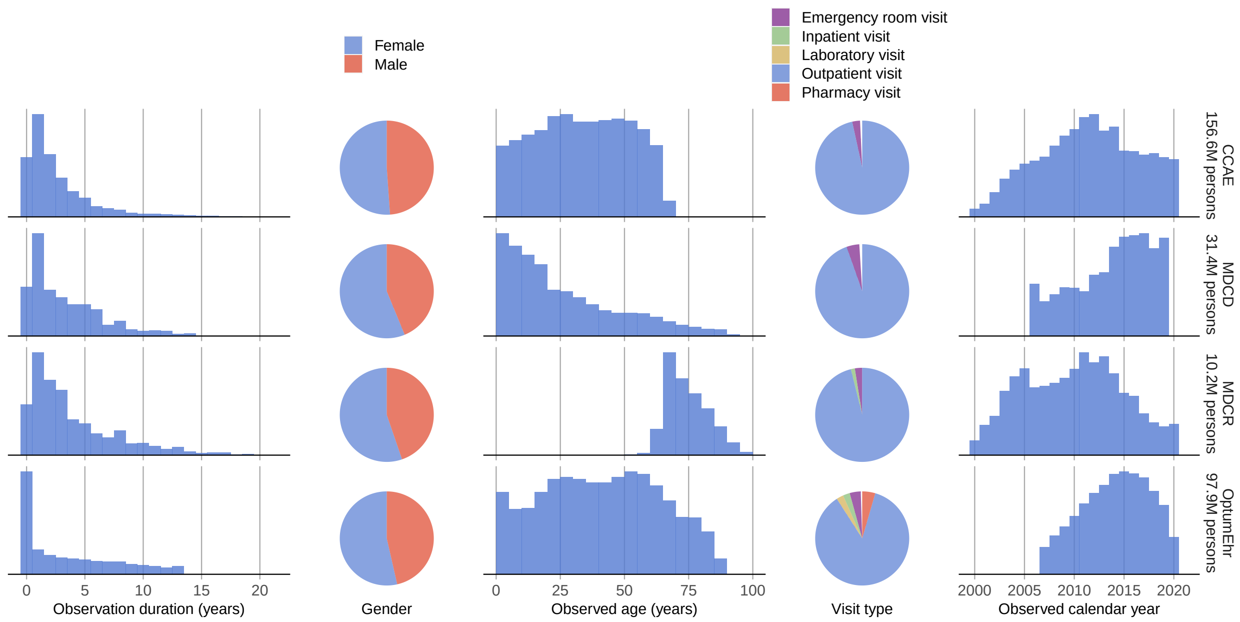

Figure 1: Overall distributions of key characteristics in each database.

5 Evaluated Methods

Vaccine safety surveillance methods can be broken down in to four components: construction of a *counterfactual* (often referred to as the ‘expected count’), a *time-at-risk*, the *statistic* to estimate, and potentially a *decision rule* on the estimate to classify signals from non-signals.

5.1 Counterfactual construction

Historic rates

Traditionally, vaccine surveillance methods compute an expected count based an incidence rate estimated during some historic time period, for example in the years prior to the initiation of the surveillance study. We use the historic period indicated in Table 1. We evaluate four variations:

- Unadjusted, entire year. Using a single rate computed across the entire historic year for the entire population.
- Age and sex adjusted, entire year. Using a rate stratifying by age (in 5 year increments) and sex, computed across the entire historic year. This allows the expected rate to be adjusted for the demographics of the vaccinated.
- Unadjusted, time-at-risk relative to outpatient visit. Using a single rate computed during the time-at-risk relative to a random outpatient visit in the historic year.
- Age and sex adjusted, time-at-risk relative to outpatient visit. Using a rate stratifying by age and sex, computed during the time-at-risk relative to a random outpatient visit in the historic year.

Initial results showed that this counterfactual approach is sensitive to changes in coding practices. We therefore introduce a study diagnostic: the percent change in overall incidence rate (across the entire population) between the historic and current time period. For each of the four variations listed above, we add a new variation where effect-size estimates are removed if the change in incidence rate is greater than 50%.

Cohort method using a contemporary non-user comparator

A comparator cohort study most closely emulates a randomized clinical trial, comparing the target cohort (those vaccinated) to some comparator cohort. We define two types of non-user comparator cohort, one having an outpatient visit on the index date, and another having a random date as the index date. For both comparator variants we exclude subjects having a vaccinations for the same disease as the target vaccine on or before the index date. When doing unadjusted comparisons, the comparator cohort is a random sample of equal size as the target cohort. When doing propensity score (PS) adjusted comparisons,

the comparator cohort is a stratified (by age and sex) random sample of four times the size of the target cohort (two times for the Seasonal Flu Vaccination (all) target cohort for computational reasons). Propensity models use a large generic set of covariates, including demographics and covariates per drug, condition, procedure, measurement, etc., and are fitted using large-scale regularized regression as described previously. [1] We evaluate 10 method variations:

Anchoring the comparator on a random outpatient visit:

- Unadjusted comparison.
- 1-on-1 PS matching.
- PS stratification. Five equally-sized strata are defined in the target (vaccinated) population.
- Inverse Probability of Treatment Weighting (IPTW). We use stabilized weights to compute the average treatment effect in the treated (ATT). Weights are truncated to a maximum value of 10, similar to Izurieta et al. (2020). [2]
- 1-on-1 PS matching within each period, using only the 'new' data in that period to fit the propensity model. Once a population is matched in a period, that matching is carried forward to subsequent periods. This method is only evaluated using the H1N1pdm vaccinations for computational reasons.

Anchoring the comparator on a random date:

- Unadjusted comparison.
- 1-on-1 PS matching.
- PS stratification.
- IPTW with trimming.
- 1-on-1 PS matching within each period. This method is only be evaluated using the H1N1pdm vaccinations for computational reasons.

Self-Controlled Case Series (SCCS) / Self-Controlled Risk Interval (SCRI)

The SCCS and SCRI designs are self-controlled, comparing the time-at-risk (the time shortly following the vaccination) to some other time in the same patient's record. The SCCS design uses all patient time when not at risk as the control time. [3] The SCRI design uses a pre-specified control interval relative to the vaccination date as the control time. [4] This unexposed time can be both before or after the time at risk. We evaluate five variations:

- A simple SCCS, using all patient time when not at risk as the control time, with the exception of the 30 days prior to vaccination which is excluded from the analysis to avoid bias due to contra-indications.

- An SCCS adjusting for age and season. Age and season are modeled to be constant within each calendar month, and vary across months as bicubic splines.
- A simple SCCS discarding all time prior to vaccination.
- An SCRI, using a control interval of 43 to 15 days prior to vaccination.
- An SCRI, using a control interval of 43 to 71 days after to vaccination.

Case-control

The case-control design compares cases (those with the outcome) to controls (those that do not have the outcome), and looks back in time for exposures to a vaccine. We evaluate two variants:

- Using up to four age and sex matched controls per case. For age we use a two-year caliper.
- By sampling controls from the general non-case population, and adjusting for age and sex in the outcome model. The control sample is be four times the number of controls. Age is modeled as one variable per 5-year age category.

5.2 Time-at-risk

The time-at-risk is the time window, relative to the vaccination date, when outcomes will potentially be attributed to the vaccine. We define the time-at-risk window top be 1-28 days after vaccination. Time-at-risk windows are constructed both for the first and second dose. The time-at-risk for one dose is censored at the time of the next dose.

5.3 Statistic

- Effect-size estimate. Each method can be used to produce an effect-size estimates such as a hazard ratio, incidence rate ratio, or odds ratio. For example, when using a historic rate we can compute the observed to expected ratio, which can be interpreted as the incidence rate ratio.

- Log likelihood ratio (LLR). A common practice in vaccine safety surveillance is to computer the LLR, which is the log of the ratio between the likelihood of the alternative hypothesis (that there is an effect) and the likelihood of the null hypothesis (of no effect). The LLR is a convenient statistic when performing sequential testing, where the LLR can be compared to a pre-computed critical value, as is done in the MaxSPRT method. [5] Although typically MaxSPRT uses a historic rate as counterfactual, any counterfactual can be used to compute the LLR and can be used in MaxSPRT.

Effect-size estimates are computed both with and without empirical calibration. [6,7] Empirical calibration is done using leave-one-out: when calibrating the estimate for a control, the systematic error distribution is fitted uses all controls except the one being calibrated.

5.4 Decision rule

To identify ‘signals’ we need a decision rule, for example in the shape of a threshold value on one of the estimates statistics. In our experiment we consider one decision rule, which is the critical value computed for the LLR at an alpha of 0.05. For the historical rates method we use a Poisson model assuming the counterfactual is known without uncertainty. For all other methods we use a binomial model. All critical values are computed using the `Sequential` package in CRAN.

6 Fitted systematic error distribution

For each method variation and vaccine group, the systematic error distribution fitted on the negative control estimates are shown. The red area indicates the maximum likelihood estimates of the distribution parameters, the pink area indicates the 95% credible interval.

6.1 Systematic error distributions in the Optum EHR database

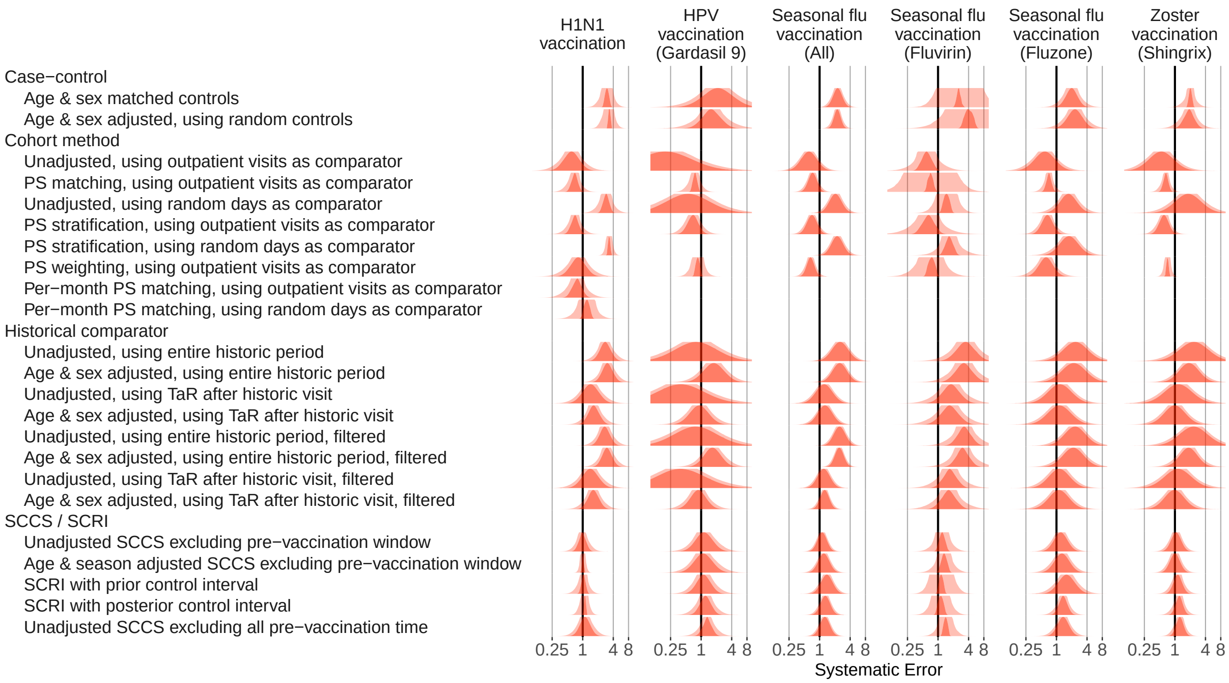

Figure 2: Fitted systematic error distributions for the Optum EHR database.

6.2 Systematic error distributions in the MDCD database

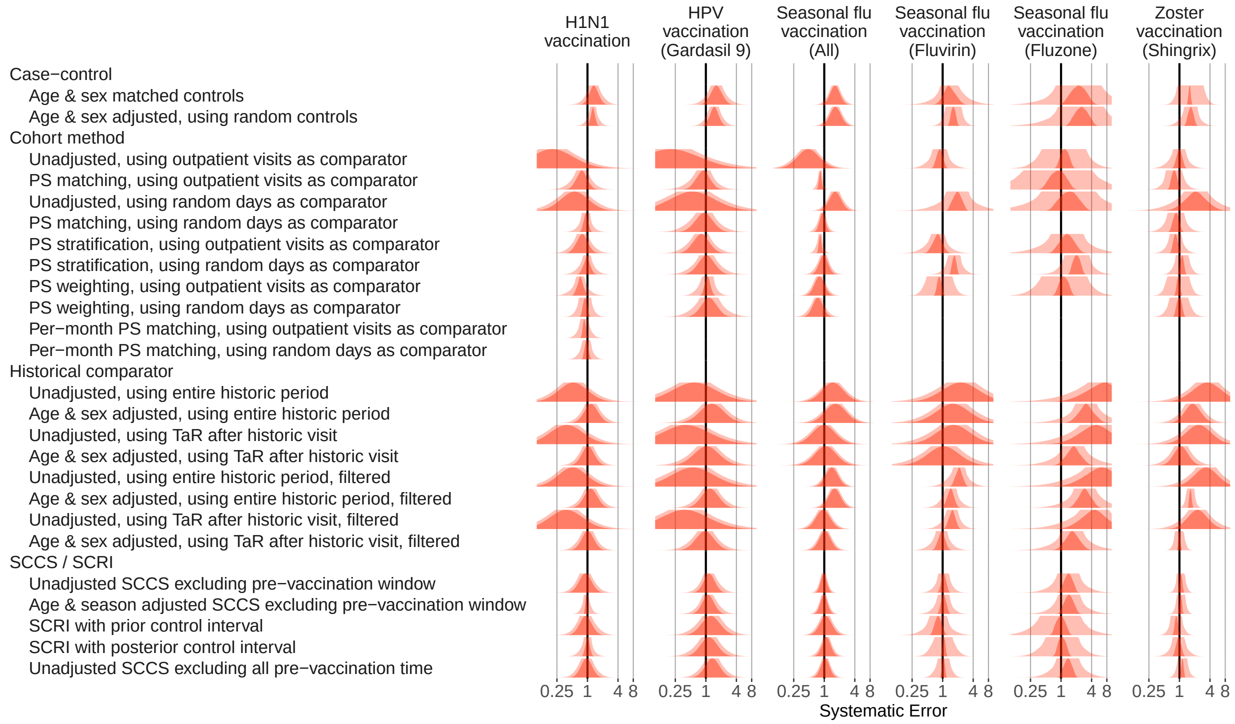

Figure 3: Fitted systematic error distributions for the MDCD database.

6.3 Systematic error distributions in the MDCR database

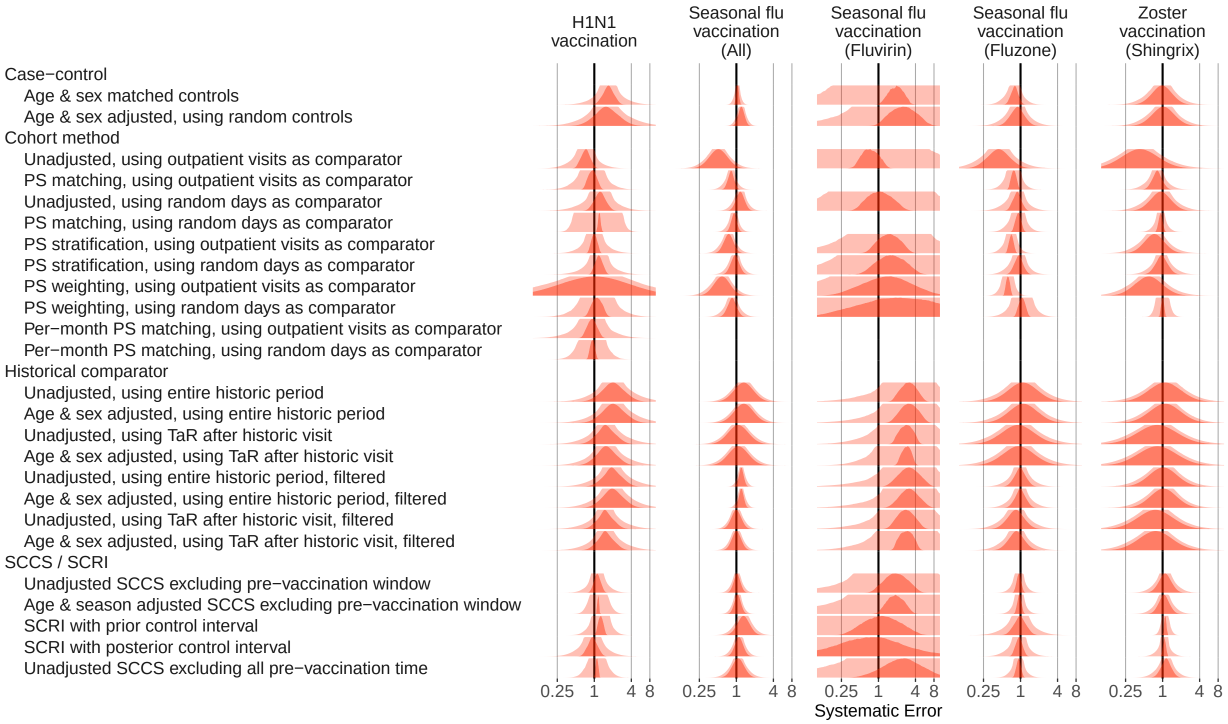

Figure 4: Fitted systematic error distributions for the MDCR database.

6.4 Systematic error distributions in the CCAE database

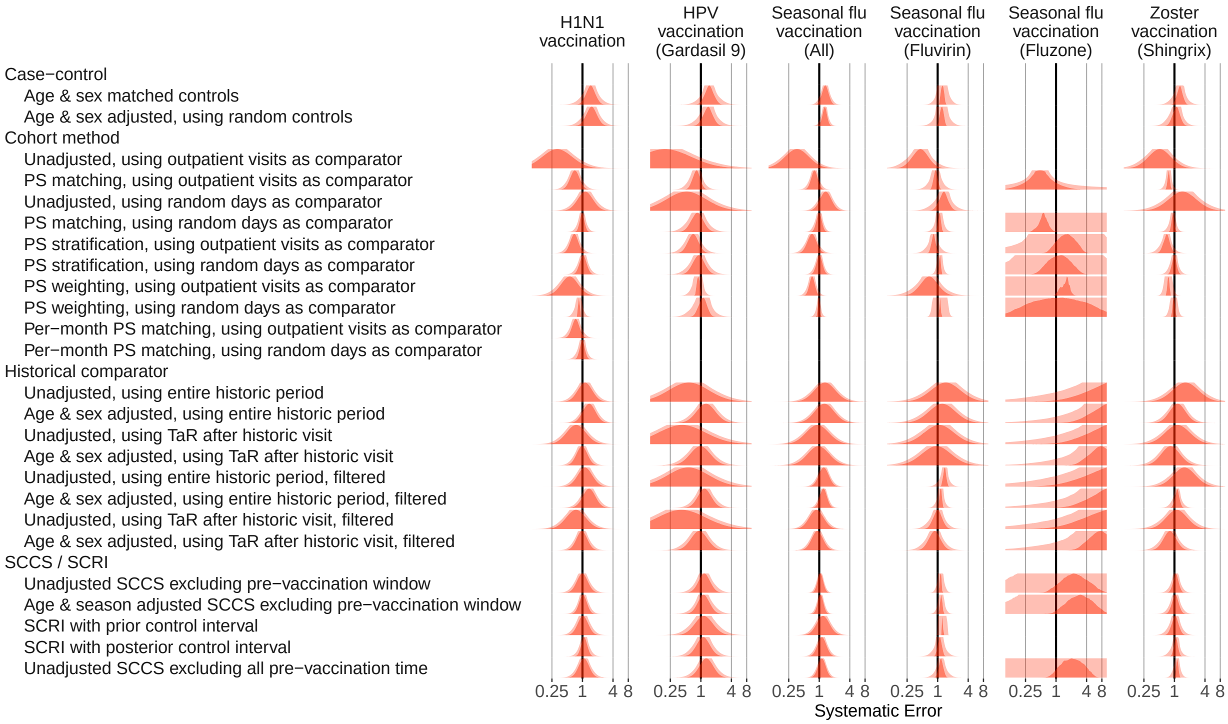

Figure 5: Fitted systematic error distributions for the CCAE database.

7 Type 1 and 2 error before and after calibration

For each method variation and vaccine group, the type 1 and 2 error before and after empirical calibration are shown. The x-axis indicates the type 1 error (higher values to the left) and type 2 error (higher values to the right), based on the (calibrated) one-sided p-value. The dashed line indicates nominal type 1 error of 5%.

7.1 Type 1 and 2 error before and after calibration in the Optum EHR database

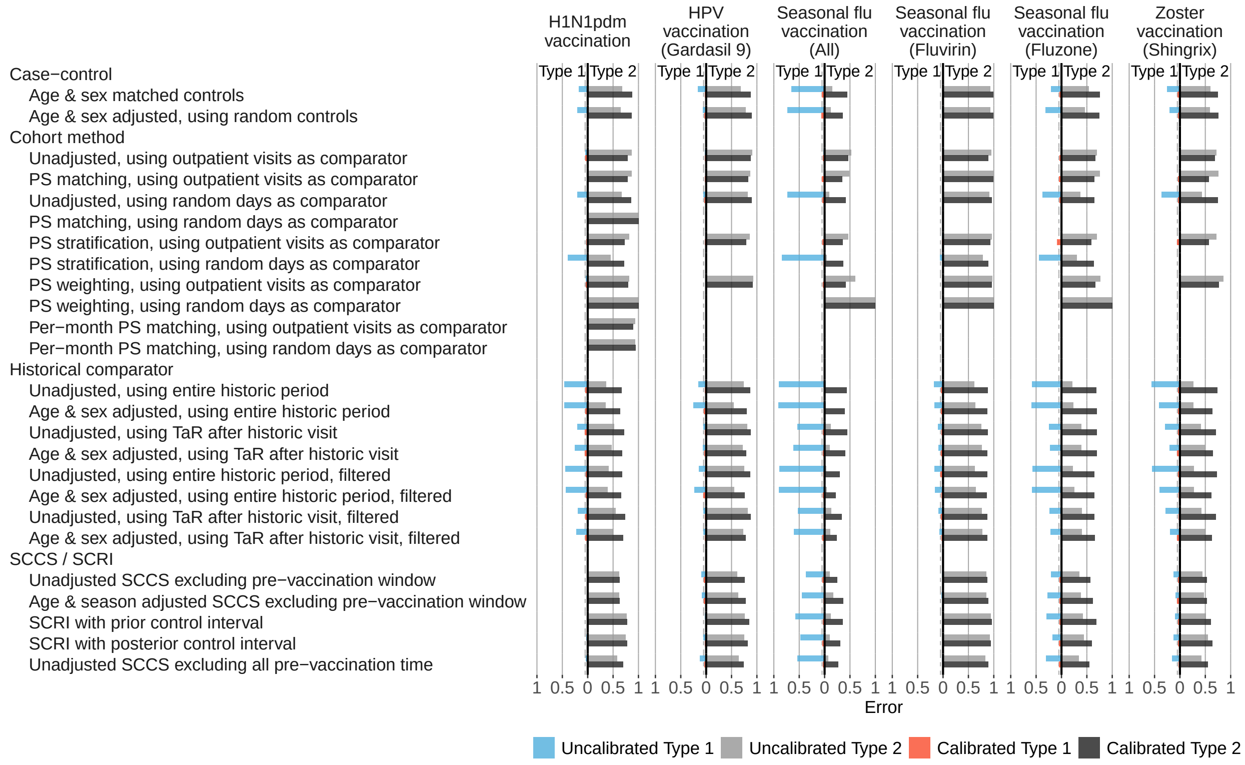

Figure 6: Type 1 and 2 error before and after empirical calibration in the Optum EHR database.

7.2 Type 1 and 2 error before and after calibration in the MDCD database

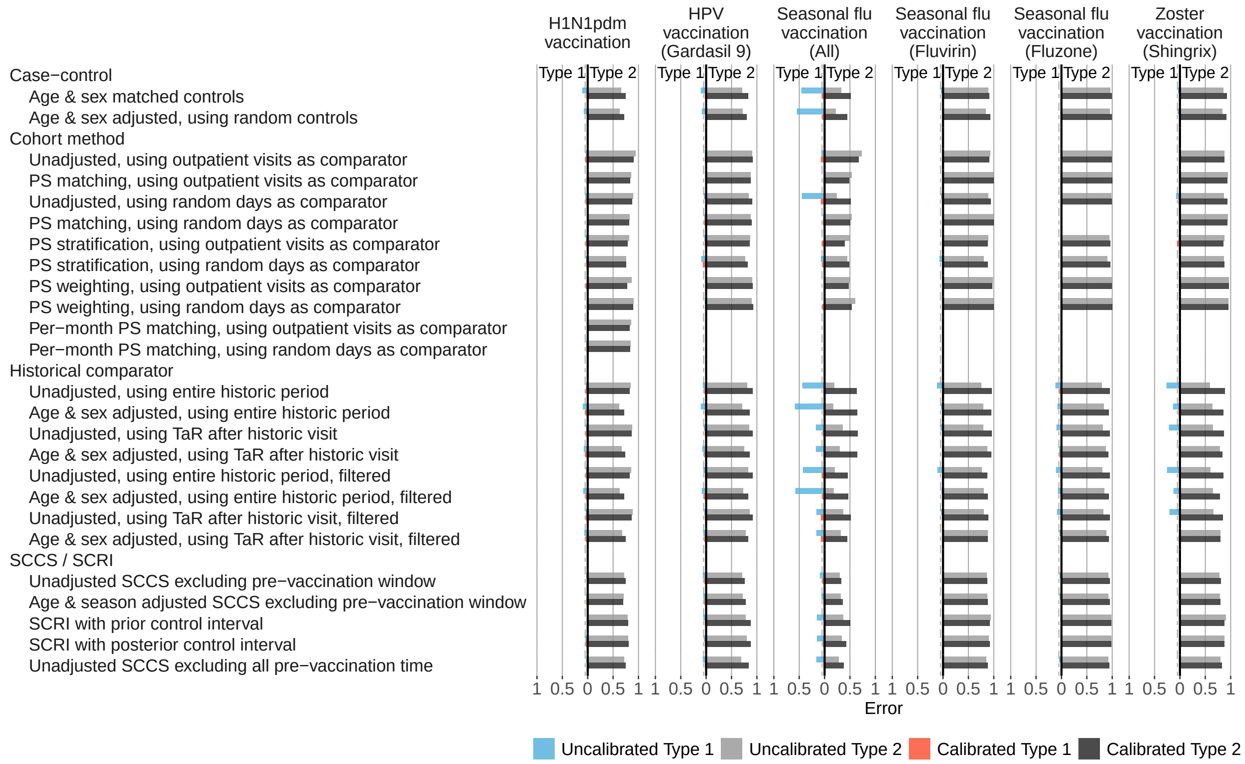

Figure 7: Type 1 and 2 error before and after empirical calibration in the MDCD database.

7.3 Type 1 and 2 error before and after calibration in the MDCR database

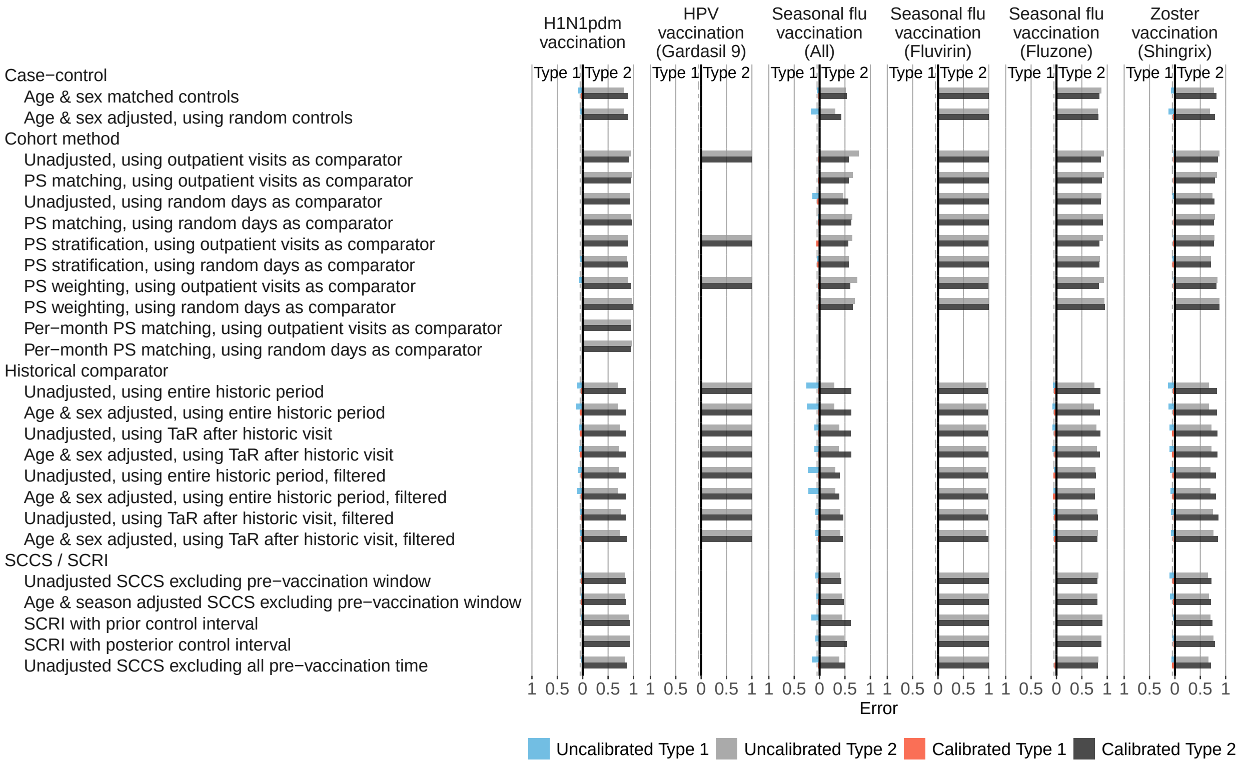

Figure 8: Type 1 and 2 error before and after empirical calibration in the MDCR database.

7.4 Type 1 and 2 error before and after calibration in the CCAE database

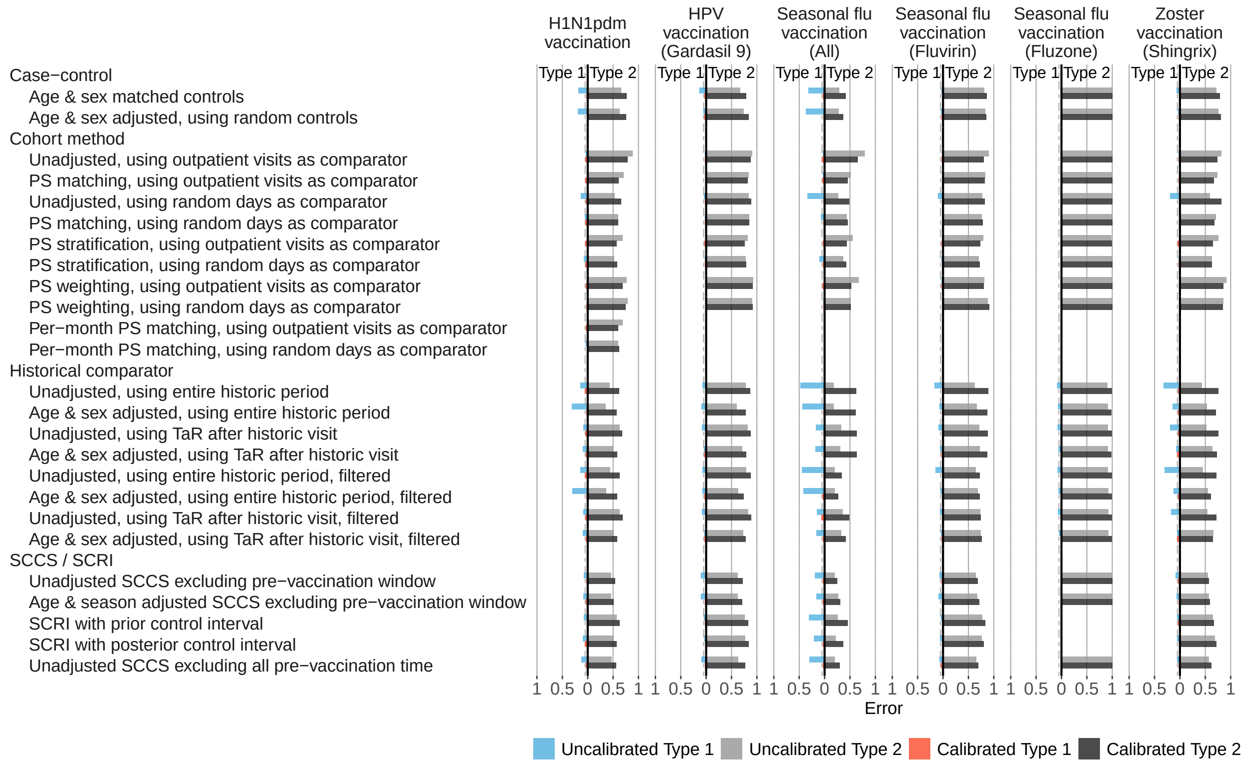

Figure 9: Type 1 and 2 error before and after empirical calibration in the CCAE database.

8 Time to 50% sensitivity

For each method variation and vaccine group, the number of months of data needed to achieve 50% sensitivity based on the calibrated MaxSPRT are shown, stratified by true effect size of the positive controls.

8.1 Time to 50% sensitivity in the Optum EHR database

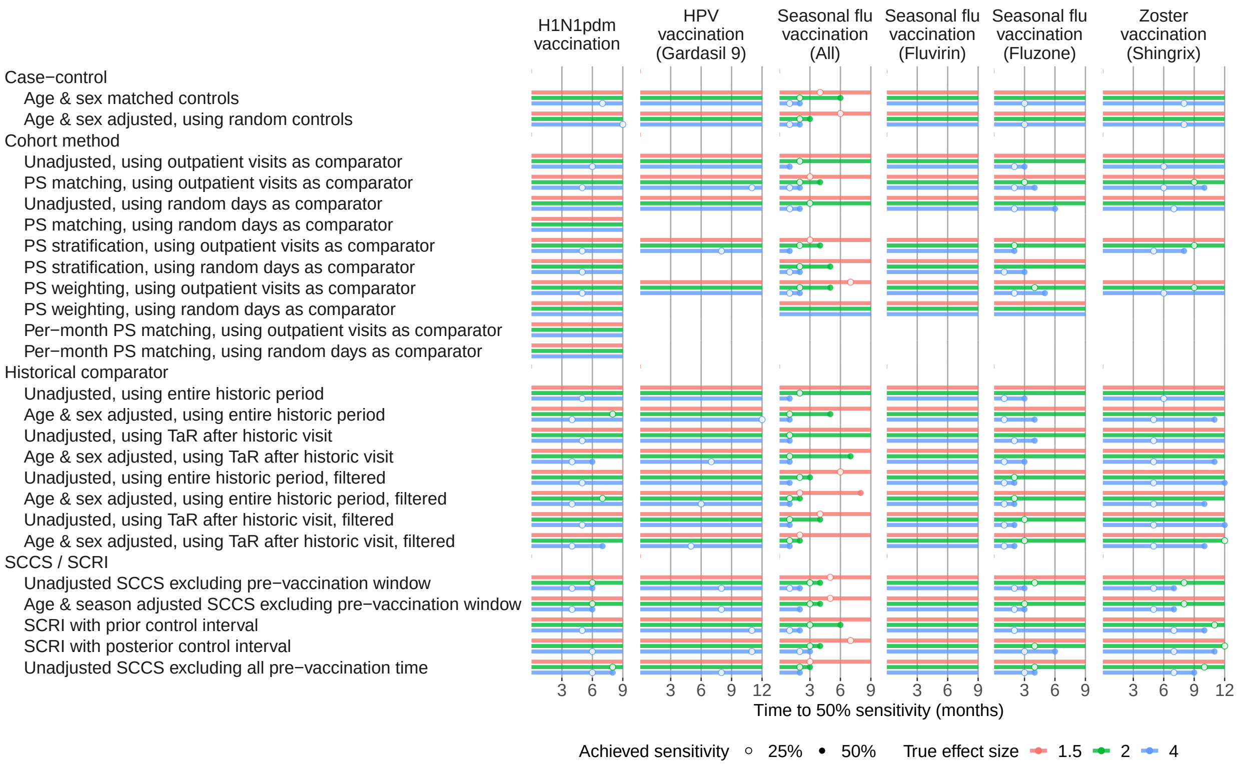

Figure 10: Time to 50% sensitivity in the Optum EHR database.

8.2 Time to 50% sensitivity in the MDCD database

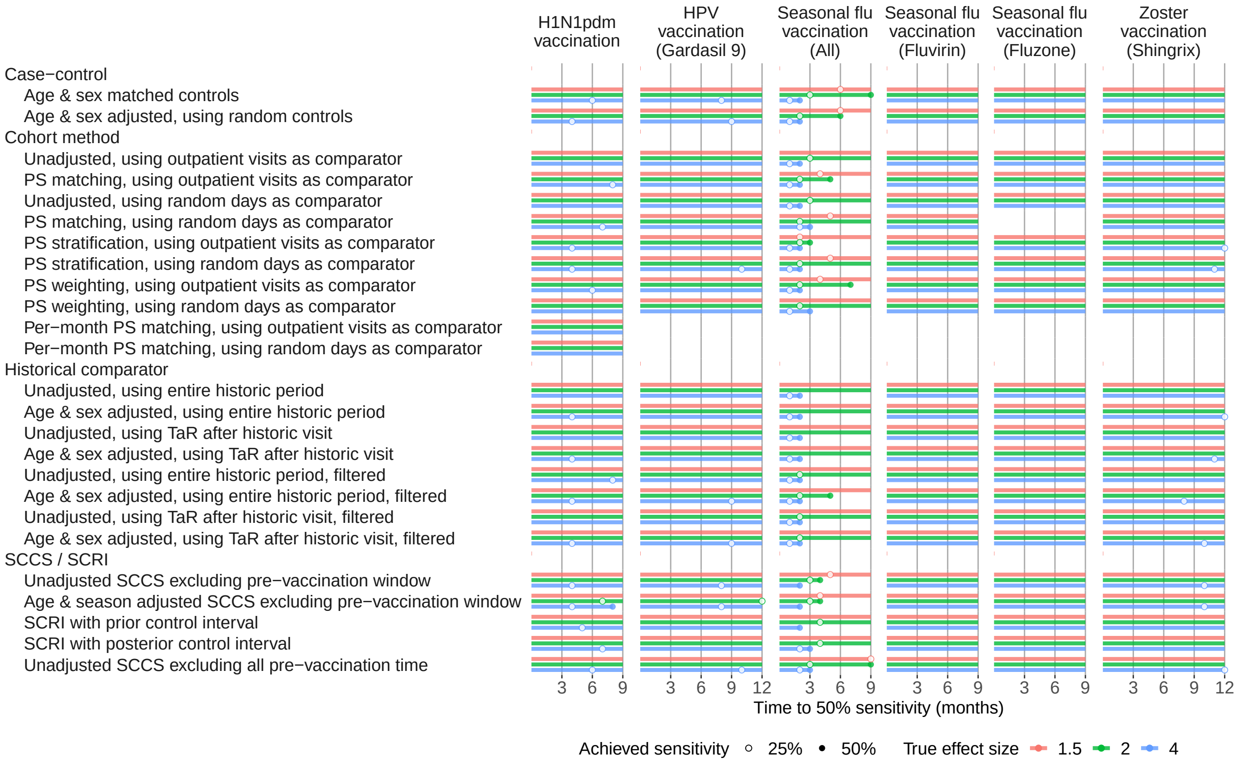

Figure 11: Time to 50% sensitivity in the MDCD database.

8.3 Time to 50% sensitivity in the MDCR database

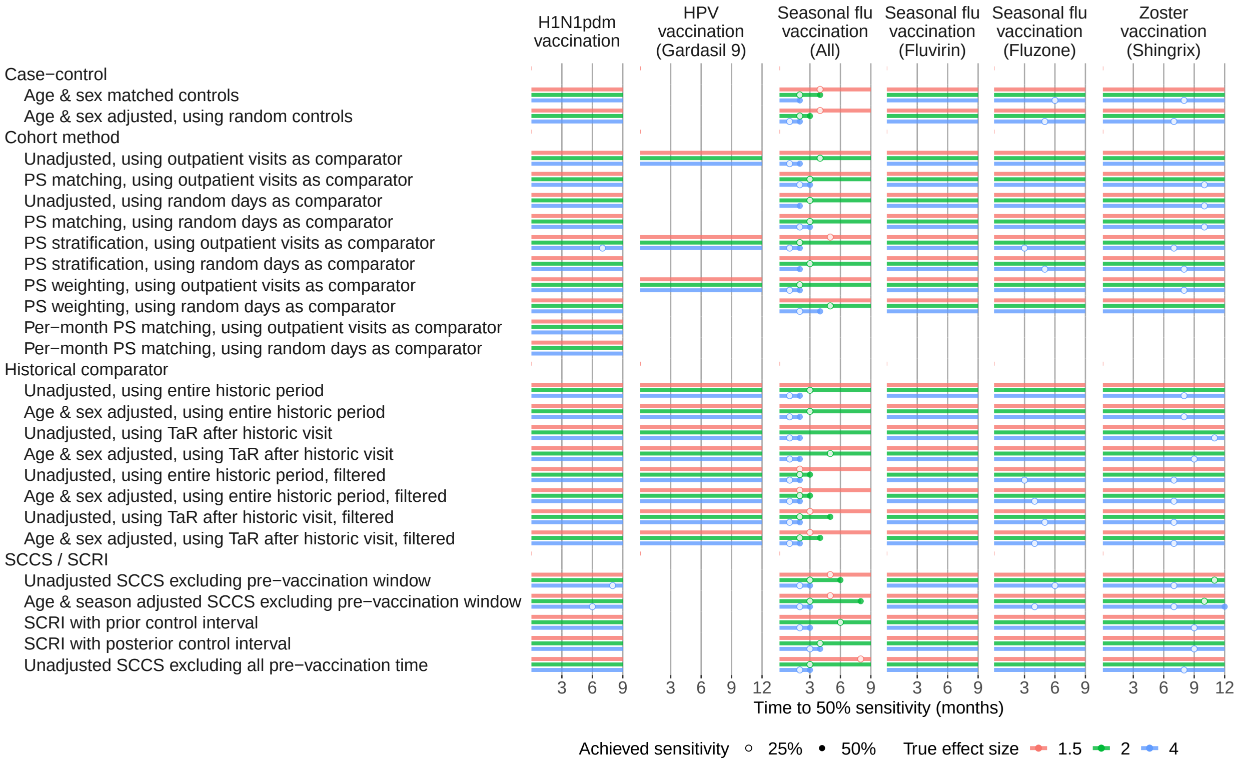

Figure 12: Time to 50% sensitivity in the MDCR database.

8.4 Time to 50% sensitivity in the CCAE database

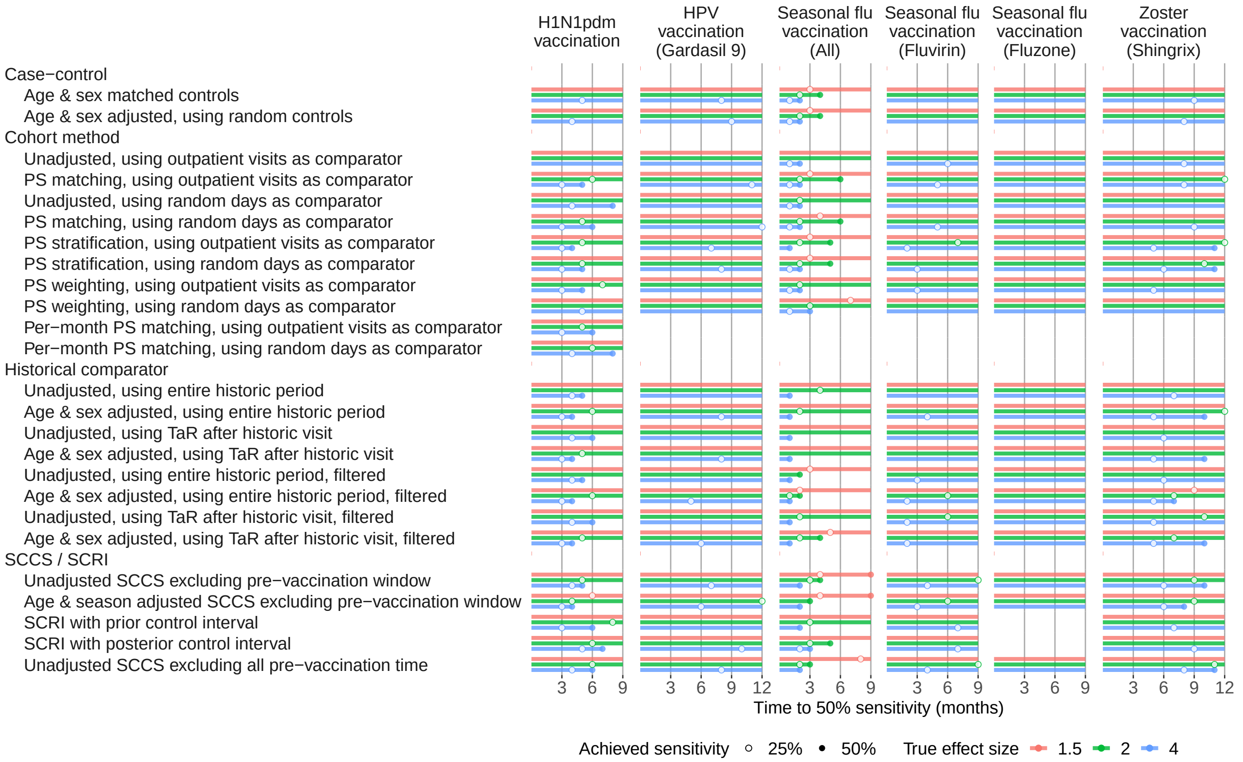

Figure 13: Time to 50% sensitivity in the CCAE database.

9 Time to 80% sensitivity

For each method variation and vaccine group, the number of months of data needed to achieve 80% sensitivity based on the calibrated MaxSPRT are shown, stratified by true effect size of the positive controls.

9.1 Time to 80% sensitivity in the Optum EHR database

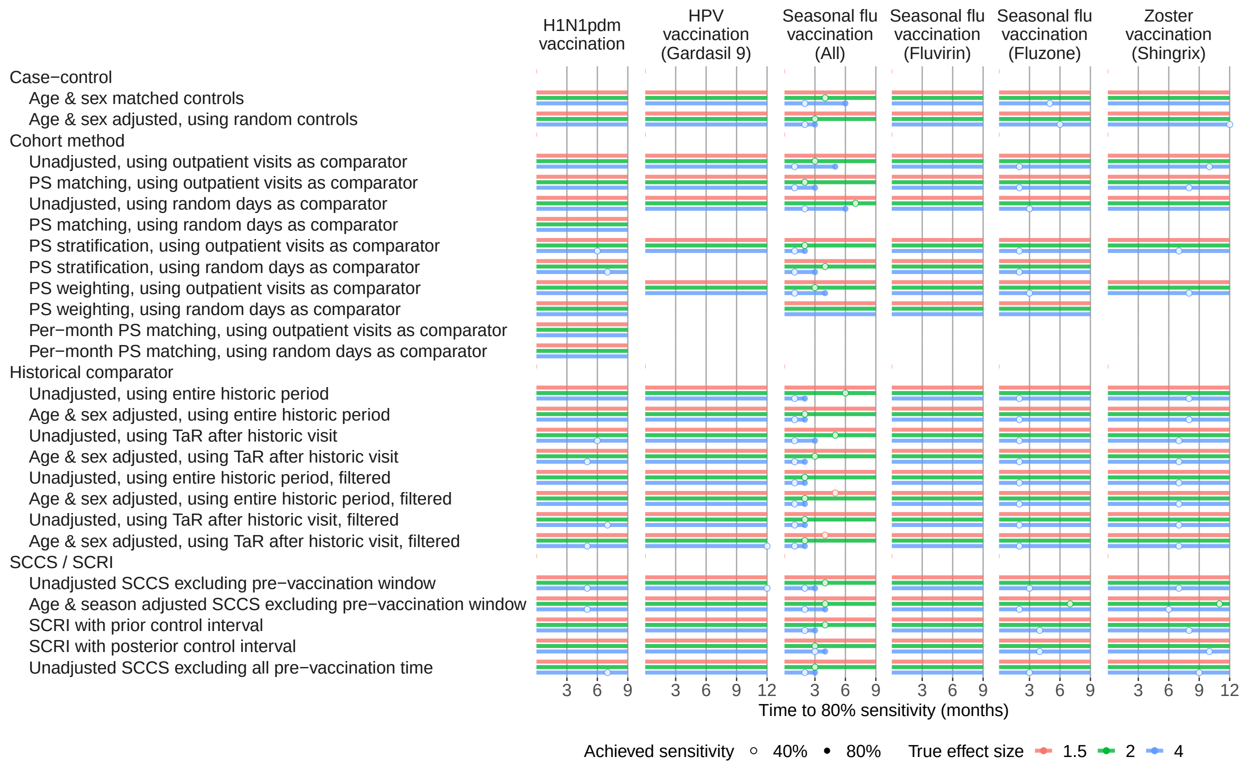

Figure 14: Time to 80% sensitivity in the Optum EHR database.

9.2 Time to 80% sensitivity in the MDCD database

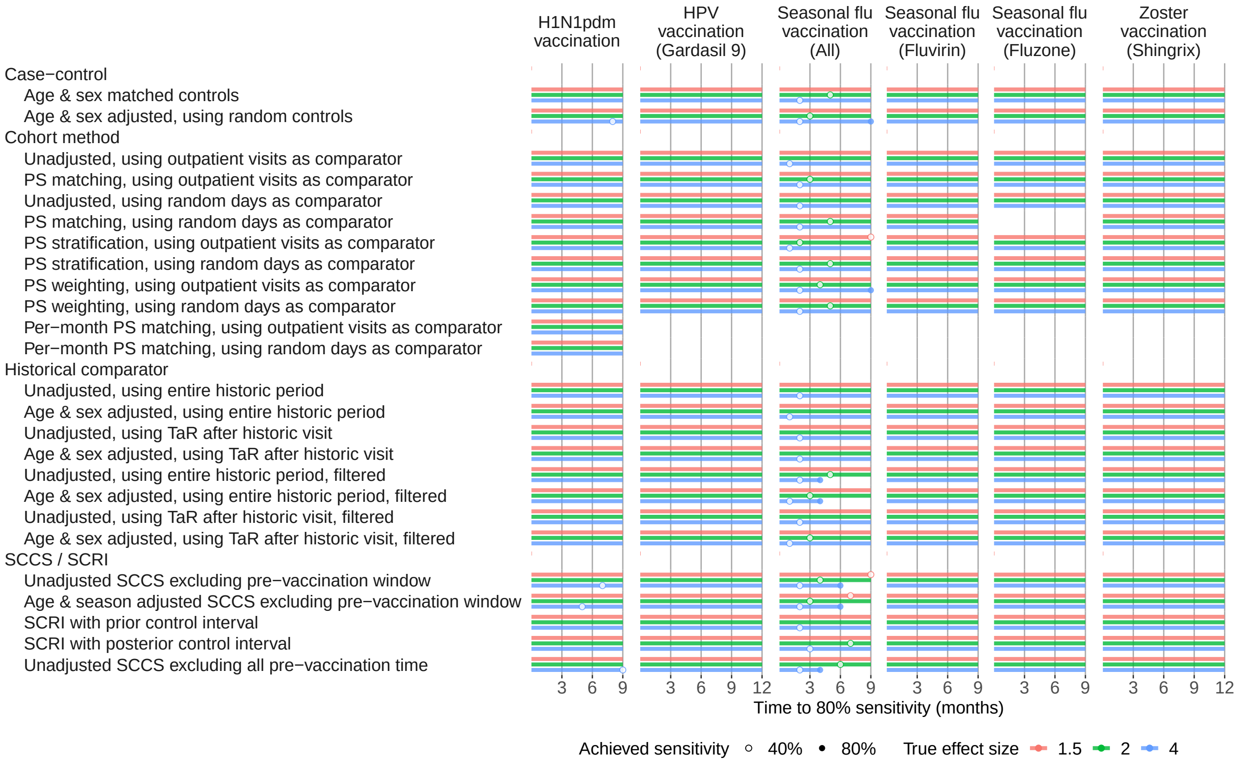

Figure 15: Time to 80% sensitivity in the MDCD database.

9.3 Time to 80% sensitivity in the MDCR database

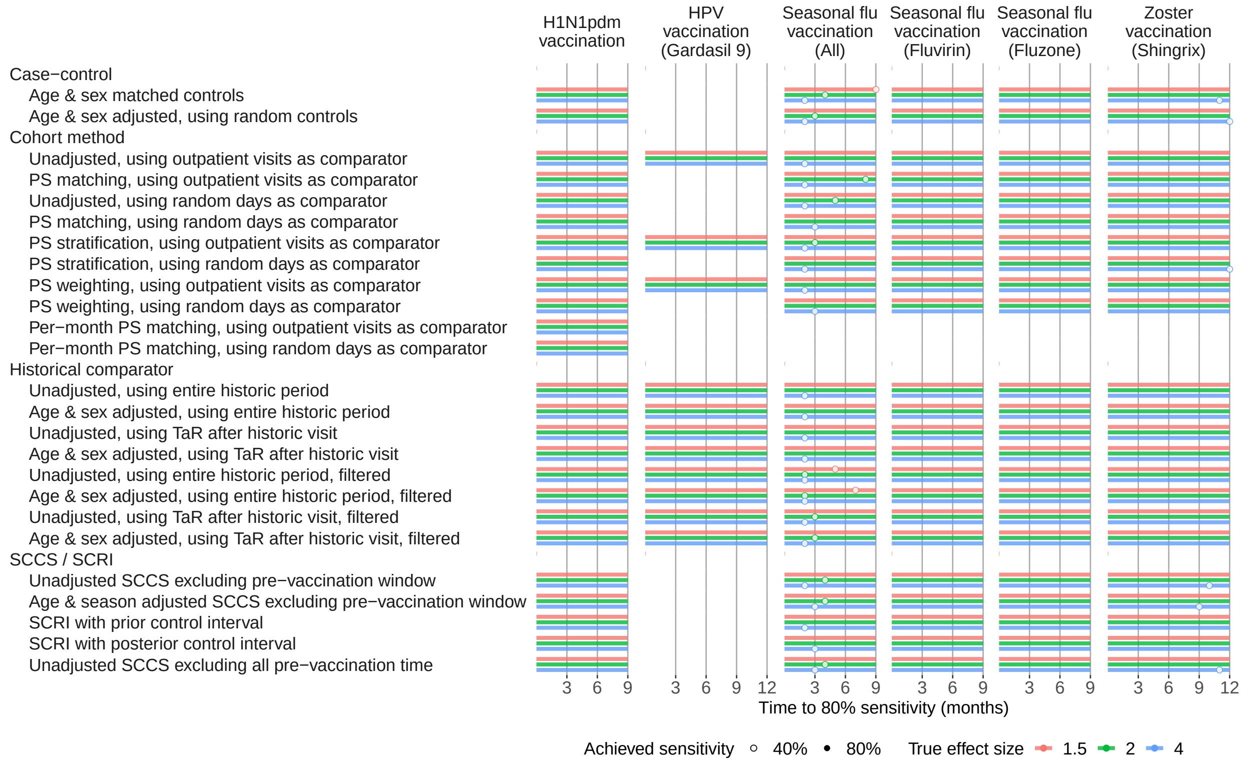

Figure 16: Time to 80% sensitivity in the MDCR database.

9.4 Time to 80% sensitivity in the CCAE database

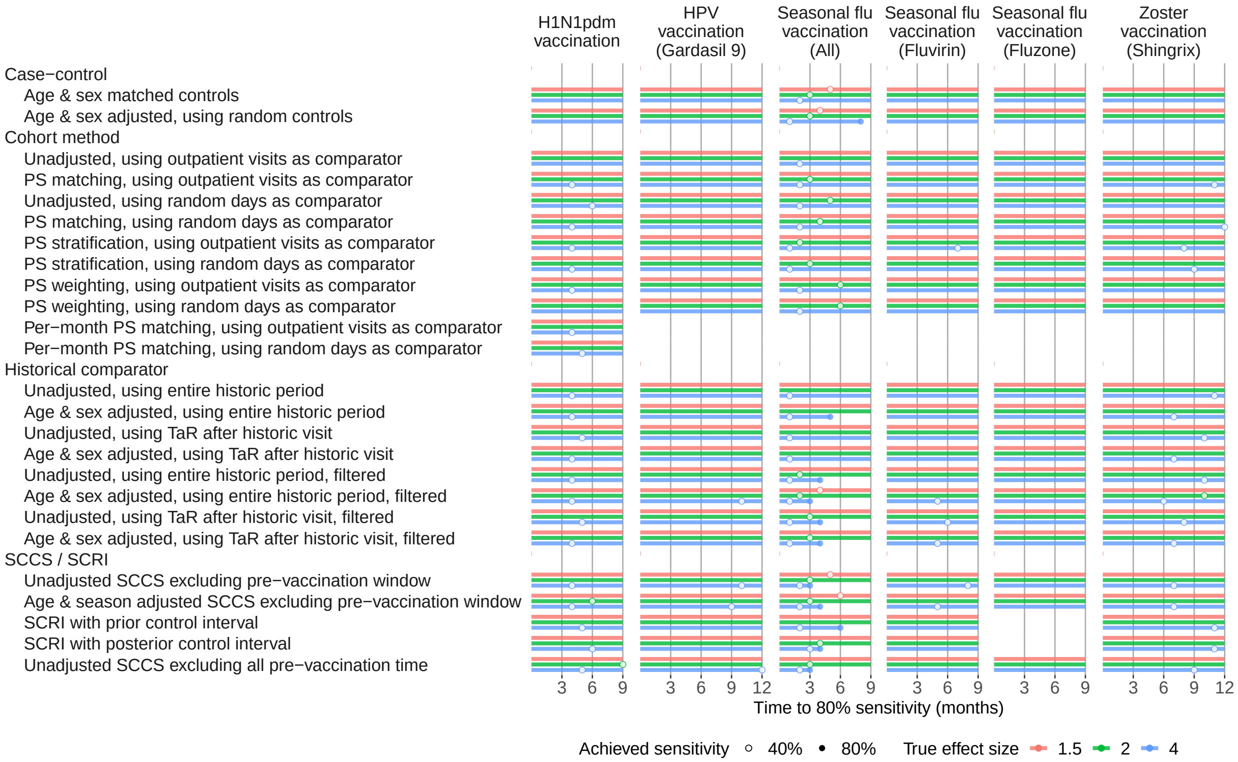

Figure 17: Time to 80% sensitivity in the CCAE database.

10 Type 1 and 2 error by true effect size based on p-value

For each method variation and vaccine group, at the end of the study period, the type 1 error and the type 2 error stratified by true effect size, based on whether $p < 0.05$.

10.1 Type 1 and 2 error for H1N1 vaccination

	Uncalibrated				Calibrated			
		Type 2 when true effect is				Type 2 when true effect is		
	Type 1	1.5	2	4	Type 1	1.5	2	4
Case−control								
Age & sex matched controls	17.2	77.4	66.7	60.2	1.1	93.5	89.2	78.5
Age & sex adjusted, using random controls	20.4	72.0	63.4	58.1	1.1	94.6	88.2	76.3
Cohort method								
Unadjusted, using outpatient visits as comparator	4.3	94.6	89.2	74.2	4.3	90.3	87.1	58.1
PS matching, using outpatient visits as comparator	0.0	97.8	91.4	69.9	1.1	90.3	83.9	61.3
Unadjusted, using random days as comparator	20.4	69.9	67.7	63.4	0.0	92.5	89.2	74.2
PS matching, using random days as comparator	0.0	100.0	100.0	100.0	0.0	100.0	100.0	100.0
PS stratification, using outpatient visits as comparator	0.0	94.6	86.0	64.5	2.2	88.2	77.4	51.6
PS stratification, using random days as comparator	38.7	49.5	46.2	39.8	0.0	86.0	76.3	53.8
PS weighting, using outpatient visits as comparator	3.2	91.4	84.9	66.7	3.2	91.4	83.9	62.4
PS weighting, using random days as comparator	0.0	100.0	100.0	100.0	0.0	100.0	100.0	100.0
Per−month PS matching, using outpatient visits as comparator	1.1	96.8	96.8	86.0	1.1	96.8	93.5	78.5
Per−month PS matching, using random days as comparator	1.1	96.8	93.5	88.2	0.0	97.8	95.7	89.2
Historical comparator								
Unadjusted, using entire historic period	46.2	43.0	35.5	31.2	4.3	83.9	71.0	45.2
Age & sex adjusted, using entire historic period	46.2	38.7	36.6	30.1	4.3	82.8	65.6	41.9
Unadjusted, using TaR after historic visit	20.4	64.5	55.9	35.5	5.4	86.0	79.6	49.5
Age & sex adjusted, using TaR after historic visit	24.7	57.0	49.5	35.5	5.4	82.8	73.1	46.2
Unadjusted, using entire historic period, filtered	44.1	46.2	39.8	35.5	3.2	82.8	73.1	48.4
Age & sex adjusted, using entire historic period, filtered	43.0	41.9	40.9	34.4	3.2	84.9	67.7	44.1
Unadjusted, using TaR after historic visit, filtered	19.4	66.7	58.1	39.8	4.3	87.1	80.6	52.7
Age & sex adjusted, using TaR after historic visit, filtered	22.6	59.1	51.6	39.8	4.3	83.9	75.3	48.4
SCCS / SCRI								
Unadjusted SCCS excluding pre−vaccination window	1.1	80.6	61.3	44.1	1.1	82.8	62.4	44.1
Age & season adjusted SCCS excluding pre−vaccination window	1.1	78.5	61.3	45.2	1.1	80.6	63.4	45.2
SCRI with prior control interval	1.1	90.3	78.5	60.2	1.1	90.3	82.8	60.2
SCRI with posterior control interval	2.2	89.2	79.6	54.8	1.1	92.5	81.7	58.1
Unadjusted SCCS excluding all pre−vaccination time	3.2	72.0	59.1	41.9	1.1	91.4	69.9	47.3

Figure 18: Type 1 and 2 error based on $p < 0.05$ at the end of the study period in the Optum EHR database for H1N1 vaccination.

	Uncalibrated				Calibrated			
		Type 2 when true effect is				Type 2 when true effect is		
	Type 1	1.5	2	4	Type 1	1.5	2	4
Case−control								
Age & sex matched controls	10.8	73.1	67.7	54.8	2.2	88.2	76.3	59.1
Age & sex adjusted, using random controls	7.5	74.2	64.5	49.5	1.1	84.9	74.2	55.9
Cohort method								
Unadjusted, using outpatient visits as comparator	3.2	95.7	95.7	92.5	4.3	94.6	93.5	83.9
PS matching, using outpatient visits as comparator	0.0	93.5	87.1	76.3	2.2	92.5	84.9	73.1
Unadjusted, using random days as comparator	4.3	93.5	93.5	79.6	3.2	93.5	92.5	77.4
PS matching, using random days as comparator	1.1	92.5	86.0	69.9	1.1	91.4	83.9	67.7
PS stratification, using outpatient visits as comparator	3.2	93.5	82.8	66.7	3.2	90.3	80.6	64.5
PS stratification, using random days as comparator	3.2	88.2	77.4	60.2	2.2	88.2	77.4	61.3
PS weighting, using outpatient visits as comparator	1.1	96.8	86.0	75.3	3.2	84.9	81.7	66.7
PS weighting, using random days as comparator	0.0	95.7	92.5	83.9	0.0	95.7	91.4	81.7
Per−month PS matching, using outpatient visits as comparator	0.0	95.7	87.1	74.2	1.1	91.4	83.9	72.0
Per−month PS matching, using random days as comparator	2.2	91.4	87.1	74.2	2.2	89.2	87.1	73.1
Historical comparator								
Unadjusted, using entire historic period	3.2	90.3	89.2	72.0	3.2	91.4	87.1	69.9
Age & sex adjusted, using entire historic period	9.7	73.1	62.4	49.5	4.3	83.9	75.3	54.8
Unadjusted, using TaR after historic visit	3.2	92.5	91.4	79.6	3.2	91.4	90.3	76.3
Age & sex adjusted, using TaR after historic visit	7.5	79.6	68.8	52.7	4.3	90.3	76.3	54.8
Unadjusted, using entire historic period, filtered	3.2	91.4	90.3	73.1	3.2	92.5	87.1	68.8
Age & sex adjusted, using entire historic period, filtered	8.6	74.2	63.4	50.5	3.2	83.9	76.3	54.8
Unadjusted, using TaR after historic visit, filtered	3.2	92.5	92.5	80.6	3.2	92.5	91.4	76.3
Age & sex adjusted, using TaR after historic visit, filtered	6.5	80.6	69.9	53.8	3.2	91.4	76.3	55.9
SCCS / SCRI								
Unadjusted SCCS excluding pre−vaccination window	2.2	86.0	72.0	57.0	1.1	89.2	77.4	57.0
Age & season adjusted SCCS excluding pre−vaccination window	2.2	86.0	71.0	55.9	2.2	83.9	71.0	53.8
SCRI with prior control interval	0.0	90.3	82.8	61.3	0.0	91.4	84.9	61.3
SCRI with posterior control interval	4.3	92.5	83.9	62.4	3.2	92.5	84.9	63.4
Unadjusted SCCS excluding all pre−vaccination time	2.2	83.9	72.0	58.1	1.1	89.2	76.3	59.1

Figure 19: Type 1 and 2 error based on $p < 0.05$ at the end of the study period in the MDCD database for H1N1 vaccination.

	Uncalibrated				Calibrated			
		Type 2 when true effect is				Type 2 when true effect is		
	Type 1	1.5	2	4	Type 1	1.5	2	4
Case−control								
Age & sex matched controls	8.6	84.9	82.8	75.3	2.2	94.6	89.2	81.7
Age & sex adjusted, using random controls	5.4	86.0	80.6	75.3	1.1	94.6	93.5	80.6
Cohort method								
Unadjusted, using outpatient visits as comparator	0.0	97.8	96.8	87.1	1.1	96.8	95.7	81.7
PS matching, using outpatient visits as comparator	0.0	97.8	96.8	93.5	0.0	97.8	96.8	91.4
Unadjusted, using random days as comparator	1.1	95.7	92.5	87.1	0.0	95.7	95.7	88.2
PS matching, using random days as comparator	0.0	96.8	95.7	91.4	0.0	98.9	96.8	92.5
PS stratification, using outpatient visits as comparator	0.0	95.7	89.2	79.6	0.0	95.7	89.2	79.6
PS stratification, using random days as comparator	5.4	92.5	87.1	78.5	0.0	94.6	89.2	80.6
PS weighting, using outpatient visits as comparator	6.5	91.4	90.3	83.9	1.1	96.8	94.6	93.5
PS weighting, using random days as comparator	0.0	98.9	97.8	93.5	0.0	100.0	97.8	95.7
Per−month PS matching, using outpatient visits as comparator	0.0	97.8	97.8	90.3	0.0	97.8	97.8	90.3
Per−month PS matching, using random days as comparator	0.0	97.8	96.8	95.7	0.0	96.8	96.8	93.5
Historical comparator								
Unadjusted, using entire historic period	10.8	75.3	68.8	63.4	3.2	93.5	89.2	72.0
Age & sex adjusted, using entire historic period	11.8	74.2	67.7	63.4	4.3	93.5	90.3	72.0
Unadjusted, using TaR after historic visit	6.5	81.7	73.1	64.5	4.3	94.6	90.3	71.0
Age & sex adjusted, using TaR after historic visit	6.5	79.6	72.0	64.5	4.3	94.6	91.4	69.9
Unadjusted, using entire historic period, filtered	9.7	76.3	69.9	64.5	3.2	94.6	89.2	73.1
Age & sex adjusted, using entire historic period, filtered	10.8	75.3	68.8	64.5	3.2	94.6	89.2	73.1
Unadjusted, using TaR after historic visit, filtered	5.4	82.8	74.2	65.6	3.2	94.6	91.4	71.0
Age & sex adjusted, using TaR after historic visit, filtered	5.4	80.6	73.1	65.6	3.2	95.7	92.5	71.0
SCCS / SCRI								
Unadjusted SCCS excluding pre−vaccination window	3.2	90.3	86.0	71.0	2.2	91.4	88.2	72.0
Age & season adjusted SCCS excluding pre−vaccination window	3.2	90.3	86.0	71.0	3.2	91.4	88.2	73.1
SCRI with prior control interval	2.2	94.6	92.5	82.8	0.0	97.8	94.6	88.2
SCRI with posterior control interval	0.0	97.8	95.7	82.8	0.0	96.8	95.7	82.8
Unadjusted SCCS excluding all pre−vaccination time	2.2	91.4	84.9	72.0	1.1	94.6	90.3	73.1

Figure 20: Type 1 and 2 error based on $p < 0.05$ at the end of the study period in the MDCR database for H1N1 vaccination.

	Uncalibrated				Calibrated			
		Type 2 when true effect is				Type 2 when true effect is		
	Type 1	1.5	2	4	Type 1	1.5	2	4
Case−control								
Age & sex matched controls	18.3	72.0	67.7	55.9	2.2	84.9	78.5	66.7
Age & sex adjusted, using random controls	19.4	68.8	64.5	54.8	1.1	89.2	77.4	59.1
Cohort method								
Unadjusted, using outpatient visits as comparator	3.2	95.7	90.3	79.6	4.3	89.2	83.9	61.3
PS matching, using outpatient visits as comparator	0.0	91.4	78.5	41.9	4.3	79.6	66.7	35.5
Unadjusted, using random days as comparator	12.9	68.8	55.9	33.3	3.2	86.0	74.2	36.6
PS matching, using random days as comparator	5.4	76.3	61.3	40.9	4.3	76.3	62.4	40.9
PS stratification, using outpatient visits as comparator	0.0	90.3	76.3	38.7	4.3	79.6	62.4	29.0
PS stratification, using random days as comparator	7.5	68.8	53.8	34.4	4.3	79.6	59.1	34.4
PS weighting, using outpatient visits as comparator	0.0	89.2	83.9	57.0	3.2	82.8	75.3	47.3
PS weighting, using random days as comparator	1.1	88.2	81.7	65.6	2.2	86.0	80.6	57.0
Per−month PS matching, using outpatient visits as comparator	1.1	88.2	76.3	41.9	3.2	80.6	63.4	34.4
Per−month PS matching, using random days as comparator	3.2	77.4	61.3	40.9	1.1	79.6	63.4	40.9
Historical comparator								
Unadjusted, using entire historic period	14.0	59.1	45.2	25.8	5.4	87.1	68.8	29.0
Age & sex adjusted, using entire historic period	31.2	44.1	36.6	24.7	2.2	82.8	55.9	32.3
Unadjusted, using TaR after historic visit	8.6	83.9	69.9	33.3	4.3	86.0	80.6	36.6
Age & sex adjusted, using TaR after historic visit	9.7	67.7	50.5	30.1	3.2	78.5	62.4	31.2
Unadjusted, using entire historic period, filtered	14.0	60.2	46.2	26.9	5.4	87.1	69.9	30.1
Age & sex adjusted, using entire historic period, filtered	30.1	45.2	37.6	25.8	2.2	82.8	57.0	33.3
Unadjusted, using TaR after historic visit, filtered	8.6	83.9	71.0	34.4	4.3	86.0	81.7	37.6
Age & sex adjusted, using TaR after historic visit, filtered	9.7	68.8	51.6	31.2	3.2	79.6	62.4	32.3
SCCS / SCRI								
Unadjusted SCCS excluding pre−vaccination window	7.5	64.5	47.3	26.9	2.2	79.6	52.7	28.0
Age & season adjusted SCCS excluding pre−vaccination window	8.6	63.4	44.1	29.0	3.2	71.0	51.6	29.0
SCRI with prior control interval	7.5	73.1	61.3	34.4	0.0	83.9	66.7	36.6
SCRI with posterior control interval	9.7	68.8	53.8	30.1	5.4	76.3	60.2	33.3
Unadjusted SCCS excluding all pre−vaccination time	11.8	64.5	47.3	28.0	3.2	80.6	58.1	29.0

Figure 21: Type 1 and 2 error based on $p < 0.05$ at the end of the study period in the CCAE database for H1N1 vaccination.

10.2 Type 1 and 2 error for Seasonal flu vaccination (Fluvirin)

	Uncalibrated				Calibrated			
		Type 2 when true effect is				Type 2 when true effect is		
	Type 1	1.5	2	4	Type 1	1.5	2	4
Case−control								
Age & sex matched controls	1.1	96.8	94.6	87.1	0.0	100.0	100.0	96.8
Age & sex adjusted, using random controls	2.2	94.6	94.6	89.2	0.0	100.0	100.0	97.8
Cohort method								
Unadjusted, using outpatient visits as comparator	0.0	97.8	96.8	89.2	2.2	95.7	92.5	78.5
PS matching, using outpatient visits as comparator	0.0	100.0	100.0	97.8	0.0	100.0	98.9	96.8
Unadjusted, using random days as comparator	0.0	95.7	94.6	83.9	0.0	100.0	97.8	90.3
PS matching, using random days as comparator								
PS stratification, using outpatient visits as comparator	2.2	97.8	96.8	92.5	2.2	95.7	93.5	89.2
PS stratification, using random days as comparator	6.5	88.2	79.6	67.7	1.1	94.6	93.5	79.6
PS weighting, using outpatient visits as comparator	0.0	100.0	96.8	94.6	0.0	96.8	96.8	93.5
PS weighting, using random days as comparator	0.0	100.0	100.0	100.0	0.0	100.0	100.0	100.0
Per−month PS matching, using outpatient visits as comparator								
Per−month PS matching, using random days as comparator								
Historical comparator								
Unadjusted, using entire historic period	18.3	65.6	63.4	55.9	4.3	93.5	92.5	77.4
Age & sex adjusted, using entire historic period	17.2	68.8	64.5	57.0	4.3	93.5	92.5	76.3
Unadjusted, using TaR after historic visit	9.7	84.9	76.3	64.5	3.2	93.5	91.4	79.6
Age & sex adjusted, using TaR after historic visit	8.6	86.0	77.4	64.5	4.3	93.5	91.4	76.3
Unadjusted, using entire historic period, filtered	17.2	66.7	64.5	57.0	5.4	94.6	92.5	75.3
Age & sex adjusted, using entire historic period, filtered	16.1	69.9	65.6	58.1	3.2	93.5	90.3	74.2
Unadjusted, using TaR after historic visit, filtered	8.6	86.0	77.4	65.6	4.3	93.5	90.3	76.3
Age & sex adjusted, using TaR after historic visit, filtered	7.5	87.1	78.5	65.6	3.2	93.5	91.4	75.3
SCCS / SCRI								
Unadjusted SCCS excluding pre−vaccination window	2.2	92.5	87.1	76.3	1.1	95.7	88.2	78.5
Age & season adjusted SCCS excluding pre−vaccination window	3.2	90.3	88.2	76.3	1.1	96.8	90.3	80.6
SCRI with prior control interval	0.0	98.9	95.7	88.2	0.0	100.0	97.8	89.2
SCRI with posterior control interval	0.0	97.8	96.8	82.8	0.0	100.0	97.8	83.9
Unadjusted SCCS excluding all pre−vaccination time	2.2	92.5	83.9	73.1	0.0	97.8	92.5	76.3

Figure 22: Type 1 and 2 error based on $p < 0.05$ at the end of the study period in the Optum EHR database for Seasonal flu vaccination (Fluvirin).

	Uncalibrated				Calibrated			
		Type 2 when true effect is				Type 2 when true effect is		
	Type 1	1.5	2	4	Type 1	1.5	2	4
Case−control								
Age & sex matched controls	4.3	92.5	90.3	83.9	1.1	94.6	92.5	87.1
Age & sex adjusted, using random controls	1.1	89.2	87.1	76.3	0.0	100.0	94.6	83.9
Cohort method								
Unadjusted, using outpatient visits as comparator	1.1	97.8	93.5	88.2	2.2	95.7	92.5	86.0
PS matching, using outpatient visits as comparator	0.0	100.0	100.0	100.0	0.0	100.0	100.0	100.0
Unadjusted, using random days as comparator	4.3	91.4	89.2	86.0	2.2	96.8	95.7	90.3
PS matching, using random days as comparator	0.0	100.0	100.0	100.0	0.0	100.0	100.0	100.0
PS stratification, using outpatient visits as comparator	0.0	94.6	92.5	81.7	2.2	92.5	91.4	79.6
PS stratification, using random days as comparator	7.5	84.9	81.7	73.1	0.0	95.7	89.2	80.6
PS weighting, using outpatient visits as comparator	0.0	100.0	98.9	94.6	0.0	100.0	98.9	93.5
PS weighting, using random days as comparator	0.0	100.0	100.0	100.0	0.0	100.0	100.0	100.0
Per−month PS matching, using outpatient visits as comparator								
Per−month PS matching, using random days as comparator								
Historical comparator								
Unadjusted, using entire historic period	11.8	80.6	77.4	67.7	1.1	98.9	98.9	91.4
Age & sex adjusted, using entire historic period	4.3	84.9	81.7	72.0	1.1	98.9	98.9	88.2
Unadjusted, using TaR after historic visit	5.4	86.0	79.6	72.0	1.1	98.9	97.8	90.3
Age & sex adjusted, using TaR after historic visit	2.2	92.5	89.2	78.5	1.1	98.9	96.8	89.2
Unadjusted, using entire historic period, filtered	10.8	81.7	78.5	68.8	0.0	93.5	89.2	79.6
Age & sex adjusted, using entire historic period, filtered	3.2	86.0	82.8	73.1	1.1	95.7	89.2	78.5
Unadjusted, using TaR after historic visit, filtered	4.3	87.1	80.6	73.1	2.2	95.7	91.4	79.6
Age & sex adjusted, using TaR after historic visit, filtered	1.1	93.5	90.3	79.6	1.1	94.6	90.3	79.6
SCCS / SCRI								
Unadjusted SCCS excluding pre−vaccination window	1.1	93.5	88.2	77.4	1.1	96.8	88.2	77.4
Age & season adjusted SCCS excluding pre−vaccination window	1.1	93.5	88.2	78.5	1.1	95.7	89.2	78.5
SCRI with prior control interval	0.0	97.8	95.7	88.2	1.1	95.7	93.5	87.1
SCRI with posterior control interval	0.0	95.7	91.4	83.9	0.0	96.8	92.5	86.0
Unadjusted SCCS excluding all pre−vaccination time	2.2	93.5	86.0	77.4	1.1	95.7	89.2	78.5

Figure 23: Type 1 and 2 error based on $p < 0.05$ at the end of the study period in the MDCD database for Seasonal flu vaccination (Fluvirin).

	Uncalibrated				Calibrated			
		Type 2 when true effect is				Type 2 when true effect is		
	Type 1	1.5	2	4	Type 1	1.5	2	4
Case−control								
Age & sex matched controls	0.0	100.0	100.0	97.8	0.0	100.0	100.0	100.0
Age & sex adjusted, using random controls	0.0	100.0	100.0	97.8	0.0	100.0	100.0	100.0
Cohort method								
Unadjusted, using outpatient visits as comparator	0.0	100.0	100.0	100.0	0.0	100.0	100.0	100.0
PS matching, using outpatient visits as comparator	0.0	100.0	100.0	100.0	0.0	100.0	100.0	100.0
Unadjusted, using random days as comparator	0.0	100.0	100.0	98.9	0.0	100.0	100.0	100.0
PS matching, using random days as comparator	0.0	100.0	100.0	100.0	0.0	100.0	100.0	100.0
PS stratification, using outpatient visits as comparator	1.1	98.9	98.9	97.8	0.0	98.9	98.9	98.9
PS stratification, using random days as comparator	0.0	100.0	98.9	97.8	0.0	100.0	100.0	100.0
PS weighting, using outpatient visits as comparator	1.1	98.9	98.9	98.9	1.1	98.9	98.9	98.9
PS weighting, using random days as comparator	0.0	100.0	100.0	100.0	0.0	100.0	100.0	100.0
Per−month PS matching, using outpatient visits as comparator								
Per−month PS matching, using random days as comparator								
Historical comparator								
Unadjusted, using entire historic period	2.2	95.7	94.6	93.5	0.0	98.9	97.8	96.8
Age & sex adjusted, using entire historic period	2.2	95.7	95.7	93.5	0.0	98.9	97.8	96.8
Unadjusted, using TaR after historic visit	1.1	95.7	94.6	93.5	0.0	98.9	98.9	96.8
Age & sex adjusted, using TaR after historic visit	1.1	95.7	94.6	93.5	0.0	100.0	98.9	96.8
Unadjusted, using entire historic period, filtered	2.2	95.7	94.6	93.5	0.0	98.9	97.8	96.8
Age & sex adjusted, using entire historic period, filtered	2.2	95.7	95.7	93.5	0.0	98.9	97.8	96.8
Unadjusted, using TaR after historic visit, filtered	1.1	95.7	94.6	93.5	0.0	98.9	98.9	96.8
Age & sex adjusted, using TaR after historic visit, filtered	1.1	95.7	94.6	93.5	0.0	100.0	98.9	96.8
SCCS / SCRI								
Unadjusted SCCS excluding pre−vaccination window	0.0	100.0	98.9	96.8	0.0	100.0	100.0	100.0
Age & season adjusted SCCS excluding pre−vaccination window	0.0	100.0	97.8	96.8	0.0	100.0	100.0	100.0
SCRI with prior control interval	0.0	100.0	100.0	98.9	0.0	100.0	100.0	100.0
SCRI with posterior control interval	0.0	100.0	100.0	98.9	0.0	100.0	100.0	100.0
Unadjusted SCCS excluding all pre−vaccination time	0.0	98.9	98.9	97.8	0.0	100.0	100.0	100.0

Figure 24: Type 1 and 2 error based on $p < 0.05$ at the end of the study period in the MDCR database for Seasonal flu vaccination (Fluvirin).

	Uncalibrated				Calibrated			
		Type 2 when true effect is				Type 2 when true effect is		
	Type 1	1.5	2	4	Type 1	1.5	2	4
Case−control								
Age & sex matched controls	3.2	87.1	83.9	73.1	0.0	93.5	87.1	78.5
Age & sex adjusted, using random controls	4.3	89.2	86.0	73.1	3.2	92.5	88.2	76.3
Cohort method								
Unadjusted, using outpatient visits as comparator	0.0	97.8	93.5	78.5	3.2	91.4	84.9	64.5
PS matching, using outpatient visits as comparator	1.1	93.5	87.1	69.9	2.2	92.5	84.9	68.8
Unadjusted, using random days as comparator	9.7	84.9	77.4	71.0	2.2	90.3	84.9	72.0
PS matching, using random days as comparator	2.2	83.9	79.6	66.7	1.1	87.1	80.6	66.7
PS stratification, using outpatient visits as comparator	2.2	91.4	80.6	64.5	3.2	84.9	76.3	60.2
PS stratification, using random days as comparator	3.2	80.6	72.0	58.1	2.2	82.8	74.2	59.1
PS weighting, using outpatient visits as comparator	3.2	91.4	82.8	71.0	3.2	92.5	82.8	66.7
PS weighting, using random days as comparator	0.0	94.6	87.1	81.7	0.0	98.9	90.3	83.9
Per−month PS matching, using outpatient visits as comparator								
Per−month PS matching, using random days as comparator								
Historical comparator								
Unadjusted, using entire historic period	17.2	74.2	63.4	49.5	2.2	97.8	94.6	74.2
Age & sex adjusted, using entire historic period	7.5	76.3	68.8	53.8	2.2	96.8	94.6	69.9
Unadjusted, using TaR after historic visit	8.6	84.9	74.2	53.8	2.2	95.7	93.5	74.2
Age & sex adjusted, using TaR after historic visit	6.5	84.9	76.3	55.9	3.2	95.7	93.5	71.0
Unadjusted, using entire historic period, filtered	15.1	76.3	65.6	51.6	2.2	82.8	76.3	59.1
Age & sex adjusted, using entire historic period, filtered	5.4	78.5	71.0	55.9	2.2	82.8	77.4	55.9
Unadjusted, using TaR after historic visit, filtered	6.5	87.1	76.3	55.9	2.2	88.2	76.3	58.1
Age & sex adjusted, using TaR after historic visit, filtered	4.3	87.1	78.5	58.1	3.2	90.3	80.6	58.1
SCCS / SCRI								
Unadjusted SCCS excluding pre−vaccination window	7.5	74.2	64.5	53.8	3.2	78.5	72.0	54.8
Age & season adjusted SCCS excluding pre−vaccination window	8.6	78.5	68.8	55.9	2.2	80.6	75.3	58.1
SCRI with prior control interval	4.3	84.9	78.5	67.7	1.1	92.5	84.9	71.0
SCRI with posterior control interval	6.5	83.9	78.5	66.7	1.1	87.1	81.7	71.0
Unadjusted SCCS excluding all pre−vaccination time	7.5	76.3	64.5	54.8	4.3	78.5	72.0	57.0

Figure 25: Type 1 and 2 error based on $p < 0.05$ at the end of the study period in the CCAE database for Seasonal flu vaccination (Fluvirin).

10.3 Type 1 and 2 error for Seasonal flu vaccination (Fluzone)

	Uncalibrated				Calibrated			
		Type 2 when true effect is				Type 2 when true effect is		
	Type 1	1.5	2	4	Type 1	1.5	2	4
Case−control								
Age & sex matched controls	20.4	61.3	57.0	43.0	3.2	88.2	81.7	57.0
Age & sex adjusted, using random controls	31.2	55.9	45.2	36.6	2.2	87.1	78.5	57.0
Cohort method								
Unadjusted, using outpatient visits as comparator	2.2	88.2	76.3	43.0	3.2	84.9	73.1	41.9
PS matching, using outpatient visits as comparator	1.1	94.6	82.8	49.5	4.3	82.8	66.7	44.1
Unadjusted, using random days as comparator	37.6	47.3	38.7	25.8	3.2	83.9	69.9	40.9
PS matching, using random days as comparator								
PS stratification, using outpatient visits as comparator	1.1	88.2	76.3	45.2	8.6	78.5	59.1	37.6
PS stratification, using random days as comparator	44.1	38.7	30.1	20.4	2.2	83.9	67.7	38.7
PS weighting, using outpatient visits as comparator	1.1	92.5	82.8	53.8	2.2	87.1	68.8	44.1
PS weighting, using random days as comparator	0.0	100.0	100.0	100.0	0.0	100.0	100.0	100.0
Per−month PS matching, using outpatient visits as comparator								
Per−month PS matching, using random days as comparator								
Historical comparator								
Unadjusted, using entire historic period	58.1	26.9	24.7	12.9	2.2	91.4	74.2	39.8
Age & sex adjusted, using entire historic period	59.1	31.2	24.7	15.1	2.2	91.4	78.5	37.6
Unadjusted, using TaR after historic visit	24.7	52.7	41.9	22.6	2.2	91.4	76.3	39.8
Age & sex adjusted, using TaR after historic visit	22.6	51.6	40.9	23.7	2.2	90.3	79.6	37.6
Unadjusted, using entire historic period, filtered	57.0	28.0	25.8	14.0	1.1	86.0	71.0	35.5
Age & sex adjusted, using entire historic period, filtered	58.1	32.3	25.8	16.1	2.2	88.2	71.0	34.4
Unadjusted, using TaR after historic visit, filtered	23.7	53.8	43.0	23.7	1.1	89.2	67.7	36.6
Age & sex adjusted, using TaR after historic visit, filtered	21.5	52.7	41.9	24.7	1.1	88.2	73.1	34.4
SCCS / SCRI								
Unadjusted SCCS excluding pre−vaccination window	20.4	49.5	35.5	19.4	3.2	82.8	59.1	26.9
Age & season adjusted SCCS excluding pre−vaccination window	26.9	49.5	39.8	23.7	3.2	83.9	65.6	35.5
SCRI with prior control interval	29.0	55.9	40.9	28.0	3.2	88.2	76.3	40.9
SCRI with posterior control interval	17.2	57.0	46.2	28.0	4.3	81.7	61.3	36.6
Unadjusted SCCS excluding all pre−vaccination time	30.1	47.3	33.3	20.4	4.3	78.5	60.2	25.8

Figure 26: Type 1 and 2 error based on $p < 0.05$ at the end of the study period in the Optum EHR database for Seasonal flu vaccination (Fluzone).

	Uncalibrated				Calibrated			
		Type 2 when true effect is				Type 2 when true effect is		
	Type 1	1.5	2	4	Type 1	1.5	2	4
Case−control								
Age & sex matched controls	0.0	97.8	95.7	93.5	0.0	100.0	100.0	96.8
Age & sex adjusted, using random controls	1.1	96.8	95.7	93.5	0.0	100.0	98.9	96.8
Cohort method								
Unadjusted, using outpatient visits as comparator	0.0	98.9	98.9	97.8	0.0	98.9	98.9	97.8
PS matching, using outpatient visits as comparator	0.0	100.0	100.0	98.9	0.0	100.0	100.0	98.9
Unadjusted, using random days as comparator	1.1	97.8	97.8	97.8	0.0	98.9	98.9	97.8
PS matching, using random days as comparator								
PS stratification, using outpatient visits as comparator	1.1	94.6	94.6	92.5	1.1	98.9	95.7	93.5
PS stratification, using random days as comparator	2.2	94.6	90.3	86.0	0.0	98.9	97.8	92.5
PS weighting, using outpatient visits as comparator	0.0	100.0	98.9	97.8	0.0	100.0	98.9	98.9
PS weighting, using random days as comparator	0.0	100.0	100.0	100.0	0.0	100.0	100.0	100.0
Per−month PS matching, using outpatient visits as comparator								
Per−month PS matching, using random days as comparator								
Historical comparator								
Unadjusted, using entire historic period	11.8	81.7	80.6	76.3	3.2	96.8	95.7	92.5
Age & sex adjusted, using entire historic period	7.5	84.9	84.9	78.5	2.2	96.8	93.5	88.2
Unadjusted, using TaR after historic visit	9.7	83.9	82.8	77.4	2.2	96.8	94.6	92.5
Age & sex adjusted, using TaR after historic visit	4.3	89.2	88.2	83.9	3.2	96.8	92.5	87.1
Unadjusted, using entire historic period, filtered	10.8	82.8	81.7	77.4	2.2	96.8	95.7	92.5
Age & sex adjusted, using entire historic period, filtered	6.5	86.0	86.0	79.6	2.2	96.8	93.5	88.2
Unadjusted, using TaR after historic visit, filtered	8.6	84.9	83.9	78.5	2.2	95.7	95.7	92.5
Age & sex adjusted, using TaR after historic visit, filtered	3.2	90.3	89.2	84.9	2.2	96.8	93.5	88.2
SCCS / SCRI								
Unadjusted SCCS excluding pre−vaccination window	3.2	95.7	92.5	87.1	1.1	96.8	95.7	91.4
Age & season adjusted SCCS excluding pre−vaccination window	3.2	96.8	92.5	87.1	1.1	96.8	96.8	90.3
SCRI with prior control interval	1.1	98.9	98.9	96.8	0.0	98.9	98.9	96.8
SCRI with posterior control interval	0.0	98.9	98.9	96.8	0.0	98.9	98.9	96.8
Unadjusted SCCS excluding all pre−vaccination time	4.3	95.7	93.5	88.2	1.1	95.7	95.7	91.4

Figure 27: Type 1 and 2 error based on $p < 0.05$ at the end of the study period in the MDCD database for Seasonal flu vaccination (Fluzone).

	Uncalibrated				Calibrated			
		Type 2 when true effect is				Type 2 when true effect is		
	Type 1	1.5	2	4	Type 1	1.5	2	4
Case−control								
Age & sex matched controls	0.0	96.8	91.4	77.4	1.1	91.4	88.2	73.1
Age & sex adjusted, using random controls	1.1	90.3	86.0	68.8	1.1	91.4	86.0	68.8
Cohort method								
Unadjusted, using outpatient visits as comparator	0.0	98.9	94.6	86.0	2.2	91.4	89.2	80.6
PS matching, using outpatient visits as comparator	0.0	98.9	95.7	84.9	0.0	95.7	89.2	83.9
Unadjusted, using random days as comparator	1.1	93.5	89.2	81.7	1.1	92.5	88.2	79.6
PS matching, using random days as comparator	1.1	95.7	93.5	84.9	1.1	95.7	93.5	83.9
PS stratification, using outpatient visits as comparator	0.0	100.0	93.5	79.6	0.0	91.4	84.9	76.3
PS stratification, using random days as comparator	1.1	95.7	88.2	72.0	1.1	95.7	87.1	69.9
PS weighting, using outpatient visits as comparator	0.0	98.9	98.9	82.8	1.1	90.3	83.9	76.3
PS weighting, using random days as comparator	2.2	97.8	94.6	90.3	2.2	97.8	96.8	91.4
Per−month PS matching, using outpatient visits as comparator								
Per−month PS matching, using random days as comparator								
Historical comparator								
Unadjusted, using entire historic period	6.5	83.9	78.5	61.3	4.3	93.5	90.3	75.3
Age & sex adjusted, using entire historic period	7.5	82.8	77.4	60.2	4.3	93.5	88.2	75.3
Unadjusted, using TaR after historic visit	7.5	84.9	81.7	67.7	3.2	93.5	88.2	76.3
Age & sex adjusted, using TaR after historic visit	7.5	88.2	81.7	68.8	3.2	92.5	87.1	75.3
Unadjusted, using entire historic period, filtered	4.3	86.0	80.6	63.4	5.4	87.1	80.6	63.4
Age & sex adjusted, using entire historic period, filtered	5.4	84.9	79.6	62.4	6.5	84.9	79.6	62.4
Unadjusted, using TaR after historic visit, filtered	5.4	87.1	83.9	69.9	4.3	89.2	84.9	68.8
Age & sex adjusted, using TaR after historic visit, filtered	5.4	90.3	83.9	71.0	4.3	89.2	82.8	67.7
SCCS / SCRI								
Unadjusted SCCS excluding pre−vaccination window	2.2	90.3	84.9	71.0	2.2	90.3	82.8	68.8
Age & season adjusted SCCS excluding pre−vaccination window	1.1	89.2	82.8	68.8	1.1	89.2	82.8	68.8
SCRI with prior control interval	1.1	93.5	90.3	86.0	1.1	93.5	91.4	86.0
SCRI with posterior control interval	2.2	93.5	90.3	81.7	2.2	93.5	90.3	81.7
Unadjusted SCCS excluding all pre−vaccination time	1.1	91.4	83.9	71.0	3.2	90.3	82.8	71.0

Figure 28: Type 1 and 2 error based on $p < 0.05$ at the end of the study period in the MDCR database for Seasonal flu vaccination (Fluzone).

	Uncalibrated				Calibrated			
		Type 2 when true effect is				Type 2 when true effect is		
	Type 1	1.5	2	4	Type 1	1.5	2	4
Case−control								
Age & sex matched controls	0.0	100.0	100.0	100.0	0.0	100.0	100.0	100.0
Age & sex adjusted, using random controls	0.0	100.0	100.0	100.0	0.0	100.0	100.0	100.0
Cohort method								
Unadjusted, using outpatient visits as comparator	0.0	100.0	100.0	100.0	0.0	100.0	100.0	100.0
PS matching, using outpatient visits as comparator	0.0	100.0	100.0	100.0	0.0	100.0	100.0	100.0
Unadjusted, using random days as comparator	0.0	100.0	100.0	100.0	0.0	100.0	100.0	100.0
PS matching, using random days as comparator	0.0	100.0	100.0	100.0	0.0	100.0	100.0	100.0
PS stratification, using outpatient visits as comparator	1.1	98.9	98.9	95.7	0.0	100.0	98.9	98.9
PS stratification, using random days as comparator	0.0	100.0	100.0	100.0	0.0	100.0	100.0	100.0
PS weighting, using outpatient visits as comparator	0.0	100.0	100.0	98.9	0.0	100.0	100.0	100.0
PS weighting, using random days as comparator	0.0	100.0	100.0	100.0	0.0	100.0	100.0	100.0
Per−month PS matching, using outpatient visits as comparator								
Per−month PS matching, using random days as comparator								
Historical comparator								
Unadjusted, using entire historic period	8.6	91.4	91.4	88.2	1.1	98.9	98.9	97.8
Age & sex adjusted, using entire historic period	6.5	91.4	91.4	89.2	1.1	98.9	98.9	96.8
Unadjusted, using TaR after historic visit	7.5	91.4	91.4	89.2	1.1	98.9	98.9	97.8
Age & sex adjusted, using TaR after historic visit	5.4	91.4	91.4	90.3	1.1	98.9	98.9	96.8
Unadjusted, using entire historic period, filtered	7.5	92.5	92.5	89.2	1.1	98.9	98.9	97.8
Age & sex adjusted, using entire historic period, filtered	5.4	92.5	92.5	90.3	1.1	98.9	98.9	97.8
Unadjusted, using TaR after historic visit, filtered	6.5	92.5	92.5	90.3	1.1	98.9	98.9	97.8
Age & sex adjusted, using TaR after historic visit, filtered	4.3	92.5	92.5	91.4	1.1	98.9	98.9	97.8
SCCS / SCRI								
Unadjusted SCCS excluding pre−vaccination window	0.0	100.0	100.0	97.8	0.0	100.0	100.0	100.0
Age & season adjusted SCCS excluding pre−vaccination window	0.0	100.0	100.0	97.8	0.0	100.0	100.0	100.0
SCRI with prior control interval								
SCRI with posterior control interval								
Unadjusted SCCS excluding all pre−vaccination time	0.0	100.0	100.0	98.9	0.0	100.0	100.0	100.0

Figure 29: Type 1 and 2 error based on $p < 0.05$ at the end of the study period in the CCAE database for Seasonal flu vaccination (Fluzone).

10.4 Type 1 and 2 error for Seasonal flu vaccination (All)

	Uncalibrated				Calibrated			
		Type 2 when true effect is				Type 2 when true effect is		
	Type 1	1.5	2	4	Type 1	1.5	2	4
Case−control								
Age & sex matched controls	65.6	20.4	16.1	9.7	5.4	68.8	46.2	19.4
Age & sex adjusted, using random controls	73.1	14.0	11.8	9.7	6.5	63.4	33.3	11.8
Cohort method								
Unadjusted, using outpatient visits as comparator	2.2	83.9	57.0	18.3	3.2	75.3	52.7	11.8
PS matching, using outpatient visits as comparator	0.0	77.4	50.5	17.2	5.4	59.1	35.5	10.8
Unadjusted, using random days as comparator	73.1	12.9	11.8	4.3	4.3	68.8	43.0	12.9
PS matching, using random days as comparator								
PS stratification, using outpatient visits as comparator	1.1	77.4	50.5	11.8	4.3	65.6	34.4	8.6
PS stratification, using random days as comparator	83.9	5.4	4.3	3.2	2.2	66.7	38.7	5.4
PS weighting, using outpatient visits as comparator	0.0	88.2	68.8	23.7	3.2	69.9	41.9	14.0
PS weighting, using random days as comparator	0.0	100.0	100.0	100.0	0.0	100.0	100.0	100.0
Per−month PS matching, using outpatient visits as comparator								
Per−month PS matching, using random days as comparator								
Historical comparator								
Unadjusted, using entire historic period	90.3	4.3	2.2	1.1	1.1	80.6	41.9	7.5
Age & sex adjusted, using entire historic period	91.4	4.3	3.2	1.1	1.1	82.8	33.3	4.3
Unadjusted, using TaR after historic visit	53.8	20.4	15.1	2.2	3.2	82.8	45.2	7.5
Age & sex adjusted, using TaR after historic visit	61.3	18.3	9.7	2.2	3.2	82.8	35.5	4.3
Unadjusted, using entire historic period, filtered	89.2	5.4	3.2	2.2	2.2	53.8	32.3	5.4
Age & sex adjusted, using entire historic period, filtered	90.3	5.4	4.3	2.2	3.2	39.8	22.6	5.4
Unadjusted, using TaR after historic visit, filtered	52.7	21.5	16.1	3.2	2.2	62.4	34.4	6.5
Age & sex adjusted, using TaR after historic visit, filtered	60.2	19.4	10.8	3.2	4.3	44.1	23.7	4.3
SCCS / SCRI								
Unadjusted SCCS excluding pre−vaccination window	36.6	17.2	11.8	2.2	4.3	52.7	17.2	4.3
Age & season adjusted SCCS excluding pre−vaccination window	45.2	24.7	17.2	9.7	2.2	61.3	35.5	12.9
SCRI with prior control interval	58.1	18.3	11.8	5.4	3.2	71.0	28.0	9.7
SCRI with posterior control interval	47.3	17.2	8.6	4.3	4.3	61.3	24.7	6.5
Unadjusted SCCS excluding all pre−vaccination time	53.8	12.9	6.5	3.2	3.2	59.1	17.2	4.3

Figure 30: Type 1 and 2 error based on $p < 0.05$ at the end of the study period in the Optum EHR database for Seasonal flu vaccination (All).

	Uncalibrated				Calibrated			
		Type 2 when true effect is				Type 2 when true effect is		
	Type 1	1.5	2	4	Type 1	1.5	2	4
Case−control								
Age & sex matched controls	46.2	37.6	34.4	26.9	5.4	73.1	50.5	32.3
Age & sex adjusted, using random controls	54.8	28.0	22.6	15.1	4.3	68.8	44.1	22.6
Cohort method								
Unadjusted, using outpatient visits as comparator	6.5	91.4	83.9	45.2	7.5	89.2	80.6	31.2
PS matching, using outpatient visits as comparator	0.0	77.4	57.0	25.8	2.2	72.0	49.5	23.7
Unadjusted, using random days as comparator	45.2	29.0	23.7	19.4	7.5	79.6	52.7	22.6
PS matching, using random days as comparator	3.2	73.1	55.9	32.3	3.2	71.0	51.6	30.1
PS stratification, using outpatient visits as comparator	1.1	75.3	52.7	22.6	5.4	60.2	39.8	19.4
PS stratification, using random days as comparator	7.5	61.3	50.5	22.6	4.3	69.9	51.6	23.7
PS weighting, using outpatient visits as comparator	1.1	74.2	53.8	21.5	1.1	73.1	48.4	20.4
PS weighting, using random days as comparator	1.1	81.7	64.5	34.4	4.3	75.3	53.8	31.2
Per−month PS matching, using outpatient visits as comparator								
Per−month PS matching, using random days as comparator								
Historical comparator								
Unadjusted, using entire historic period	44.1	22.6	19.4	15.1	2.2	91.4	80.6	19.4
Age & sex adjusted, using entire historic period	59.1	19.4	18.3	12.9	2.2	93.5	80.6	19.4
Unadjusted, using TaR after historic visit	17.2	53.8	34.4	18.3	3.2	91.4	78.5	28.0
Age & sex adjusted, using TaR after historic visit	17.2	44.1	29.0	18.3	2.2	91.4	80.6	22.6
Unadjusted, using entire historic period, filtered	43.0	23.7	20.4	16.1	5.4	75.3	45.2	18.3
Age & sex adjusted, using entire historic period, filtered	58.1	20.4	19.4	14.0	4.3	78.5	41.9	19.4
Unadjusted, using TaR after historic visit, filtered	16.1	54.8	35.5	19.4	7.5	78.5	54.8	20.4
Age & sex adjusted, using TaR after historic visit, filtered	16.1	45.2	30.1	19.4	7.5	69.9	45.2	19.4
SCCS / SCRI								
Unadjusted SCCS excluding pre−vaccination window	9.7	45.2	24.7	19.4	4.3	52.7	25.8	19.4
Age & season adjusted SCCS excluding pre−vaccination window	6.5	46.2	26.9	22.6	2.2	54.8	29.0	22.6
SCRI with prior control interval	15.1	51.6	37.6	20.4	1.1	76.3	52.7	22.6
SCRI with posterior control interval	15.1	49.5	33.3	19.4	3.2	66.7	41.9	19.4
Unadjusted SCCS excluding all pre−vaccination time	16.1	40.9	24.7	19.4	2.2	61.3	33.3	19.4

Figure 31: Type 1 and 2 error based on $p < 0.05$ at the end of the study period in the MDCD database for Seasonal flu vaccination (All).

	Uncalibrated				Calibrated			
		Type 2 when true effect is				Type 2 when true effect is		
	Type 1	1.5	2	4	Type 1	1.5	2	4
Case−control								
Age & sex matched controls	5.4	64.5	52.7	37.6	2.2	69.9	52.7	39.8
Age & sex adjusted, using random controls	17.2	40.9	29.0	22.6	1.1	67.7	37.6	23.7
Cohort method								
Unadjusted, using outpatient visits as comparator	0.0	95.7	84.9	50.5	2.2	81.7	62.4	30.1
PS matching, using outpatient visits as comparator	1.1	83.9	68.8	44.1	3.2	72.0	63.4	36.6
Unadjusted, using random days as comparator	14.0	65.6	45.2	29.0	4.3	76.3	60.2	32.3
PS matching, using random days as comparator	2.2	80.6	69.9	44.1	3.2	78.5	65.6	43.0
PS stratification, using outpatient visits as comparator	1.1	84.9	69.9	39.8	6.5	76.3	59.1	35.5
PS stratification, using random days as comparator	5.4	76.3	58.1	37.6	4.3	76.3	59.1	37.6
PS weighting, using outpatient visits as comparator	0.0	94.6	80.6	48.4	3.2	78.5	64.5	39.8
PS weighting, using random days as comparator	1.1	89.2	74.2	46.2	2.2	82.8	69.9	44.1
Per−month PS matching, using outpatient visits as comparator								
Per−month PS matching, using random days as comparator								
Historical comparator								
Unadjusted, using entire historic period	25.8	39.8	28.0	19.4	2.2	93.5	68.8	24.7
Age & sex adjusted, using entire historic period	24.7	39.8	28.0	19.4	2.2	94.6	67.7	24.7
Unadjusted, using TaR after historic visit	10.8	60.2	34.4	21.5	2.2	92.5	66.7	26.9
Age & sex adjusted, using TaR after historic visit	10.8	58.1	34.4	22.6	2.2	92.5	66.7	28.0
Unadjusted, using entire historic period, filtered	23.7	41.9	30.1	21.5	2.2	55.9	37.6	24.7
Age & sex adjusted, using entire historic period, filtered	22.6	41.9	30.1	21.5	1.1	54.8	37.6	24.7
Unadjusted, using TaR after historic visit, filtered	8.6	62.4	36.6	23.7	2.2	71.0	44.1	24.7
Age & sex adjusted, using TaR after historic visit, filtered	8.6	60.2	36.6	24.7	3.2	69.9	43.0	24.7
SCCS / SCRI								
Unadjusted SCCS excluding pre−vaccination window	8.6	55.9	39.8	23.7	2.2	63.4	40.9	23.7
Age & season adjusted SCCS excluding pre−vaccination window	6.5	58.1	43.0	32.3	3.2	65.6	44.1	32.3
SCRI with prior control interval	16.1	57.0	47.3	31.2	1.1	83.9	63.4	38.7
SCRI with posterior control interval	8.6	66.7	49.5	29.0	1.1	75.3	55.9	30.1
Unadjusted SCCS excluding all pre−vaccination time	15.1	55.9	39.8	21.5	3.2	73.1	52.7	25.8

Figure 32: Type 1 and 2 error based on $p < 0.05$ at the end of the study period in the MDCR database for Seasonal flu vaccination (All).

	Uncalibrated				Calibrated			
		Type 2 when true effect is				Type 2 when true effect is		
	Type 1	1.5	2	4	Type 1	1.5	2	4
Case−control								
Age & sex matched controls	32.3	37.6	30.1	19.4	3.2	63.4	38.7	22.6
Age & sex adjusted, using random controls	36.6	37.6	28.0	17.2	2.2	55.9	36.6	18.3
Cohort method								
Unadjusted, using outpatient visits as comparator	3.2	93.5	87.1	58.1	5.4	87.1	76.3	34.4
PS matching, using outpatient visits as comparator	3.2	74.2	55.9	25.8	4.3	67.7	45.2	24.7
Unadjusted, using random days as comparator	34.4	37.6	24.7	18.3	2.2	74.2	52.7	18.3
PS matching, using random days as comparator	7.5	64.5	43.0	23.7	2.2	66.7	45.2	24.7
PS stratification, using outpatient visits as comparator	1.1	77.4	60.2	28.0	3.2	67.7	43.0	20.4
PS stratification, using random days as comparator	10.8	53.8	36.6	20.4	3.2	64.5	43.0	20.4
PS weighting, using outpatient visits as comparator	0.0	90.3	74.2	37.6	4.3	75.3	55.9	28.0
PS weighting, using random days as comparator	2.2	68.8	54.8	32.3	2.2	68.8	52.7	32.3
Per−month PS matching, using outpatient visits as comparator								
Per−month PS matching, using random days as comparator								
Historical comparator								
Unadjusted, using entire historic period	47.3	23.7	18.3	12.9	2.2	95.7	74.2	18.3
Age & sex adjusted, using entire historic period	44.1	22.6	18.3	12.9	2.2	95.7	71.0	17.2
Unadjusted, using TaR after historic visit	17.2	52.7	30.1	17.2	2.2	89.2	80.6	21.5
Age & sex adjusted, using TaR after historic visit	18.3	51.6	24.7	16.1	2.2	93.5	77.4	20.4
Unadjusted, using entire historic period, filtered	45.2	25.8	20.4	15.1	2.2	54.8	30.1	18.3
Age & sex adjusted, using entire historic period, filtered	41.9	24.7	20.4	15.1	4.3	39.8	24.7	17.2
Unadjusted, using TaR after historic visit, filtered	15.1	54.8	32.3	19.4	6.5	77.4	50.5	19.4
Age & sex adjusted, using TaR after historic visit, filtered	16.1	53.8	26.9	18.3	3.2	67.7	38.7	19.4
SCCS / SCRI								
Unadjusted SCCS excluding pre−vaccination window	19.4	28.0	18.3	14.0	3.2	41.9	18.3	14.0
Age & season adjusted SCCS excluding pre−vaccination window	16.1	35.5	24.7	21.5	3.2	47.3	24.7	21.5
SCRI with prior control interval	31.2	36.6	24.7	16.1	1.1	74.2	41.9	20.4
SCRI with posterior control interval	21.5	31.2	21.5	15.1	3.2	64.5	30.1	15.1
Unadjusted SCCS excluding all pre−vaccination time	30.1	28.0	18.3	14.0	3.2	52.7	24.7	14.0

Figure 33: Type 1 and 2 error based on $p < 0.05$ at the end of the study period in the CCAE database for Seasonal flu vaccination (All).

10.5 Type 1 and 2 error for Zoster vaccination (Shingrix)

	Uncalibrated Type 1	Uncalibrated Type 2 when true effect is 1.5	Uncalibrated Type 2 when true effect is 2	Uncalibrated Type 2 when true effect is 4	Calibrated Type 1	Calibrated Type 2 when true effect is 1.5	Calibrated Type 2 when true effect is 2	Calibrated Type 2 when true effect is 4
**Case−control**								
Age & sex matched controls	24.7	66.7	63.4	48.4	4.3	86.0	75.3	63.4
Age & sex adjusted, using random controls	20.4	67.7	62.4	46.2	3.2	83.9	79.6	62.4
**Cohort method**								
Unadjusted, using outpatient visits as comparator	1.1	87.1	76.3	51.6	1.1	86.0	75.3	45.2
PS matching, using outpatient visits as comparator	0.0	95.7	81.7	48.4	3.2	76.3	61.3	33.3
Unadjusted, using random days as comparator	35.5	51.6	45.2	32.3	3.2	89.2	82.8	52.7
PS matching, using random days as comparator								
PS stratification, using outpatient visits as comparator	0.0	93.5	78.5	43.0	5.4	76.3	62.4	32.3
PS stratification, using random days as comparator								
PS weighting, using outpatient visits as comparator	0.0	96.8	90.3	68.8	1.1	89.2	80.6	59.1
PS weighting, using random days as comparator								
Per−month PS matching, using outpatient visits as comparator								
Per−month PS matching, using random days as comparator								
**Historical comparator**								
Unadjusted, using entire historic period	55.9	30.1	28.0	20.4	3.2	89.2	83.9	48.4
Age & sex adjusted, using entire historic period	40.9	35.5	26.9	17.2	3.2	83.9	69.9	36.6
Unadjusted, using TaR after historic visit	29.0	52.7	41.9	29.0	3.2	90.3	79.6	43.0
Age & sex adjusted, using TaR after historic visit	20.4	63.4	55.9	28.0	5.4	82.8	72.0	39.8
Unadjusted, using entire historic period, filtered	54.8	31.2	29.0	21.5	3.2	89.2	81.7	48.4
Age & sex adjusted, using entire historic period, filtered	39.8	36.6	28.0	18.3	2.2	81.7	69.9	34.4
Unadjusted, using TaR after historic visit, filtered	28.0	53.8	43.0	30.1	2.2	89.2	78.5	43.0
Age & sex adjusted, using TaR after historic visit, filtered	19.4	64.5	57.0	29.0	5.4	81.7	69.9	37.6
**SCCS / SCRI**								
Unadjusted SCCS excluding pre−vaccination window	11.8	61.3	48.4	22.6	3.2	78.5	57.0	24.7
Age & season adjusted SCCS excluding pre−vaccination window	8.6	67.7	50.5	22.6	5.4	79.6	55.9	23.7
SCRI with prior control interval	9.7	66.7	51.6	33.3	3.2	83.9	63.4	35.5
SCRI with posterior control interval	11.8	69.9	59.1	36.6	3.2	80.6	68.8	40.9
Unadjusted SCCS excluding all pre−vaccination time	15.1	55.9	43.0	26.9	2.2	77.4	57.0	30.1

Figure 34: Type 1 and 2 error based on $p < 0.05$ at the end of the study period in the Optum EHR database for Zoster vaccination (Shingrix).

	Uncalibrated				Calibrated			
		Type 2 when true effect is				Type 2 when true effect is		
	Type 1	1.5	2	4	Type 1	1.5	2	4
Case−control								
Age & sex matched controls	3.2	90.3	87.1	77.4	1.1	96.8	93.5	86.0
Age & sex adjusted, using random controls	4.3	91.4	87.1	72.0	1.1	95.7	94.6	84.9
Cohort method								
Unadjusted, using outpatient visits as comparator	1.1	93.5	88.2	79.6	0.0	94.6	88.2	79.6
PS matching, using outpatient visits as comparator	0.0	98.9	97.8	84.9	1.1	98.9	96.8	83.9
Unadjusted, using random days as comparator	7.5	89.2	87.1	82.8	2.2	96.8	94.6	89.2
PS matching, using random days as comparator	0.0	97.8	95.7	90.3	0.0	97.8	94.6	88.2
PS stratification, using outpatient visits as comparator	2.2	93.5	90.3	79.6	5.4	92.5	88.2	76.3
PS stratification, using random days as comparator	1.1	93.5	90.3	74.2	0.0	93.5	90.3	77.4
PS weighting, using outpatient visits as comparator	0.0	100.0	98.9	90.3	0.0	100.0	98.9	90.3
PS weighting, using random days as comparator	0.0	100.0	95.7	89.2	0.0	100.0	95.7	89.2
Per−month PS matching, using outpatient visits as comparator								
Per−month PS matching, using random days as comparator								
Historical comparator								
Unadjusted, using entire historic period	25.8	66.7	59.1	50.5	1.1	96.8	90.3	79.6
Age & sex adjusted, using entire historic period	12.9	71.0	64.5	57.0	2.2	95.7	91.4	67.7
Unadjusted, using TaR after historic visit	21.5	72.0	69.9	51.6	1.1	94.6	88.2	75.3
Age & sex adjusted, using TaR after historic visit	2.2	90.3	80.6	65.6	1.1	95.7	88.2	66.7
Unadjusted, using entire historic period, filtered	24.7	67.7	60.2	51.6	1.1	91.4	89.2	74.2
Age & sex adjusted, using entire historic period, filtered	11.8	72.0	65.6	58.1	1.1	88.2	81.7	64.5
Unadjusted, using TaR after historic visit, filtered	20.4	73.1	71.0	52.7	0.0	91.4	88.2	73.1
Age & sex adjusted, using TaR after historic visit, filtered	1.1	91.4	81.7	66.7	2.2	91.4	81.7	66.7
SCCS / SCRI								
Unadjusted SCCS excluding pre−vaccination window	1.1	89.2	76.3	66.7	1.1	92.5	82.8	66.7
Age & season adjusted SCCS excluding pre−vaccination window	2.2	90.3	78.5	66.7	1.1	92.5	80.6	66.7
SCRI with prior control interval	0.0	98.9	93.5	78.5	0.0	96.8	89.2	77.4
SCRI with posterior control interval	1.1	95.7	89.2	77.4	1.1	95.7	89.2	77.4
Unadjusted SCCS excluding all pre−vaccination time	1.1	90.3	83.9	65.6	0.0	92.5	86.0	68.8

Figure 35: Type 1 and 2 error based on $p < 0.05$ at the end of the study period in the MDCD database for Zoster vaccination (Shingrix).

	Uncalibrated				Calibrated			
		Type 2 when true effect is				Type 2 when true effect is		
	Type 1	1.5	2	4	Type 1	1.5	2	4
**Case−control**								
Age & sex matched controls	7.5	82.8	78.5	67.7	1.1	92.5	80.6	71.0
Age & sex adjusted, using random controls	11.8	76.3	72.0	58.1	3.2	91.4	79.6	63.4
**Cohort method**								
Unadjusted, using outpatient visits as comparator	2.2	95.7	89.2	77.4	2.2	92.5	89.2	72.0
PS matching, using outpatient visits as comparator	1.1	93.5	84.9	69.9	2.2	86.0	82.8	65.6
Unadjusted, using random days as comparator	4.3	80.6	76.3	63.4	1.1	87.1	79.6	64.5
PS matching, using random days as comparator	1.1	88.2	82.8	63.4	2.2	86.0	80.6	63.4
PS stratification, using outpatient visits as comparator	2.2	89.2	79.6	64.5	3.2	90.3	77.4	61.3
PS stratification, using random days as comparator	4.3	81.7	75.3	54.8	4.3	82.8	75.3	54.8
PS weighting, using outpatient visits as comparator	2.2	95.7	84.9	71.0	2.2	95.7	81.7	67.7
PS weighting, using random days as comparator	1.1	95.7	87.1	79.6	1.1	96.8	87.1	79.6
Per−month PS matching, using outpatient visits as comparator								
Per−month PS matching, using random days as comparator								
**Historical comparator**								
Unadjusted, using entire historic period	12.9	76.3	67.7	55.9	3.2	91.4	88.2	67.7
Age & sex adjusted, using entire historic period	11.8	76.3	67.7	54.8	3.2	91.4	88.2	66.7
Unadjusted, using TaR after historic visit	10.8	80.6	73.1	61.3	5.4	91.4	89.2	71.0
Age & sex adjusted, using TaR after historic visit	10.8	81.7	73.1	61.3	5.4	91.4	88.2	69.9
Unadjusted, using entire historic period, filtered	9.7	79.6	71.0	59.1	4.3	92.5	84.9	65.6
Age & sex adjusted, using entire historic period, filtered	8.6	79.6	71.0	58.1	4.3	92.5	83.9	64.5
Unadjusted, using TaR after historic visit, filtered	7.5	83.9	76.3	64.5	2.2	94.6	90.3	71.0
Age & sex adjusted, using TaR after historic visit, filtered	7.5	84.9	76.3	64.5	2.2	94.6	89.2	68.8
**SCCS / SCRI**								
Unadjusted SCCS excluding pre−vaccination window	10.8	76.3	68.8	50.5	4.3	87.1	74.2	54.8
Age & season adjusted SCCS excluding pre−vaccination window	9.7	77.4	69.9	51.6	3.2	83.9	73.1	53.8
SCRI with prior control interval	3.2	82.8	67.7	58.1	1.1	87.1	73.1	60.2
SCRI with posterior control interval	4.3	83.9	80.6	63.4	2.2	87.1	82.8	64.5
Unadjusted SCCS excluding all pre−vaccination time	6.5	75.3	71.0	51.6	5.4	83.9	73.1	54.8

Figure 36: Type 1 and 2 error based on $p < 0.05$ at the end of the study period in the MDCR database for Zoster vaccination (Shingrix).

	Uncalibrated				Calibrated			
		Type 2 when true effect is				Type 2 when true effect is		
	Type 1	1.5	2	4	Type 1	1.5	2	4
Case−control								
Age & sex matched controls	6.5	80.6	73.1	61.3	1.1	87.1	80.6	66.7
Age & sex adjusted, using random controls	3.2	88.2	79.6	58.1	2.2	94.6	87.1	61.3
Cohort method								
Unadjusted, using outpatient visits as comparator	2.2	94.6	88.2	62.4	2.2	89.2	78.5	52.7
PS matching, using outpatient visits as comparator	0.0	89.2	78.5	53.8	2.2	81.7	72.0	45.2
Unadjusted, using random days as comparator	19.4	66.7	60.2	48.4	1.1	93.5	89.2	61.3
PS matching, using random days as comparator	1.1	89.2	73.1	48.4	1.1	86.0	69.9	46.2
PS stratification, using outpatient visits as comparator	1.1	91.4	81.7	53.8	4.3	81.7	67.7	45.2
PS stratification, using random days as comparator	2.2	81.7	69.9	37.6	2.2	81.7	69.9	37.6
PS weighting, using outpatient visits as comparator	0.0	100.0	92.5	80.6	0.0	93.5	88.2	73.1
PS weighting, using random days as comparator	0.0	94.6	86.0	74.2	0.0	94.6	83.9	74.2
Per−month PS matching, using outpatient visits as comparator								
Per−month PS matching, using random days as comparator								
Historical comparator								
Unadjusted, using entire historic period	32.3	49.5	45.2	34.4	3.2	91.4	84.9	50.5
Age & sex adjusted, using entire historic period	14.0	68.8	57.0	33.3	3.2	92.5	74.2	44.1
Unadjusted, using TaR after historic visit	19.4	63.4	54.8	37.6	3.2	92.5	82.8	51.6
Age & sex adjusted, using TaR after historic visit	7.5	79.6	64.5	47.3	5.4	90.3	76.3	50.5
Unadjusted, using entire historic period, filtered	30.1	51.6	47.3	36.6	1.1	89.2	76.3	50.5
Age & sex adjusted, using entire historic period, filtered	11.8	71.0	59.1	35.5	3.2	76.3	66.7	38.7
Unadjusted, using TaR after historic visit, filtered	17.2	65.6	57.0	39.8	1.1	86.0	76.3	51.6
Age & sex adjusted, using TaR after historic visit, filtered	5.4	81.7	66.7	49.5	5.4	81.7	67.7	45.2
SCCS / SCRI								
Unadjusted SCCS excluding pre−vaccination window	8.6	71.0	61.3	33.3	3.2	74.2	62.4	35.5
Age & season adjusted SCCS excluding pre−vaccination window	6.5	69.9	64.5	35.5	2.2	75.3	66.7	35.5
SCRI with prior control interval	5.4	81.7	68.8	44.1	3.2	83.9	71.0	46.2
SCRI with posterior control interval	5.4	83.9	72.0	51.6	1.1	87.1	77.4	51.6
Unadjusted SCCS excluding all pre−vaccination time	5.4	74.2	59.1	38.7	2.2	77.4	64.5	43.0

Figure 37: Type 1 and 2 error based on $p < 0.05$ at the end of the study period in the CCAE database for Zoster vaccination (Shingrix).

10.6 Type 1 and 2 error for HPV vaccination (Gardasil 9)

	Uncalibrated				Calibrated			
		Type 2 when true effect is				Type 2 when true effect is		
	Type 1	1.5	2	4	Type 1	1.5	2	4
**Case−control**								
Age & sex matched controls	16.1	76.3	68.8	60.2	2.2	94.6	90.3	79.6
Age & sex adjusted, using random controls	6.5	84.9	80.6	66.7	3.2	95.7	93.5	80.6
**Cohort method**								
Unadjusted, using outpatient visits as comparator	2.2	95.7	93.5	81.7	2.2	95.7	89.2	78.5
PS matching, using outpatient visits as comparator	1.1	96.8	92.5	72.0	2.2	94.6	87.1	66.7
Unadjusted, using random days as comparator	5.4	88.2	82.8	75.3	3.2	95.7	93.5	79.6
PS matching, using random days as comparator								
PS stratification, using outpatient visits as comparator	1.1	96.8	92.5	66.7	2.2	92.5	84.9	58.1
PS stratification, using random days as comparator								
PS weighting, using outpatient visits as comparator	1.1	96.8	94.6	86.0	1.1	96.8	94.6	83.9
PS weighting, using random days as comparator								
Per−month PS matching, using outpatient visits as comparator								
Per−month PS matching, using random days as comparator								
**Historical comparator**								
Unadjusted, using entire historic period	15.1	76.3	75.3	68.8	2.2	95.7	88.2	76.3
Age & sex adjusted, using entire historic period	24.7	63.4	52.7	45.2	4.3	92.5	86.0	61.3
Unadjusted, using TaR after historic visit	5.4	88.2	80.6	73.1	2.2	93.5	90.3	78.5
Age & sex adjusted, using TaR after historic visit	6.5	81.7	73.1	60.2	3.2	92.5	80.6	63.4
Unadjusted, using entire historic period, filtered	14.0	77.4	76.3	69.9	2.2	95.7	88.2	77.4
Age & sex adjusted, using entire historic period, filtered	23.7	64.5	53.8	46.2	5.4	91.4	80.6	55.9
Unadjusted, using TaR after historic visit, filtered	5.4	88.2	81.7	74.2	2.2	93.5	90.3	80.6
Age & sex adjusted, using TaR after historic visit, filtered	5.4	82.8	74.2	61.3	2.2	91.4	79.6	61.3
**SCCS / SCRI**								
Unadjusted SCCS excluding pre−vaccination window	9.7	73.1	62.4	48.4	4.3	91.4	78.5	58.1
Age & season adjusted SCCS excluding pre−vaccination window	8.6	76.3	64.5	49.5	4.3	91.4	80.6	60.2
SCRI with prior control interval	2.2	84.9	75.3	67.7	1.1	96.8	87.1	71.0
SCRI with posterior control interval	5.4	83.9	75.3	65.6	2.2	93.5	83.9	68.8
Unadjusted SCCS excluding all pre−vaccination time	11.8	74.2	64.5	53.8	3.2	87.1	76.3	57.0

Figure 38: Type 1 and 2 error based on $p < 0.05$ at the end of the study period in the Optum EHR database for HPV vaccination (Gardasil 9).

	Uncalibrated				Calibrated			
		Type 2 when true effect is				Type 2 when true effect is		
	Type 1	1.5	2	4	Type 1	1.5	2	4
Case−control								
Age & sex matched controls	10.8	74.2	72.0	66.7	2.2	92.5	87.1	67.7
Age & sex adjusted, using random controls	8.6	77.4	74.2	65.6	1.1	92.5	80.6	67.7
Cohort method								
Unadjusted, using outpatient visits as comparator	2.2	94.6	92.5	84.9	2.2	95.7	92.5	86.0
PS matching, using outpatient visits as comparator	1.1	95.7	89.2	79.6	1.1	95.7	90.3	78.5
Unadjusted, using random days as comparator	4.3	88.2	82.8	79.6	1.1	97.8	93.5	80.6
PS matching, using random days as comparator	3.2	94.6	89.2	80.6	3.2	96.8	91.4	80.6
PS stratification, using outpatient visits as comparator	3.2	94.6	88.2	76.3	2.2	94.6	91.4	72.0
PS stratification, using random days as comparator	9.7	84.9	75.3	69.9	6.5	90.3	84.9	71.0
PS weighting, using outpatient visits as comparator	1.1	95.7	92.5	83.9	1.1	97.8	93.5	83.9
PS weighting, using random days as comparator	2.2	95.7	90.3	82.8	1.1	96.8	95.7	84.9
Per−month PS matching, using outpatient visits as comparator								
Per−month PS matching, using random days as comparator								
Historical comparator								
Unadjusted, using entire historic period	6.5	84.9	83.9	74.2	3.2	96.8	96.8	80.6
Age & sex adjusted, using entire historic period	10.8	77.4	72.0	62.4	2.2	93.5	90.3	74.2
Unadjusted, using TaR after historic visit	4.3	88.2	86.0	79.6	2.2	96.8	94.6	82.8
Age & sex adjusted, using TaR after historic visit	7.5	83.9	77.4	64.5	3.2	93.5	91.4	72.0
Unadjusted, using entire historic period, filtered	5.4	86.0	84.9	76.3	2.2	97.8	94.6	81.7
Age & sex adjusted, using entire historic period, filtered	8.6	79.6	74.2	64.5	4.3	93.5	87.1	68.8
Unadjusted, using TaR after historic visit, filtered	3.2	89.2	87.1	80.6	2.2	97.8	94.6	82.8
Age & sex adjusted, using TaR after historic visit, filtered	5.4	86.0	79.6	66.7	3.2	93.5	84.9	71.0
SCCS / SCRI								
Unadjusted SCCS excluding pre−vaccination window	6.5	77.4	73.1	61.3	3.2	88.2	77.4	63.4
Age & season adjusted SCCS excluding pre−vaccination window	5.4	79.6	74.2	62.4	3.2	89.2	79.6	63.4
SCRI with prior control interval	5.4	86.0	79.6	68.8	2.2	95.7	92.5	74.2
SCRI with posterior control interval	4.3	86.0	81.7	72.0	2.2	95.7	94.6	74.2
Unadjusted SCCS excluding all pre−vaccination time	6.5	76.3	69.9	60.2	2.2	95.7	88.2	67.7

Figure 39: Type 1 and 2 error based on $p < 0.05$ at the end of the study period in the MDCD database for HPV vaccination (Gardasil 9).

	Uncalibrated				Calibrated			
		Type 2 when true effect is				Type 2 when true effect is		
	Type 1	1.5	2	4	Type 1	1.5	2	4
Case−control								
Age & sex matched controls								
Age & sex adjusted, using random controls								
Cohort method								
Unadjusted, using outpatient visits as comparator	0.0	100.0	100.0	100.0	0.0	100.0	100.0	100.0
PS matching, using outpatient visits as comparator								
Unadjusted, using random days as comparator								
PS matching, using random days as comparator								
PS stratification, using outpatient visits as comparator	0.0	100.0	100.0	100.0	0.0	100.0	100.0	100.0
PS stratification, using random days as comparator								
PS weighting, using outpatient visits as comparator	0.0	100.0	100.0	100.0	0.0	100.0	100.0	100.0
PS weighting, using random days as comparator								
Per−month PS matching, using outpatient visits as comparator								
Per−month PS matching, using random days as comparator								
Historical comparator								
Unadjusted, using entire historic period	0.0	100.0	100.0	100.0	0.0	100.0	100.0	100.0
Age & sex adjusted, using entire historic period	0.0	100.0	100.0	100.0	0.0	100.0	100.0	100.0
Unadjusted, using TaR after historic visit	0.0	100.0	100.0	100.0	0.0	100.0	100.0	100.0
Age & sex adjusted, using TaR after historic visit	0.0	100.0	100.0	100.0	0.0	100.0	100.0	100.0
Unadjusted, using entire historic period, filtered	0.0	100.0	100.0	100.0	0.0	100.0	100.0	100.0
Age & sex adjusted, using entire historic period, filtered	0.0	100.0	100.0	100.0	0.0	100.0	100.0	100.0
Unadjusted, using TaR after historic visit, filtered	0.0	100.0	100.0	100.0	0.0	100.0	100.0	100.0
Age & sex adjusted, using TaR after historic visit, filtered	0.0	100.0	100.0	100.0	0.0	100.0	100.0	100.0

Figure 40: Type 1 and 2 error based on $p < 0.05$ at the end of the study period in the MDCR database for HPV vaccination (Gardasil 9).

	Uncalibrated				Calibrated			
		Type 2 when true effect is				Type 2 when true effect is		
	Type 1	1.5	2	4	Type 1	1.5	2	4
Case−control								
Age & sex matched controls	12.9	77.4	67.7	57.0	3.2	91.4	80.6	65.6
Age & sex adjusted, using random controls	5.4	81.7	75.3	64.5	3.2	94.6	86.0	72.0
Cohort method								
Unadjusted, using outpatient visits as comparator	2.2	96.8	89.2	84.9	2.2	95.7	89.2	78.5
PS matching, using outpatient visits as comparator	0.0	97.8	86.0	68.8	1.1	94.6	83.9	67.7
Unadjusted, using random days as comparator	4.3	89.2	86.0	77.4	2.2	95.7	92.5	78.5
PS matching, using random days as comparator	2.2	93.5	88.2	72.0	2.2	94.6	88.2	72.0
PS stratification, using outpatient visits as comparator	1.1	93.5	87.1	64.5	3.2	90.3	78.5	59.1
PS stratification, using random days as comparator	2.2	88.2	82.8	61.3	2.2	91.4	83.9	60.2
PS weighting, using outpatient visits as comparator	0.0	97.8	95.7	83.9	0.0	97.8	93.5	82.8
PS weighting, using random days as comparator	1.1	95.7	93.5	81.7	1.1	97.8	94.6	83.9
Per−month PS matching, using outpatient visits as comparator								
Per−month PS matching, using random days as comparator								
Historical comparator								
Unadjusted, using entire historic period	7.5	84.9	80.6	67.7	1.1	95.7	89.2	75.3
Age & sex adjusted, using entire historic period	9.7	75.3	61.3	45.2	1.1	92.5	84.9	57.0
Unadjusted, using TaR after historic visit	8.6	87.1	82.8	76.3	1.1	96.8	88.2	78.5
Age & sex adjusted, using TaR after historic visit	4.3	82.8	77.4	52.7	3.2	93.5	83.9	59.1
Unadjusted, using entire historic period, filtered	7.5	86.0	81.7	69.9	1.1	95.7	89.2	77.4
Age & sex adjusted, using entire historic period, filtered	7.5	77.4	63.4	47.3	3.2	88.2	79.6	52.7
Unadjusted, using TaR after historic visit, filtered	8.6	87.1	83.9	78.5	1.1	96.8	89.2	79.6
Age & sex adjusted, using TaR after historic visit, filtered	3.2	84.9	79.6	54.8	3.2	91.4	82.8	58.1
SCCS / SCRI								
Unadjusted SCCS excluding pre−vaccination window	10.8	76.3	60.2	48.4	1.1	87.1	75.3	53.8
Age & season adjusted SCCS excluding pre−vaccination window	10.8	77.4	60.2	48.4	2.2	86.0	73.1	53.8
SCRI with prior control interval	4.3	87.1	78.5	61.3	2.2	94.6	89.2	63.4
SCRI with posterior control interval	3.2	89.2	77.4	63.4	1.1	94.6	90.3	65.6
Unadjusted SCCS excluding all pre−vaccination time	9.7	75.3	63.4	49.5	2.2	91.4	81.7	58.1

Figure 41: Type 1 and 2 error based on $p < 0.05$ at the end of the study period in the CCAE database for HPV vaccination (Gardasil 9).

11 Type 1 and 2 error by true effect size based on MaxSPRT

For each method variation and vaccine group, the type 1 error and the type 2 error stratified by true effect size, based on whether the log likelihood ratio exceeded the critical value any time during the study period.

11.1 Type 1 and 2 error for H1N1 vaccination

	Uncalibrated				Calibrated			
		Type 2 when true effect is				Type 2 when true effect is		
	Type 1	1.5	2	4	Type 1	1.5	2	4
Case−control								
Age & sex matched controls	29.0	68.8	64.5	52.7	4.3	83.9	77.4	69.9
Age & sex adjusted, using random controls	29.0	66.7	61.3	53.8	4.3	89.2	87.1	73.1
Cohort method								
Unadjusted, using outpatient visits as comparator	4.3	90.3	88.2	76.3	7.5	91.4	88.2	71.0
PS matching, using outpatient visits as comparator	0.0	96.8	91.4	73.1	3.2	90.3	86.0	65.6
Unadjusted, using random days as comparator	20.4	79.6	75.3	66.7	3.2	95.7	91.4	82.8
PS matching, using random days as comparator	1.1	98.9	97.8	95.7	0.0	100.0	98.9	95.7
PS stratification, using outpatient visits as comparator	0.0	93.5	83.9	63.4	4.3	87.1	79.6	51.6
PS stratification, using random days as comparator	35.5	52.7	49.5	41.9	0.0	89.2	80.6	58.1
PS weighting, using outpatient visits as comparator	5.4	87.1	80.6	61.3	5.4	90.3	81.7	62.4
PS weighting, using random days as comparator	0.0	100.0	100.0	97.8	0.0	100.0	100.0	97.8
Per−month PS matching, using outpatient visits as comparator	1.1	96.8	96.8	90.3	3.2	96.8	95.7	90.3
Per−month PS matching, using random days as comparator	1.1	97.8	96.8	89.2	0.0	97.8	96.8	90.3
Historical comparator								
Unadjusted, using entire historic period	36.6	61.3	59.1	55.9	4.3	88.2	79.6	63.4
Age & sex adjusted, using entire historic period	33.3	61.3	61.3	58.1	4.3	87.1	73.1	63.4
Unadjusted, using TaR after historic visit	16.1	71.0	58.1	46.2	5.4	89.2	84.9	55.9
Age & sex adjusted, using TaR after historic visit	18.3	59.1	49.5	46.2	5.4	83.9	76.3	47.3
Unadjusted, using entire historic period, filtered	36.6	61.3	59.1	55.9	4.3	88.2	76.3	63.4
Age & sex adjusted, using entire historic period, filtered	33.3	61.3	61.3	58.1	4.3	86.0	72.0	63.4
Unadjusted, using TaR after historic visit, filtered	16.1	72.0	60.2	48.4	5.4	89.2	86.0	58.1
Age & sex adjusted, using TaR after historic visit, filtered	18.3	59.1	50.5	47.3	6.5	82.8	76.3	48.4
SCCS / SCRI								
Unadjusted SCCS excluding pre−vaccination window	4.3	83.9	67.7	47.3	4.3	87.1	67.7	47.3
Age & season adjusted SCCS excluding pre−vaccination window	2.2	79.6	61.3	43.0	4.3	76.3	60.2	41.9
SCRI with prior control interval	3.2	91.4	86.0	64.5	0.0	93.5	86.0	65.6
SCRI with posterior control interval	4.3	91.4	83.9	61.3	3.2	92.5	88.2	61.3
Unadjusted SCCS excluding all pre−vaccination time	6.5	83.9	62.4	45.2	3.2	89.2	73.1	46.2

Figure 42: Type 1 and 2 error based on whether the log likelihood ratio exceeded the critical value any time during the study period in the Optum EHR database for H1N1 vaccination.

	Uncalibrated				Calibrated			
		Type 2 when true effect is				Type 2 when true effect is		
	Type 1	1.5	2	4	Type 1	1.5	2	4
Case−control								
Age & sex matched controls	15.1	69.9	64.5	52.7	4.3	84.9	75.3	59.1
Age & sex adjusted, using random controls	9.7	75.3	67.7	54.8	2.2	86.0	75.3	57.0
Cohort method								
Unadjusted, using outpatient visits as comparator	3.2	57.0	66.7	81.7	4.3	95.7	94.6	91.4
PS matching, using outpatient visits as comparator	0.0	91.4	89.2	76.3	4.3	92.5	88.2	74.2
Unadjusted, using random days as comparator	3.2	86.0	91.4	82.8	2.2	95.7	93.5	84.9
PS matching, using random days as comparator	3.2	88.2	87.1	72.0	4.3	88.2	87.1	72.0
PS stratification, using outpatient visits as comparator	4.3	83.9	76.3	68.8	6.5	89.2	77.4	67.7
PS stratification, using random days as comparator	4.3	86.0	80.6	64.5	3.2	88.2	81.7	65.6
PS weighting, using outpatient visits as comparator	6.5	53.8	52.7	57.0	7.5	81.7	78.5	63.4
PS weighting, using random days as comparator	0.0	97.8	94.6	86.0	0.0	96.8	92.5	86.0
Per−month PS matching, using outpatient visits as comparator	1.1	95.7	89.2	79.6	2.2	95.7	87.1	76.3
Per−month PS matching, using random days as comparator	1.1	92.5	88.2	78.5	1.1	92.5	88.2	78.5
Historical comparator								
Unadjusted, using entire historic period	4.3	76.3	82.8	68.8	2.2	93.5	89.2	75.3
Age & sex adjusted, using entire historic period	7.5	73.1	67.7	57.0	0.0	94.6	79.6	63.4
Unadjusted, using TaR after historic visit	4.3	71.0	77.4	78.5	3.2	94.6	92.5	80.6
Age & sex adjusted, using TaR after historic visit	5.4	79.6	68.8	59.1	1.1	92.5	83.9	63.4
Unadjusted, using entire historic period, filtered	3.2	76.3	82.8	68.8	2.2	94.6	89.2	72.0
Age & sex adjusted, using entire historic period, filtered	7.5	73.1	68.8	57.0	0.0	92.5	79.6	63.4
Unadjusted, using TaR after historic visit, filtered	3.2	71.0	77.4	78.5	3.2	94.6	93.5	77.4
Age & sex adjusted, using TaR after historic visit, filtered	5.4	80.6	68.8	59.1	1.1	92.5	81.7	62.4
SCCS / SCRI								
Unadjusted SCCS excluding pre−vaccination window	4.3	84.9	77.4	57.0	4.3	92.5	81.7	57.0
Age & season adjusted SCCS excluding pre−vaccination window	4.3	81.7	69.9	51.6	6.5	78.5	67.7	48.4
SCRI with prior control interval	4.3	90.3	84.9	67.7	3.2	95.7	87.1	67.7
SCRI with posterior control interval	6.5	93.5	90.3	66.7	4.3	93.5	90.3	67.7
Unadjusted SCCS excluding all pre−vaccination time	6.5	87.1	77.4	55.9	3.2	89.2	80.6	57.0

Figure 43: Type 1 and 2 error based on whether the log likelihood ratio exceeded the critical value any time during the study period in the MDCD database for H1N1 vaccination.

	Uncalibrated				Calibrated			
		Type 2 when true effect is				Type 2 when true effect is		
	Type 1	1.5	2	4	Type 1	1.5	2	4
Case−control								
Age & sex matched controls	15.1	83.9	76.3	71.0	3.2	96.8	86.0	76.3
Age & sex adjusted, using random controls	11.8	86.0	80.6	74.2	5.4	92.5	89.2	79.6
Cohort method								
Unadjusted, using outpatient visits as comparator	0.0	97.8	95.7	91.4	1.1	96.8	94.6	89.2
PS matching, using outpatient visits as comparator	1.1	98.9	95.7	95.7	1.1	97.8	95.7	93.5
Unadjusted, using random days as comparator	2.2	92.5	92.5	89.2	1.1	98.9	93.5	89.2
PS matching, using random days as comparator	0.0	97.8	95.7	93.5	0.0	98.9	96.8	92.5
PS stratification, using outpatient visits as comparator	0.0	91.4	83.9	75.3	4.3	90.3	84.9	74.2
PS stratification, using random days as comparator	5.4	80.6	81.7	78.5	2.2	95.7	87.1	79.6
PS weighting, using outpatient visits as comparator	7.5	89.2	89.2	82.8	2.2	97.8	97.8	89.2
PS weighting, using random days as comparator	0.0	100.0	100.0	97.8	0.0	100.0	100.0	97.8
Per−month PS matching, using outpatient visits as comparator	0.0	98.9	97.8	93.5	0.0	97.8	97.8	93.5
Per−month PS matching, using random days as comparator	0.0	98.9	96.8	95.7	0.0	97.8	96.8	95.7
Historical comparator								
Unadjusted, using entire historic period	7.5	84.9	82.8	81.7	1.1	98.9	96.8	84.9
Age & sex adjusted, using entire historic period	8.6	83.9	82.8	79.6	1.1	98.9	96.8	83.9
Unadjusted, using TaR after historic visit	2.2	87.1	83.9	80.6	1.1	98.9	95.7	83.9
Age & sex adjusted, using TaR after historic visit	4.3	86.0	81.7	79.6	1.1	96.8	94.6	81.7
Unadjusted, using entire historic period, filtered	7.5	84.9	82.8	81.7	1.1	98.9	96.8	84.9
Age & sex adjusted, using entire historic period, filtered	8.6	83.9	82.8	79.6	1.1	97.8	94.6	82.8
Unadjusted, using TaR after historic visit, filtered	2.2	87.1	83.9	80.6	1.1	98.9	95.7	82.8
Age & sex adjusted, using TaR after historic visit, filtered	4.3	86.0	81.7	79.6	1.1	96.8	94.6	81.7
SCCS / SCRI								
Unadjusted SCCS excluding pre−vaccination window	5.4	92.5	82.8	71.0	4.3	93.5	87.1	73.1
Age & season adjusted SCCS excluding pre−vaccination window	7.5	84.9	74.2	68.8	7.5	88.2	79.6	69.9
SCRI with prior control interval	2.2	96.8	95.7	84.9	0.0	97.8	96.8	88.2
SCRI with posterior control interval	0.0	97.8	95.7	88.2	1.1	97.8	95.7	86.0
Unadjusted SCCS excluding all pre−vaccination time	3.2	95.7	86.0	74.2	3.2	96.8	89.2	75.3

Figure 44: Type 1 and 2 error based on whether the log likelihood ratio exceeded the critical value any time during the study period in the MDCR database for H1N1 vaccination.

	Uncalibrated				Calibrated			
		Type 2 when true effect is				Type 2 when true effect is		
	Type 1	1.5	2	4	Type 1	1.5	2	4
Case−control								
Age & sex matched controls	21.5	73.1	65.6	53.8	3.2	84.9	75.3	61.3
Age & sex adjusted, using random controls	19.4	67.7	61.3	53.8	2.2	90.3	75.3	60.2
Cohort method								
Unadjusted, using outpatient visits as comparator	4.3	49.5	54.8	71.0	5.4	92.5	90.3	77.4
PS matching, using outpatient visits as comparator	0.0	87.1	79.6	45.2	5.4	87.1	66.7	33.3
Unadjusted, using random days as comparator	10.8	73.1	62.4	37.6	3.2	92.5	83.9	49.5
PS matching, using random days as comparator	5.4	78.5	63.4	40.9	5.4	80.6	64.5	40.9
PS stratification, using outpatient visits as comparator	1.1	84.9	76.3	41.9	4.3	82.8	63.4	33.3
PS stratification, using random days as comparator	6.5	75.3	59.1	37.6	5.4	83.9	63.4	40.9
PS weighting, using outpatient visits as comparator	6.5	67.7	67.7	47.3	2.2	83.9	73.1	44.1
PS weighting, using random days as comparator	1.1	91.4	84.9	68.8	2.2	87.1	81.7	66.7
Per−month PS matching, using outpatient visits as comparator	1.1	91.4	81.7	50.5	5.4	87.1	67.7	43.0
Per−month PS matching, using random days as comparator	4.3	80.6	71.0	49.5	4.3	84.9	72.0	49.5
Historical comparator								
Unadjusted, using entire historic period	16.1	61.3	44.1	29.0	6.5	86.0	77.4	29.0
Age & sex adjusted, using entire historic period	29.0	40.9	34.4	29.0	5.4	87.1	66.7	33.3
Unadjusted, using TaR after historic visit	10.8	69.9	66.7	33.3	6.5	88.2	81.7	44.1
Age & sex adjusted, using TaR after historic visit	11.8	64.5	48.4	29.0	5.4	80.6	64.5	29.0
Unadjusted, using entire historic period, filtered	16.1	62.4	45.2	30.1	6.5	87.1	78.5	30.1
Age & sex adjusted, using entire historic period, filtered	28.0	41.9	35.5	30.1	4.3	88.2	65.6	34.4
Unadjusted, using TaR after historic visit, filtered	10.8	69.9	67.7	34.4	5.4	88.2	82.8	45.2
Age & sex adjusted, using TaR after historic visit, filtered	11.8	65.6	49.5	30.1	5.4	81.7	65.6	30.1
SCCS / SCRI								
Unadjusted SCCS excluding pre−vaccination window	5.4	69.9	53.8	28.0	2.2	83.9	59.1	28.0
Age & season adjusted SCCS excluding pre−vaccination window	6.5	63.4	49.5	26.9	5.4	68.8	50.5	28.0
SCRI with prior control interval	6.5	77.4	65.6	40.9	2.2	87.1	73.1	46.2
SCRI with posterior control interval	10.8	71.0	60.2	35.5	5.4	81.7	66.7	37.6
Unadjusted SCCS excluding all pre−vaccination time	12.9	68.8	50.5	29.0	4.3	80.6	58.1	31.2

Figure 45: Type 1 and 2 error based on whether the log likelihood ratio exceeded the critical value any time during the study period in the CCAE database for H1N1 vaccination.

11.2 Type 1 and 2 error for Seasonal flu vaccination (Fluvirin)

	Uncalibrated				Calibrated			
		Type 2 when true effect is				Type 2 when true effect is		
	Type 1	1.5	2	4	Type 1	1.5	2	4
Case−control								
Age & sex matched controls	5.4	95.7	95.7	87.1	1.1	100.0	100.0	95.7
Age & sex adjusted, using random controls	4.3	95.7	93.5	91.4	0.0	100.0	98.9	97.8
Cohort method								
Unadjusted, using outpatient visits as comparator	1.1	97.8	97.8	95.7	3.2	95.7	95.7	87.1
PS matching, using outpatient visits as comparator	0.0	100.0	100.0	98.9	1.1	100.0	100.0	95.7
Unadjusted, using random days as comparator	1.1	98.9	96.8	88.2	0.0	100.0	100.0	95.7
PS matching, using random days as comparator								
PS stratification, using outpatient visits as comparator	2.2	96.8	96.8	92.5	2.2	96.8	93.5	89.2
PS stratification, using random days as comparator	9.7	87.1	83.9	73.1	2.2	96.8	91.4	79.6
PS weighting, using outpatient visits as comparator	0.0	100.0	98.9	94.6	0.0	98.9	97.8	92.5
PS weighting, using random days as comparator	0.0	100.0	100.0	100.0	0.0	100.0	100.0	100.0
Per−month PS matching, using outpatient visits as comparator								
Per−month PS matching, using random days as comparator								
Historical comparator								
Unadjusted, using entire historic period	5.4	89.2	88.2	87.1	0.0	100.0	100.0	93.5
Age & sex adjusted, using entire historic period	8.6	88.2	84.9	84.9	0.0	98.9	97.8	90.3
Unadjusted, using TaR after historic visit	6.5	87.1	83.9	76.3	1.1	98.9	93.5	84.9
Age & sex adjusted, using TaR after historic visit	6.5	87.1	83.9	75.3	1.1	96.8	93.5	84.9
Unadjusted, using entire historic period, filtered	5.4	89.2	88.2	87.1	0.0	100.0	97.8	90.3
Age & sex adjusted, using entire historic period, filtered	8.6	88.2	84.9	84.9	0.0	98.9	96.8	89.2
Unadjusted, using TaR after historic visit, filtered	6.5	87.1	83.9	76.3	1.1	98.9	93.5	84.9
Age & sex adjusted, using TaR after historic visit, filtered	6.5	87.1	83.9	75.3	1.1	96.8	92.5	83.9
SCCS / SCRI								
Unadjusted SCCS excluding pre−vaccination window	3.2	92.5	88.2	82.8	3.2	95.7	94.6	83.9
Age & season adjusted SCCS excluding pre−vaccination window	2.2	91.4	86.0	78.5	3.2	96.8	92.5	81.7
SCRI with prior control interval	3.2	100.0	98.9	90.3	1.1	100.0	98.9	93.5
SCRI with posterior control interval	2.2	100.0	97.8	88.2	1.1	100.0	97.8	92.5
Unadjusted SCCS excluding all pre−vaccination time	2.2	93.5	88.2	75.3	1.1	96.8	94.6	81.7

Figure 46: Type 1 and 2 error based on whether the log likelihood ratio exceeded the critical value any time during the study period in the Optum EHR database for Seasonal flu vaccination (Fluvirin).

	Uncalibrated				Calibrated			
		Type 2 when true effect is				Type 2 when true effect is		
	Type 1	1.5	2	4	Type 1	1.5	2	4
Case−control								
Age & sex matched controls	8.6	88.2	87.1	80.6	6.5	96.8	92.5	83.9
Age & sex adjusted, using random controls	1.1	90.3	88.2	82.8	1.1	100.0	94.6	83.9
Cohort method								
Unadjusted, using outpatient visits as comparator	0.0	98.9	95.7	88.2	1.1	95.7	94.6	87.1
PS matching, using outpatient visits as comparator	0.0	98.9	98.9	97.8	1.1	98.9	97.8	94.6
Unadjusted, using random days as comparator	6.5	93.5	91.4	88.2	2.2	97.8	96.8	91.4
PS matching, using random days as comparator	0.0	97.8	97.8	91.4	1.1	98.9	95.7	89.2
PS stratification, using outpatient visits as comparator	0.0	92.5	86.0	79.6	3.2	88.2	87.1	77.4
PS stratification, using random days as comparator	5.4	84.9	81.7	76.3	1.1	95.7	88.2	77.4
PS weighting, using outpatient visits as comparator	0.0	98.9	97.8	95.7	0.0	98.9	97.8	94.6
PS weighting, using random days as comparator	0.0	100.0	100.0	96.8	0.0	100.0	100.0	96.8
Per−month PS matching, using outpatient visits as comparator								
Per−month PS matching, using random days as comparator								
Historical comparator								
Unadjusted, using entire historic period	7.5	88.2	86.0	82.8	0.0	97.8	96.8	91.4
Age & sex adjusted, using entire historic period	4.3	86.0	83.9	78.5	1.1	95.7	91.4	88.2
Unadjusted, using TaR after historic visit	4.3	88.2	82.8	79.6	0.0	96.8	95.7	87.1
Age & sex adjusted, using TaR after historic visit	3.2	93.5	92.5	80.6	2.2	95.7	93.5	90.3
Unadjusted, using entire historic period, filtered	7.5	88.2	86.0	82.8	2.2	96.8	93.5	87.1
Age & sex adjusted, using entire historic period, filtered	4.3	86.0	83.9	78.5	2.2	93.5	89.2	80.6
Unadjusted, using TaR after historic visit, filtered	4.3	88.2	82.8	79.6	2.2	95.7	92.5	80.6
Age & sex adjusted, using TaR after historic visit, filtered	3.2	93.5	92.5	80.6	3.2	94.6	93.5	82.8
SCCS / SCRI								
Unadjusted SCCS excluding pre−vaccination window	1.1	97.8	89.2	79.6	1.1	98.9	89.2	80.6
Age & season adjusted SCCS excluding pre−vaccination window	1.1	93.5	88.2	80.6	1.1	97.8	88.2	80.6
SCRI with prior control interval	2.2	98.9	97.8	91.4	0.0	97.8	94.6	90.3
SCRI with posterior control interval	1.1	95.7	95.7	87.1	0.0	95.7	95.7	88.2
Unadjusted SCCS excluding all pre−vaccination time	0.0	95.7	92.5	79.6	0.0	95.7	93.5	79.6

Figure 47: Type 1 and 2 error based on whether the log likelihood ratio exceeded the critical value any time during the study period in the MDCD database for Seasonal flu vaccination (Fluvirin).

	Uncalibrated				Calibrated			
		Type 2 when true effect is				Type 2 when true effect is		
	Type 1	1.5	2	4	Type 1	1.5	2	4
Case−control								
Age & sex matched controls	0.0	100.0	100.0	98.9	0.0	100.0	100.0	100.0
Age & sex adjusted, using random controls	0.0	100.0	100.0	100.0	0.0	100.0	100.0	100.0
Cohort method								
Unadjusted, using outpatient visits as comparator	0.0	100.0	100.0	100.0	0.0	100.0	100.0	100.0
PS matching, using outpatient visits as comparator	0.0	100.0	100.0	100.0	0.0	100.0	100.0	100.0
Unadjusted, using random days as comparator	0.0	100.0	100.0	100.0	0.0	100.0	100.0	100.0
PS matching, using random days as comparator	0.0	100.0	100.0	100.0	0.0	100.0	100.0	100.0
PS stratification, using outpatient visits as comparator	0.0	100.0	98.9	96.8	0.0	100.0	100.0	98.9
PS stratification, using random days as comparator	0.0	100.0	100.0	98.9	0.0	100.0	100.0	100.0
PS weighting, using outpatient visits as comparator	1.1	98.9	98.9	98.9	1.1	100.0	100.0	98.9
PS weighting, using random days as comparator	0.0	100.0	100.0	100.0	0.0	100.0	100.0	100.0
Per−month PS matching, using outpatient visits as comparator								
Per−month PS matching, using random days as comparator								
Historical comparator								
Unadjusted, using entire historic period	0.0	98.9	98.9	98.9	0.0	100.0	100.0	98.9
Age & sex adjusted, using entire historic period	0.0	98.9	98.9	98.9	0.0	100.0	100.0	98.9
Unadjusted, using TaR after historic visit	0.0	98.9	98.9	98.9	0.0	100.0	100.0	98.9
Age & sex adjusted, using TaR after historic visit	0.0	98.9	98.9	98.9	0.0	100.0	100.0	98.9
Unadjusted, using entire historic period, filtered	0.0	98.9	98.9	98.9	0.0	100.0	100.0	98.9
Age & sex adjusted, using entire historic period, filtered	0.0	98.9	98.9	98.9	0.0	100.0	100.0	98.9
Unadjusted, using TaR after historic visit, filtered	0.0	98.9	98.9	98.9	0.0	100.0	100.0	98.9
Age & sex adjusted, using TaR after historic visit, filtered	0.0	98.9	98.9	98.9	0.0	100.0	100.0	98.9
SCCS / SCRI								
Unadjusted SCCS excluding pre−vaccination window	0.0	100.0	98.9	97.8	0.0	100.0	100.0	100.0
Age & season adjusted SCCS excluding pre−vaccination window	0.0	100.0	100.0	98.9	0.0	100.0	100.0	100.0
SCRI with prior control interval	0.0	100.0	100.0	100.0	0.0	100.0	100.0	100.0
SCRI with posterior control interval	0.0	100.0	100.0	100.0	0.0	100.0	100.0	100.0
Unadjusted SCCS excluding all pre−vaccination time	0.0	98.9	98.9	97.8	0.0	100.0	100.0	100.0

Figure 48: Type 1 and 2 error based on whether the log likelihood ratio exceeded the critical value any time during the study period in the MDCR database for Seasonal flu vaccination (Fluvirin).

	Uncalibrated				Calibrated			
		Type 2 when true effect is				Type 2 when true effect is		
	Type 1	1.5	2	4	Type 1	1.5	2	4
Case−control								
Age & sex matched controls	6.5	84.9	83.9	72.0	2.2	94.6	83.9	75.3
Age & sex adjusted, using random controls	4.3	88.2	82.8	71.0	3.2	90.3	84.9	75.3
Cohort method								
Unadjusted, using outpatient visits as comparator	0.0	87.1	89.2	79.6	4.3	92.5	86.0	72.0
PS matching, using outpatient visits as comparator	3.2	90.3	87.1	74.2	5.4	88.2	83.9	73.1
Unadjusted, using random days as comparator	11.8	86.0	80.6	73.1	3.2	89.2	86.0	76.3
PS matching, using random days as comparator	2.2	84.9	80.6	67.7	2.2	87.1	80.6	68.8
PS stratification, using outpatient visits as comparator	2.2	86.0	79.6	63.4	4.3	84.9	74.2	59.1
PS stratification, using random days as comparator	5.4	79.6	72.0	59.1	3.2	81.7	75.3	60.2
PS weighting, using outpatient visits as comparator	3.2	88.2	78.5	66.7	3.2	92.5	80.6	64.5
PS weighting, using random days as comparator	0.0	100.0	90.3	83.9	0.0	100.0	95.7	84.9
Per−month PS matching, using outpatient visits as comparator								
Per−month PS matching, using random days as comparator								
Historical comparator								
Unadjusted, using entire historic period	16.1	72.0	64.5	54.8	2.2	95.7	92.5	78.5
Age & sex adjusted, using entire historic period	12.9	73.1	64.5	50.5	2.2	93.5	90.3	69.9
Unadjusted, using TaR after historic visit	8.6	78.5	68.8	53.8	1.1	95.7	93.5	76.3
Age & sex adjusted, using TaR after historic visit	8.6	76.3	71.0	53.8	4.3	92.5	89.2	75.3
Unadjusted, using entire historic period, filtered	15.1	73.1	65.6	55.9	3.2	83.9	75.3	61.3
Age & sex adjusted, using entire historic period, filtered	10.8	75.3	66.7	52.7	1.1	78.5	73.1	57.0
Unadjusted, using TaR after historic visit, filtered	7.5	79.6	69.9	54.8	3.2	81.7	74.2	55.9
Age & sex adjusted, using TaR after historic visit, filtered	6.5	78.5	73.1	55.9	5.4	81.7	75.3	55.9
SCCS / SCRI								
Unadjusted SCCS excluding pre−vaccination window	6.5	77.4	67.7	55.9	4.3	80.6	73.1	58.1
Age & season adjusted SCCS excluding pre−vaccination window	9.7	72.0	65.6	51.6	4.3	75.3	67.7	57.0
SCRI with prior control interval	4.3	90.3	84.9	68.8	2.2	95.7	88.2	74.2
SCRI with posterior control interval	7.5	86.0	82.8	72.0	2.2	90.3	83.9	72.0
Unadjusted SCCS excluding all pre−vaccination time	8.6	78.5	68.8	58.1	2.2	84.9	73.1	61.3

Figure 49: Type 1 and 2 error based on whether the log likelihood ratio exceeded the critical value any time during the study period in the CCAE database for Seasonal flu vaccination (Fluvirin).

11.3 Type 1 and 2 error for Seasonal flu vaccination (Fluzone)

	Uncalibrated				Calibrated			
		Type 2 when true effect is				Type 2 when true effect is		
	Type 1	1.5	2	4	Type 1	1.5	2	4
Case−control								
Age & sex matched controls	30.1	60.2	52.7	37.6	8.6	87.1	79.6	53.8
Age & sex adjusted, using random controls	34.4	49.5	45.2	38.7	5.4	86.0	77.4	48.4
Cohort method								
Unadjusted, using outpatient visits as comparator	3.2	72.0	76.3	49.5	5.4	90.3	77.4	41.9
PS matching, using outpatient visits as comparator	2.2	88.2	76.3	47.3	10.8	77.4	62.4	37.6
Unadjusted, using random days as comparator	34.4	51.6	46.2	32.3	4.3	91.4	80.6	48.4
PS matching, using random days as comparator								
PS stratification, using outpatient visits as comparator	1.1	82.8	73.1	39.8	8.6	76.3	60.2	34.4
PS stratification, using random days as comparator	36.6	39.8	30.1	20.4	4.3	83.9	75.3	34.4
PS weighting, using outpatient visits as comparator	1.1	86.0	77.4	54.8	3.2	83.9	68.8	43.0
PS weighting, using random days as comparator	0.0	100.0	100.0	100.0	0.0	100.0	100.0	100.0
Per−month PS matching, using outpatient visits as comparator								
Per−month PS matching, using random days as comparator								
Historical comparator								
Unadjusted, using entire historic period	58.1	29.0	25.8	19.4	3.2	92.5	76.3	41.9
Age & sex adjusted, using entire historic period	54.8	31.2	28.0	21.5	1.1	91.4	76.3	43.0
Unadjusted, using TaR after historic visit	25.8	52.7	40.9	24.7	2.2	90.3	81.7	45.2
Age & sex adjusted, using TaR after historic visit	23.7	48.4	39.8	24.7	3.2	90.3	78.5	35.5
Unadjusted, using entire historic period, filtered	58.1	29.0	25.8	20.4	4.3	80.6	63.4	32.3
Age & sex adjusted, using entire historic period, filtered	53.8	31.2	28.0	22.6	6.5	82.8	65.6	31.2
Unadjusted, using TaR after historic visit, filtered	23.7	53.8	40.9	25.8	2.2	80.6	63.4	31.2
Age & sex adjusted, using TaR after historic visit, filtered	22.6	49.5	40.9	25.8	6.5	81.7	66.7	26.9
SCCS / SCRI								
Unadjusted SCCS excluding pre−vaccination window	17.2	47.3	38.7	23.7	4.3	81.7	59.1	26.9
Age & season adjusted SCCS excluding pre−vaccination window	23.7	43.0	33.3	21.5	6.5	80.6	58.1	28.0
SCRI with prior control interval	28.0	61.3	55.9	30.1	3.2	90.3	81.7	52.7
SCRI with posterior control interval	18.3	64.5	50.5	33.3	7.5	86.0	69.9	46.2
Unadjusted SCCS excluding all pre−vaccination time	22.6	54.8	40.9	22.6	8.6	82.8	64.5	29.0

Figure 50: Type 1 and 2 error based on whether the log likelihood ratio exceeded the critical value any time during the study period in the Optum EHR database for Seasonal flu vaccination (Fluzone).

	Uncalibrated				Calibrated			
		Type 2 when true effect is				Type 2 when true effect is		
	Type 1	1.5	2	4	Type 1	1.5	2	4
Case−control								
Age & sex matched controls	3.2	97.8	96.8	91.4	0.0	98.9	98.9	97.8
Age & sex adjusted, using random controls	3.2	96.8	96.8	92.5	0.0	100.0	100.0	96.8
Cohort method								
Unadjusted, using outpatient visits as comparator	0.0	100.0	98.9	98.9	0.0	100.0	98.9	98.9
PS matching, using outpatient visits as comparator	0.0	100.0	100.0	98.9	0.0	100.0	100.0	100.0
Unadjusted, using random days as comparator	1.1	98.9	97.8	97.8	1.1	100.0	98.9	97.8
PS matching, using random days as comparator								
PS stratification, using outpatient visits as comparator	1.1	95.7	92.5	90.3	1.1	96.8	96.8	90.3
PS stratification, using random days as comparator	4.3	95.7	91.4	87.1	0.0	100.0	98.9	91.4
PS weighting, using outpatient visits as comparator	0.0	100.0	97.8	95.7	0.0	100.0	100.0	96.8
PS weighting, using random days as comparator	0.0	100.0	100.0	100.0	0.0	100.0	100.0	100.0
Per−month PS matching, using outpatient visits as comparator								
Per−month PS matching, using random days as comparator								
Historical comparator								
Unadjusted, using entire historic period	2.2	96.8	96.8	94.6	0.0	100.0	100.0	100.0
Age & sex adjusted, using entire historic period	1.1	96.8	95.7	93.5	0.0	100.0	98.9	96.8
Unadjusted, using TaR after historic visit	1.1	97.8	96.8	94.6	0.0	100.0	100.0	100.0
Age & sex adjusted, using TaR after historic visit	1.1	97.8	96.8	91.4	0.0	100.0	98.9	96.8
Unadjusted, using entire historic period, filtered	2.2	96.8	96.8	94.6	0.0	100.0	100.0	100.0
Age & sex adjusted, using entire historic period, filtered	1.1	96.8	95.7	93.5	0.0	100.0	98.9	96.8
Unadjusted, using TaR after historic visit, filtered	1.1	97.8	96.8	94.6	0.0	100.0	100.0	98.9
Age & sex adjusted, using TaR after historic visit, filtered	1.1	97.8	96.8	91.4	0.0	100.0	97.8	96.8
SCCS / SCRI								
Unadjusted SCCS excluding pre−vaccination window	2.2	95.7	94.6	89.2	2.2	98.9	95.7	92.5
Age & season adjusted SCCS excluding pre−vaccination window	2.2	96.8	95.7	89.2	2.2	97.8	96.8	92.5
SCRI with prior control interval	1.1	100.0	98.9	98.9	1.1	100.0	98.9	97.8
SCRI with posterior control interval	0.0	100.0	98.9	97.8	0.0	100.0	98.9	97.8
Unadjusted SCCS excluding all pre−vaccination time	2.2	95.7	94.6	90.3	1.1	98.9	95.7	90.3

Figure 51: Type 1 and 2 error based on whether the log likelihood ratio exceeded the critical value any time during the study period in the MDCD database for Seasonal flu vaccination (Fluzone).

	Uncalibrated				Calibrated			
		Type 2 when true effect is				Type 2 when true effect is		
	Type 1	1.5	2	4	Type 1	1.5	2	4
Case−control								
Age & sex matched controls	2.2	91.4	84.9	72.0	3.2	91.4	82.8	69.9
Age & sex adjusted, using random controls	4.3	87.1	83.9	66.7	4.3	91.4	83.9	66.7
Cohort method								
Unadjusted, using outpatient visits as comparator	0.0	93.5	92.5	89.2	2.2	94.6	91.4	84.9
PS matching, using outpatient visits as comparator	0.0	98.9	97.8	84.9	1.1	97.8	90.3	82.8
Unadjusted, using random days as comparator	1.1	96.8	92.5	84.9	1.1	95.7	91.4	83.9
PS matching, using random days as comparator	1.1	97.8	93.5	86.0	1.1	95.7	90.3	82.8
PS stratification, using outpatient visits as comparator	0.0	93.5	89.2	80.6	2.2	91.4	83.9	68.8
PS stratification, using random days as comparator	1.1	96.8	91.4	77.4	1.1	94.6	90.3	73.1
PS weighting, using outpatient visits as comparator	1.1	95.7	95.7	81.7	2.2	90.3	83.9	76.3
PS weighting, using random days as comparator	0.0	97.8	96.8	91.4	1.1	97.8	96.8	91.4
Per−month PS matching, using outpatient visits as comparator								
Per−month PS matching, using random days as comparator								
Historical comparator								
Unadjusted, using entire historic period	4.3	86.0	83.9	65.6	3.2	96.8	95.7	83.9
Age & sex adjusted, using entire historic period	4.3	86.0	82.8	66.7	3.2	96.8	95.7	80.6
Unadjusted, using TaR after historic visit	3.2	87.1	82.8	73.1	3.2	95.7	93.5	81.7
Age & sex adjusted, using TaR after historic visit	3.2	88.2	83.9	71.0	3.2	96.8	93.5	79.6
Unadjusted, using entire historic period, filtered	3.2	87.1	84.9	66.7	3.2	91.4	87.1	69.9
Age & sex adjusted, using entire historic period, filtered	3.2	87.1	83.9	67.7	3.2	89.2	84.9	69.9
Unadjusted, using TaR after historic visit, filtered	2.2	88.2	83.9	74.2	5.4	92.5	87.1	74.2
Age & sex adjusted, using TaR after historic visit, filtered	2.2	89.2	84.9	72.0	5.4	92.5	84.9	72.0
SCCS / SCRI								
Unadjusted SCCS excluding pre−vaccination window	2.2	91.4	83.9	72.0	2.2	90.3	83.9	71.0
Age & season adjusted SCCS excluding pre−vaccination window	2.2	90.3	82.8	69.9	2.2	89.2	82.8	69.9
SCRI with prior control interval	4.3	95.7	93.5	87.1	1.1	96.8	93.5	87.1
SCRI with posterior control interval	3.2	93.5	92.5	86.0	1.1	93.5	92.5	86.0
Unadjusted SCCS excluding all pre−vaccination time	3.2	90.3	84.9	77.4	2.2	90.3	83.9	76.3

Figure 52: Type 1 and 2 error based on whether the log likelihood ratio exceeded the critical value any time during the study period in the MDCR database for Seasonal flu vaccination (Fluzone).

	Uncalibrated				Calibrated			
		Type 2 when true effect is				Type 2 when true effect is		
	Type 1	1.5	2	4	Type 1	1.5	2	4
Case−control								
Age & sex matched controls	0.0	100.0	100.0	100.0	0.0	100.0	100.0	100.0
Age & sex adjusted, using random controls	0.0	100.0	100.0	100.0	0.0	100.0	100.0	100.0
Cohort method								
Unadjusted, using outpatient visits as comparator	0.0	100.0	100.0	100.0	0.0	100.0	100.0	100.0
PS matching, using outpatient visits as comparator	0.0	100.0	100.0	100.0	0.0	100.0	100.0	100.0
Unadjusted, using random days as comparator	0.0	100.0	100.0	100.0	0.0	100.0	100.0	100.0
PS matching, using random days as comparator	0.0	100.0	100.0	100.0	0.0	100.0	100.0	100.0
PS stratification, using outpatient visits as comparator	0.0	100.0	100.0	98.9	0.0	100.0	100.0	100.0
PS stratification, using random days as comparator	0.0	98.9	98.9	98.9	0.0	100.0	100.0	100.0
PS weighting, using outpatient visits as comparator	0.0	100.0	100.0	100.0	0.0	100.0	100.0	100.0
PS weighting, using random days as comparator	0.0	100.0	100.0	100.0	0.0	100.0	100.0	100.0
Per−month PS matching, using outpatient visits as comparator								
Per−month PS matching, using random days as comparator								
Historical comparator								
Unadjusted, using entire historic period	0.0	100.0	100.0	100.0	0.0	100.0	100.0	100.0
Age & sex adjusted, using entire historic period	0.0	100.0	100.0	100.0	0.0	100.0	100.0	100.0
Unadjusted, using TaR after historic visit	0.0	100.0	100.0	100.0	0.0	100.0	100.0	100.0
Age & sex adjusted, using TaR after historic visit	0.0	100.0	100.0	100.0	0.0	100.0	100.0	100.0
Unadjusted, using entire historic period, filtered	0.0	100.0	100.0	100.0	0.0	100.0	100.0	100.0
Age & sex adjusted, using entire historic period, filtered	0.0	100.0	100.0	100.0	0.0	100.0	100.0	100.0
Unadjusted, using TaR after historic visit, filtered	0.0	100.0	100.0	100.0	0.0	100.0	100.0	100.0
Age & sex adjusted, using TaR after historic visit, filtered	0.0	100.0	100.0	100.0	0.0	100.0	100.0	100.0
SCCS / SCRI								
Unadjusted SCCS excluding pre−vaccination window	0.0	100.0	100.0	100.0	0.0	100.0	100.0	100.0
Age & season adjusted SCCS excluding pre−vaccination window	0.0	100.0	100.0	100.0	0.0	100.0	100.0	100.0
SCRI with prior control interval								
SCRI with posterior control interval								
Unadjusted SCCS excluding all pre−vaccination time	0.0	100.0	100.0	100.0	0.0	100.0	100.0	100.0

Figure 53: Type 1 and 2 error based on whether the log likelihood ratio exceeded the critical value any time during the study period in the CCAE database for Seasonal flu vaccination (Fluzone).

11.4 Type 1 and 2 error for Seasonal flu vaccination (All)

	Uncalibrated				Calibrated			
		Type 2 when true effect is				Type 2 when true effect is		
	Type 1	1.5	2	4	Type 1	1.5	2	4
Case−control								
Age & sex matched controls	78.5	15.1	11.8	7.5	5.4	60.2	31.2	15.1
Age & sex adjusted, using random controls	81.7	14.0	8.6	5.4	7.5	58.1	26.9	11.8
Cohort method								
Unadjusted, using outpatient visits as comparator	2.2	59.1	50.5	22.6	4.3	80.6	53.8	16.1
PS matching, using outpatient visits as comparator	1.1	65.6	47.3	12.9	4.3	61.3	34.4	10.8
Unadjusted, using random days as comparator	67.7	17.2	14.0	7.5	5.4	84.9	58.1	15.1
PS matching, using random days as comparator								
PS stratification, using outpatient visits as comparator	1.1	64.5	48.4	11.8	4.3	67.7	43.0	8.6
PS stratification, using random days as comparator	79.6	11.8	5.4	4.3	1.1	78.5	45.2	9.7
PS weighting, using outpatient visits as comparator	3.2	60.2	41.9	15.1	6.5	64.5	34.4	12.9
PS weighting, using random days as comparator	0.0	100.0	100.0	100.0	0.0	100.0	100.0	100.0
Per−month PS matching, using outpatient visits as comparator								
Per−month PS matching, using random days as comparator								
Historical comparator								
Unadjusted, using entire historic period	91.4	3.2	2.2	1.1	1.1	78.5	58.1	8.6
Age & sex adjusted, using entire historic period	92.5	3.2	3.2	1.1	3.2	80.6	43.0	5.4
Unadjusted, using TaR after historic visit	59.1	18.3	11.8	3.2	3.2	89.2	55.9	11.8
Age & sex adjusted, using TaR after historic visit	60.2	18.3	7.5	3.2	4.3	86.0	48.4	6.5
Unadjusted, using entire historic period, filtered	90.3	3.2	2.2	1.1	2.2	71.0	37.6	3.2
Age & sex adjusted, using entire historic period, filtered	91.4	3.2	3.2	1.1	3.2	49.5	23.7	3.2
Unadjusted, using TaR after historic visit, filtered	58.1	20.4	12.9	3.2	3.2	69.9	43.0	5.4
Age & sex adjusted, using TaR after historic visit, filtered	59.1	20.4	8.6	3.2	5.4	52.7	25.8	3.2
SCCS / SCRI								
Unadjusted SCCS excluding pre−vaccination window	34.4	7.5	7.5	4.3	4.3	62.4	24.7	5.4
Age & season adjusted SCCS excluding pre−vaccination window	45.2	9.7	10.8	2.2	3.2	66.7	35.5	5.4
SCRI with prior control interval	53.8	19.4	11.8	7.5	6.5	88.2	47.3	10.8
SCRI with posterior control interval	43.0	21.5	14.0	4.3	6.5	72.0	35.5	6.5
Unadjusted SCCS excluding all pre−vaccination time	49.5	11.8	6.5	3.2	6.5	61.3	24.7	4.3

Figure 54: Type 1 and 2 error based on whether the log likelihood ratio exceeded the critical value any time during the study period in the Optum EHR database for Seasonal flu vaccination (All).

	Uncalibrated				Calibrated			
		Type 2 when true effect is				Type 2 when true effect is		
	Type 1	1.5	2	4	Type 1	1.5	2	4
Case−control								
Age & sex matched controls	54.8	30.1	23.7	21.5	5.4	71.0	49.5	23.7
Age & sex adjusted, using random controls	63.4	25.8	19.4	15.1	6.5	66.7	39.8	18.3
Cohort method								
Unadjusted, using outpatient visits as comparator	7.5	53.8	65.6	35.5	8.6	82.8	73.1	30.1
PS matching, using outpatient visits as comparator	3.2	75.3	53.8	23.7	8.6	67.7	41.9	23.7
Unadjusted, using random days as comparator	47.3	31.2	24.7	19.4	8.6	83.9	63.4	24.7
PS matching, using random days as comparator	3.2	68.8	53.8	29.0	4.3	66.7	50.5	25.8
PS stratification, using outpatient visits as comparator	3.2	66.7	43.0	22.6	7.5	59.1	40.9	21.5
PS stratification, using random days as comparator	8.6	61.3	48.4	23.7	3.2	72.0	52.7	24.7
PS weighting, using outpatient visits as comparator	4.3	62.4	48.4	20.4	3.2	71.0	49.5	18.3
PS weighting, using random days as comparator	8.6	69.9	67.7	34.4	8.6	75.3	55.9	31.2
Per−month PS matching, using outpatient visits as comparator								
Per−month PS matching, using random days as comparator								
Historical comparator								
Unadjusted, using entire historic period	53.8	18.3	17.2	12.9	4.3	93.5	83.9	23.7
Age & sex adjusted, using entire historic period	60.2	18.3	17.2	12.9	3.2	90.3	78.5	22.6
Unadjusted, using TaR after historic visit	28.0	45.2	30.1	17.2	4.3	91.4	83.9	31.2
Age & sex adjusted, using TaR after historic visit	22.6	44.1	31.2	17.2	4.3	89.2	82.8	29.0
Unadjusted, using entire historic period, filtered	52.7	19.4	18.3	14.0	4.3	82.8	55.9	18.3
Age & sex adjusted, using entire historic period, filtered	59.1	19.4	18.3	14.0	3.2	79.6	45.2	18.3
Unadjusted, using TaR after historic visit, filtered	26.9	46.2	31.2	18.3	6.5	81.7	60.2	22.6
Age & sex adjusted, using TaR after historic visit, filtered	21.5	45.2	32.3	18.3	7.5	81.7	51.6	20.4
SCCS / SCRI								
Unadjusted SCCS excluding pre−vaccination window	7.5	41.9	26.9	19.4	6.5	58.1	29.0	19.4
Age & season adjusted SCCS excluding pre−vaccination window	6.5	36.6	25.8	18.3	3.2	51.6	30.1	18.3
SCRI with prior control interval	12.9	55.9	41.9	22.6	4.3	87.1	63.4	26.9
SCRI with posterior control interval	20.4	53.8	34.4	19.4	3.2	78.5	51.6	21.5
Unadjusted SCCS excluding all pre−vaccination time	19.4	47.3	25.8	18.3	3.2	72.0	45.2	19.4

Figure 55: Type 1 and 2 error based on whether the log likelihood ratio exceeded the critical value any time during the study period in the MDCD database for Seasonal flu vaccination (All).

	Uncalibrated				Calibrated			
		Type 2 when true effect is				Type 2 when true effect is		
	Type 1	1.5	2	4	Type 1	1.5	2	4
Case−control								
Age & sex matched controls	22.6	44.1	31.2	24.7	6.5	59.1	34.4	24.7
Age & sex adjusted, using random controls	24.7	39.8	26.9	19.4	11.8	59.1	32.3	21.5
Cohort method								
Unadjusted, using outpatient visits as comparator	1.1	79.6	76.3	53.8	3.2	86.0	72.0	40.9
PS matching, using outpatient visits as comparator	2.2	84.9	67.7	44.1	7.5	78.5	59.1	37.6
Unadjusted, using random days as comparator	12.9	71.0	51.6	32.3	5.4	82.8	59.1	38.7
PS matching, using random days as comparator	2.2	78.5	65.6	39.8	6.5	75.3	60.2	39.8
PS stratification, using outpatient visits as comparator	5.4	82.8	66.7	39.8	7.5	73.1	55.9	35.5
PS stratification, using random days as comparator	7.5	76.3	62.4	34.4	6.5	80.6	62.4	35.5
PS weighting, using outpatient visits as comparator	1.1	80.6	68.8	48.4	6.5	79.6	61.3	41.9
PS weighting, using random days as comparator	3.2	90.3	80.6	48.4	3.2	88.2	74.2	44.1
Per−month PS matching, using outpatient visits as comparator								
Per−month PS matching, using random days as comparator								
Historical comparator								
Unadjusted, using entire historic period	30.1	36.6	26.9	19.4	7.5	84.9	72.0	23.7
Age & sex adjusted, using entire historic period	29.0	36.6	26.9	20.4	6.5	86.0	72.0	24.7
Unadjusted, using TaR after historic visit	14.0	59.1	37.6	22.6	8.6	87.1	75.3	29.0
Age & sex adjusted, using TaR after historic visit	15.1	57.0	34.4	20.4	8.6	84.9	73.1	28.0
Unadjusted, using entire historic period, filtered	26.9	38.7	29.0	20.4	8.6	58.1	34.4	22.6
Age & sex adjusted, using entire historic period, filtered	25.8	38.7	29.0	21.5	7.5	59.1	36.6	23.7
Unadjusted, using TaR after historic visit, filtered	11.8	61.3	39.8	24.7	7.5	67.7	48.4	25.8
Age & sex adjusted, using TaR after historic visit, filtered	12.9	59.1	36.6	22.6	8.6	66.7	43.0	23.7
SCCS / SCRI								
Unadjusted SCCS excluding pre−vaccination window	7.5	50.5	40.9	24.7	5.4	62.4	46.2	23.7
Age & season adjusted SCCS excluding pre−vaccination window	6.5	53.8	44.1	32.3	3.2	62.4	47.3	32.3
SCRI with prior control interval	17.2	64.5	51.6	36.6	1.1	89.2	73.1	41.9
SCRI with posterior control interval	11.8	75.3	55.9	32.3	4.3	80.6	64.5	33.3
Unadjusted SCCS excluding all pre−vaccination time	17.2	57.0	41.9	24.7	5.4	74.2	53.8	28.0

Figure 56: Type 1 and 2 error based on whether the log likelihood ratio exceeded the critical value any time during the study period in the MDCR database for Seasonal flu vaccination (All).

	Uncalibrated				Calibrated			
		Type 2 when true effect is				Type 2 when true effect is		
	Type 1	1.5	2	4	Type 1	1.5	2	4
Case−control								
Age & sex matched controls	41.9	36.6	25.8	18.3	5.4	52.7	38.7	21.5
Age & sex adjusted, using random controls	45.2	32.3	22.6	16.1	9.7	52.7	35.5	19.4
Cohort method								
Unadjusted, using outpatient visits as comparator	1.1	34.4	49.5	49.5	4.3	87.1	80.6	41.9
PS matching, using outpatient visits as comparator	2.2	74.2	53.8	25.8	9.7	62.4	43.0	23.7
Unadjusted, using random days as comparator	33.3	47.3	30.1	17.2	3.2	75.3	58.1	22.6
PS matching, using random days as comparator	10.8	60.2	43.0	26.9	7.5	63.4	45.2	26.9
PS stratification, using outpatient visits as comparator	2.2	73.1	54.8	25.8	7.5	68.8	44.1	21.5
PS stratification, using random days as comparator	12.9	60.2	43.0	23.7	5.4	65.6	47.3	23.7
PS weighting, using outpatient visits as comparator	2.2	58.1	61.3	32.3	6.5	75.3	53.8	28.0
PS weighting, using random days as comparator	1.1	75.3	59.1	37.6	2.2	74.2	58.1	36.6
Per−month PS matching, using outpatient visits as comparator								
Per−month PS matching, using random days as comparator								
Historical comparator								
Unadjusted, using entire historic period	52.7	22.6	18.3	12.9	5.4	89.2	72.0	22.6
Age & sex adjusted, using entire historic period	48.4	20.4	19.4	11.8	5.4	88.2	72.0	19.4
Unadjusted, using TaR after historic visit	21.5	43.0	29.0	16.1	5.4	92.5	80.6	34.4
Age & sex adjusted, using TaR after historic visit	18.3	47.3	24.7	16.1	5.4	91.4	80.6	26.9
Unadjusted, using entire historic period, filtered	50.5	23.7	18.3	12.9	3.2	65.6	32.3	17.2
Age & sex adjusted, using entire historic period, filtered	46.2	21.5	19.4	11.8	4.3	52.7	23.7	14.0
Unadjusted, using TaR after historic visit, filtered	20.4	46.2	29.0	16.1	8.6	79.6	52.7	16.1
Age & sex adjusted, using TaR after historic visit, filtered	17.2	50.5	24.7	16.1	7.5	73.1	46.2	16.1
SCCS / SCRI								
Unadjusted SCCS excluding pre−vaccination window	20.4	24.7	16.1	15.1	6.5	47.3	21.5	15.1
Age & season adjusted SCCS excluding pre−vaccination window	19.4	24.7	17.2	15.1	2.2	46.2	21.5	15.1
SCRI with prior control interval	30.1	36.6	30.1	16.1	2.2	88.2	60.2	19.4
SCRI with posterior control interval	25.8	37.6	24.7	15.1	5.4	78.5	38.7	15.1
Unadjusted SCCS excluding all pre−vaccination time	30.1	24.7	19.4	14.0	5.4	64.5	28.0	15.1

Figure 57: Type 1 and 2 error based on whether the log likelihood ratio exceeded the critical value any time during the study period in the CCAE database for Seasonal flu vaccination (All).

11.5 Type 1 and 2 error for Zoster vaccination (Shingrix)

	Uncalibrated				Calibrated			
		Type 2 when true effect is				Type 2 when true effect is		
	Type 1	1.5	2	4	Type 1	1.5	2	4
Case−control								
Age & sex matched controls	30.1	65.6	62.4	48.4	8.6	88.2	75.3	63.4
Age & sex adjusted, using random controls	24.7	63.4	55.9	46.2	10.8	82.8	76.3	57.0
Cohort method								
Unadjusted, using outpatient visits as comparator	3.2	74.2	68.8	55.9	5.4	93.5	79.6	55.9
PS matching, using outpatient visits as comparator	1.1	86.0	82.8	52.7	6.5	80.6	67.7	43.0
Unadjusted, using random days as comparator	34.4	58.1	48.4	39.8	3.2	96.8	89.2	62.4
PS matching, using random days as comparator								
PS stratification, using outpatient visits as comparator	1.1	81.7	78.5	51.6	6.5	86.0	69.9	35.5
PS stratification, using random days as comparator								
PS weighting, using outpatient visits as comparator	1.1	90.3	81.7	62.4	3.2	84.9	74.2	53.8
PS weighting, using random days as comparator								
Per−month PS matching, using outpatient visits as comparator								
Per−month PS matching, using random days as comparator								
Historical comparator								
Unadjusted, using entire historic period	57.0	32.3	32.3	29.0	3.2	89.2	83.9	51.6
Age & sex adjusted, using entire historic period	46.2	39.8	31.2	24.7	2.2	90.3	76.3	47.3
Unadjusted, using TaR after historic visit	36.6	40.9	39.8	29.0	2.2	91.4	77.4	50.5
Age & sex adjusted, using TaR after historic visit	24.7	53.8	48.4	28.0	6.5	87.1	79.6	47.3
Unadjusted, using entire historic period, filtered	55.9	33.3	33.3	30.1	3.2	88.2	83.9	49.5
Age & sex adjusted, using entire historic period, filtered	45.2	40.9	32.3	25.8	2.2	87.1	75.3	46.2
Unadjusted, using TaR after historic visit, filtered	35.5	41.9	40.9	30.1	2.2	89.2	77.4	49.5
Age & sex adjusted, using TaR after historic visit, filtered	23.7	54.8	49.5	29.0	6.5	83.9	74.2	44.1
SCCS / SCRI								
Unadjusted SCCS excluding pre−vaccination window	12.9	63.4	47.3	19.4	4.3	83.9	59.1	23.7
Age & season adjusted SCCS excluding pre−vaccination window	11.8	64.5	46.2	19.4	8.6	77.4	54.8	20.4
SCRI with prior control interval	21.5	71.0	57.0	32.3	10.8	86.0	71.0	40.9
SCRI with posterior control interval	16.1	76.3	62.4	39.8	2.2	89.2	74.2	44.1
Unadjusted SCCS excluding all pre−vaccination time	21.5	61.3	46.2	26.9	6.5	78.5	62.4	31.2

Figure 58: Type 1 and 2 error based on whether the log likelihood ratio exceeded the critical value any time during the study period in the Optum EHR database for Zoster vaccination (Shingrix).

	Uncalibrated				Calibrated			
		Type 2 when true effect is				Type 2 when true effect is		
	Type 1	1.5	2	4	Type 1	1.5	2	4
Case−control								
Age & sex matched controls	9.7	88.2	83.9	69.9	2.2	95.7	93.5	80.6
Age & sex adjusted, using random controls	6.5	86.0	79.6	69.9	2.2	95.7	91.4	76.3
Cohort method								
Unadjusted, using outpatient visits as comparator	0.0	96.8	91.4	82.8	0.0	97.8	92.5	84.9
PS matching, using outpatient visits as comparator	0.0	100.0	97.8	91.4	0.0	98.9	96.8	86.0
Unadjusted, using random days as comparator	8.6	90.3	90.3	84.9	2.2	97.8	96.8	90.3
PS matching, using random days as comparator	0.0	100.0	95.7	89.2	0.0	100.0	96.8	88.2
PS stratification, using outpatient visits as comparator	2.2	96.8	92.5	77.4	2.2	94.6	92.5	74.2
PS stratification, using random days as comparator	3.2	92.5	89.2	73.1	0.0	96.8	93.5	74.2
PS weighting, using outpatient visits as comparator	0.0	100.0	100.0	96.8	0.0	100.0	100.0	95.7
PS weighting, using random days as comparator	0.0	100.0	97.8	93.5	0.0	100.0	98.9	93.5
Per−month PS matching, using outpatient visits as comparator								
Per−month PS matching, using random days as comparator								
Historical comparator								
Unadjusted, using entire historic period	15.1	80.6	79.6	77.4	0.0	97.8	97.8	90.3
Age & sex adjusted, using entire historic period	12.9	72.0	68.8	66.7	1.1	97.8	94.6	74.2
Unadjusted, using TaR after historic visit	16.1	79.6	75.3	71.0	1.1	95.7	95.7	87.1
Age & sex adjusted, using TaR after historic visit	5.4	81.7	74.2	67.7	1.1	95.7	88.2	72.0
Unadjusted, using entire historic period, filtered	15.1	80.6	79.6	77.4	1.1	97.8	93.5	88.2
Age & sex adjusted, using entire historic period, filtered	10.8	73.1	69.9	67.7	1.1	91.4	89.2	71.0
Unadjusted, using TaR after historic visit, filtered	15.1	80.6	76.3	72.0	3.2	95.7	93.5	84.9
Age & sex adjusted, using TaR after historic visit, filtered	3.2	83.9	76.3	68.8	2.2	91.4	82.8	72.0
SCCS / SCRI								
Unadjusted SCCS excluding pre−vaccination window	2.2	92.5	83.9	62.4	2.2	93.5	84.9	62.4
Age & season adjusted SCCS excluding pre−vaccination window	4.3	86.0	76.3	62.4	3.2	90.3	80.6	64.5
SCRI with prior control interval	5.4	98.9	94.6	80.6	1.1	98.9	92.5	79.6
SCRI with posterior control interval	0.0	98.9	92.5	84.9	1.1	98.9	91.4	84.9
Unadjusted SCCS excluding all pre−vaccination time	6.5	92.5	87.1	67.7	3.2	96.8	89.2	72.0

Figure 59: Type 1 and 2 error based on whether the log likelihood ratio exceeded the critical value any time during the study period in the MDCD database for Zoster vaccination (Shingrix).

	Uncalibrated				Calibrated			
		Type 2 when true effect is				Type 2 when true effect is		
	Type 1	1.5	2	4	Type 1	1.5	2	4
Case−control								
Age & sex matched controls	15.1	77.4	68.8	57.0	5.4	84.9	78.5	59.1
Age & sex adjusted, using random controls	14.0	74.2	69.9	50.5	8.6	80.6	75.3	57.0
Cohort method								
Unadjusted, using outpatient visits as comparator	1.1	84.9	86.0	77.4	2.2	95.7	91.4	77.4
PS matching, using outpatient visits as comparator	2.2	93.5	86.0	72.0	4.3	89.2	82.8	69.9
Unadjusted, using random days as comparator	2.2	84.9	81.7	66.7	1.1	91.4	84.9	71.0
PS matching, using random days as comparator	2.2	90.3	87.1	67.7	2.2	88.2	86.0	67.7
PS stratification, using outpatient visits as comparator	3.2	90.3	81.7	65.6	3.2	88.2	81.7	64.5
PS stratification, using random days as comparator	4.3	86.0	78.5	55.9	4.3	89.2	79.6	58.1
PS weighting, using outpatient visits as comparator	1.1	88.2	83.9	67.7	2.2	92.5	84.9	66.7
PS weighting, using random days as comparator	0.0	98.9	94.6	86.0	0.0	98.9	94.6	86.0
Per−month PS matching, using outpatient visits as comparator								
Per−month PS matching, using random days as comparator								
Historical comparator								
Unadjusted, using entire historic period	15.1	72.0	68.8	52.7	4.3	91.4	87.1	71.0
Age & sex adjusted, using entire historic period	12.9	71.0	66.7	51.6	5.4	92.5	86.0	67.7
Unadjusted, using TaR after historic visit	12.9	73.1	67.7	57.0	5.4	92.5	89.2	73.1
Age & sex adjusted, using TaR after historic visit	9.7	74.2	69.9	58.1	4.3	93.5	88.2	71.0
Unadjusted, using entire historic period, filtered	14.0	73.1	69.9	53.8	5.4	88.2	81.7	66.7
Age & sex adjusted, using entire historic period, filtered	10.8	73.1	68.8	53.8	4.3	88.2	80.6	64.5
Unadjusted, using TaR after historic visit, filtered	9.7	76.3	69.9	59.1	3.2	91.4	83.9	68.8
Age & sex adjusted, using TaR after historic visit, filtered	8.6	76.3	71.0	59.1	3.2	90.3	83.9	68.8
SCCS / SCRI								
Unadjusted SCCS excluding pre−vaccination window	14.0	75.3	66.7	48.4	6.5	82.8	71.0	51.6
Age & season adjusted SCCS excluding pre−vaccination window	10.8	74.2	65.6	48.4	9.7	81.7	69.9	48.4
SCRI with prior control interval	7.5	84.9	75.3	59.1	2.2	89.2	80.6	62.4
SCRI with posterior control interval	8.6	88.2	83.9	65.6	4.3	89.2	83.9	66.7
Unadjusted SCCS excluding all pre−vaccination time	8.6	81.7	75.3	52.7	3.2	82.8	77.4	55.9

Figure 60: Type 1 and 2 error based on whether the log likelihood ratio exceeded the critical value any time during the study period in the MDCR database for Zoster vaccination (Shingrix).

	Uncalibrated Type 1	Uncalibrated Type 2 when true effect is 1.5	2	4	Calibrated Type 1	Calibrated Type 2 when true effect is 1.5	2	4
Case−control								
Age & sex matched controls	8.6	79.6	75.3	62.4	3.2	84.9	77.4	65.6
Age & sex adjusted, using random controls	9.7	80.6	73.1	58.1	4.3	92.5	80.6	60.2
Cohort method								
Unadjusted, using outpatient visits as comparator	2.2	86.0	82.8	69.9	2.2	97.8	90.3	61.3
PS matching, using outpatient visits as comparator	0.0	94.6	82.8	64.5	2.2	84.9	71.0	54.8
Unadjusted, using random days as comparator	18.3	74.2	67.7	55.9	2.2	98.9	93.5	76.3
PS matching, using random days as comparator	1.1	88.2	80.6	54.8	2.2	88.2	79.6	54.8
PS stratification, using outpatient visits as comparator	0.0	90.3	84.9	55.9	4.3	86.0	74.2	48.4
PS stratification, using random days as comparator	4.3	79.6	71.0	44.1	3.2	84.9	71.0	46.2
PS weighting, using outpatient visits as comparator	0.0	93.5	87.1	75.3	3.2	88.2	84.9	74.2
PS weighting, using random days as comparator	0.0	96.8	93.5	82.8	0.0	95.7	93.5	82.8
Per−month PS matching, using outpatient visits as comparator								
Per−month PS matching, using random days as comparator								
Historical comparator								
Unadjusted, using entire historic period	35.5	50.5	44.1	38.7	5.4	89.2	82.8	57.0
Age & sex adjusted, using entire historic period	18.3	55.9	48.4	28.0	5.4	88.2	74.2	40.9
Unadjusted, using TaR after historic visit	24.7	57.0	48.4	36.6	3.2	88.2	80.6	51.6
Age & sex adjusted, using TaR after historic visit	9.7	68.8	60.2	44.1	5.4	88.2	79.6	48.4
Unadjusted, using entire historic period, filtered	33.3	52.7	46.2	40.9	4.3	86.0	76.3	52.7
Age & sex adjusted, using entire historic period, filtered	15.1	58.1	50.5	30.1	5.4	68.8	55.9	33.3
Unadjusted, using TaR after historic visit, filtered	22.6	59.1	50.5	38.7	3.2	87.1	73.1	50.5
Age & sex adjusted, using TaR after historic visit, filtered	6.5	71.0	62.4	46.2	5.4	80.6	64.5	45.2
SCCS / SCRI								
Unadjusted SCCS excluding pre−vaccination window	8.6	67.7	58.1	32.3	5.4	77.4	60.2	36.6
Age & season adjusted SCCS excluding pre−vaccination window	12.9	65.6	53.8	24.7	6.5	77.4	58.1	26.9
SCRI with prior control interval	7.5	84.9	75.3	47.3	3.2	89.2	77.4	50.5
SCRI with posterior control interval	6.5	87.1	77.4	53.8	3.2	90.3	80.6	57.0
Unadjusted SCCS excluding all pre−vaccination time	10.8	76.3	62.4	43.0	3.2	83.9	69.9	44.1

Figure 61: Type 1 and 2 error based on whether the log likelihood ratio exceeded the critical value any time during the study period in the CCAE database for Zoster vaccination (Shingrix).

11.6 Type 1 and 2 error for HPV vaccination (Gardasil 9)

	Uncalibrated				Calibrated			
		Type 2 when true effect is				Type 2 when true effect is		
	Type 1	1.5	2	4	Type 1	1.5	2	4
Case−control								
Age & sex matched controls	19.4	74.2	66.7	60.2	2.2	92.5	89.2	78.5
Age & sex adjusted, using random controls	8.6	81.7	79.6	73.1	5.4	93.5	89.2	78.5
Cohort method								
Unadjusted, using outpatient visits as comparator	1.1	64.5	67.7	76.3	3.2	96.8	93.5	87.1
PS matching, using outpatient visits as comparator	1.1	95.7	91.4	77.4	4.3	94.6	88.2	71.0
Unadjusted, using random days as comparator	4.3	83.9	79.6	74.2	3.2	96.8	94.6	87.1
PS matching, using random days as comparator								
PS stratification, using outpatient visits as comparator	1.1	93.5	89.2	72.0	3.2	90.3	82.8	63.4
PS stratification, using random days as comparator								
PS weighting, using outpatient visits as comparator	0.0	97.8	96.8	91.4	0.0	97.8	95.7	88.2
PS weighting, using random days as comparator								
Per−month PS matching, using outpatient visits as comparator								
Per−month PS matching, using random days as comparator								
Historical comparator								
Unadjusted, using entire historic period	12.9	72.0	68.8	63.4	1.1	94.6	89.2	84.9
Age & sex adjusted, using entire historic period	18.3	64.5	62.4	59.1	3.2	95.7	91.4	74.2
Unadjusted, using TaR after historic visit	7.5	69.9	73.1	67.7	2.2	95.7	92.5	83.9
Age & sex adjusted, using TaR after historic visit	7.5	79.6	71.0	54.8	2.2	92.5	84.9	61.3
Unadjusted, using entire historic period, filtered	11.8	73.1	69.9	64.5	1.1	94.6	90.3	86.0
Age & sex adjusted, using entire historic period, filtered	17.2	65.6	63.4	60.2	2.2	93.5	83.9	64.5
Unadjusted, using TaR after historic visit, filtered	7.5	71.0	74.2	68.8	1.1	96.8	92.5	84.9
Age & sex adjusted, using TaR after historic visit, filtered	6.5	80.6	72.0	55.9	2.2	90.3	77.4	59.1
SCCS / SCRI								
Unadjusted SCCS excluding pre−vaccination window	8.6	68.8	60.2	51.6	4.3	91.4	87.1	57.0
Age & season adjusted SCCS excluding pre−vaccination window	7.5	69.9	61.3	52.7	3.2	91.4	81.7	63.4
SCRI with prior control interval	11.8	90.3	80.6	68.8	2.2	95.7	92.5	72.0
SCRI with posterior control interval	11.8	90.3	78.5	68.8	2.2	96.8	91.4	73.1
Unadjusted SCCS excluding all pre−vaccination time	10.8	75.3	71.0	55.9	5.4	91.4	79.6	60.2

Figure 62: Type 1 and 2 error based on whether the log likelihood ratio exceeded the critical value any time during the study period in the Optum EHR database for HPV vaccination (Gardasil 9).

	Uncalibrated				Calibrated			
		Type 2 when true effect is				Type 2 when true effect is		
	Type 1	1.5	2	4	Type 1	1.5	2	4
Case−control								
Age & sex matched controls	15.1	71.0	65.6	59.1	2.2	89.2	82.8	65.6
Age & sex adjusted, using random controls	14.0	75.3	71.0	64.5	3.2	92.5	80.6	66.7
Cohort method								
Unadjusted, using outpatient visits as comparator	2.2	72.0	73.1	76.3	1.1	98.9	98.9	91.4
PS matching, using outpatient visits as comparator	2.2	93.5	86.0	79.6	1.1	96.8	93.5	80.6
Unadjusted, using random days as comparator	5.4	82.8	78.5	78.5	2.2	97.8	97.8	89.2
PS matching, using random days as comparator	3.2	92.5	91.4	80.6	2.2	96.8	94.6	80.6
PS stratification, using outpatient visits as comparator	3.2	89.2	83.9	76.3	3.2	93.5	89.2	77.4
PS stratification, using random days as comparator	7.5	81.7	75.3	66.7	5.4	91.4	86.0	69.9
PS weighting, using outpatient visits as comparator	1.1	96.8	93.5	86.0	1.1	98.9	95.7	86.0
PS weighting, using random days as comparator	2.2	95.7	93.5	86.0	1.1	98.9	96.8	90.3
Per−month PS matching, using outpatient visits as comparator								
Per−month PS matching, using random days as comparator								
Historical comparator								
Unadjusted, using entire historic period	8.6	71.0	69.9	67.7	2.2	94.6	93.5	83.9
Age & sex adjusted, using entire historic period	10.8	77.4	71.0	65.6	4.3	94.6	94.6	78.5
Unadjusted, using TaR after historic visit	5.4	72.0	68.8	68.8	2.2	97.8	96.8	82.8
Age & sex adjusted, using TaR after historic visit	5.4	78.5	76.3	65.6	4.3	95.7	95.7	79.6
Unadjusted, using entire historic period, filtered	6.5	74.2	73.1	69.9	2.2	96.8	96.8	86.0
Age & sex adjusted, using entire historic period, filtered	9.7	79.6	72.0	66.7	3.2	94.6	92.5	68.8
Unadjusted, using TaR after historic visit, filtered	4.3	74.2	72.0	71.0	2.2	97.8	97.8	86.0
Age & sex adjusted, using TaR after historic visit, filtered	4.3	80.6	78.5	66.7	2.2	96.8	88.2	72.0
SCCS / SCRI								
Unadjusted SCCS excluding pre−vaccination window	4.3	77.4	73.1	62.4	2.2	92.5	76.3	63.4
Age & season adjusted SCCS excluding pre−vaccination window	4.3	74.2	69.9	59.1	3.2	82.8	74.2	60.2
SCRI with prior control interval	12.9	84.9	79.6	73.1	2.2	97.8	94.6	78.5
SCRI with posterior control interval	5.4	91.4	82.8	74.2	1.1	97.8	95.7	81.7
Unadjusted SCCS excluding all pre−vaccination time	7.5	81.7	73.1	63.4	1.1	96.8	94.6	68.8

Figure 63: Type 1 and 2 error based on whether the log likelihood ratio exceeded the critical value any time during the study period in the MDCD database for HPV vaccination (Gardasil 9).

	Uncalibrated				Calibrated			
		Type 2 when true effect is				Type 2 when true effect is		
	Type 1	1.5	2	4	Type 1	1.5	2	4
Case−control								
Age & sex matched controls								
Age & sex adjusted, using random controls								
Cohort method								
Unadjusted, using outpatient visits as comparator	0.0	100.0	100.0	100.0	0.0	100.0	100.0	100.0
PS matching, using outpatient visits as comparator								
Unadjusted, using random days as comparator								
PS matching, using random days as comparator								
PS stratification, using outpatient visits as comparator	0.0	100.0	100.0	100.0	0.0	100.0	100.0	100.0
PS stratification, using random days as comparator								
PS weighting, using outpatient visits as comparator	0.0	100.0	100.0	100.0	0.0	100.0	100.0	100.0
PS weighting, using random days as comparator								
Per−month PS matching, using outpatient visits as comparator								
Per−month PS matching, using random days as comparator								
Historical comparator								
Unadjusted, using entire historic period	0.0	100.0	100.0	100.0	0.0	100.0	100.0	100.0
Age & sex adjusted, using entire historic period	0.0	100.0	100.0	100.0	0.0	100.0	100.0	100.0
Unadjusted, using TaR after historic visit	0.0	100.0	100.0	100.0	0.0	100.0	100.0	100.0
Age & sex adjusted, using TaR after historic visit	0.0	100.0	100.0	100.0	0.0	100.0	100.0	100.0
Unadjusted, using entire historic period, filtered	0.0	100.0	100.0	100.0	0.0	100.0	100.0	100.0
Age & sex adjusted, using entire historic period, filtered	0.0	100.0	100.0	100.0	0.0	100.0	100.0	100.0
Unadjusted, using TaR after historic visit, filtered	0.0	100.0	100.0	100.0	0.0	100.0	100.0	100.0
Age & sex adjusted, using TaR after historic visit, filtered	0.0	100.0	100.0	100.0	0.0	100.0	100.0	100.0

Figure 64: Type 1 and 2 error based on whether the log likelihood ratio exceeded the critical value any time during the study period in the MDCR database for HPV vaccination (Gardasil 9).

	Uncalibrated				Calibrated			
		Type 2 when true effect is				Type 2 when true effect is		
	Type 1	1.5	2	4	Type 1	1.5	2	4
Case−control								
Age & sex matched controls	12.9	73.1	64.5	54.8	4.3	91.4	81.7	62.4
Age & sex adjusted, using random controls	11.8	80.6	73.1	61.3	4.3	92.5	87.1	65.6
Cohort method								
Unadjusted, using outpatient visits as comparator	2.2	66.7	62.4	74.2	2.2	98.9	97.8	91.4
PS matching, using outpatient visits as comparator	1.1	97.8	88.2	71.0	1.1	97.8	87.1	69.9
Unadjusted, using random days as comparator	5.4	81.7	81.7	75.3	2.2	96.8	94.6	91.4
PS matching, using random days as comparator	2.2	93.5	90.3	73.1	2.2	95.7	93.5	73.1
PS stratification, using outpatient visits as comparator	1.1	92.5	86.0	69.9	4.3	93.5	82.8	64.5
PS stratification, using random days as comparator	2.2	90.3	84.9	61.3	2.2	95.7	86.0	64.5
PS weighting, using outpatient visits as comparator	0.0	98.9	97.8	88.2	0.0	97.8	95.7	87.1
PS weighting, using random days as comparator	1.1	97.8	94.6	88.2	1.1	98.9	96.8	88.2
Per−month PS matching, using outpatient visits as comparator								
Per−month PS matching, using random days as comparator								
Historical comparator								
Unadjusted, using entire historic period	9.7	75.3	68.8	63.4	2.2	97.8	95.7	80.6
Age & sex adjusted, using entire historic period	12.9	68.8	60.2	52.7	3.2	94.6	87.1	62.4
Unadjusted, using TaR after historic visit	8.6	68.8	69.9	65.6	2.2	98.9	96.8	88.2
Age & sex adjusted, using TaR after historic visit	9.7	77.4	73.1	52.7	4.3	92.5	88.2	69.9
Unadjusted, using entire historic period, filtered	9.7	77.4	71.0	66.7	2.2	97.8	95.7	82.8
Age & sex adjusted, using entire historic period, filtered	10.8	71.0	62.4	54.8	4.3	90.3	78.5	57.0
Unadjusted, using TaR after historic visit, filtered	8.6	68.8	71.0	67.7	1.1	98.9	96.8	88.2
Age & sex adjusted, using TaR after historic visit, filtered	7.5	79.6	75.3	54.8	4.3	91.4	84.9	60.2
SCCS / SCRI								
Unadjusted SCCS excluding pre−vaccination window	11.8	72.0	60.2	47.3	5.4	86.0	76.3	55.9
Age & season adjusted SCCS excluding pre−vaccination window	14.0	68.8	60.2	48.4	7.5	84.9	73.1	51.6
SCRI with prior control interval	12.9	88.2	80.6	62.4	2.2	95.7	92.5	76.3
SCRI with posterior control interval	4.3	91.4	86.0	65.6	1.1	97.8	91.4	69.9
Unadjusted SCCS excluding all pre−vaccination time	9.7	79.6	64.5	53.8	3.2	93.5	84.9	58.1

Figure 65: Type 1 and 2 error based on whether the log likelihood ratio exceeded the critical value any time during the study period in the CCAE database for HPV vaccination (Gardasil 9).

12 AUC based on effect-size estimate

For each method variation and vaccine group, the area under the receiver operator curve (AUC) stratified by true effect size, based on the effect-size estimate at the end of the study period.

12.1 AUC for H1N1 vaccination

	Uncalibrated AUC when true effect is			Calibrated AUC when true effect is		
	1.5	2	4	1.5	2	4
Case−control						
Age & sex matched controls	55.1	57.2	61.1	59.6	63.7	69.1
Age & sex adjusted, using random controls	57.4	59.9	64.2	60.5	66.5	75.4
Cohort method						
Unadjusted, using outpatient visits as comparator	62.3	68.2	83.1	62.2	69.9	77.8
PS matching, using outpatient visits as comparator	62.8	69.6	82.1	64.7	70.0	81.0
Unadjusted, using random days as comparator	55.9	58.0	61.8	59.9	63.6	73.0
PS matching, using random days as comparator	50.0	50.0	50.0	50.0	50.0	50.0
PS stratification, using outpatient visits as comparator	62.9	71.0	86.5	62.7	70.0	79.1
PS stratification, using random days as comparator	59.3	64.1	72.2	63.5	70.3	78.0
PS weighting, using outpatient visits as comparator	62.3	70.3	86.2	61.0	67.4	78.2
PS weighting, using random days as comparator	50.0	50.1	50.1	50.0	50.0	50.0
Per−month PS matching, using outpatient visits as comparator	61.2	66.6	76.9	60.5	66.7	74.8
Per−month PS matching, using random days as comparator	57.9	59.6	65.6	57.6	60.1	66.1
Historical comparator						
Unadjusted, using entire historic period	60.4	66.8	74.4	65.3	72.9	81.6
Age & sex adjusted, using entire historic period	61.3	67.1	74.2	66.2	73.2	81.7
Unadjusted, using TaR after historic visit	63.0	68.6	77.9	65.3	72.9	82.2
Age & sex adjusted, using TaR after historic visit	62.9	68.5	76.9	65.5	73.1	81.3
Unadjusted, using entire historic period, filtered	59.5	65.5	72.0	64.8	71.8	79.7
Age & sex adjusted, using entire historic period, filtered	60.5	65.8	71.9	65.5	71.5	79.3
Unadjusted, using TaR after historic visit, filtered	62.5	67.6	76.2	65.5	72.6	81.2
Age & sex adjusted, using TaR after historic visit, filtered	62.3	67.3	75.1	64.9	72.3	79.7
SCCS / SCRI						
Unadjusted SCCS excluding pre−vaccination window	65.3	72.4	80.7	65.3	72.4	80.7
Age & season adjusted SCCS excluding pre−vaccination window	64.5	71.6	80.3	64.0	71.7	79.7
SCRI with prior control interval	64.4	70.6	79.3	65.8	71.6	80.9
SCRI with posterior control interval	63.7	69.0	75.2	64.6	70.0	76.9
Unadjusted SCCS excluding all pre−vaccination time	64.4	69.8	76.2	66.1	71.8	78.6

Figure 66: The area under the receiver operator curve (AUC) based on the effect-size estimate at the end of the study period in the Optum EHR database for H1N1 vaccination.

	Uncalibrated AUC when true effect is			Calibrated AUC when true effect is		
	1.5	2	4	1.5	2	4
**Case−control**						
Age & sex matched controls	58.8	62.9	68.4	61.6	67.1	73.4
Age & sex adjusted, using random controls	62.5	67.2	73.8	62.5	71.2	77.7
**Cohort method**						
Unadjusted, using outpatient visits as comparator	60.4	65.7	75.4	58.9	64.4	75.8
PS matching, using outpatient visits as comparator	60.4	67.3	75.0	60.9	68.5	74.3
Unadjusted, using random days as comparator	58.5	63.9	78.3	62.2	67.8	76.4
PS matching, using random days as comparator	61.6	66.6	74.8	61.5	66.2	73.9
PS stratification, using outpatient visits as comparator	61.6	69.0	82.4	62.2	69.7	77.8
PS stratification, using random days as comparator	62.4	69.8	81.4	63.7	70.3	78.2
PS weighting, using outpatient visits as comparator	62.7	68.0	85.4	58.7	65.9	76.4
PS weighting, using random days as comparator	59.3	65.1	77.8	59.4	65.1	74.4
Per−month PS matching, using outpatient visits as comparator	64.9	70.3	76.5	64.2	67.9	73.6
Per−month PS matching, using random days as comparator	61.1	66.9	74.3	62.5	68.3	75.5
**Historical comparator**						
Unadjusted, using entire historic period	58.7	64.9	79.7	59.7	67.4	78.0
Age & sex adjusted, using entire historic period	63.1	70.2	77.1	63.4	71.0	79.2
Unadjusted, using TaR after historic visit	57.4	62.9	76.1	59.6	65.7	76.8
Age & sex adjusted, using TaR after historic visit	63.6	70.3	79.6	64.3	71.0	80.4
Unadjusted, using entire historic period, filtered	58.7	65.0	79.9	59.8	67.6	78.1
Age & sex adjusted, using entire historic period, filtered	63.2	70.5	77.3	63.2	70.5	78.8
Unadjusted, using TaR after historic visit, filtered	57.4	62.9	76.2	59.3	66.2	76.6
Age & sex adjusted, using TaR after historic visit, filtered	63.8	70.5	80.0	63.8	70.5	80.0
**SCCS / SCRI**						
Unadjusted SCCS excluding pre−vaccination window	66.2	74.2	80.7	65.8	73.0	79.2
Age & season adjusted SCCS excluding pre−vaccination window	66.7	73.9	79.2	67.1	73.5	78.4
SCRI with prior control interval	62.8	68.4	75.1	63.4	68.1	74.9
SCRI with posterior control interval	61.3	70.0	77.9	61.5	70.2	78.3
Unadjusted SCCS excluding all pre−vaccination time	65.9	73.3	81.3	65.1	73.3	80.5

Figure 67: The area under the receiver operator curve (AUC) based on the effect-size estimate at the end of the study period in the MDCD database for H1N1 vaccination.

	Uncalibrated AUC when true effect is			Calibrated AUC when true effect is		
	1.5	2	4	1.5	2	4
Case−control						
Age & sex matched controls	54.6	57.1	60.5	57.2	60.3	65.6
Age & sex adjusted, using random controls	54.7	58.9	63.0	55.3	57.6	65.5
Cohort method						
Unadjusted, using outpatient visits as comparator	57.6	61.5	72.6	60.3	63.2	69.1
PS matching, using outpatient visits as comparator	57.2	59.9	64.1	54.2	56.4	59.8
Unadjusted, using random days as comparator	54.0	55.5	60.5	54.8	55.5	60.5
PS matching, using random days as comparator	56.4	57.9	63.7	56.6	58.2	64.0
PS stratification, using outpatient visits as comparator	59.2	62.4	76.5	59.5	62.1	65.9
PS stratification, using random days as comparator	56.4	57.5	69.3	56.2	57.9	62.3
PS weighting, using outpatient visits as comparator	59.8	64.5	81.2	56.4	63.5	67.7
PS weighting, using random days as comparator	55.9	58.0	70.3	55.3	57.4	61.6
Per−month PS matching, using outpatient visits as comparator	55.7	57.6	60.2	53.3	55.3	57.9
Per−month PS matching, using random days as comparator	58.8	61.7	67.5	53.8	60.1	61.9
Historical comparator						
Unadjusted, using entire historic period	53.9	56.8	61.6	58.6	62.6	67.9
Age & sex adjusted, using entire historic period	54.0	57.4	61.6	59.7	62.6	68.6
Unadjusted, using TaR after historic visit	55.8	58.0	63.3	60.4	64.4	70.2
Age & sex adjusted, using TaR after historic visit	54.7	56.9	62.2	59.3	63.3	69.1
Unadjusted, using entire historic period, filtered	53.9	56.8	61.6	59.8	62.7	68.1
Age & sex adjusted, using entire historic period, filtered	54.0	57.4	61.7	59.9	62.7	68.8
Unadjusted, using TaR after historic visit, filtered	55.8	58.0	63.4	60.0	63.9	69.9
Age & sex adjusted, using TaR after historic visit, filtered	54.7	56.9	62.3	58.8	62.8	69.3
SCCS / SCRI						
Unadjusted SCCS excluding pre−vaccination window	59.9	62.2	67.5	59.9	63.4	69.3
Age & season adjusted SCCS excluding pre−vaccination window	58.9	61.2	67.3	60.6	63.5	70.1
SCRI with prior control interval	54.0	55.2	59.6	63.7	64.6	69.3
SCRI with posterior control interval	55.3	59.3	65.7	55.3	59.2	65.7
Unadjusted SCCS excluding all pre−vaccination time	58.9	61.7	66.9	59.5	63.5	68.7

Figure 68: The area under the receiver operator curve (AUC) based on the effect-size estimate at the end of the study period in the MDCR database for H1N1 vaccination.

	Uncalibrated AUC when true effect is			Calibrated AUC when true effect is		
	1.5	2	4	1.5	2	4
Case−control						
Age & sex matched controls	58.2	63.7	70.6	62.2	67.6	76.8
Age & sex adjusted, using random controls	61.8	67.0	74.4	61.8	70.3	79.4
Cohort method						
Unadjusted, using outpatient visits as comparator	61.3	68.4	82.6	62.5	71.4	83.2
PS matching, using outpatient visits as comparator	70.4	79.6	90.2	67.8	76.1	83.7
Unadjusted, using random days as comparator	69.2	76.2	85.9	68.7	77.1	87.0
PS matching, using random days as comparator	70.5	78.5	87.0	70.5	78.5	87.0
PS stratification, using outpatient visits as comparator	71.4	83.1	92.8	73.4	81.1	88.4
PS stratification, using random days as comparator	69.3	77.8	87.3	70.6	79.2	87.9
PS weighting, using outpatient visits as comparator	63.8	73.5	89.0	64.7	72.3	85.7
PS weighting, using random days as comparator	66.8	77.2	88.0	67.4	75.9	85.2
Per−month PS matching, using outpatient visits as comparator	68.6	77.8	87.4	69.9	77.8	85.3
Per−month PS matching, using random days as comparator	69.8	78.2	84.8	70.4	79.1	85.4
Historical comparator						
Unadjusted, using entire historic period	71.8	79.5	87.7	73.2	81.6	90.0
Age & sex adjusted, using entire historic period	70.4	77.4	84.2	73.3	81.1	88.6
Unadjusted, using TaR after historic visit	68.9	80.5	91.9	70.3	79.7	89.0
Age & sex adjusted, using TaR after historic visit	74.8	83.9	90.7	73.9	83.1	89.8
Unadjusted, using entire historic period, filtered	71.9	79.4	87.2	73.4	81.6	89.6
Age & sex adjusted, using entire historic period, filtered	70.4	77.2	83.6	73.5	81.2	88.2
Unadjusted, using TaR after historic visit, filtered	68.8	80.5	91.7	70.4	79.6	88.6
Age & sex adjusted, using TaR after historic visit, filtered	75.0	84.0	90.4	74.1	83.1	89.5
SCCS / SCRI						
Unadjusted SCCS excluding pre−vaccination window	73.5	81.0	89.0	73.3	80.8	88.8
Age & season adjusted SCCS excluding pre−vaccination window	73.0	80.5	87.5	73.4	80.7	87.7
SCRI with prior control interval	73.0	81.1	87.3	73.0	80.9	87.3
SCRI with posterior control interval	68.5	76.2	84.5	70.1	77.8	86.7
Unadjusted SCCS excluding all pre−vaccination time	72.9	80.4	88.0	74.4	82.5	90.1

Figure 69: The area under the receiver operator curve (AUC) based on the effect-size estimate at the end of the study period in the CCAE database for H1N1 vaccination.

12.2 AUC for Seasonal flu vaccination (Fluvirin)

	Uncalibrated AUC when true effect is			Calibrated AUC when true effect is		
	1.5	2	4	1.5	2	4
Case−control						
Age & sex matched controls	50.4	51.6	52.1	54.7	55.9	56.3
Age & sex adjusted, using random controls	50.3	50.4	50.7	54.9	56.0	58.2
Cohort method						
Unadjusted, using outpatient visits as comparator	57.9	60.8	70.1	55.8	60.6	66.8
PS matching, using outpatient visits as comparator	55.3	56.3	59.3	53.1	53.3	55.4
Unadjusted, using random days as comparator	59.4	60.3	62.4	54.8	63.2	66.1
PS matching, using random days as comparator						
PS stratification, using outpatient visits as comparator	66.5	71.6	88.8	59.6	65.8	70.1
PS stratification, using random days as comparator	57.8	60.6	74.1	57.2	60.5	67.0
PS weighting, using outpatient visits as comparator	66.8	72.9	89.5	57.5	62.0	70.9
PS weighting, using random days as comparator	50.0	50.0	50.0	50.0	50.0	50.0
Per−month PS matching, using outpatient visits as comparator						
Per−month PS matching, using random days as comparator						
Historical comparator						
Unadjusted, using entire historic period	53.4	55.5	59.3	59.1	62.8	71.2
Age & sex adjusted, using entire historic period	53.3	55.2	59.3	60.1	64.3	71.8
Unadjusted, using TaR after historic visit	57.3	60.4	64.4	59.0	63.8	71.7
Age & sex adjusted, using TaR after historic visit	56.5	60.0	64.1	57.7	61.7	69.8
Unadjusted, using entire historic period, filtered	53.4	55.4	59.0	58.6	64.1	70.6
Age & sex adjusted, using entire historic period, filtered	53.3	55.1	59.1	59.6	64.4	71.2
Unadjusted, using TaR after historic visit, filtered	57.3	60.4	64.2	60.2	63.8	71.7
Age & sex adjusted, using TaR after historic visit, filtered	56.5	60.0	63.9	58.2	62.3	69.7
SCCS / SCRI						
Unadjusted SCCS excluding pre−vaccination window	55.8	58.7	63.5	56.5	60.7	65.5
Age & season adjusted SCCS excluding pre−vaccination window	56.0	60.1	63.0	55.8	59.9	64.6
SCRI with prior control interval	55.7	57.1	58.8	56.6	58.1	59.9
SCRI with posterior control interval	54.2	55.2	58.6	60.9	62.5	65.3
Unadjusted SCCS excluding all pre−vaccination time	55.9	58.6	61.5	56.6	59.9	63.5

Figure 70: The area under the receiver operator curve (AUC) based on the effect-size estimate at the end of the study period in the Optum EHR database for Seasonal flu vaccination (Fluvirin).

	Uncalibrated AUC when true effect is			Calibrated AUC when true effect is		
	1.5	2	4	1.5	2	4
Case−control						
Age & sex matched controls	55.8	57.3	59.2	56.6	59.6	61.5
Age & sex adjusted, using random controls	54.0	55.9	58.2	57.6	60.9	64.7
Cohort method						
Unadjusted, using outpatient visits as comparator	58.0	60.9	67.7	54.7	58.6	64.0
PS matching, using outpatient visits as comparator	50.0	50.0	50.0	50.0	50.0	50.0
Unadjusted, using random days as comparator	55.4	55.7	59.1	51.5	54.3	59.3
PS matching, using random days as comparator	50.0	50.0	50.0	50.0	50.0	50.0
PS stratification, using outpatient visits as comparator	64.1	70.1	87.6	57.5	62.9	66.9
PS stratification, using random days as comparator	59.0	60.4	75.2	58.9	62.0	65.4
PS weighting, using outpatient visits as comparator	65.8	71.0	90.2	56.2	62.2	66.9
PS weighting, using random days as comparator	50.0	50.0	50.0	50.0	50.0	50.0
Per−month PS matching, using outpatient visits as comparator						
Per−month PS matching, using random days as comparator						
Historical comparator						
Unadjusted, using entire historic period	54.5	56.6	59.6	55.8	60.5	66.8
Age & sex adjusted, using entire historic period	56.1	59.0	62.5	61.3	64.9	71.7
Unadjusted, using TaR after historic visit	56.0	58.8	63.0	58.6	62.1	68.3
Age & sex adjusted, using TaR after historic visit	60.0	64.7	69.6	61.4	66.0	70.9
Unadjusted, using entire historic period, filtered	54.5	56.6	59.6	55.2	60.6	66.3
Age & sex adjusted, using entire historic period, filtered	56.1	59.1	62.6	58.1	62.4	68.0
Unadjusted, using TaR after historic visit, filtered	56.1	58.9	63.1	58.7	62.2	69.1
Age & sex adjusted, using TaR after historic visit, filtered	60.2	64.9	69.8	60.2	64.2	69.8
SCCS / SCRI						
Unadjusted SCCS excluding pre−vaccination window	57.1	59.9	65.3	58.4	61.2	66.6
Age & season adjusted SCCS excluding pre−vaccination window	57.2	59.9	65.3	57.0	59.7	65.0
SCRI with prior control interval	54.7	57.5	62.2	54.1	57.9	60.5
SCRI with posterior control interval	53.8	57.8	59.1	62.1	64.5	67.4
Unadjusted SCCS excluding all pre−vaccination time	57.1	60.6	66.1	58.4	61.2	67.4

Figure 71: The area under the receiver operator curve (AUC) based on the effect-size estimate at the end of the study period in the MDCD database for Seasonal flu vaccination (Fluvirin).

	Uncalibrated AUC when true effect is			Calibrated AUC when true effect is		
	1.5	2	4	1.5	2	4
Case−control						
Age & sex matched controls	51.1	51.1	51.1	50.0	50.0	50.0
Age & sex adjusted, using random controls	50.1	50.1	50.1	50.0	50.0	50.0
Cohort method						
Unadjusted, using outpatient visits as comparator	52.9	52.9	55.0	50.0	50.0	50.0
PS matching, using outpatient visits as comparator	50.3	50.3	50.3	50.0	50.0	50.0
Unadjusted, using random days as comparator	53.1	53.1	54.2	50.0	50.0	50.0
PS matching, using random days as comparator	50.1	50.1	50.1	50.0	50.0	50.0
PS stratification, using outpatient visits as comparator	55.6	57.3	76.8	52.1	52.1	54.2
PS stratification, using random days as comparator	51.1	51.2	61.9	50.0	50.0	50.0
PS weighting, using outpatient visits as comparator	57.0	57.4	79.1	51.1	51.1	53.1
PS weighting, using random days as comparator	51.3	51.5	65.2	50.0	50.0	50.0
Per−month PS matching, using outpatient visits as comparator						
Per−month PS matching, using random days as comparator						
Historical comparator						
Unadjusted, using entire historic period	51.1	51.1	51.2	51.1	52.1	54.2
Age & sex adjusted, using entire historic period	51.1	51.1	51.2	52.1	53.1	55.1
Unadjusted, using TaR after historic visit	51.1	51.1	51.2	51.1	52.1	54.2
Age & sex adjusted, using TaR after historic visit	51.1	51.1	51.2	52.0	52.1	54.2
Unadjusted, using entire historic period, filtered	51.1	51.1	51.2	51.1	52.1	54.2
Age & sex adjusted, using entire historic period, filtered	51.1	51.1	51.2	52.1	53.1	55.1
Unadjusted, using TaR after historic visit, filtered	51.1	51.1	51.2	51.1	52.1	54.2
Age & sex adjusted, using TaR after historic visit, filtered	51.1	51.1	51.2	52.0	52.1	54.2
SCCS / SCRI						
Unadjusted SCCS excluding pre−vaccination window	51.1	51.1	51.1	50.0	50.0	50.0
Age & season adjusted SCCS excluding pre−vaccination window	51.1	51.1	51.1	50.0	50.0	50.0
SCRI with prior control interval	50.0	50.0	51.1	50.0	50.0	50.0
SCRI with posterior control interval	50.0	51.1	51.1	50.0	50.0	50.0
Unadjusted SCCS excluding all pre−vaccination time	51.1	51.1	51.1	50.0	50.0	50.0

Figure 72: The area under the receiver operator curve (AUC) based on the effect-size estimate at the end of the study period in the MDCR database for Seasonal flu vaccination (Fluvirin).

	Uncalibrated AUC when true effect is			Calibrated AUC when true effect is		
	1.5	2	4	1.5	2	4
Case−control						
Age & sex matched controls	55.2	59.5	64.4	57.6	61.3	68.1
Age & sex adjusted, using random controls	59.4	64.1	69.7	58.8	66.4	72.0
Cohort method						
Unadjusted, using outpatient visits as comparator	61.3	68.4	82.5	60.6	66.5	78.1
PS matching, using outpatient visits as comparator	60.8	66.8	74.6	61.7	68.1	74.7
Unadjusted, using random days as comparator	60.8	65.0	74.3	63.8	67.3	76.6
PS matching, using random days as comparator	62.6	67.9	78.4	64.4	70.8	81.3
PS stratification, using outpatient visits as comparator	64.0	72.2	89.3	62.1	69.2	79.1
PS stratification, using random days as comparator	63.5	71.4	86.7	64.5	74.1	82.3
PS weighting, using outpatient visits as comparator	64.4	71.6	87.7	59.8	65.4	75.2
PS weighting, using random days as comparator	63.9	70.0	86.5	65.3	74.1	82.1
Per−month PS matching, using outpatient visits as comparator						
Per−month PS matching, using random days as comparator						
Historical comparator						
Unadjusted, using entire historic period	62.6	68.8	76.2	66.3	73.3	83.3
Age & sex adjusted, using entire historic period	64.9	71.7	79.7	67.1	73.6	83.4
Unadjusted, using TaR after historic visit	65.2	72.1	81.9	66.3	72.8	83.4
Age & sex adjusted, using TaR after historic visit	63.6	72.8	82.6	64.3	72.4	82.2
Unadjusted, using entire historic period, filtered	62.8	69.2	76.6	65.6	72.3	82.1
Age & sex adjusted, using entire historic period, filtered	65.2	72.2	80.3	66.4	73.0	82.6
Unadjusted, using TaR after historic visit, filtered	65.6	72.6	82.7	65.2	72.2	82.3
Age & sex adjusted, using TaR after historic visit, filtered	63.9	73.5	83.4	63.9	71.9	81.8
SCCS / SCRI						
Unadjusted SCCS excluding pre−vaccination window	64.5	70.5	78.0	66.0	72.3	80.5
Age & season adjusted SCCS excluding pre−vaccination window	64.3	71.1	77.9	66.1	72.7	80.4
SCRI with prior control interval	59.1	63.3	69.8	62.9	66.6	74.3
SCRI with posterior control interval	58.2	64.9	71.3	61.3	66.1	75.0
Unadjusted SCCS excluding all pre−vaccination time	61.9	67.8	76.7	63.0	69.7	79.4

Figure 73: The area under the receiver operator curve (AUC) based on the effect-size estimate at the end of the study period in the CCAE database for Seasonal flu vaccination (Fluvirin).

12.3 AUC for Seasonal flu vaccination (Fluzone)

	Uncalibrated AUC when true effect is			Calibrated AUC when true effect is		
	1.5	2	4	1.5	2	4
Case−control						
Age & sex matched controls	58.9	64.4	72.3	61.9	70.1	78.6
Age & sex adjusted, using random controls	58.9	65.4	75.1	61.7	69.8	82.0
Cohort method						
Unadjusted, using outpatient visits as comparator	66.0	75.5	89.1	66.2	75.4	88.2
PS matching, using outpatient visits as comparator	66.5	74.6	87.5	66.8	74.0	86.0
Unadjusted, using random days as comparator	61.9	69.5	80.2	64.0	72.4	84.5
PS matching, using random days as comparator						
PS stratification, using outpatient visits as comparator	69.5	80.6	94.7	69.3	80.4	92.2
PS stratification, using random days as comparator	65.6	74.2	88.4	66.3	76.1	89.6
PS weighting, using outpatient visits as comparator	67.1	76.8	93.0	65.5	74.2	87.6
PS weighting, using random days as comparator	50.3	50.4	50.6	50.0	50.0	50.0
Per−month PS matching, using outpatient visits as comparator						
Per−month PS matching, using random days as comparator						
Historical comparator						
Unadjusted, using entire historic period	66.7	75.8	87.6	68.0	77.7	90.3
Age & sex adjusted, using entire historic period	66.6	76.0	87.7	67.8	77.6	90.2
Unadjusted, using TaR after historic visit	67.7	77.6	90.0	67.9	77.6	90.2
Age & sex adjusted, using TaR after historic visit	68.5	77.7	89.9	69.5	78.6	91.0
Unadjusted, using entire historic period, filtered	66.8	75.9	87.5	68.3	77.8	90.5
Age & sex adjusted, using entire historic period, filtered	66.7	76.1	87.7	68.0	77.9	90.4
Unadjusted, using TaR after historic visit, filtered	67.9	78.0	90.2	68.2	78.0	90.5
Age & sex adjusted, using TaR after historic visit, filtered	68.7	78.0	90.1	69.7	78.9	91.2
SCCS / SCRI						
Unadjusted SCCS excluding pre−vaccination window	71.1	80.9	91.4	71.2	81.5	92.2
Age & season adjusted SCCS excluding pre−vaccination window	69.5	78.9	90.4	68.1	77.9	89.1
SCRI with prior control interval	64.2	72.7	85.2	65.7	75.0	88.4
SCRI with posterior control interval	67.7	75.9	85.9	69.6	79.0	89.6
Unadjusted SCCS excluding all pre−vaccination time	70.2	79.6	90.6	71.3	81.2	92.5

Figure 74: The area under the receiver operator curve (AUC) based on the effect-size estimate at the end of the study period in the Optum EHR database for Seasonal flu vaccination (Fluzone).

	Uncalibrated AUC when true effect is			Calibrated AUC when true effect is		
	1.5	2	4	1.5	2	4
Case−control						
Age & sex matched controls	51.2	51.3	52.5	50.3	52.3	53.4
Age & sex adjusted, using random controls	51.1	51.1	51.2	51.1	53.1	54.2
Cohort method						
Unadjusted, using outpatient visits as comparator	56.2	57.1	58.3	57.0	58.1	59.2
PS matching, using outpatient visits as comparator	52.2	52.4	54.5	50.1	52.1	53.2
Unadjusted, using random days as comparator	52.1	52.1	54.2	51.1	53.1	55.2
PS matching, using random days as comparator						
PS stratification, using outpatient visits as comparator	57.4	59.7	77.5	50.4	52.4	54.6
PS stratification, using random days as comparator	54.6	55.8	70.7	55.8	57.0	61.1
PS weighting, using outpatient visits as comparator	59.7	60.3	80.4	52.2	53.3	57.4
PS weighting, using random days as comparator	50.0	50.0	50.0	50.0	50.0	50.0
Per−month PS matching, using outpatient visits as comparator						
Per−month PS matching, using random days as comparator						
Historical comparator						
Unadjusted, using entire historic period	51.4	51.8	52.6	53.9	56.6	59.9
Age & sex adjusted, using entire historic period	51.7	52.0	53.6	55.8	57.7	60.8
Unadjusted, using TaR after historic visit	52.2	52.6	53.5	52.2	55.0	59.1
Age & sex adjusted, using TaR after historic visit	51.6	53.6	55.1	54.8	57.6	61.6
Unadjusted, using entire historic period, filtered	51.4	51.8	52.5	54.7	56.7	59.9
Age & sex adjusted, using entire historic period, filtered	51.7	52.0	53.5	56.6	58.5	61.7
Unadjusted, using TaR after historic visit, filtered	52.2	52.6	53.4	53.1	55.9	60.0
Age & sex adjusted, using TaR after historic visit, filtered	51.5	53.6	55.1	54.0	56.8	60.8
SCCS / SCRI						
Unadjusted SCCS excluding pre−vaccination window	54.0	55.1	57.6	56.6	57.7	61.0
Age & season adjusted SCCS excluding pre−vaccination window	53.2	54.3	56.7	54.9	56.9	60.1
SCRI with prior control interval	55.7	55.9	56.0	55.0	55.1	55.2
SCRI with posterior control interval	51.4	54.4	55.6	55.0	57.1	59.3
Unadjusted SCCS excluding all pre−vaccination time	53.2	56.1	57.5	54.9	56.9	60.1

Figure 75: The area under the receiver operator curve (AUC) based on the effect-size estimate at the end of the study period in the MDCD database for Seasonal flu vaccination (Fluzone).

	Uncalibrated AUC when true effect is 1.5	2	4	Calibrated AUC when true effect is 1.5	2	4
**Case−control**						
Age & sex matched controls	58.8	66.8	75.1	59.4	65.7	71.1
Age & sex adjusted, using random controls	60.4	66.2	76.5	61.3	68.0	75.2
**Cohort method**						
Unadjusted, using outpatient visits as comparator	59.5	64.8	77.8	59.4	66.8	75.5
PS matching, using outpatient visits as comparator	59.8	64.2	73.8	57.3	62.9	69.3
Unadjusted, using random days as comparator	59.7	64.5	75.7	57.6	63.4	72.3
PS matching, using random days as comparator	62.6	67.3	75.0	58.7	64.0	70.1
PS stratification, using outpatient visits as comparator	63.8	72.1	88.3	63.0	70.3	78.0
PS stratification, using random days as comparator	59.9	66.8	81.2	60.4	66.1	74.3
PS weighting, using outpatient visits as comparator	63.9	72.1	89.4	57.8	63.0	72.1
PS weighting, using random days as comparator	60.1	65.2	80.0	59.0	64.1	71.6
Per−month PS matching, using outpatient visits as comparator						
Per−month PS matching, using random days as comparator						
**Historical comparator**						
Unadjusted, using entire historic period	62.5	68.3	76.3	62.1	68.7	77.5
Age & sex adjusted, using entire historic period	61.4	67.9	75.9	62.6	69.1	77.9
Unadjusted, using TaR after historic visit	60.8	68.1	78.1	61.2	68.5	77.3
Age & sex adjusted, using TaR after historic visit	61.0	68.3	77.9	61.8	68.8	77.5
Unadjusted, using entire historic period, filtered	62.8	68.7	76.7	62.8	68.7	76.7
Age & sex adjusted, using entire historic period, filtered	61.6	68.3	76.3	61.6	67.9	75.9
Unadjusted, using TaR after historic visit, filtered	61.1	68.5	78.7	61.1	68.1	76.5
Age & sex adjusted, using TaR after historic visit, filtered	61.3	68.8	78.4	62.5	69.7	77.2
**SCCS / SCRI**						
Unadjusted SCCS excluding pre−vaccination window	64.3	70.6	76.7	64.8	70.1	76.2
Age & season adjusted SCCS excluding pre−vaccination window	63.0	69.3	75.8	62.5	68.3	74.8
SCRI with prior control interval	55.3	59.9	66.6	54.8	59.7	66.7
SCRI with posterior control interval	57.4	62.9	68.7	57.5	62.4	68.4
Unadjusted SCCS excluding all pre−vaccination time	64.7	70.3	78.0	65.2	70.8	77.1

Figure 76: The area under the receiver operator curve (AUC) based on the effect-size estimate at the end of the study period in the MDCR database for Seasonal flu vaccination (Fluzone).

	Uncalibrated AUC when true effect is			Calibrated AUC when true effect is		
	1.5	2	4	1.5	2	4
Case−control						
Age & sex matched controls	50.0	50.0	50.0	50.0	50.0	50.0
Age & sex adjusted, using random controls	50.0	50.0	50.0	50.0	50.0	50.0
Cohort method						
Unadjusted, using outpatient visits as comparator	50.3	50.3	50.3	50.0	50.0	50.0
PS matching, using outpatient visits as comparator	51.1	52.1	52.2	50.0	50.0	50.0
Unadjusted, using random days as comparator	50.1	50.1	50.1	50.0	50.0	50.0
PS matching, using random days as comparator	52.2	52.2	54.3	50.0	50.0	50.0
PS stratification, using outpatient visits as comparator	53.1	54.3	69.1	50.1	50.1	52.2
PS stratification, using random days as comparator	51.6	52.8	59.2	50.0	50.0	50.0
PS weighting, using outpatient visits as comparator	53.7	53.7	72.2	52.1	52.1	53.2
PS weighting, using random days as comparator	51.2	51.2	63.4	50.0	50.0	50.0
Per−month PS matching, using outpatient visits as comparator						
Per−month PS matching, using random days as comparator						
Historical comparator						
Unadjusted, using entire historic period	50.1	50.2	50.3	51.1	53.0	53.1
Age & sex adjusted, using entire historic period	51.1	51.2	51.3	52.0	52.1	53.2
Unadjusted, using TaR after historic visit	50.1	50.2	50.3	52.0	52.1	53.2
Age & sex adjusted, using TaR after historic visit	51.1	51.2	51.3	52.1	52.1	53.2
Unadjusted, using entire historic period, filtered	50.1	50.2	50.2	50.1	52.1	52.1
Age & sex adjusted, using entire historic period, filtered	51.1	51.1	51.3	52.0	52.1	54.1
Unadjusted, using TaR after historic visit, filtered	50.1	50.2	50.3	51.1	51.1	52.2
Age & sex adjusted, using TaR after historic visit, filtered	51.1	51.1	51.3	52.0	52.1	53.2
SCCS / SCRI						
Unadjusted SCCS excluding pre−vaccination window	50.0	50.0	50.1	50.0	50.0	50.0
Age & season adjusted SCCS excluding pre−vaccination window	50.0	50.0	50.1	50.0	50.0	50.0
SCRI with prior control interval						
SCRI with posterior control interval						
Unadjusted SCCS excluding all pre−vaccination time	50.0	50.0	50.1	50.0	50.0	50.0

Figure 77: The area under the receiver operator curve (AUC) based on the effect-size estimate at the end of the study period in the CCAE database for Seasonal flu vaccination (Fluzone).

12.4 AUC for Seasonal flu vaccination (All)

	Uncalibrated AUC when true effect is			Calibrated AUC when true effect is		
	1.5	2	4	1.5	2	4
Case−control						
Age & sex matched controls	76.1	86.9	94.1	78.1	89.5	96.9
Age & sex adjusted, using random controls	80.8	89.9	95.1	82.7	92.1	97.4
Cohort method						
Unadjusted, using outpatient visits as comparator	75.0	86.8	98.1	75.4	87.3	98.3
PS matching, using outpatient visits as comparator	77.3	88.1	96.3	77.8	88.6	96.4
Unadjusted, using random days as comparator	73.7	85.3	95.5	74.4	86.4	96.8
PS matching, using random days as comparator						
PS stratification, using outpatient visits as comparator	79.5	90.3	97.0	79.8	90.8	97.3
PS stratification, using random days as comparator	78.1	90.6	98.6	78.4	91.0	99.0
PS weighting, using outpatient visits as comparator	76.1	87.0	97.0	76.4	87.3	96.7
PS weighting, using random days as comparator	50.7	51.1	51.8	50.0	50.0	50.0
Per−month PS matching, using outpatient visits as comparator						
Per−month PS matching, using random days as comparator						
Historical comparator						
Unadjusted, using entire historic period	79.3	91.2	98.6	79.3	91.2	98.6
Age & sex adjusted, using entire historic period	84.5	94.1	98.6	84.5	94.1	98.6
Unadjusted, using TaR after historic visit	79.5	90.7	98.4	79.5	90.7	98.4
Age & sex adjusted, using TaR after historic visit	83.5	94.0	98.8	83.5	94.0	98.8
Unadjusted, using entire historic period, filtered	79.3	91.2	98.6	79.6	91.7	99.1
Age & sex adjusted, using entire historic period, filtered	84.5	94.1	98.6	84.9	94.5	99.1
Unadjusted, using TaR after historic visit, filtered	79.8	91.0	98.7	79.9	91.2	98.9
Age & sex adjusted, using TaR after historic visit, filtered	83.7	94.2	99.0	83.9	94.5	99.3
SCCS / SCRI						
Unadjusted SCCS excluding pre−vaccination window	84.5	95.4	99.4	84.5	95.4	99.4
Age & season adjusted SCCS excluding pre−vaccination window	80.7	92.7	98.5	78.5	90.0	95.5
SCRI with prior control interval	79.7	87.4	95.4	80.1	87.8	95.8
SCRI with posterior control interval	81.0	90.7	97.7	81.3	91.0	98.0
Unadjusted SCCS excluding all pre−vaccination time	85.7	94.2	99.6	85.7	94.2	99.6

Figure 78: The area under the receiver operator curve (AUC) based on the effect-size estimate at the end of the study period in the Optum EHR database for Seasonal flu vaccination (All).

	Uncalibrated AUC when true effect is			Calibrated AUC when true effect is		
	1.5	2	4	1.5	2	4
Case−control						
Age & sex matched controls	68.1	75.2	80.0	74.0	82.5	87.8
Age & sex adjusted, using random controls	73.8	82.4	88.8	77.0	86.4	93.5
Cohort method						
Unadjusted, using outpatient visits as comparator	67.8	78.1	93.7	70.2	79.7	91.1
PS matching, using outpatient visits as comparator	83.5	92.0	97.1	81.9	89.5	93.7
Unadjusted, using random days as comparator	69.9	78.7	86.1	72.3	83.1	90.6
PS matching, using random days as comparator	75.3	85.5	94.2	74.4	84.6	92.9
PS stratification, using outpatient visits as comparator	78.8	88.2	95.9	77.4	86.0	92.4
PS stratification, using random days as comparator	71.1	81.5	91.7	71.1	81.6	91.5
PS weighting, using outpatient visits as comparator	77.7	88.0	96.0	76.4	85.8	92.5
PS weighting, using random days as comparator	69.2	78.8	91.4	68.1	77.3	88.6
Per−month PS matching, using outpatient visits as comparator						
Per−month PS matching, using random days as comparator						
Historical comparator						
Unadjusted, using entire historic period	74.5	83.1	88.1	78.7	88.1	93.6
Age & sex adjusted, using entire historic period	75.2	83.3	87.6	79.8	89.2	93.7
Unadjusted, using TaR after historic visit	75.3	85.7	93.5	75.2	85.5	93.4
Age & sex adjusted, using TaR after historic visit	77.0	86.5	92.8	77.2	87.2	93.6
Unadjusted, using entire historic period, filtered	74.7	83.2	88.3	78.6	88.0	93.4
Age & sex adjusted, using entire historic period, filtered	75.3	83.4	87.7	79.2	88.5	93.1
Unadjusted, using TaR after historic visit, filtered	75.7	86.2	94.1	75.1	85.9	93.5
Age & sex adjusted, using TaR after historic visit, filtered	77.4	87.0	93.3	77.3	87.4	93.7
SCCS / SCRI						
Unadjusted SCCS excluding pre−vaccination window	82.7	89.8	93.9	82.7	89.5	93.7
Age & season adjusted SCCS excluding pre−vaccination window	81.3	88.3	92.6	80.5	87.3	91.6
SCRI with prior control interval	77.0	85.7	92.3	77.7	86.5	93.3
SCRI with posterior control interval	78.6	85.9	91.5	80.1	87.8	93.7
Unadjusted SCCS excluding all pre−vaccination time	79.2	87.3	93.0	79.8	87.7	93.6

Figure 79: The area under the receiver operator curve (AUC) based on the effect-size estimate at the end of the study period in the MDCD database for Seasonal flu vaccination (All).

	Uncalibrated AUC when true effect is 1.5	2	4	Calibrated AUC when true effect is 1.5	2	4
Case−control						
Age & sex matched controls	70.3	77.3	81.5	71.0	78.0	82.2
Age & sex adjusted, using random controls	75.0	84.2	88.7	77.3	87.1	91.6
Cohort method						
Unadjusted, using outpatient visits as comparator	66.3	75.9	91.2	67.1	77.1	87.4
PS matching, using outpatient visits as comparator	67.3	76.4	87.0	67.9	76.6	86.1
Unadjusted, using random days as comparator	68.8	76.9	85.7	69.8	78.8	87.5
PS matching, using random days as comparator	70.0	77.7	88.2	69.8	76.7	87.2
PS stratification, using outpatient visits as comparator	69.0	79.2	92.6	69.4	78.2	87.7
PS stratification, using random days as comparator	69.0	77.9	87.8	68.9	77.6	87.1
PS weighting, using outpatient visits as comparator	66.7	76.4	92.2	66.5	74.8	86.2
PS weighting, using random days as comparator	65.1	72.6	85.4	64.8	72.6	84.5
Per−month PS matching, using outpatient visits as comparator						
Per−month PS matching, using random days as comparator						
Historical comparator						
Unadjusted, using entire historic period	75.2	83.1	87.6	79.0	87.6	92.3
Age & sex adjusted, using entire historic period	75.0	82.9	87.4	79.2	87.7	92.3
Unadjusted, using TaR after historic visit	72.5	83.1	90.2	72.8	83.8	91.0
Age & sex adjusted, using TaR after historic visit	73.0	83.3	90.1	73.6	84.8	91.6
Unadjusted, using entire historic period, filtered	75.6	83.5	87.9	78.5	87.1	91.5
Age & sex adjusted, using entire historic period, filtered	75.2	83.2	87.6	78.7	87.3	91.7
Unadjusted, using TaR after historic visit, filtered	73.1	83.8	90.8	73.1	83.8	90.8
Age & sex adjusted, using TaR after historic visit, filtered	73.6	84.0	90.6	73.4	84.0	90.6
SCCS / SCRI						
Unadjusted SCCS excluding pre−vaccination window	75.2	83.1	89.6	75.9	83.8	90.3
Age & season adjusted SCCS excluding pre−vaccination window	74.1	80.1	82.0	77.0	82.7	85.0
SCRI with prior control interval	66.5	75.1	83.5	69.8	78.3	87.9
SCRI with posterior control interval	71.7	79.8	86.6	72.8	81.6	88.6
Unadjusted SCCS excluding all pre−vaccination time	73.2	81.4	88.3	73.5	82.3	89.4

Figure 80: The area under the receiver operator curve (AUC) based on the effect-size estimate at the end of the study period in the MDCR database for Seasonal flu vaccination (All).

	Uncalibrated AUC when true effect is			Calibrated AUC when true effect is		
	1.5	2	4	1.5	2	4
Case−control						
Age & sex matched controls	72.3	81.4	88.3	73.8	83.6	91.1
Age & sex adjusted, using random controls	76.5	84.8	90.2	78.8	87.3	93.1
Cohort method						
Unadjusted, using outpatient visits as comparator	65.6	75.1	91.2	68.2	77.4	89.1
PS matching, using outpatient visits as comparator	76.7	86.7	95.1	76.9	85.8	93.7
Unadjusted, using random days as comparator	73.3	83.1	89.8	75.0	85.7	92.3
PS matching, using random days as comparator	76.7	84.5	90.9	77.8	85.7	92.1
PS stratification, using outpatient visits as comparator	74.7	86.3	96.1	75.9	85.9	94.0
PS stratification, using random days as comparator	77.0	85.0	92.6	78.1	86.2	93.7
PS weighting, using outpatient visits as comparator	76.0	87.9	97.0	77.9	87.7	94.8
PS weighting, using random days as comparator	71.4	81.3	92.5	72.1	82.0	92.3
Per−month PS matching, using outpatient visits as comparator						
Per−month PS matching, using random days as comparator						
Historical comparator						
Unadjusted, using entire historic period	76.9	84.4	89.9	78.8	86.5	92.4
Age & sex adjusted, using entire historic period	79.7	86.1	90.1	81.8	88.4	92.6
Unadjusted, using TaR after historic visit	75.4	86.4	93.8	75.5	85.8	93.0
Age & sex adjusted, using TaR after historic visit	78.0	88.1	93.5	77.3	87.2	92.6
Unadjusted, using entire historic period, filtered	77.1	84.8	90.2	79.2	87.1	92.7
Age & sex adjusted, using entire historic period, filtered	80.0	86.5	90.4	82.1	88.6	92.8
Unadjusted, using TaR after historic visit, filtered	76.2	87.6	95.0	75.8	86.2	93.3
Age & sex adjusted, using TaR after historic visit, filtered	78.9	89.3	94.7	77.9	87.5	93.0
SCCS / SCRI						
Unadjusted SCCS excluding pre−vaccination window	84.7	89.6	92.6	85.8	90.5	93.6
Age & season adjusted SCCS excluding pre−vaccination window	83.8	88.0	91.2	82.6	86.6	89.7
SCRI with prior control interval	75.1	83.2	90.6	76.2	84.6	92.2
SCRI with posterior control interval	83.0	88.5	91.3	84.8	90.4	93.2
Unadjusted SCCS excluding all pre−vaccination time	84.6	88.6	91.8	86.1	90.2	93.2

Figure 81: The area under the receiver operator curve (AUC) based on the effect-size estimate at the end of the study period in the CCAE database for Seasonal flu vaccination (All).

12.5 AUC for Zoster vaccination (Shingrix)

	Uncalibrated AUC when true effect is			Calibrated AUC when true effect is		
	1.5	2	4	1.5	2	4
Case−control						
Age & sex matched controls	58.6	63.1	71.0	61.8	67.7	76.0
Age & sex adjusted, using random controls	60.4	66.3	74.2	60.4	69.6	80.6
Cohort method						
Unadjusted, using outpatient visits as comparator	63.3	71.0	85.6	63.6	71.1	84.0
PS matching, using outpatient visits as comparator	69.0	78.0	90.3	70.8	80.1	89.6
Unadjusted, using random days as comparator	61.2	67.8	77.5	61.9	70.2	80.7
PS matching, using random days as comparator						
PS stratification, using outpatient visits as comparator	70.1	81.4	95.7	70.7	80.7	91.6
PS stratification, using random days as comparator						
PS weighting, using outpatient visits as comparator	70.8	81.2	95.7	72.2	81.5	92.3
PS weighting, using random days as comparator						
Per−month PS matching, using outpatient visits as comparator						
Per−month PS matching, using random days as comparator						
Historical comparator						
Unadjusted, using entire historic period	63.6	70.6	82.6	66.1	73.3	85.5
Age & sex adjusted, using entire historic period	65.3	74.1	86.1	67.6	78.3	90.6
Unadjusted, using TaR after historic visit	64.7	73.0	83.8	65.0	73.1	84.4
Age & sex adjusted, using TaR after historic visit	65.8	75.5	89.1	65.8	75.5	89.1
Unadjusted, using entire historic period, filtered	63.5	70.6	82.2	66.2	73.4	85.5
Age & sex adjusted, using entire historic period, filtered	65.4	74.0	85.7	68.0	78.5	90.5
Unadjusted, using TaR after historic visit, filtered	64.8	73.0	83.4	65.3	73.4	84.1
Age & sex adjusted, using TaR after historic visit, filtered	66.0	75.6	89.0	66.2	75.5	88.9
SCCS / SCRI						
Unadjusted SCCS excluding pre−vaccination window	73.2	83.1	91.9	73.5	84.0	92.8
Age & season adjusted SCCS excluding pre−vaccination window	73.2	83.7	92.4	73.2	83.7	92.4
SCRI with prior control interval	67.3	76.6	87.1	69.2	79.1	90.0
SCRI with posterior control interval	66.9	76.4	87.7	69.1	78.4	90.3
Unadjusted SCCS excluding all pre−vaccination time	71.5	81.2	89.7	73.0	83.1	92.1

Figure 82: The area under the receiver operator curve (AUC) based on the effect-size estimate at the end of the study period in the Optum EHR database for Zoster vaccination (Shingrix).

	Uncalibrated AUC when true effect is			Calibrated AUC when true effect is		
	1.5	2	4	1.5	2	4
**Case−control**						
Age & sex matched controls	54.6	56.3	58.6	56.1	60.0	62.3
Age & sex adjusted, using random controls	56.8	58.4	61.2	62.7	68.3	73.1
**Cohort method**						
Unadjusted, using outpatient visits as comparator	55.7	60.0	69.8	55.7	61.2	69.8
PS matching, using outpatient visits as comparator	57.8	60.2	64.4	54.8	57.7	60.9
Unadjusted, using random days as comparator	55.6	57.0	59.8	56.5	59.6	66.2
PS matching, using random days as comparator	58.1	62.7	70.5	56.4	63.5	66.4
PS stratification, using outpatient visits as comparator	62.0	68.8	83.9	61.7	67.8	72.9
PS stratification, using random days as comparator	59.8	64.9	78.3	61.2	67.6	73.6
PS weighting, using outpatient visits as comparator	59.5	63.3	77.3	57.8	62.4	66.8
PS weighting, using random days as comparator	58.6	62.9	77.5	57.7	62.0	68.3
Per−month PS matching, using outpatient visits as comparator						
Per−month PS matching, using random days as comparator						
**Historical comparator**						
Unadjusted, using entire historic period	54.4	57.7	61.9	57.5	62.4	69.7
Age & sex adjusted, using entire historic period	58.6	62.1	65.6	65.4	72.5	78.6
Unadjusted, using TaR after historic visit	56.2	59.9	64.5	60.3	66.1	73.9
Age & sex adjusted, using TaR after historic visit	61.8	67.2	74.4	62.3	68.2	76.0
Unadjusted, using entire historic period, filtered	54.4	57.7	61.8	57.6	62.0	69.8
Age & sex adjusted, using entire historic period, filtered	58.7	62.1	65.5	64.5	70.6	75.1
Unadjusted, using TaR after historic visit, filtered	56.2	59.9	64.4	59.4	65.2	72.4
Age & sex adjusted, using TaR after historic visit, filtered	61.9	67.3	74.4	61.9	67.3	74.4
**SCCS / SCRI**						
Unadjusted SCCS excluding pre−vaccination window	64.2	68.4	72.3	65.3	69.4	73.9
Age & season adjusted SCCS excluding pre−vaccination window	63.0	67.8	71.7	64.1	69.9	73.8
SCRI with prior control interval	60.0	64.3	69.4	57.4	60.7	65.9
SCRI with posterior control interval	56.6	62.7	68.7	58.5	63.0	69.1
Unadjusted SCCS excluding all pre−vaccination time	60.9	65.4	69.5	62.2	67.7	72.4

Figure 83: The area under the receiver operator curve (AUC) based on the effect-size estimate at the end of the study period in the MDCD database for Zoster vaccination (Shingrix).

	Uncalibrated AUC when true effect is			Calibrated AUC when true effect is		
	1.5	2	4	1.5	2	4
Case−control						
Age & sex matched controls	58.3	64.1	71.8	58.3	64.1	71.8
Age & sex adjusted, using random controls	61.4	67.5	80.2	61.4	67.5	80.2
Cohort method						
Unadjusted, using outpatient visits as comparator	57.6	64.8	78.4	59.1	64.5	73.4
PS matching, using outpatient visits as comparator	61.1	68.1	79.2	61.3	69.0	78.5
Unadjusted, using random days as comparator	59.2	65.2	74.9	59.9	65.7	75.3
PS matching, using random days as comparator	63.6	69.7	78.4	62.4	68.9	76.0
PS stratification, using outpatient visits as comparator	62.6	70.0	86.0	59.4	66.4	77.5
PS stratification, using random days as comparator	63.6	69.3	84.2	63.4	69.6	79.5
PS weighting, using outpatient visits as comparator	63.3	68.7	85.9	56.4	62.2	71.9
PS weighting, using random days as comparator	62.6	69.0	84.0	63.3	70.1	79.7
Per−month PS matching, using outpatient visits as comparator						
Per−month PS matching, using random days as comparator						
Historical comparator						
Unadjusted, using entire historic period	61.3	66.6	78.2	60.4	66.9	78.5
Age & sex adjusted, using entire historic period	61.4	66.9	78.7	61.1	67.9	79.0
Unadjusted, using TaR after historic visit	59.8	65.9	77.2	59.2	64.3	75.9
Age & sex adjusted, using TaR after historic visit	60.2	66.1	78.1	59.6	65.5	76.8
Unadjusted, using entire historic period, filtered	61.6	66.9	78.3	61.6	66.5	77.9
Age & sex adjusted, using entire historic period, filtered	61.7	67.2	78.7	61.7	67.2	78.7
Unadjusted, using TaR after historic visit, filtered	60.1	66.4	77.7	59.1	65.0	75.9
Age & sex adjusted, using TaR after historic visit, filtered	60.6	66.6	78.6	59.5	65.1	76.8
SCCS / SCRI						
Unadjusted SCCS excluding pre−vaccination window	61.9	70.0	79.3	63.3	71.7	80.9
Age & season adjusted SCCS excluding pre−vaccination window	61.9	69.6	78.1	63.4	71.1	79.7
SCRI with prior control interval	59.0	64.0	74.2	62.0	66.7	77.9
SCRI with posterior control interval	60.5	68.7	74.5	62.6	70.8	77.6
Unadjusted SCCS excluding all pre−vaccination time	65.0	71.0	79.1	62.9	71.4	79.8

Figure 84: The area under the receiver operator curve (AUC) based on the effect-size estimate at the end of the study period in the MDCR database for Zoster vaccination (Shingrix).

	Uncalibrated AUC when true effect is			Calibrated AUC when true effect is		
	1.5	2	4	1.5	2	4
Case−control						
Age & sex matched controls	60.5	67.2	76.8	63.3	69.8	80.9
Age & sex adjusted, using random controls	64.8	73.9	84.2	65.1	74.5	85.4
Cohort method						
Unadjusted, using outpatient visits as comparator	62.5	70.3	84.7	62.2	69.8	82.3
PS matching, using outpatient visits as comparator	66.9	74.8	86.2	65.9	73.8	84.3
Unadjusted, using random days as comparator	59.4	63.3	74.0	61.3	65.5	75.3
PS matching, using random days as comparator	70.1	78.6	87.5	68.1	77.8	84.5
PS stratification, using outpatient visits as comparator	67.7	78.4	93.1	67.8	76.9	88.2
PS stratification, using random days as comparator	68.7	77.1	88.9	69.5	78.2	88.3
PS weighting, using outpatient visits as comparator	68.7	78.6	93.4	68.8	77.7	88.8
PS weighting, using random days as comparator	67.6	76.4	89.0	68.4	77.5	87.6
Per−month PS matching, using outpatient visits as comparator						
Per−month PS matching, using random days as comparator						
Historical comparator						
Unadjusted, using entire historic period	62.7	69.7	80.9	63.7	72.2	83.7
Age & sex adjusted, using entire historic period	69.5	77.9	87.8	69.7	78.9	89.0
Unadjusted, using TaR after historic visit	64.9	73.0	83.3	65.3	73.8	84.5
Age & sex adjusted, using TaR after historic visit	68.2	77.3	89.4	68.2	76.6	87.9
Unadjusted, using entire historic period, filtered	62.8	69.8	80.5	64.1	72.6	83.4
Age & sex adjusted, using entire historic period, filtered	69.9	78.4	87.5	70.1	79.4	88.9
Unadjusted, using TaR after historic visit, filtered	65.3	73.4	83.0	65.3	73.8	83.7
Age & sex adjusted, using TaR after historic visit, filtered	68.7	78.0	89.6	67.9	76.2	87.2
SCCS / SCRI						
Unadjusted SCCS excluding pre−vaccination window	70.1	79.8	89.5	70.1	79.8	90.1
Age & season adjusted SCCS excluding pre−vaccination window	69.7	79.6	88.8	69.9	79.4	89.0
SCRI with prior control interval	67.3	74.5	83.0	68.6	76.0	84.6
SCRI with posterior control interval	63.6	69.4	80.8	65.6	71.6	83.8
Unadjusted SCCS excluding all pre−vaccination time	66.8	75.4	84.6	68.1	77.3	86.6

Figure 85: The area under the receiver operator curve (AUC) based on the effect-size estimate at the end of the study period in the CCAE database for Zoster vaccination (Shingrix).

12.6 AUC for HPV vaccination (Gardasil 9)

	Uncalibrated AUC when true effect is			Calibrated AUC when true effect is		
	1.5	2	4	1.5	2	4
Case−control						
Age & sex matched controls	56.9	59.5	63.3	55.8	58.4	67.7
Age & sex adjusted, using random controls	55.0	58.1	63.7	56.2	59.9	66.7
Cohort method						
Unadjusted, using outpatient visits as comparator	63.4	70.2	81.8	54.3	58.9	67.9
PS matching, using outpatient visits as comparator	64.5	69.8	77.9	61.9	67.7	72.9
Unadjusted, using random days as comparator	60.3	65.1	75.4	58.6	61.7	72.4
PS matching, using random days as comparator						
PS stratification, using outpatient visits as comparator	67.6	77.4	93.7	64.5	70.4	78.3
PS stratification, using random days as comparator						
PS weighting, using outpatient visits as comparator	63.9	70.9	86.3	61.6	67.1	74.4
PS weighting, using random days as comparator						
Per−month PS matching, using outpatient visits as comparator						
Per−month PS matching, using random days as comparator						
Historical comparator						
Unadjusted, using entire historic period	57.3	61.4	74.3	56.5	60.6	71.9
Age & sex adjusted, using entire historic period	60.4	64.5	70.6	62.4	70.6	79.5
Unadjusted, using TaR after historic visit	56.4	61.2	69.4	56.0	59.5	70.7
Age & sex adjusted, using TaR after historic visit	62.9	70.7	79.9	63.7	70.3	79.1
Unadjusted, using entire historic period, filtered	57.2	61.3	74.2	56.4	60.4	71.7
Age & sex adjusted, using entire historic period, filtered	60.4	64.5	70.4	62.9	70.4	78.3
Unadjusted, using TaR after historic visit, filtered	55.9	60.6	68.9	55.9	59.8	70.5
Age & sex adjusted, using TaR after historic visit, filtered	63.0	70.9	80.0	64.3	70.5	78.7
SCCS / SCRI						
Unadjusted SCCS excluding pre−vaccination window	62.1	68.8	75.4	64.5	70.0	77.8
Age & season adjusted SCCS excluding pre−vaccination window	62.2	69.0	76.0	63.1	69.4	77.2
SCRI with prior control interval	60.4	62.4	67.1	61.6	64.7	69.6
SCRI with posterior control interval	60.9	64.2	70.3	62.7	67.5	73.7
Unadjusted SCCS excluding all pre−vaccination time	62.8	67.8	73.8	63.2	69.9	77.6

Figure 86: The area under the receiver operator curve (AUC) based on the effect-size estimate at the end of the study period in the Optum EHR database for HPV vaccination (Gardasil 9).

	Uncalibrated AUC when true effect is			Calibrated AUC when true effect is		
	1.5	2	4	1.5	2	4
Case−control						
Age & sex matched controls	54.0	56.8	59.0	61.9	66.1	70.2
Age & sex adjusted, using random controls	57.5	61.3	63.9	65.0	69.9	73.7
Cohort method						
Unadjusted, using outpatient visits as comparator	62.7	65.8	74.8	55.4	59.2	64.9
PS matching, using outpatient visits as comparator	58.5	62.6	67.7	57.7	61.8	67.3
Unadjusted, using random days as comparator	61.1	64.9	72.5	55.7	58.4	66.3
PS matching, using random days as comparator	60.9	64.1	68.7	57.9	60.2	67.5
PS stratification, using outpatient visits as comparator	62.5	70.5	86.9	61.0	64.4	72.4
PS stratification, using random days as comparator	60.6	65.9	80.2	58.7	64.3	71.6
PS weighting, using outpatient visits as comparator	61.0	65.7	81.2	59.0	62.6	68.3
PS weighting, using random days as comparator	58.6	60.8	74.9	55.3	59.5	65.5
Per−month PS matching, using outpatient visits as comparator						
Per−month PS matching, using random days as comparator						
Historical comparator						
Unadjusted, using entire historic period	58.3	61.0	68.0	55.3	59.0	65.5
Age & sex adjusted, using entire historic period	61.9	66.6	71.7	63.4	70.5	77.6
Unadjusted, using TaR after historic visit	57.4	62.1	68.6	55.4	58.1	65.6
Age & sex adjusted, using TaR after historic visit	60.2	66.1	73.8	60.2	65.6	73.8
Unadjusted, using entire historic period, filtered	58.4	61.0	67.8	56.3	58.9	65.8
Age & sex adjusted, using entire historic period, filtered	62.1	66.8	71.7	65.3	70.5	76.9
Unadjusted, using TaR after historic visit, filtered	56.8	61.5	67.9	55.3	57.8	65.3
Age & sex adjusted, using TaR after historic visit, filtered	60.4	66.5	74.2	60.9	65.4	73.2
SCCS / SCRI						
Unadjusted SCCS excluding pre−vaccination window	61.8	66.7	70.7	63.3	69.8	74.8
Age & season adjusted SCCS excluding pre−vaccination window	62.3	67.4	71.4	65.4	71.1	75.5
SCRI with prior control interval	58.5	62.0	67.9	65.0	67.9	75.5
SCRI with posterior control interval	57.7	61.9	68.1	63.8	66.1	73.0
Unadjusted SCCS excluding all pre−vaccination time	60.2	64.5	69.5	62.9	68.2	74.8

Figure 87: The area under the receiver operator curve (AUC) based on the effect-size estimate at the end of the study period in the MDCD database for HPV vaccination (Gardasil 9).

	Uncalibrated AUC when true effect is			Calibrated AUC when true effect is		
	1.5	2	4	1.5	2	4
Case−control						
Age & sex matched controls						
Age & sex adjusted, using random controls						
Cohort method						
Unadjusted, using outpatient visits as comparator	50.0	50.0	50.0	50.0	50.0	50.0
PS matching, using outpatient visits as comparator						
Unadjusted, using random days as comparator						
PS matching, using random days as comparator						
PS stratification, using outpatient visits as comparator	50.0	50.0	52.1	50.0	50.0	50.0
PS stratification, using random days as comparator						
PS weighting, using outpatient visits as comparator	50.0	50.0	52.1	50.0	50.0	50.0
PS weighting, using random days as comparator						
Per−month PS matching, using outpatient visits as comparator						
Per−month PS matching, using random days as comparator						
Historical comparator						
Unadjusted, using entire historic period	50.0	50.0	50.0	50.0	50.0	50.0
Age & sex adjusted, using entire historic period	50.0	50.0	50.0	50.0	50.0	50.0
Unadjusted, using TaR after historic visit	50.0	50.0	50.0	50.0	50.0	50.0
Age & sex adjusted, using TaR after historic visit	50.0	50.0	50.0	50.0	50.0	50.0
Unadjusted, using entire historic period, filtered	50.0	50.0	50.0	50.0	50.0	50.0
Age & sex adjusted, using entire historic period, filtered	50.0	50.0	50.0	50.0	50.0	50.0
Unadjusted, using TaR after historic visit, filtered	50.0	50.0	50.0	50.0	50.0	50.0
Age & sex adjusted, using TaR after historic visit, filtered	50.0	50.0	50.0	50.0	50.0	50.0

Figure 88: The area under the receiver operator curve (AUC) based on the effect-size estimate at the end of the study period in the MDCR database for HPV vaccination (Gardasil 9).

	Uncalibrated AUC when true effect is			Calibrated AUC when true effect is		
	1.5	2	4	1.5	2	4
**Case−control**						
Age & sex matched controls	57.5	61.0	64.7	60.8	66.4	71.2
Age & sex adjusted, using random controls	57.7	62.2	66.4	58.9	65.0	71.5
**Cohort method**						
Unadjusted, using outpatient visits as comparator	62.6	68.1	76.4	55.5	58.8	68.3
PS matching, using outpatient visits as comparator	60.7	65.9	73.6	61.5	66.7	72.8
Unadjusted, using random days as comparator	61.3	66.8	78.0	56.6	62.0	72.6
PS matching, using random days as comparator	63.9	69.2	75.6	62.8	68.2	73.3
PS stratification, using outpatient visits as comparator	66.7	74.1	91.9	62.1	68.5	75.9
PS stratification, using random days as comparator	63.2	69.2	83.1	62.8	68.7	74.9
PS weighting, using outpatient visits as comparator	63.3	70.6	87.1	61.1	68.3	75.0
PS weighting, using random days as comparator	58.7	64.3	78.3	58.1	62.4	70.9
Per−month PS matching, using outpatient visits as comparator						
Per−month PS matching, using random days as comparator						
**Historical comparator**						
Unadjusted, using entire historic period	57.9	62.7	74.4	57.9	62.7	72.8
Age & sex adjusted, using entire historic period	64.1	69.7	75.5	65.3	73.0	80.0
Unadjusted, using TaR after historic visit	57.1	61.1	69.3	57.1	62.7	72.1
Age & sex adjusted, using TaR after historic visit	63.3	70.1	79.5	63.3	70.1	79.5
Unadjusted, using entire historic period, filtered	57.2	62.0	73.7	57.2	62.4	71.9
Age & sex adjusted, using entire historic period, filtered	64.3	69.8	75.1	66.0	73.2	79.3
Unadjusted, using TaR after historic visit, filtered	57.0	60.0	67.8	56.6	63.0	71.3
Age & sex adjusted, using TaR after historic visit, filtered	63.4	70.2	79.4	63.9	70.6	78.5
**SCCS / SCRI**						
Unadjusted SCCS excluding pre−vaccination window	63.5	69.3	74.7	63.9	70.5	76.3
Age & season adjusted SCCS excluding pre−vaccination window	63.7	69.9	75.4	63.3	70.3	76.2
SCRI with prior control interval	60.9	65.0	69.5	62.7	68.7	73.8
SCRI with posterior control interval	64.6	69.9	73.0	64.8	70.1	75.3
Unadjusted SCCS excluding all pre−vaccination time	62.5	67.7	72.5	63.0	71.2	76.0

Figure 89: The area under the receiver operator curve (AUC) based on the effect-size estimate at the end of the study period in the CCAE database for HPV vaccination (Gardasil 9).

13 Confidence-interval-based metrics

For each method variation and vaccine group, the coverage of the 95% confidence interval, (geometric) mean of the precision (1/SE^2), mean squared error (MSE), and fraction non-estimable, based on the negative control effect-size estimates at the end of the study period.

13.1 Confidence-interval-based metrics for H1N1 vaccination

	Uncalibrated				Calibrated			
	Coverage	Mean precision	MSE	Non estimable	Coverage	Mean precision	MSE	Non estimable
Case−control								
Age & sex matched controls	0.83	0.60	81.26	0.49	0.98	0.60	76.12	0.49
Age & sex adjusted, using random controls	0.81	0.81	0.79	0.47	1.00	0.81	0.27	0.47
Cohort method								
Unadjusted, using outpatient visits as comparator	0.80	1.70	1.11	0.34	0.96	1.11	0.41	0.34
PS matching, using outpatient visits as comparator	0.89	1.37	0.95	0.35	0.99	1.31	0.32	0.35
Unadjusted, using random days as comparator	0.78	0.73	0.82	0.52	1.00	0.72	0.23	0.52
PS matching, using random days as comparator	1.00	0.00	0.00	1.00	1.00	0.00	0.00	1.00
PS stratification, using outpatient visits as comparator	0.82	1.88	0.96	0.29	0.98	1.58	0.42	0.29
PS stratification, using random days as comparator	0.67	1.60	1.34	0.34	1.00	1.59	0.25	0.34
PS weighting, using outpatient visits as comparator	0.82	1.51	0.93	0.31	0.98	1.03	0.57	0.31
PS weighting, using random days as comparator	1.00	0.00	2.78	1.00	1.00	0.00	0.00	1.00
Per−month PS matching, using outpatient visits as comparator	0.97	0.69	0.97	0.48	0.98	0.61	0.20	0.48
Per−month PS matching, using random days as comparator	0.99	0.32	0.44	0.68	1.00	0.31	0.15	0.68
Historical comparator								
Unadjusted, using entire historic period	0.59	2.87	1.12	0.27	0.95	2.00	0.30	0.27
Age & sex adjusted, using entire historic period	0.59	2.86	1.30	0.27	0.94	2.05	0.31	0.27
Unadjusted, using TaR after historic visit	0.82	2.78	0.43	0.27	0.98	1.53	0.34	0.27
Age & sex adjusted, using TaR after historic visit	0.80	2.77	0.50	0.27	0.96	1.92	0.31	0.27
Unadjusted, using entire historic period, filtered	0.61	2.69	0.99	0.31	0.96	1.92	0.28	0.31
Age & sex adjusted, using entire historic period, filtered	0.61	2.69	1.15	0.31	0.95	1.97	0.28	0.31
Unadjusted, using TaR after historic visit, filtered	0.83	2.62	0.39	0.31	0.98	1.44	0.32	0.31
Age & sex adjusted, using TaR after historic visit, filtered	0.81	2.60	0.44	0.31	0.97	1.82	0.29	0.31
SCCS / SCRI								
Unadjusted SCCS excluding pre−vaccination window	0.95	2.55	0.26	0.28	0.98	2.14	0.26	0.28
Age & season adjusted SCCS excluding pre−vaccination window	0.96	2.41	0.26	0.29	0.99	2.39	0.25	0.29
SCRI with prior control interval	0.97	1.52	0.25	0.33	0.98	1.48	0.25	0.33
SCRI with posterior control interval	0.97	1.45	4.52	0.38	0.99	1.42	4.56	0.38
Unadjusted SCCS excluding all pre−vaccination time	0.95	2.29	4.50	0.29	0.99	1.70	0.35	0.29

Figure 90: The coverage, mean precision, mean squared error (MSE), and fraction non-estimable based on the negative control effect-size estimate at the end of the study period in the Optum EHR database for H1N1 vaccination.

	Uncalibrated				Calibrated			
Case−control	Coverage	Mean precision	MSE	Non estimable	Coverage	Mean precision	MSE	Non estimable
Age & sex matched controls	0.88	1.48	15.22	0.42	0.99	1.18	0.37	0.42
Age & sex adjusted, using random controls	0.97	1.90	0.28	0.37	0.99	1.73	0.23	0.37
Cohort method								
Unadjusted, using outpatient visits as comparator	0.48	1.90	3.17	0.35	0.97	0.44	0.74	0.35
PS matching, using outpatient visits as comparator	0.92	1.10	0.71	0.48	0.99	0.88	0.20	0.48
Unadjusted, using random days as comparator	0.73	1.54	1.12	0.42	0.98	0.64	0.40	0.42
PS matching, using random days as comparator	0.97	1.05	0.51	0.49	0.98	1.02	0.20	0.49
PS stratification, using outpatient visits as comparator	0.91	1.56	0.67	0.38	0.96	1.19	0.34	0.38
PS stratification, using random days as comparator	0.95	1.51	0.50	0.38	0.97	1.31	0.31	0.38
PS weighting, using outpatient visits as comparator	0.92	1.18	0.65	0.40	0.96	1.12	0.36	0.40
PS weighting, using random days as comparator	0.98	0.55	1.16	0.39	1.00	0.54	1.02	0.39
Per−month PS matching, using outpatient visits as comparator	0.97	0.99	0.52	0.48	0.98	0.97	0.15	0.48
Per−month PS matching, using random days as comparator	0.99	0.98	0.61	0.49	0.99	0.97	0.22	0.49
Historical comparator								
Unadjusted, using entire historic period	0.62	2.44	0.86	0.33	0.98	0.78	0.51	0.33
Age & sex adjusted, using entire historic period	0.92	2.43	0.41	0.33	0.96	1.47	0.38	0.33
Unadjusted, using TaR after historic visit	0.54	2.35	1.31	0.33	0.98	0.65	0.62	0.33
Age & sex adjusted, using TaR after historic visit	0.91	2.28	0.40	0.33	0.97	1.50	0.40	0.33
Unadjusted, using entire historic period, filtered	0.62	2.40	0.85	0.34	0.98	0.78	0.48	0.34
Age & sex adjusted, using entire historic period, filtered	0.94	2.39	0.34	0.34	0.97	1.49	0.31	0.34
Unadjusted, using TaR after historic visit, filtered	0.54	2.32	1.30	0.34	0.98	0.64	0.59	0.34
Age & sex adjusted, using TaR after historic visit, filtered	0.92	2.25	0.31	0.34	0.97	1.50	0.31	0.34
SCCS / SCRI								
Unadjusted SCCS excluding pre−vaccination window	0.94	2.17	0.23	0.37	0.98	1.44	0.23	0.37
Age & season adjusted SCCS excluding pre−vaccination window	0.94	2.07	0.21	0.38	0.97	1.95	0.21	0.38
SCRI with prior control interval	0.99	1.30	14.02	0.42	0.99	0.95	0.22	0.42
SCRI with posterior control interval	0.97	1.30	14.04	0.42	1.00	1.24	0.23	0.42
Unadjusted SCCS excluding all pre−vaccination time	0.96	1.97	0.21	0.37	0.98	1.42	0.21	0.37

Figure 91: The coverage, mean precision, mean squared error (MSE), and fraction non-estimable based on the negative control effect-size estimate at the end of the study period in the MDCD database for H1N1 vaccination.

	Uncalibrated				Calibrated			
Case−control	Coverage	Mean precision	MSE	Non estimable	Coverage	Mean precision	MSE	Non estimable
Age & sex matched controls	0.92	0.46	17.76	0.63	0.98	0.44	17.12	0.63
Age & sex adjusted, using random controls	0.97	0.46	0.50	0.58	1.00	0.36	0.34	0.58
Cohort method								
Unadjusted, using outpatient visits as comparator	0.98	0.47	0.55	0.60	0.98	0.45	0.18	0.60
PS matching, using outpatient visits as comparator	1.00	0.22	0.51	0.76	1.00	0.22	0.11	0.76
Unadjusted, using random days as comparator	0.97	0.29	0.52	0.74	0.99	0.28	0.17	0.74
PS matching, using random days as comparator	1.00	0.22	0.29	0.74	1.00	0.22	0.13	0.74
PS stratification, using outpatient visits as comparator	0.96	0.47	0.72	0.58	0.98	0.46	0.36	0.58
PS stratification, using random days as comparator	0.99	0.37	0.69	0.66	0.99	0.36	0.43	0.66
PS weighting, using outpatient visits as comparator	0.86	0.58	1.26	0.58	0.99	0.14	0.88	0.58
PS weighting, using random days as comparator	1.00	0.13	0.91	0.66	1.00	0.13	0.61	0.66
Per−month PS matching, using outpatient visits as comparator	0.98	0.22	0.38	0.76	1.00	0.21	0.16	0.76
Per−month PS matching, using random days as comparator	1.00	0.21	0.42	0.74	1.00	0.21	0.07	0.74
Historical comparator								
Unadjusted, using entire historic period	0.92	0.79	0.72	0.54	0.95	0.54	0.43	0.54
Age & sex adjusted, using entire historic period	0.92	0.79	0.72	0.54	0.95	0.56	0.43	0.54
Unadjusted, using TaR after historic visit	0.92	0.78	0.61	0.54	0.95	0.58	0.48	0.54
Age & sex adjusted, using TaR after historic visit	0.94	0.78	0.59	0.54	0.94	0.59	0.45	0.54
Unadjusted, using entire historic period, filtered	0.94	0.78	0.59	0.55	0.96	0.55	0.36	0.55
Age & sex adjusted, using entire historic period, filtered	0.94	0.78	0.59	0.55	0.96	0.55	0.35	0.55
Unadjusted, using TaR after historic visit, filtered	0.94	0.77	0.47	0.55	0.95	0.58	0.38	0.55
Age & sex adjusted, using TaR after historic visit, filtered	0.95	0.77	0.47	0.55	0.95	0.59	0.37	0.55
SCCS / SCRI								
Unadjusted SCCS excluding pre−vaccination window	0.96	0.68	0.24	0.57	1.00	0.66	0.22	0.57
Age & season adjusted SCCS excluding pre−vaccination window	0.97	0.68	0.22	0.57	0.99	0.67	0.20	0.57
SCRI with prior control interval	1.00	0.35	13.96	0.65	1.00	0.35	0.14	0.65
SCRI with posterior control interval	0.98	0.41	0.17	0.61	0.98	0.41	0.17	0.61
Unadjusted SCCS excluding all pre−vaccination time	0.96	0.62	15.75	0.57	0.98	0.60	15.65	0.57

Figure 92: The coverage, mean precision, mean squared error (MSE), and fraction non-estimable based on the negative control effect-size estimate at the end of the study period in the MDCR database for H1N1 vaccination.

Case−control	Uncalibrated Coverage	Uncalibrated Mean precision	Uncalibrated MSE	Uncalibrated Non estimable	Calibrated Coverage	Calibrated Mean precision	Calibrated MSE	Calibrated Non estimable
Age & sex matched controls	0.88	1.61	29.95	0.42	0.98	1.23	29.33	0.42
Age & sex adjusted, using random controls	0.88	1.87	0.31	0.39	0.99	1.38	0.24	0.39
Cohort method								
Unadjusted, using outpatient visits as comparator	0.39	6.32	1.67	0.20	0.96	1.13	0.51	0.20
PS matching, using outpatient visits as comparator	0.77	4.50	0.46	0.22	0.97	2.87	0.34	0.22
Unadjusted, using random days as comparator	0.80	4.38	0.32	0.20	0.97	2.03	0.31	0.20
PS matching, using random days as comparator	0.92	4.19	0.32	0.23	0.95	3.60	0.32	0.23
PS stratification, using outpatient visits as comparator	0.70	6.25	0.39	0.19	0.96	3.95	0.22	0.19
PS stratification, using random days as comparator	0.90	5.52	0.26	0.20	0.95	3.91	0.25	0.20
PS weighting, using outpatient visits as comparator	0.55	5.59	0.82	0.19	0.99	2.07	0.37	0.19
PS weighting, using random days as comparator	0.96	2.07	0.38	0.20	0.97	2.02	0.34	0.20
Per−month PS matching, using outpatient visits as comparator	0.81	4.16	0.47	0.24	0.99	3.00	0.30	0.24
Per−month PS matching, using random days as comparator	0.95	3.92	0.30	0.26	0.97	3.34	0.21	0.26
Historical comparator								
Unadjusted, using entire historic period	0.80	8.60	0.19	0.19	0.96	3.02	0.19	0.19
Age & sex adjusted, using entire historic period	0.74	8.59	0.22	0.19	0.97	3.46	0.16	0.19
Unadjusted, using TaR after historic visit	0.61	8.35	0.33	0.19	0.96	2.15	0.24	0.19
Age & sex adjusted, using TaR after historic visit	0.82	8.27	0.15	0.19	0.97	3.89	0.14	0.19
Unadjusted, using entire historic period, filtered	0.80	8.44	0.18	0.20	0.96	2.96	0.18	0.20
Age & sex adjusted, using entire historic period, filtered	0.74	8.43	0.21	0.20	0.97	3.42	0.15	0.20
Unadjusted, using TaR after historic visit, filtered	0.61	8.20	0.33	0.20	0.96	2.11	0.23	0.20
Age & sex adjusted, using TaR after historic visit, filtered	0.82	8.12	0.14	0.20	0.97	3.82	0.14	0.20
SCCS / SCRI								
Unadjusted SCCS excluding pre−vaccination window	0.89	7.96	0.15	0.19	0.98	3.82	0.15	0.19
Age & season adjusted SCCS excluding pre−vaccination window	0.91	7.13	0.14	0.22	0.96	5.20	0.14	0.22
SCRI with prior control interval	0.90	4.72	0.22	0.20	0.99	2.83	0.22	0.20
SCRI with posterior control interval	0.91	4.55	15.08	0.22	0.98	3.54	0.23	0.22
Unadjusted SCCS excluding all pre−vaccination time	0.87	7.06	0.15	0.19	0.97	3.69	0.15	0.19

Figure 93: The coverage, mean precision, mean squared error (MSE), and fraction non-estimable based on the negative control effect-size estimate at the end of the study period in the CCAE database for H1N1 vaccination.

13.2 Confidence-interval-based metrics for Seasonal flu vaccination (Fluvirin)

	Uncalibrated				Calibrated			
	Coverage	Mean precision	MSE	Non estimable	Coverage	Mean precision	MSE	Non estimable
Case−control								
Age & sex matched controls	0.97	0.10	33.76	0.82	1.00	0.10	31.89	0.82
Age & sex adjusted, using random controls	0.98	0.06	0.32	0.87	1.00	0.06	0.06	0.87
Cohort method								
Unadjusted, using outpatient visits as comparator	0.95	0.38	1.45	0.60	0.98	0.37	0.40	0.60
PS matching, using outpatient visits as comparator	0.99	0.08	0.58	0.85	1.00	0.08	0.09	0.85
Unadjusted, using random days as comparator	0.99	0.23	0.57	0.70	1.00	0.23	0.10	0.70
PS matching, using random days as comparator								
PS stratification, using outpatient visits as comparator	0.96	0.33	1.02	0.54	0.97	0.32	0.50	0.54
PS stratification, using random days as comparator	0.95	0.44	0.99	0.57	0.98	0.44	0.36	0.57
PS weighting, using outpatient visits as comparator	0.98	0.19	0.91	0.57	1.00	0.19	0.35	0.57
PS weighting, using random days as comparator	1.00	0.00	0.00	1.00	1.00	0.00	0.00	1.00
Per−month PS matching, using outpatient visits as comparator								
Per−month PS matching, using random days as comparator								
Historical comparator								
Unadjusted, using entire historic period	0.86	0.70	1.09	0.53	0.96	0.52	0.34	0.53
Age & sex adjusted, using entire historic period	0.87	0.70	1.00	0.53	0.97	0.54	0.32	0.53
Unadjusted, using TaR after historic visit	0.92	0.70	0.51	0.53	0.97	0.55	0.37	0.53
Age & sex adjusted, using TaR after historic visit	0.94	0.70	0.52	0.53	0.97	0.53	0.39	0.53
Unadjusted, using entire historic period, filtered	0.87	0.67	1.02	0.54	0.96	0.57	0.32	0.54
Age & sex adjusted, using entire historic period, filtered	0.88	0.67	0.94	0.54	0.96	0.57	0.31	0.54
Unadjusted, using TaR after historic visit, filtered	0.94	0.67	0.48	0.54	0.97	0.56	0.35	0.54
Age & sex adjusted, using TaR after historic visit, filtered	0.95	0.67	0.49	0.54	0.97	0.54	0.38	0.54
SCCS / SCRI								
Unadjusted SCCS excluding pre−vaccination window	0.99	0.51	0.17	0.62	1.00	0.51	0.15	0.62
Age & season adjusted SCCS excluding pre−vaccination window	0.99	0.47	0.18	0.65	1.00	0.46	0.14	0.65
SCRI with prior control interval	1.00	0.21	39.95	0.74	1.00	0.20	4.63	0.74
SCRI with posterior control interval	1.00	0.26	13.95	0.69	1.00	0.26	0.13	0.69
Unadjusted SCCS excluding all pre−vaccination time	0.99	0.47	0.26	0.62	1.00	0.46	0.17	0.62

Figure 94: The coverage, mean precision, mean squared error (MSE), and fraction non-estimable based on the negative control effect-size estimate at the end of the study period in the Optum EHR database for Seasonal flu vaccination (Fluvirin).

Case−control	Uncalibrated Coverage	Uncalibrated Mean precision	Uncalibrated MSE	Uncalibrated Non estimable	Calibrated Coverage	Calibrated Mean precision	Calibrated MSE	Calibrated Non estimable
Age & sex matched controls	0.90	0.47	0.34	0.72	0.99	0.26	0.27	0.72
Age & sex adjusted, using random controls	1.00	0.35	0.26	0.67	1.00	0.35	0.16	0.67
Cohort method								
Unadjusted, using outpatient visits as comparator	0.97	0.41	1.07	0.68	0.99	0.41	0.15	0.68
PS matching, using outpatient visits as comparator	1.00	0.00	0.00	1.00	1.00	0.00	0.00	1.00
Unadjusted, using random days as comparator	0.95	0.22	0.51	0.75	0.98	0.21	0.25	0.75
PS matching, using random days as comparator	1.00	0.00	0.00	1.00	1.00	0.00	0.00	1.00
PS stratification, using outpatient visits as comparator	0.96	0.60	0.92	0.62	0.98	0.59	0.26	0.62
PS stratification, using random days as comparator	0.96	0.51	0.81	0.63	0.99	0.50	0.22	0.63
PS weighting, using outpatient visits as comparator	0.99	0.18	0.75	0.62	1.00	0.18	0.26	0.62
PS weighting, using random days as comparator	1.00	0.00	0.72	1.00	1.00	0.00	0.00	1.00
Per−month PS matching, using outpatient visits as comparator								
Per−month PS matching, using random days as comparator								
Historical comparator								
Unadjusted, using entire historic period	0.92	0.70	0.49	0.61	0.99	0.28	0.29	0.61
Age & sex adjusted, using entire historic period	0.97	0.70	0.28	0.61	0.99	0.31	0.23	0.61
Unadjusted, using TaR after historic visit	0.96	0.70	0.31	0.61	0.99	0.30	0.26	0.61
Age & sex adjusted, using TaR after historic visit	0.97	0.69	0.23	0.61	0.99	0.32	0.23	0.61
Unadjusted, using entire historic period, filtered	0.94	0.65	0.29	0.62	1.00	0.58	0.15	0.62
Age & sex adjusted, using entire historic period, filtered	0.98	0.65	0.14	0.62	0.98	0.62	0.11	0.62
Unadjusted, using TaR after historic visit, filtered	0.97	0.65	0.16	0.62	0.99	0.64	0.14	0.62
Age & sex adjusted, using TaR after historic visit, filtered	0.98	0.65	0.14	0.62	0.98	0.61	0.13	0.62
SCCS / SCRI								
Unadjusted SCCS excluding pre−vaccination window	0.97	0.63	0.14	0.61	0.98	0.63	0.14	0.61
Age & season adjusted SCCS excluding pre−vaccination window	0.97	0.60	0.14	0.62	0.98	0.59	0.13	0.62
SCRI with prior control interval	0.99	0.30	51.47	0.75	1.00	0.29	0.17	0.75
SCRI with posterior control interval	0.98	0.39	4.09	0.65	0.99	0.38	4.11	0.65
Unadjusted SCCS excluding all pre−vaccination time	0.98	0.61	3.94	0.61	0.99	0.60	3.96	0.61

Figure 95: The coverage, mean precision, mean squared error (MSE), and fraction non-estimable based on the negative control effect-size estimate at the end of the study period in the MDCD database for Seasonal flu vaccination (Fluvirin).

Case−control	Uncalibrated Coverage	Uncalibrated Mean precision	Uncalibrated MSE	Uncalibrated Non estimable	Calibrated Coverage	Calibrated Mean precision	Calibrated MSE	Calibrated Non estimable
Age & sex matched controls	1.00	0.02	4.46	0.96	1.00	0.00	0.00	1.00
Age & sex adjusted, using random controls	1.00	0.02	0.06	0.95	1.00	0.00	0.00	1.00
Cohort method								
Unadjusted, using outpatient visits as comparator	1.00	0.02	0.61	0.96	1.00	0.00	0.00	1.00
PS matching, using outpatient visits as comparator	1.00	0.00	0.29	0.99	1.00	0.00	0.00	1.00
Unadjusted, using random days as comparator	1.00	0.02	0.06	0.95	1.00	0.00	0.00	1.00
PS matching, using random days as comparator	1.00	0.00	0.13	0.99	1.00	0.00	0.00	1.00
PS stratification, using outpatient visits as comparator	1.00	0.03	0.56	0.92	1.00	0.03	0.10	0.92
PS stratification, using random days as comparator	1.00	0.02	0.29	0.96	1.00	0.00	0.00	1.00
PS weighting, using outpatient visits as comparator	0.99	0.02	0.72	0.92	1.00	0.02	0.22	0.92
PS weighting, using random days as comparator	1.00	0.00	0.36	0.96	1.00	0.00	0.00	1.00
Per−month PS matching, using outpatient visits as comparator								
Per−month PS matching, using random days as comparator								
Historical comparator								
Unadjusted, using entire historic period	0.99	0.06	0.22	0.91	1.00	0.06	0.09	0.91
Age & sex adjusted, using entire historic period	1.00	0.06	0.22	0.91	1.00	0.06	0.08	0.91
Unadjusted, using TaR after historic visit	1.00	0.06	0.19	0.91	1.00	0.06	0.08	0.91
Age & sex adjusted, using TaR after historic visit	1.00	0.06	0.19	0.91	1.00	0.06	0.08	0.91
Unadjusted, using entire historic period, filtered	0.99	0.06	0.22	0.91	1.00	0.06	0.09	0.91
Age & sex adjusted, using entire historic period, filtered	1.00	0.06	0.22	0.91	1.00	0.06	0.08	0.91
Unadjusted, using TaR after historic visit, filtered	1.00	0.06	0.19	0.91	1.00	0.06	0.08	0.91
Age & sex adjusted, using TaR after historic visit, filtered	1.00	0.06	0.19	0.91	1.00	0.06	0.08	0.91
SCCS / SCRI								
Unadjusted SCCS excluding pre−vaccination window	1.00	0.03	0.06	0.96	1.00	0.00	0.00	1.00
Age & season adjusted SCCS excluding pre−vaccination window	1.00	0.03	0.06	0.96	1.00	0.00	0.00	1.00
SCRI with prior control interval	1.00	0.01	0.02	0.97	1.00	0.00	0.00	1.00
SCRI with posterior control interval	1.00	0.01	3.81	0.97	1.00	0.00	0.00	1.00
Unadjusted SCCS excluding all pre−vaccination time	1.00	0.02	3.94	0.97	1.00	0.00	0.00	1.00

Figure 96: The coverage, mean precision, mean squared error (MSE), and fraction non-estimable based on the negative control effect-size estimate at the end of the study period in the MDCR database for Seasonal flu vaccination (Fluvirin).

Case−control	Uncalibrated Coverage	Uncalibrated Mean precision	Uncalibrated MSE	Uncalibrated Non estimable	Calibrated Coverage	Calibrated Mean precision	Calibrated MSE	Calibrated Non estimable
Age & sex matched controls	1.00	0.61	0.28	0.57	1.00	0.61	0.22	0.57
Age & sex adjusted, using random controls	0.97	0.66	0.28	0.54	0.99	0.65	0.26	0.54
Cohort method								
Unadjusted, using outpatient visits as comparator	0.70	1.77	1.69	0.35	0.97	1.07	0.42	0.35
PS matching, using outpatient visits as comparator	0.95	1.03	0.96	0.45	0.98	1.01	0.22	0.45
Unadjusted, using random days as comparator	0.90	1.16	0.52	0.44	0.99	1.00	0.26	0.44
PS matching, using random days as comparator	0.99	1.28	0.70	0.38	1.00	1.27	0.23	0.38
PS stratification, using outpatient visits as comparator	0.90	1.69	0.83	0.35	0.97	1.61	0.39	0.35
PS stratification, using random days as comparator	0.96	1.87	0.52	0.35	0.98	1.85	0.17	0.35
PS weighting, using outpatient visits as comparator	0.76	1.70	1.32	0.38	0.96	0.94	0.70	0.38
PS weighting, using random days as comparator	1.00	0.56	0.54	0.35	1.00	0.56	0.20	0.35
Per−month PS matching, using outpatient visits as comparator								
Per−month PS matching, using random days as comparator								
Historical comparator								
Unadjusted, using entire historic period	0.86	2.40	0.39	0.34	0.98	0.75	0.34	0.34
Age & sex adjusted, using entire historic period	0.95	2.39	0.31	0.34	0.98	0.84	0.30	0.34
Unadjusted, using TaR after historic visit	0.88	2.38	0.36	0.34	0.98	0.74	0.36	0.34
Age & sex adjusted, using TaR after historic visit	0.88	2.37	0.38	0.34	0.98	0.76	0.37	0.34
Unadjusted, using entire historic period, filtered	0.88	2.15	0.21	0.37	0.98	2.11	0.19	0.37
Age & sex adjusted, using entire historic period, filtered	0.97	2.15	0.17	0.37	0.97	2.12	0.18	0.37
Unadjusted, using TaR after historic visit, filtered	0.90	2.14	0.20	0.37	0.98	1.63	0.20	0.37
Age & sex adjusted, using TaR after historic visit, filtered	0.90	2.14	0.26	0.37	0.98	1.46	0.23	0.37
SCCS / SCRI								
Unadjusted SCCS excluding pre−vaccination window	0.96	2.08	0.26	0.34	0.98	2.04	0.25	0.34
Age & season adjusted SCCS excluding pre−vaccination window	0.97	1.74	0.25	0.38	0.98	1.73	0.24	0.38
SCRI with prior control interval	0.97	0.98	57.53	0.45	0.99	0.96	0.25	0.45
SCRI with posterior control interval	0.95	1.19	4.22	0.46	1.00	1.16	0.31	0.46
Unadjusted SCCS excluding all pre−vaccination time	0.95	1.95	0.30	0.35	0.97	1.83	0.28	0.35

Figure 97: The coverage, mean precision, mean squared error (MSE), and fraction non-estimable based on the negative control effect-size estimate at the end of the study period in the CCAE database for Seasonal flu vaccination (Fluvirin).

13.3 Confidence-interval-based metrics for Seasonal flu vaccination (Fluzone)

	Uncalibrated				Calibrated			
	Coverage	Mean precision	MSE	Non estimable	Coverage	Mean precision	MSE	Non estimable
Case−control								
Age & sex matched controls	0.81	1.64	48.65	0.30	0.99	1.27	46.63	0.30
Age & sex adjusted, using random controls	0.75	1.92	0.89	0.26	0.99	1.31	0.39	0.26
Cohort method								
Unadjusted, using outpatient visits as comparator	0.60	6.78	0.95	0.08	0.96	2.14	0.51	0.08
PS matching, using outpatient visits as comparator	0.82	3.06	0.80	0.20	0.97	2.78	0.34	0.20
Unadjusted, using random days as comparator	0.61	4.06	0.75	0.18	0.95	1.96	0.46	0.18
PS matching, using random days as comparator								
PS stratification, using outpatient visits as comparator	0.71	5.64	0.69	0.09	0.95	3.46	0.35	0.09
PS stratification, using random days as comparator	0.66	7.31	0.76	0.10	0.97	2.40	0.40	0.10
PS weighting, using outpatient visits as comparator	0.69	3.67	1.02	0.12	0.96	2.12	0.51	0.12
PS weighting, using random days as comparator	1.00	0.00	13.14	1.00	1.00	0.00	0.00	1.00
Per−month PS matching, using outpatient visits as comparator								
Per−month PS matching, using random days as comparator								
Historical comparator								
Unadjusted, using entire historic period	0.43	10.56	1.02	0.08	0.95	2.01	0.44	0.08
Age & sex adjusted, using entire historic period	0.45	10.55	0.86	0.08	0.95	2.05	0.40	0.08
Unadjusted, using TaR after historic visit	0.70	10.38	0.43	0.08	0.96	2.08	0.43	0.08
Age & sex adjusted, using TaR after historic visit	0.71	10.32	0.39	0.08	0.97	2.13	0.39	0.08
Unadjusted, using entire historic period, filtered	0.44	9.98	0.94	0.09	0.95	2.35	0.40	0.09
Age & sex adjusted, using entire historic period, filtered	0.46	9.98	0.79	0.09	0.95	2.38	0.36	0.09
Unadjusted, using TaR after historic visit, filtered	0.71	9.82	0.40	0.09	0.95	2.37	0.40	0.09
Age & sex adjusted, using TaR after historic visit, filtered	0.72	9.77	0.35	0.09	0.95	2.46	0.36	0.09
SCCS / SCRI								
Unadjusted SCCS excluding pre−vaccination window	0.80	8.63	0.30	0.08	0.97	3.74	0.27	0.08
Age & season adjusted SCCS excluding pre−vaccination window	0.77	7.00	0.33	0.13	0.97	2.89	0.29	0.13
SCRI with prior control interval	0.74	3.78	28.95	0.14	0.96	1.94	0.35	0.14
SCRI with posterior control interval	0.84	4.09	14.33	0.15	0.96	3.09	0.29	0.15
Unadjusted SCCS excluding all pre−vaccination time	0.76	7.37	0.38	0.08	0.95	3.97	0.30	0.08

Figure 98: The coverage, mean precision, mean squared error (MSE), and fraction non-estimable based on the negative control effect-size estimate at the end of the study period in the Optum EHR database for Seasonal flu vaccination (Fluzone).

	Uncalibrated				Calibrated			
Case−control	Coverage	Mean precision	MSE	Non estimable	Coverage	Mean precision	MSE	Non estimable
Age & sex matched controls	1.00	0.06	15.00	0.86	1.00	0.06	0.13	0.86
Age & sex adjusted, using random controls	0.99	0.05	0.16	0.91	1.00	0.05	0.06	0.91
Cohort method								
Unadjusted, using outpatient visits as comparator	1.00	0.07	0.63	0.88	1.00	0.07	0.03	0.88
PS matching, using outpatient visits as comparator	1.00	0.03	0.39	0.92	1.00	0.03	0.05	0.92
Unadjusted, using random days as comparator	0.99	0.05	0.30	0.91	1.00	0.04	0.08	0.91
PS matching, using random days as comparator								
PS stratification, using outpatient visits as comparator	0.99	0.12	0.79	0.80	1.00	0.12	0.30	0.80
PS stratification, using random days as comparator	0.99	0.16	0.57	0.81	1.00	0.16	0.09	0.81
PS weighting, using outpatient visits as comparator	1.00	0.07	0.62	0.80	1.00	0.07	0.21	0.80
PS weighting, using random days as comparator	1.00	0.00	0.00	1.00	1.00	0.00	0.00	1.00
Per−month PS matching, using outpatient visits as comparator								
Per−month PS matching, using random days as comparator								
Historical comparator								
Unadjusted, using entire historic period	0.90	0.27	1.24	0.75	1.00	0.14	0.35	0.75
Age & sex adjusted, using entire historic period	0.96	0.27	0.58	0.75	0.99	0.24	0.23	0.75
Unadjusted, using TaR after historic visit	0.91	0.27	0.90	0.75	1.00	0.16	0.31	0.75
Age & sex adjusted, using TaR after historic visit	0.97	0.27	0.32	0.75	1.00	0.25	0.23	0.75
Unadjusted, using entire historic period, filtered	0.91	0.26	1.07	0.76	0.99	0.15	0.31	0.76
Age & sex adjusted, using entire historic period, filtered	0.97	0.26	0.51	0.76	0.99	0.23	0.21	0.76
Unadjusted, using TaR after historic visit, filtered	0.92	0.25	0.76	0.76	1.00	0.17	0.27	0.76
Age & sex adjusted, using TaR after historic visit, filtered	0.98	0.25	0.28	0.76	0.99	0.25	0.21	0.76
SCCS / SCRI								
Unadjusted SCCS excluding pre−vaccination window	0.99	0.20	0.17	0.80	0.99	0.20	0.15	0.80
Age & season adjusted SCCS excluding pre−vaccination window	0.99	0.20	0.17	0.80	1.00	0.20	0.15	0.80
SCRI with prior control interval	0.99	0.08	43.71	0.86	1.00	0.08	0.12	0.86
SCRI with posterior control interval	1.00	0.11	0.07	0.84	1.00	0.10	0.07	0.84
Unadjusted SCCS excluding all pre−vaccination time	0.99	0.20	0.18	0.80	1.00	0.19	0.16	0.80

Figure 99: The coverage, mean precision, mean squared error (MSE), and fraction non-estimable based on the negative control effect-size estimate at the end of the study period in the MDCD database for Seasonal flu vaccination (Fluzone).

	Uncalibrated				Calibrated			
Case−control	Coverage	Mean precision	MSE	Non estimable	Coverage	Mean precision	MSE	Non estimable
Age & sex matched controls	0.95	0.90	0.28	0.53	0.97	0.86	0.26	0.53
Age & sex adjusted, using random controls	0.94	1.03	0.34	0.42	0.99	0.85	0.34	0.42
Cohort method								
Unadjusted, using outpatient visits as comparator	0.81	0.91	1.45	0.44	0.99	0.61	0.40	0.44
PS matching, using outpatient visits as comparator	0.98	0.52	0.93	0.60	1.00	0.51	0.15	0.60
Unadjusted, using random days as comparator	0.97	0.74	0.59	0.52	0.99	0.69	0.22	0.52
PS matching, using random days as comparator	0.99	0.61	0.70	0.52	0.99	0.60	0.21	0.52
PS stratification, using outpatient visits as comparator	0.94	0.92	0.87	0.39	0.97	0.91	0.34	0.39
PS stratification, using random days as comparator	0.96	0.93	0.78	0.41	0.98	0.88	0.36	0.41
PS weighting, using outpatient visits as comparator	0.91	0.73	1.00	0.43	0.99	0.72	0.40	0.43
PS weighting, using random days as comparator	1.00	0.28	1.31	0.41	0.99	0.28	0.95	0.42
Per−month PS matching, using outpatient visits as comparator								
Per−month PS matching, using random days as comparator								
Historical comparator								
Unadjusted, using entire historic period	0.91	1.39	0.49	0.38	0.97	0.70	0.49	0.38
Age & sex adjusted, using entire historic period	0.91	1.39	0.48	0.38	0.97	0.72	0.47	0.38
Unadjusted, using TaR after historic visit	0.88	1.36	0.61	0.38	0.97	0.64	0.60	0.38
Age & sex adjusted, using TaR after historic visit	0.89	1.35	0.59	0.38	0.96	0.66	0.59	0.38
Unadjusted, using entire historic period, filtered	0.94	1.28	0.40	0.40	0.96	1.20	0.40	0.40
Age & sex adjusted, using entire historic period, filtered	0.94	1.28	0.39	0.40	0.94	1.28	0.40	0.40
Unadjusted, using TaR after historic visit, filtered	0.90	1.25	0.51	0.40	0.95	0.91	0.49	0.40
Age & sex adjusted, using TaR after historic visit, filtered	0.91	1.25	0.49	0.40	0.96	0.94	0.48	0.40
SCCS / SCRI								
Unadjusted SCCS excluding pre−vaccination window	0.96	1.11	0.26	0.45	0.97	1.10	0.25	0.45
Age & season adjusted SCCS excluding pre−vaccination window	0.97	1.09	0.23	0.46	0.98	1.08	0.23	0.46
SCRI with prior control interval	0.97	0.49	44.75	0.60	0.98	0.46	0.23	0.60
SCRI with posterior control interval	0.95	0.66	15.20	0.54	1.00	0.63	0.29	0.54
Unadjusted SCCS excluding all pre−vaccination time	0.95	1.07	0.28	0.45	0.99	1.05	0.28	0.45

Figure 100: The coverage, mean precision, mean squared error (MSE), and fraction non-estimable based on the negative control effect-size estimate at the end of the study period in the MDCR database for Seasonal flu vaccination (Fluzone).

	Uncalibrated				Calibrated			
Case−control	Coverage	Mean precision	MSE	Non estimable	Coverage	Mean precision	MSE	Non estimable
Age & sex matched controls	1.00	0.00	0.00	1.00	1.00	0.00	0.00	1.00
Age & sex adjusted, using random controls	1.00	0.00	0.00	1.00	1.00	0.00	0.00	1.00
Cohort method								
Unadjusted, using outpatient visits as comparator	1.00	0.00	0.29	0.99	1.00	0.00	0.00	1.00
PS matching, using outpatient visits as comparator	1.00	0.01	0.18	0.98	1.00	0.00	0.00	1.00
Unadjusted, using random days as comparator	1.00	0.00	0.20	1.00	1.00	0.00	0.00	1.00
PS matching, using random days as comparator	1.00	0.02	0.28	0.96	1.00	0.00	0.00	1.00
PS stratification, using outpatient visits as comparator	1.00	0.03	0.52	0.91	1.00	0.03	0.17	0.91
PS stratification, using random days as comparator	1.00	0.02	0.32	0.95	1.00	0.00	0.00	1.00
PS weighting, using outpatient visits as comparator	1.00	0.02	0.42	0.92	1.00	0.02	0.08	0.92
PS weighting, using random days as comparator	1.00	0.00	0.25	0.95	1.00	0.00	0.00	1.00
Per−month PS matching, using outpatient visits as comparator								
Per−month PS matching, using random days as comparator								
Historical comparator								
Unadjusted, using entire historic period	0.94	0.07	1.37	0.88	0.99	0.04	0.33	0.88
Age & sex adjusted, using entire historic period	0.97	0.07	0.93	0.88	0.98	0.07	0.25	0.88
Unadjusted, using TaR after historic visit	0.95	0.07	1.09	0.88	0.99	0.04	0.30	0.88
Age & sex adjusted, using TaR after historic visit	0.98	0.07	0.73	0.88	0.99	0.06	0.23	0.88
Unadjusted, using entire historic period, filtered	0.95	0.07	1.22	0.89	0.99	0.03	0.32	0.89
Age & sex adjusted, using entire historic period, filtered	0.97	0.07	0.85	0.89	0.99	0.05	0.25	0.89
Unadjusted, using TaR after historic visit, filtered	0.96	0.07	0.98	0.89	0.99	0.04	0.29	0.89
Age & sex adjusted, using TaR after historic visit, filtered	0.98	0.07	0.68	0.89	0.99	0.05	0.23	0.89
SCCS / SCRI								
Unadjusted SCCS excluding pre−vaccination window	1.00	0.01	0.04	0.97	1.00	0.00	0.00	1.00
Age & season adjusted SCCS excluding pre−vaccination window	1.00	0.01	0.05	0.97	1.00	0.00	0.00	1.00
SCRI with prior control interval								
SCRI with posterior control interval								
Unadjusted SCCS excluding all pre−vaccination time	1.00	0.01	0.03	0.97	1.00	0.00	0.00	1.00

Figure 101: The coverage, mean precision, mean squared error (MSE), and fraction non-estimable based on the negative control effect-size estimate at the end of the study period in the CCAE database for Seasonal flu vaccination (Fluzone).

13.4 Confidence-interval-based metrics for Seasonal flu vaccination (All)

	Uncalibrated				Calibrated			
Case−control	Coverage	Mean precision	MSE	Non estimable	Coverage	Mean precision	MSE	Non estimable
Age & sex matched controls	0.38	12.24	0.70	0.05	0.95	6.44	0.14	0.05
Age & sex adjusted, using random controls	0.33	14.93	0.65	0.04	0.94	9.25	0.12	0.04
Cohort method								
Unadjusted, using outpatient visits as comparator	0.35	32.94	0.45	0.01	0.95	5.22	0.18	0.01
PS matching, using outpatient visits as comparator	0.60	21.16	0.41	0.03	0.92	9.09	0.19	0.03
Unadjusted, using random days as comparator	0.29	18.56	0.75	0.03	0.99	5.89	0.19	0.03
PS matching, using random days as comparator								
PS stratification, using outpatient visits as comparator	0.48	28.66	0.36	0.01	0.92	8.95	0.17	0.01
PS stratification, using random days as comparator	0.19	26.91	0.79	0.01	0.97	7.29	0.13	0.01
PS weighting, using outpatient visits as comparator	0.49	13.43	0.48	0.01	0.96	7.90	0.26	0.01
PS weighting, using random days as comparator	1.00	0.00	23.17	1.00	1.00	0.00	0.00	1.00
Per−month PS matching, using outpatient visits as comparator								
Per−month PS matching, using random days as comparator								
Historical comparator								
Unadjusted, using entire historic period	0.12	90.76	0.98	0.00	0.99	6.13	0.17	0.00
Age & sex adjusted, using entire historic period	0.12	90.50	0.92	0.00	0.99	7.36	0.14	0.00
Unadjusted, using TaR after historic visit	0.45	79.37	0.21	0.00	0.96	5.84	0.18	0.00
Age & sex adjusted, using TaR after historic visit	0.44	78.42	0.19	0.00	0.99	7.13	0.14	0.00
Unadjusted, using entire historic period, filtered	0.13	84.07	0.88	0.01	0.98	10.15	0.12	0.01
Age & sex adjusted, using entire historic period, filtered	0.13	83.84	0.81	0.01	0.96	15.32	0.09	0.01
Unadjusted, using TaR after historic visit, filtered	0.46	74.03	0.15	0.01	0.96	8.86	0.13	0.01
Age & sex adjusted, using TaR after historic visit, filtered	0.45	73.16	0.13	0.01	0.94	14.63	0.09	0.01
SCCS / SCRI								
Unadjusted SCCS excluding pre−vaccination window	0.61	78.64	0.10	0.00	0.97	14.21	0.10	0.00
Age & season adjusted SCCS excluding pre−vaccination window	0.51	49.23	0.14	0.08	0.97	8.49	0.12	0.08
SCRI with prior control interval	0.43	31.65	18.65	0.01	0.95	7.81	4.62	0.01
SCRI with posterior control interval	0.60	38.69	0.21	0.01	0.97	10.76	0.15	0.01
Unadjusted SCCS excluding all pre−vaccination time	0.51	70.99	0.14	0.00	0.97	12.92	0.10	0.00

Figure 102: The coverage, mean precision, mean squared error (MSE), and fraction non-estimable based on the negative control effect-size estimate at the end of the study period in the Optum EHR database for Seasonal flu vaccination (All).

	Uncalibrated				Calibrated			
Case−control	Coverage	Mean precision	MSE	Non estimable	Coverage	Mean precision	MSE	Non estimable
Age & sex matched controls	0.60	9.03	0.34	0.23	0.96	4.39	0.18	0.23
Age & sex adjusted, using random controls	0.53	14.63	0.31	0.13	0.95	5.74	0.11	0.13
Cohort method								
Unadjusted, using outpatient visits as comparator	0.28	16.72	0.81	0.13	0.91	2.45	0.26	0.13
PS matching, using outpatient visits as comparator	0.83	9.62	0.13	0.15	0.96	8.91	0.08	0.15
Unadjusted, using random days as comparator	0.58	10.55	0.47	0.14	0.96	4.25	0.22	0.14
PS matching, using random days as comparator	0.89	7.71	0.20	0.13	0.97	5.79	0.14	0.13
PS stratification, using outpatient visits as comparator	0.73	12.63	0.21	0.13	0.94	9.66	0.15	0.13
PS stratification, using random days as comparator	0.86	9.81	0.24	0.13	0.99	5.38	0.19	0.13
PS weighting, using outpatient visits as comparator	0.77	12.60	0.19	0.13	0.94	5.68	0.12	0.13
PS weighting, using random days as comparator	0.73	7.48	0.48	0.13	0.96	4.09	0.36	0.13
Per−month PS matching, using outpatient visits as comparator								
Per−month PS matching, using random days as comparator								
Historical comparator								
Unadjusted, using entire historic period	0.57	28.61	0.32	0.13	0.98	2.79	0.21	0.13
Age & sex adjusted, using entire historic period	0.46	28.57	0.41	0.13	0.98	2.88	0.20	0.13
Unadjusted, using TaR after historic visit	0.61	24.99	0.24	0.13	0.99	2.55	0.24	0.13
Age & sex adjusted, using TaR after historic visit	0.72	24.83	0.22	0.13	0.99	2.78	0.22	0.13
Unadjusted, using entire historic period, filtered	0.58	26.84	0.19	0.14	0.97	5.95	0.10	0.14
Age & sex adjusted, using entire historic period, filtered	0.47	26.81	0.27	0.14	0.96	6.22	0.09	0.14
Unadjusted, using TaR after historic visit, filtered	0.62	23.76	0.14	0.14	0.95	4.84	0.13	0.14
Age & sex adjusted, using TaR after historic visit, filtered	0.73	23.62	0.11	0.14	0.95	6.30	0.11	0.14
SCCS / SCRI								
Unadjusted SCCS excluding pre−vaccination window	0.86	25.96	0.08	0.13	0.95	12.56	0.08	0.13
Age & season adjusted SCCS excluding pre−vaccination window	0.90	21.28	0.08	0.16	0.94	9.78	0.08	0.16
SCRI with prior control interval	0.84	12.60	0.12	0.13	0.97	4.72	0.12	0.13
SCRI with posterior control interval	0.86	15.26	0.12	0.13	0.96	7.14	0.12	0.13
Unadjusted SCCS excluding all pre−vaccination time	0.83	24.53	0.10	0.13	0.97	8.82	0.10	0.13

Figure 103: The coverage, mean precision, mean squared error (MSE), and fraction non-estimable based on the negative control effect-size estimate at the end of the study period in the MDCD database for Seasonal flu vaccination (All).

Case−control	Uncalibrated Coverage	Uncalibrated Mean precision	Uncalibrated MSE	Uncalibrated Non estimable	Calibrated Coverage	Calibrated Mean precision	Calibrated MSE	Calibrated Non estimable
Age & sex matched controls	0.98	5.52	0.13	0.32	0.99	5.12	0.13	0.32
Age & sex adjusted, using random controls	0.88	8.60	0.14	0.14	0.99	6.68	0.10	0.14
Cohort method								
Unadjusted, using outpatient visits as comparator	0.53	6.47	0.88	0.17	0.96	2.68	0.32	0.17
PS matching, using outpatient visits as comparator	0.87	3.91	0.63	0.24	0.96	3.47	0.25	0.24
Unadjusted, using random days as comparator	0.87	4.97	0.27	0.19	0.99	3.71	0.26	0.19
PS matching, using random days as comparator	0.91	3.56	0.36	0.23	0.96	3.28	0.26	0.23
PS stratification, using outpatient visits as comparator	0.80	5.64	0.50	0.18	0.99	3.90	0.29	0.18
PS stratification, using random days as comparator	0.91	4.63	0.33	0.19	0.97	3.55	0.24	0.19
PS weighting, using outpatient visits as comparator	0.62	5.31	0.77	0.18	0.98	2.63	0.36	0.18
PS weighting, using random days as comparator	0.92	2.70	0.53	0.19	0.96	2.31	0.49	0.19
Per−month PS matching, using outpatient visits as comparator								
Per−month PS matching, using random days as comparator								
Historical comparator								
Unadjusted, using entire historic period	0.81	11.18	0.22	0.14	0.98	3.26	0.16	0.14
Age & sex adjusted, using entire historic period	0.81	11.18	0.22	0.14	0.98	3.28	0.16	0.14
Unadjusted, using TaR after historic visit	0.85	9.70	0.21	0.14	0.98	2.92	0.20	0.14
Age & sex adjusted, using TaR after historic visit	0.85	9.69	0.20	0.14	0.98	2.99	0.20	0.14
Unadjusted, using entire historic period, filtered	0.83	10.18	0.13	0.16	0.98	8.22	0.09	0.16
Age & sex adjusted, using entire historic period, filtered	0.83	10.18	0.13	0.16	0.99	8.25	0.09	0.16
Unadjusted, using TaR after historic visit, filtered	0.87	8.96	0.13	0.16	0.99	5.24	0.13	0.16
Age & sex adjusted, using TaR after historic visit, filtered	0.87	8.95	0.12	0.16	0.99	5.46	0.12	0.16
SCCS / SCRI								
Unadjusted SCCS excluding pre−vaccination window	0.90	9.38	0.15	0.16	0.98	6.84	0.15	0.16
Age & season adjusted SCCS excluding pre−vaccination window	0.91	7.84	0.07	0.30	0.98	5.73	0.07	0.30
SCRI with prior control interval	0.85	4.20	18.13	0.20	0.97	2.77	0.19	0.20
SCRI with posterior control interval	0.94	5.36	15.49	0.19	0.99	3.97	0.18	0.19
Unadjusted SCCS excluding all pre−vaccination time	0.88	8.69	0.23	0.16	0.98	4.48	0.22	0.16

Figure 104: The coverage, mean precision, mean squared error (MSE), and fraction non-estimable based on the negative control effect-size estimate at the end of the study period in the MDCR database for Seasonal flu vaccination (All).

	Uncalibrated				Calibrated			
Case−control	Coverage	Mean precision	MSE	Non estimable	Coverage	Mean precision	MSE	Non estimable
Age & sex matched controls	0.72	11.55	0.22	0.14	0.96	7.21	0.16	0.14
Age & sex adjusted, using random controls	0.73	14.63	0.15	0.12	0.96	10.71	0.13	0.12
Cohort method								
Unadjusted, using outpatient visits as comparator	0.26	18.02	1.44	0.12	0.92	2.17	0.37	0.12
PS matching, using outpatient visits as comparator	0.76	11.27	0.28	0.13	0.94	7.06	0.14	0.13
Unadjusted, using random days as comparator	0.67	12.28	0.26	0.12	0.95	4.90	0.15	0.12
PS matching, using random days as comparator	0.88	10.69	0.34	0.13	0.95	7.52	0.21	0.13
PS stratification, using outpatient visits as comparator	0.54	15.98	0.35	0.12	0.97	6.81	0.15	0.12
PS stratification, using random days as comparator	0.85	13.70	0.24	0.13	0.96	8.63	0.13	0.13
PS weighting, using outpatient visits as comparator	0.77	6.60	0.31	0.12	0.99	5.13	0.13	0.12
PS weighting, using random days as comparator	0.97	6.83	0.29	0.13	0.98	6.63	0.22	0.13
Per−month PS matching, using outpatient visits as comparator								
Per−month PS matching, using random days as comparator								
Historical comparator								
Unadjusted, using entire historic period	0.56	50.06	0.29	0.10	0.97	3.21	0.25	0.10
Age & sex adjusted, using entire historic period	0.56	49.93	0.25	0.10	0.98	3.58	0.22	0.10
Unadjusted, using TaR after historic visit	0.53	42.23	0.29	0.10	0.98	2.72	0.28	0.10
Age & sex adjusted, using TaR after historic visit	0.58	41.91	0.26	0.10	0.98	3.00	0.26	0.10
Unadjusted, using entire historic period, filtered	0.58	43.78	0.15	0.12	0.96	9.18	0.13	0.12
Age & sex adjusted, using entire historic period, filtered	0.58	43.68	0.11	0.12	0.96	13.91	0.10	0.12
Unadjusted, using TaR after historic visit, filtered	0.55	37.68	0.18	0.12	0.96	5.36	0.15	0.12
Age & sex adjusted, using TaR after historic visit, filtered	0.60	37.41	0.14	0.12	0.97	7.43	0.13	0.12
SCCS / SCRI								
Unadjusted SCCS excluding pre−vaccination window	0.73	44.73	0.09	0.10	0.94	17.86	0.09	0.10
Age & season adjusted SCCS excluding pre−vaccination window	0.76	28.80	0.09	0.17	0.95	13.44	0.09	0.17
SCRI with prior control interval	0.67	20.73	4.30	0.10	0.95	5.37	4.34	0.10
SCRI with posterior control interval	0.83	25.87	0.16	0.10	0.97	8.96	0.16	0.10
Unadjusted SCCS excluding all pre−vaccination time	0.73	41.73	0.12	0.10	0.96	12.75	0.11	0.10

Figure 105: The coverage, mean precision, mean squared error (MSE), and fraction non-estimable based on the negative control effect-size estimate at the end of the study period in the CCAE database for Seasonal flu vaccination (All).

13.5 Confidence-interval-based metrics for Zoster vaccination (Shingrix)

	Uncalibrated				Calibrated			
	Coverage	Mean precision	MSE	Non estimable	Coverage	Mean precision	MSE	Non estimable
Case−control								
Age & sex matched controls	0.78	1.66	30.98	0.35	0.94	1.61	29.68	0.35
Age & sex adjusted, using random controls	0.80	1.79	0.51	0.33	0.98	1.43	0.33	0.33
Cohort method								
Unadjusted, using outpatient visits as comparator	0.56	6.08	1.35	0.12	0.95	1.58	0.62	0.12
PS matching, using outpatient visits as comparator	0.69	4.78	0.82	0.14	0.94	4.19	0.28	0.14
Unadjusted, using random days as comparator	0.61	3.41	1.14	0.18	0.96	1.13	0.65	0.18
PS matching, using random days as comparator								
PS stratification, using outpatient visits as comparator	0.62	6.53	0.76	0.11	0.96	4.00	0.28	0.11
PS stratification, using random days as comparator								
PS weighting, using outpatient visits as comparator	0.92	1.83	0.58	0.11	0.99	1.81	0.26	0.11
PS weighting, using random days as comparator								
Per−month PS matching, using outpatient visits as comparator								
Per−month PS matching, using random days as comparator								
Historical comparator								
Unadjusted, using entire historic period	0.46	9.19	1.17	0.11	0.94	1.45	0.63	0.11
Age & sex adjusted, using entire historic period	0.58	9.18	0.65	0.11	0.96	2.39	0.37	0.11
Unadjusted, using TaR after historic visit	0.61	9.06	0.60	0.11	0.92	1.55	0.61	0.11
Age & sex adjusted, using TaR after historic visit	0.60	8.95	0.44	0.11	0.97	2.20	0.44	0.11
Unadjusted, using entire historic period, filtered	0.47	8.62	1.12	0.12	0.95	1.49	0.61	0.12
Age & sex adjusted, using entire historic period, filtered	0.59	8.61	0.61	0.12	0.97	2.64	0.35	0.12
Unadjusted, using TaR after historic visit, filtered	0.62	8.50	0.59	0.12	0.92	1.56	0.59	0.12
Age & sex adjusted, using TaR after historic visit, filtered	0.61	8.40	0.43	0.12	0.98	2.31	0.42	0.12
SCCS / SCRI								
Unadjusted SCCS excluding pre−vaccination window	0.88	7.78	0.24	0.11	0.97	4.29	0.24	0.11
Age & season adjusted SCCS excluding pre−vaccination window	0.85	7.75	0.25	0.11	0.97	4.28	0.25	0.11
SCRI with prior control interval	0.90	4.30	45.63	0.14	0.97	3.06	0.26	0.14
SCRI with posterior control interval	0.91	3.87	30.27	0.13	0.98	3.11	0.32	0.13
Unadjusted SCCS excluding all pre−vaccination time	0.84	6.13	0.33	0.11	0.98	4.36	0.32	0.11

Figure 106: The coverage, mean precision, mean squared error (MSE), and fraction non-estimable based on the negative control effect-size estimate at the end of the study period in the Optum EHR database for Zoster vaccination (Shingrix).

	Uncalibrated				Calibrated			
Case−control	Coverage	Mean precision	MSE	Non estimable	Coverage	Mean precision	MSE	Non estimable
Age & sex matched controls	0.98	0.35	15.64	0.68	0.99	0.34	0.17	0.68
Age & sex adjusted, using random controls	0.97	0.51	0.14	0.61	1.00	0.51	0.09	0.61
Cohort method								
Unadjusted, using outpatient visits as comparator	0.99	0.64	0.78	0.55	1.00	0.62	0.26	0.55
PS matching, using outpatient visits as comparator	0.98	0.36	0.75	0.66	1.00	0.36	0.15	0.66
Unadjusted, using random days as comparator	0.91	0.34	0.48	0.71	0.99	0.26	0.20	0.71
PS matching, using random days as comparator	0.99	0.34	0.83	0.66	1.00	0.34	0.14	0.66
PS stratification, using outpatient visits as comparator	0.97	0.68	0.94	0.47	0.99	0.67	0.43	0.47
PS stratification, using random days as comparator	1.00	0.57	0.72	0.49	1.00	0.57	0.25	0.49
PS weighting, using outpatient visits as comparator	1.00	0.24	1.24	0.47	1.00	0.24	0.84	0.47
PS weighting, using random days as comparator	1.00	0.26	1.47	0.49	1.00	0.26	1.07	0.49
Per−month PS matching, using outpatient visits as comparator								
Per−month PS matching, using random days as comparator								
Historical comparator								
Unadjusted, using entire historic period	0.76	1.15	1.14	0.48	0.97	0.51	0.33	0.48
Age & sex adjusted, using entire historic period	0.92	1.15	0.35	0.48	0.99	0.79	0.17	0.48
Unadjusted, using TaR after historic visit	0.81	1.14	0.62	0.48	0.98	0.58	0.27	0.48
Age & sex adjusted, using TaR after historic visit	0.98	1.13	0.19	0.48	0.99	0.82	0.19	0.48
Unadjusted, using entire historic period, filtered	0.77	1.08	1.04	0.49	0.98	0.58	0.29	0.49
Age & sex adjusted, using entire historic period, filtered	0.94	1.08	0.30	0.49	1.00	1.07	0.15	0.49
Unadjusted, using TaR after historic visit, filtered	0.82	1.07	0.56	0.49	0.98	0.66	0.24	0.49
Age & sex adjusted, using TaR after historic visit, filtered	0.99	1.07	0.17	0.49	1.00	1.06	0.17	0.49
SCCS / SCRI								
Unadjusted SCCS excluding pre−vaccination window	1.00	0.99	0.17	0.49	1.00	0.99	0.16	0.49
Age & season adjusted SCCS excluding pre−vaccination window	1.00	0.97	0.18	0.49	1.00	0.96	0.17	0.49
SCRI with prior control interval	0.98	0.61	58.54	0.57	1.00	0.61	0.14	0.57
SCRI with posterior control interval	0.99	0.56	29.74	0.57	0.99	0.56	0.18	0.57
Unadjusted SCCS excluding all pre−vaccination time	1.00	0.85	16.39	0.52	1.00	0.84	0.14	0.52

Figure 107: The coverage, mean precision, mean squared error (MSE), and fraction non-estimable based on the negative control effect-size estimate at the end of the study period in the MDCD database for Zoster vaccination (Shingrix).

Case−control	Uncalibrated Coverage	Uncalibrated Mean precision	Uncalibrated MSE	Uncalibrated Non estimable	Calibrated Coverage	Calibrated Mean precision	Calibrated MSE	Calibrated Non estimable
Age & sex matched controls	0.89	1.57	0.21	0.52	0.99	0.90	0.21	0.52
Age & sex adjusted, using random controls	0.85	2.37	0.39	0.30	0.98	1.18	0.39	0.30
Cohort method								
Unadjusted, using outpatient visits as comparator	0.65	2.01	1.63	0.33	0.96	0.68	0.65	0.33
PS matching, using outpatient visits as comparator	0.94	1.37	0.79	0.41	0.98	1.22	0.22	0.41
Unadjusted, using random days as comparator	0.91	1.65	0.77	0.34	0.97	1.00	0.42	0.34
PS matching, using random days as comparator	0.97	1.34	0.51	0.40	0.99	1.32	0.25	0.40
PS stratification, using outpatient visits as comparator	0.83	2.01	0.80	0.33	0.97	1.17	0.38	0.33
PS stratification, using random days as comparator	0.91	1.96	0.60	0.31	0.96	1.68	0.38	0.31
PS weighting, using outpatient visits as comparator	0.77	1.72	1.22	0.38	0.97	0.80	0.61	0.38
PS weighting, using random days as comparator	1.00	0.59	0.97	0.31	0.99	0.59	0.75	0.31
Per−month PS matching, using outpatient visits as comparator								
Per−month PS matching, using random days as comparator								
Historical comparator								
Unadjusted, using entire historic period	0.81	2.89	0.45	0.30	0.98	0.92	0.45	0.30
Age & sex adjusted, using entire historic period	0.81	2.89	0.42	0.30	0.98	0.97	0.43	0.30
Unadjusted, using TaR after historic visit	0.72	2.74	0.66	0.30	0.97	0.72	0.60	0.30
Age & sex adjusted, using TaR after historic visit	0.74	2.74	0.63	0.30	0.98	0.75	0.58	0.30
Unadjusted, using entire historic period, filtered	0.83	2.58	0.37	0.33	0.99	1.00	0.37	0.33
Age & sex adjusted, using entire historic period, filtered	0.83	2.58	0.35	0.33	0.99	1.06	0.36	0.33
Unadjusted, using TaR after historic visit, filtered	0.75	2.47	0.56	0.33	0.97	0.78	0.47	0.33
Age & sex adjusted, using TaR after historic visit, filtered	0.77	2.47	0.54	0.33	0.97	0.81	0.45	0.33
SCCS / SCRI								
Unadjusted SCCS excluding pre−vaccination window	0.92	2.50	0.27	0.31	0.97	1.76	0.27	0.31
Age & season adjusted SCCS excluding pre−vaccination window	0.95	2.41	0.23	0.35	0.98	1.88	0.23	0.35
SCRI with prior control interval	0.97	1.55	29.60	0.38	0.99	1.53	0.31	0.38
SCRI with posterior control interval	0.95	1.50	45.39	0.42	0.98	1.45	0.24	0.42
Unadjusted SCCS excluding all pre−vaccination time	0.91	2.17	0.30	0.32	0.96	1.99	0.30	0.32

Figure 108: The coverage, mean precision, mean squared error (MSE), and fraction non-estimable based on the negative control effect-size estimate at the end of the study period in the MDCR database for Zoster vaccination (Shingrix).

Case−control	Uncalibrated Coverage	Uncalibrated Mean precision	Uncalibrated MSE	Uncalibrated Non estimable	Calibrated Coverage	Calibrated Mean precision	Calibrated MSE	Calibrated Non estimable
Age & sex matched controls	0.97	1.41	15.00	0.32	0.98	1.31	0.31	0.32
Age & sex adjusted, using random controls	0.97	1.59	0.26	0.28	0.98	1.42	0.28	0.28
Cohort method								
Unadjusted, using outpatient visits as comparator	0.62	3.56	1.35	0.18	0.94	1.33	0.56	0.18
PS matching, using outpatient visits as comparator	0.87	2.59	0.59	0.24	0.99	2.55	0.31	0.24
Unadjusted, using random days as comparator	0.74	2.10	0.76	0.28	0.97	0.79	0.59	0.28
PS matching, using random days as comparator	0.97	2.63	0.36	0.25	0.98	2.52	0.23	0.25
PS stratification, using outpatient visits as comparator	0.76	3.82	0.62	0.18	0.96	3.00	0.33	0.18
PS stratification, using random days as comparator	0.94	3.57	0.35	0.17	0.97	3.17	0.24	0.17
PS weighting, using outpatient visits as comparator	0.98	1.02	0.44	0.18	1.00	1.02	0.24	0.18
PS weighting, using random days as comparator	1.00	1.14	0.39	0.17	1.00	1.14	0.31	0.17
Per−month PS matching, using outpatient visits as comparator								
Per−month PS matching, using random days as comparator								
Historical comparator								
Unadjusted, using entire historic period	0.68	5.06	0.58	0.18	0.96	1.30	0.44	0.18
Age & sex adjusted, using entire historic period	0.88	5.04	0.24	0.18	0.98	2.19	0.26	0.18
Unadjusted, using TaR after historic visit	0.76	4.98	0.41	0.18	0.95	1.39	0.42	0.18
Age & sex adjusted, using TaR after historic visit	0.76	4.86	0.41	0.18	0.96	1.86	0.35	0.18
Unadjusted, using entire historic period, filtered	0.70	4.52	0.50	0.20	0.96	1.49	0.39	0.20
Age & sex adjusted, using entire historic period, filtered	0.90	4.51	0.21	0.20	0.96	3.87	0.22	0.20
Unadjusted, using TaR after historic visit, filtered	0.78	4.46	0.36	0.20	0.95	1.56	0.37	0.20
Age & sex adjusted, using TaR after historic visit, filtered	0.78	4.36	0.39	0.20	0.92	2.38	0.31	0.20
SCCS / SCRI								
Unadjusted SCCS excluding pre−vaccination window	0.96	4.42	0.18	0.18	0.98	3.62	0.18	0.18
Age & season adjusted SCCS excluding pre−vaccination window	0.96	4.11	0.17	0.20	0.99	3.17	0.17	0.20
SCRI with prior control interval	0.94	2.74	18.91	0.23	0.97	2.24	0.26	0.23
SCRI with posterior control interval	0.96	2.40	59.36	0.30	0.98	2.22	0.29	0.30
Unadjusted SCCS excluding all pre−vaccination time	0.96	3.66	0.37	0.18	0.97	3.40	0.37	0.18

Figure 109: The coverage, mean precision, mean squared error (MSE), and fraction non-estimable based on the negative control effect-size estimate at the end of the study period in the CCAE database for Zoster vaccination (Shingrix).

13.6 Confidence-interval-based metrics for HPV vaccination (Gardasil 9)

	Uncalibrated				Calibrated			
	Coverage	Mean precision	MSE	Non estimable	Coverage	Mean precision	MSE	Non estimable
**Case−control**								
Age & sex matched controls	0.90	0.74	1.04	0.52	0.99	0.38	0.62	0.52
Age & sex adjusted, using random controls	0.96	0.63	0.47	0.56	0.98	0.46	0.33	0.56
**Cohort method**								
Unadjusted, using outpatient visits as comparator	0.53	2.03	4.33	0.39	0.97	0.31	1.13	0.39
PS matching, using outpatient visits as comparator	0.95	1.09	0.81	0.52	0.99	1.02	0.15	0.52
Unadjusted, using random days as comparator	0.74	1.68	1.92	0.40	0.96	0.40	0.73	0.40
PS matching, using random days as comparator								
PS stratification, using outpatient visits as comparator	0.87	1.65	0.76	0.39	0.98	1.24	0.23	0.39
PS stratification, using random days as comparator								
PS weighting, using outpatient visits as comparator	1.00	0.54	0.92	0.39	0.99	0.53	0.47	0.39
PS weighting, using random days as comparator								
Per−month PS matching, using outpatient visits as comparator								
Per−month PS matching, using random days as comparator								
**Historical comparator**								
Unadjusted, using entire historic period	0.70	2.48	0.89	0.38	0.97	0.46	0.82	0.38
Age & sex adjusted, using entire historic period	0.83	2.47	0.45	0.38	0.98	1.05	0.23	0.38
Unadjusted, using TaR after historic visit	0.56	2.47	1.66	0.38	0.97	0.39	0.97	0.38
Age & sex adjusted, using TaR after historic visit	0.87	2.35	0.29	0.38	0.95	1.11	0.28	0.38
Unadjusted, using entire historic period, filtered	0.71	2.35	0.89	0.39	0.97	0.44	0.81	0.39
Age & sex adjusted, using entire historic period, filtered	0.84	2.33	0.41	0.39	0.97	1.19	0.21	0.39
Unadjusted, using TaR after historic visit, filtered	0.57	2.33	1.65	0.39	0.97	0.37	0.96	0.39
Age & sex adjusted, using TaR after historic visit, filtered	0.88	2.23	0.28	0.39	0.96	1.23	0.27	0.39
**SCCS / SCRI**								
Unadjusted SCCS excluding pre−vaccination window	0.90	2.17	0.40	0.38	0.97	1.13	0.36	0.38
Age & season adjusted SCCS excluding pre−vaccination window	0.90	2.17	0.41	0.38	0.98	1.09	0.37	0.38
SCRI with prior control interval	0.96	1.07	74.44	0.56	0.98	0.73	0.15	0.56
SCRI with posterior control interval	0.96	1.10	30.11	0.47	1.00	0.86	0.22	0.47
Unadjusted SCCS excluding all pre−vaccination time	0.91	1.77	0.43	0.39	0.98	1.32	0.34	0.39

Figure 110: The coverage, mean precision, mean squared error (MSE), and fraction non-estimable based on the negative control effect-size estimate at the end of the study period in the Optum EHR database for HPV vaccination (Gardasil 9).

	Uncalibrated				Calibrated			
Case−control	Coverage	Mean precision	MSE	Non estimable	Coverage	Mean precision	MSE	Non estimable
Age & sex matched controls	0.90	1.07	0.34	0.61	0.98	0.76	0.20	0.61
Age & sex adjusted, using random controls	0.94	1.22	0.23	0.54	0.99	0.97	0.14	0.54
Cohort method								
Unadjusted, using outpatient visits as comparator	0.59	1.55	4.09	0.47	1.00	0.23	1.14	0.47
PS matching, using outpatient visits as comparator	0.94	0.82	0.88	0.61	0.99	0.58	0.21	0.61
Unadjusted, using random days as comparator	0.78	1.30	2.30	0.49	0.97	0.30	0.79	0.49
PS matching, using random days as comparator	0.94	0.77	0.59	0.61	0.99	0.48	0.19	0.61
PS stratification, using outpatient visits as comparator	0.88	1.25	0.73	0.49	0.98	0.76	0.23	0.49
PS stratification, using random days as comparator	0.92	1.12	1.03	0.47	0.99	0.70	0.67	0.47
PS weighting, using outpatient visits as comparator	0.97	0.47	1.06	0.49	0.99	0.46	0.57	0.49
PS weighting, using random days as comparator	0.99	0.44	1.83	0.48	1.00	0.33	1.40	0.49
Per−month PS matching, using outpatient visits as comparator								
Per−month PS matching, using random days as comparator								
Historical comparator								
Unadjusted, using entire historic period	0.75	1.92	1.07	0.46	0.96	0.35	0.81	0.46
Age & sex adjusted, using entire historic period	0.89	1.91	0.30	0.46	0.98	0.70	0.26	0.46
Unadjusted, using TaR after historic visit	0.71	1.89	1.55	0.46	0.97	0.31	0.91	0.46
Age & sex adjusted, using TaR after historic visit	0.88	1.78	0.35	0.46	0.97	0.65	0.35	0.46
Unadjusted, using entire historic period, filtered	0.76	1.77	1.06	0.48	0.95	0.34	0.76	0.48
Age & sex adjusted, using entire historic period, filtered	0.90	1.76	0.22	0.48	0.97	0.87	0.21	0.48
Unadjusted, using TaR after historic visit, filtered	0.72	1.75	1.55	0.48	0.97	0.30	0.87	0.48
Age & sex adjusted, using TaR after historic visit, filtered	0.90	1.66	0.26	0.48	0.96	0.80	0.26	0.48
SCCS / SCRI								
Unadjusted SCCS excluding pre−vaccination window	0.94	1.71	0.27	0.47	0.97	1.12	0.23	0.47
Age & season adjusted SCCS excluding pre−vaccination window	0.94	1.60	0.28	0.48	0.97	1.04	0.26	0.48
SCRI with prior control interval	0.96	1.03	0.21	0.55	0.99	0.58	0.19	0.55
SCRI with posterior control interval	0.95	1.03	0.17	0.57	0.98	0.61	0.18	0.57
Unadjusted SCCS excluding all pre−vaccination time	0.92	1.48	0.22	0.49	0.98	0.87	0.18	0.49

Figure 111: The coverage, mean precision, mean squared error (MSE), and fraction non-estimable based on the negative control effect-size estimate at the end of the study period in the MDCD database for HPV vaccination (Gardasil 9).

	Uncalibrated				Calibrated			
Case−control	Coverage	Mean precision	MSE	Non estimable	Coverage	Mean precision	MSE	Non estimable
Age & sex matched controls								
Age & sex adjusted, using random controls								
Cohort method								
Unadjusted, using outpatient visits as comparator	1.00	0.00	0.04	1.00	1.00	0.00	0.00	1.00
PS matching, using outpatient visits as comparator								
Unadjusted, using random days as comparator								
PS matching, using random days as comparator								
PS stratification, using outpatient visits as comparator	1.00	0.00	0.03	1.00	1.00	0.00	0.00	1.00
PS stratification, using random days as comparator								
PS weighting, using outpatient visits as comparator	1.00	0.00	0.02	1.00	1.00	0.00	0.00	1.00
PS weighting, using random days as comparator								
Per−month PS matching, using outpatient visits as comparator								
Per−month PS matching, using random days as comparator								
Historical comparator								
Unadjusted, using entire historic period	1.00	0.00	0.00	1.00	1.00	0.00	0.00	1.00
Age & sex adjusted, using entire historic period	1.00	0.00	0.00	1.00	1.00	0.00	0.00	1.00
Unadjusted, using TaR after historic visit	1.00	0.00	0.00	1.00	1.00	0.00	0.00	1.00
Age & sex adjusted, using TaR after historic visit	1.00	0.00	0.00	1.00	1.00	0.00	0.00	1.00
Unadjusted, using entire historic period, filtered	1.00	0.00	0.00	1.00	1.00	0.00	0.00	1.00
Age & sex adjusted, using entire historic period, filtered	1.00	0.00	0.00	1.00	1.00	0.00	0.00	1.00
Unadjusted, using TaR after historic visit, filtered	1.00	0.00	0.00	1.00	1.00	0.00	0.00	1.00
Age & sex adjusted, using TaR after historic visit, filtered	1.00	0.00	0.00	1.00	1.00	0.00	0.00	1.00

Figure 112: The coverage, mean precision, mean squared error (MSE), and fraction non-estimable based on the negative control effect-size estimate at the end of the study period in the MDCR database for HPV vaccination (Gardasil 9).

Case−control	Uncalibrated Coverage	Uncalibrated Mean precision	Uncalibrated MSE	Uncalibrated Non estimable	Calibrated Coverage	Calibrated Mean precision	Calibrated MSE	Calibrated Non estimable
Age & sex matched controls	0.92	1.16	0.50	0.51	0.99	0.96	0.35	0.51
Age & sex adjusted, using random controls	0.95	0.99	0.37	0.53	0.96	0.82	0.29	0.53
Cohort method								
Unadjusted, using outpatient visits as comparator	0.54	2.27	4.01	0.39	0.98	0.32	1.02	0.39
PS matching, using outpatient visits as comparator	0.96	1.26	0.80	0.53	1.00	1.06	0.14	0.53
Unadjusted, using random days as comparator	0.75	1.93	1.88	0.40	0.97	0.45	0.57	0.40
PS matching, using random days as comparator	0.95	1.16	0.65	0.55	0.99	0.84	0.11	0.55
PS stratification, using outpatient visits as comparator	0.89	1.87	0.63	0.42	0.96	1.32	0.20	0.42
PS stratification, using random days as comparator	0.92	1.59	0.74	0.41	0.97	1.12	0.35	0.41
PS weighting, using outpatient visits as comparator	1.00	0.62	0.91	0.42	1.00	0.62	0.54	0.42
PS weighting, using random days as comparator	0.98	0.50	1.00	0.41	0.99	0.46	0.71	0.41
Per−month PS matching, using outpatient visits as comparator								
Per−month PS matching, using random days as comparator								
Historical comparator								
Unadjusted, using entire historic period	0.69	2.77	0.86	0.38	0.97	0.49	0.60	0.38
Age & sex adjusted, using entire historic period	0.91	2.75	0.24	0.38	0.99	1.16	0.20	0.38
Unadjusted, using TaR after historic visit	0.58	2.73	1.38	0.38	0.98	0.39	0.82	0.38
Age & sex adjusted, using TaR after historic visit	0.89	2.54	0.27	0.38	0.99	1.06	0.27	0.38
Unadjusted, using entire historic period, filtered	0.69	2.53	0.86	0.40	0.97	0.46	0.59	0.40
Age & sex adjusted, using entire historic period, filtered	0.92	2.51	0.20	0.40	0.98	1.41	0.17	0.40
Unadjusted, using TaR after historic visit, filtered	0.59	2.50	1.38	0.40	0.98	0.36	0.81	0.40
Age & sex adjusted, using TaR after historic visit, filtered	0.90	2.33	0.25	0.40	0.99	1.17	0.25	0.40
SCCS / SCRI								
Unadjusted SCCS excluding pre−vaccination window	0.90	2.44	0.30	0.39	0.97	1.50	0.25	0.39
Age & season adjusted SCCS excluding pre−vaccination window	0.90	2.43	0.30	0.39	0.99	1.49	0.26	0.39
SCRI with prior control interval	0.95	1.37	75.77	0.52	0.99	0.74	0.21	0.52
SCRI with posterior control interval	0.97	1.43	15.03	0.49	0.99	0.94	0.09	0.49
Unadjusted SCCS excluding all pre−vaccination time	0.91	2.11	0.35	0.41	0.98	1.22	0.26	0.41

Figure 113: The coverage, mean precision, mean squared error (MSE), and fraction non-estimable based on the negative control effect-size estimate at the end of the study period in the CCAE database for HPV vaccination (Gardasil 9).

14 Negative controls effect size estimates

Dots indicate the estimated effect size (x-axis) such as odds ratio or incidence rate ratio, and corresponding standard error (y-axis), which is linearly related to the width of the confidence interval. Estimates below the red dashed line have a one-sided p-value < 0.05, and filled dots indicate the LLR exceeds the CV.

14.1 Negative controls effect size estimates in Optum EHR

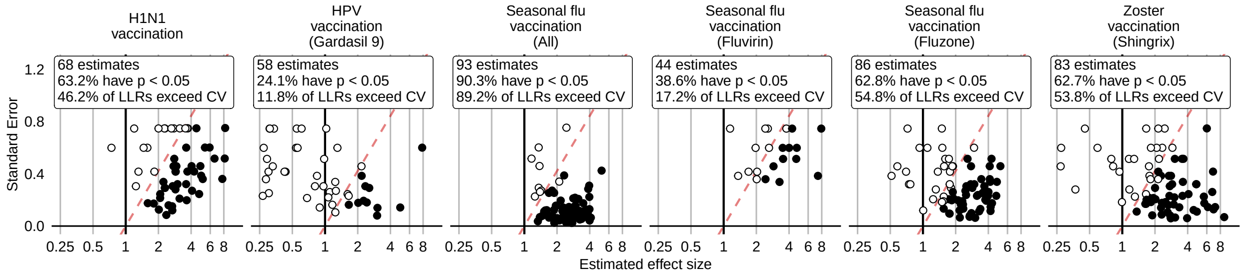

Figure 114: Negative control effect-size estimates and standard errors at the end of the study period using the HistoricalComparator method (Unadjusted, using entire historic period), in the Optum EHR database.

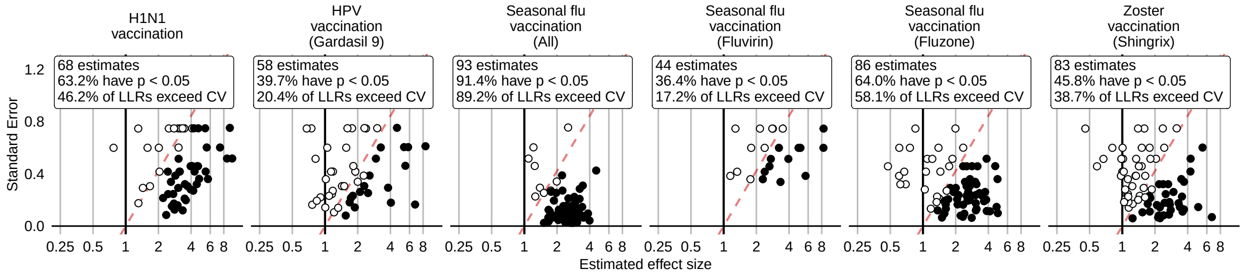

Figure 115: Negative control effect-size estimates and standard errors at the end of the study period using the HistoricalComparator method (Age & sex adjusted, using entire historic period), in the Optum EHR database.

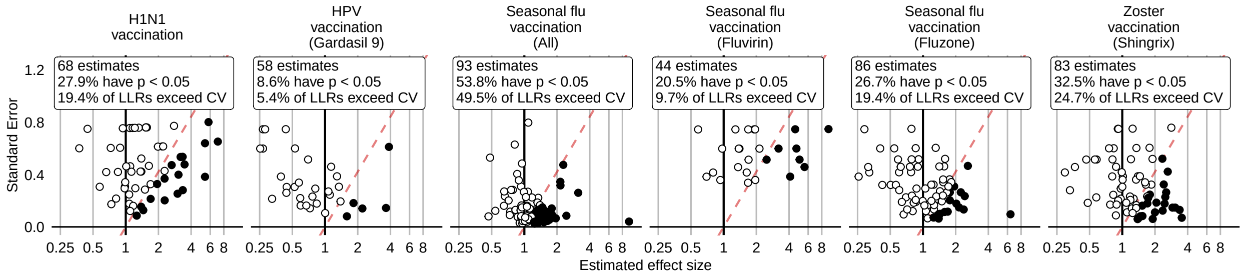

Figure 116: Negative control effect-size estimates and standard errors at the end of the study period using the HistoricalComparator method (Unadjusted, using TaR after historic visit), in the Optum EHR database.

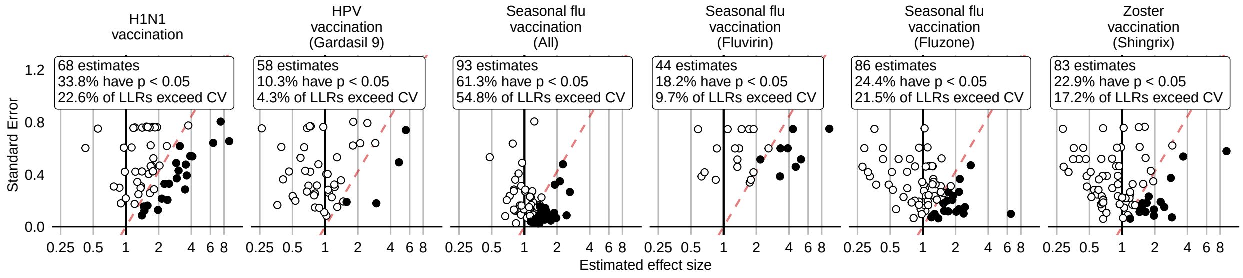

Figure 117: Negative control effect-size estimates and standard errors at the end of the study period using the HistoricalComparator method (Age & sex adjusted, using TaR after historic visit), in the Optum EHR database.

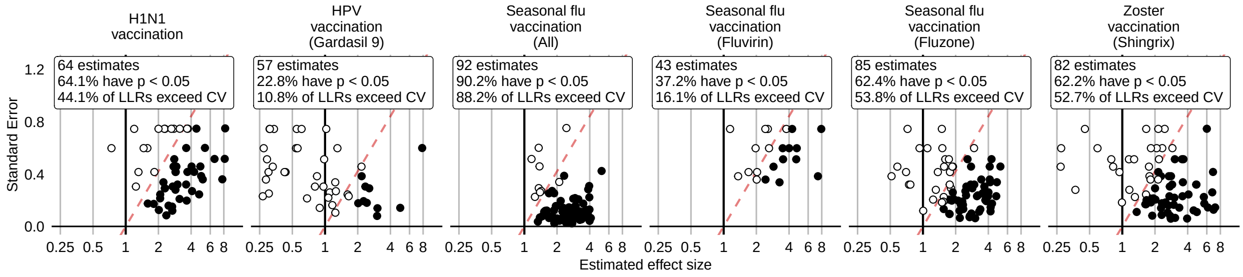

Figure 118: Negative control effect-size estimates and standard errors at the end of the study period using the HistoricalComparator method (Unadjusted, using entire historic period, filtered), in the Optum EHR database.

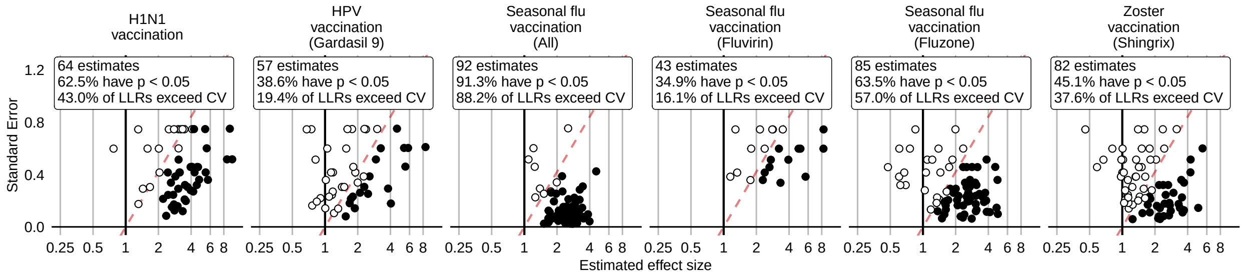

Figure 119: Negative control effect-size estimates and standard errors at the end of the study period using the HistoricalComparator method (Age & sex adjusted, using entire historic period, filtered), in the Optum EHR database.

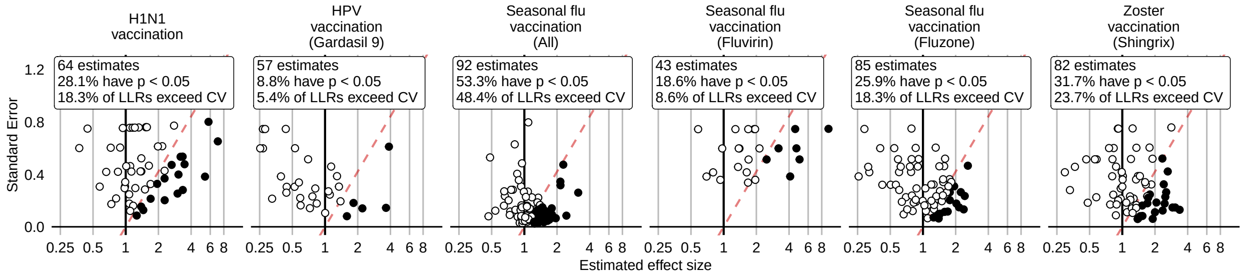

Figure 120: Negative control effect-size estimates and standard errors at the end of the study period using the HistoricalComparator method (Unadjusted, using TaR after historic visit, filtered), in the Optum EHR database.

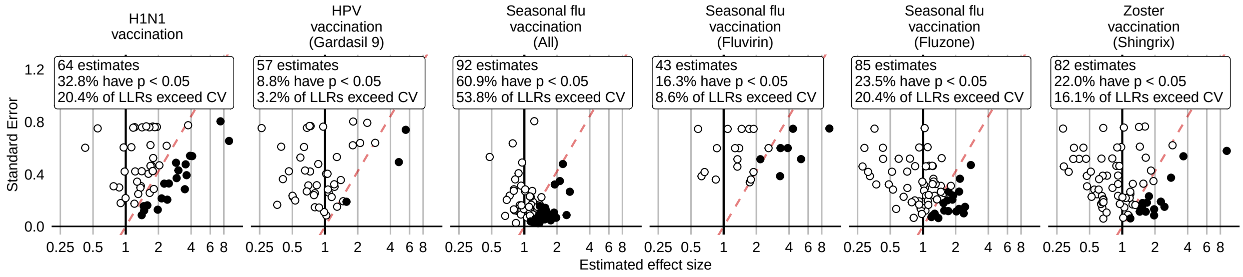

Figure 121: Negative control effect-size estimates and standard errors at the end of the study period using the HistoricalComparator method (Age & sex adjusted, using TaR after historic visit, filtered), in the Optum EHR database.

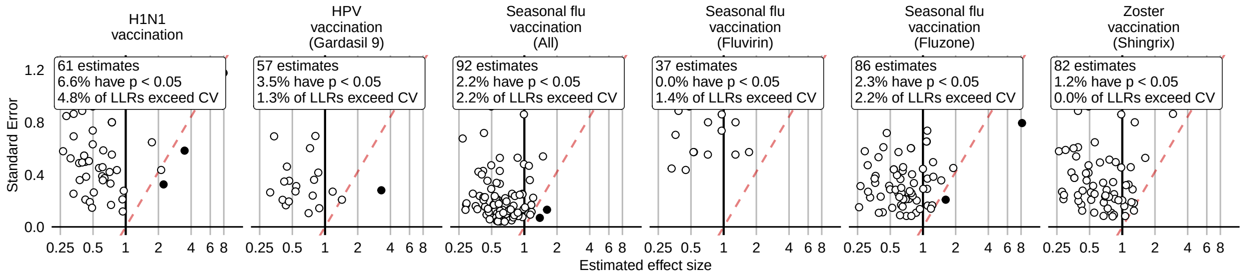

Figure 122: Negative control effect-size estimates and standard errors at the end of the study period using the CohortMethod method (Unadjusted, using outpatient visits as comparator), in the Optum EHR database.

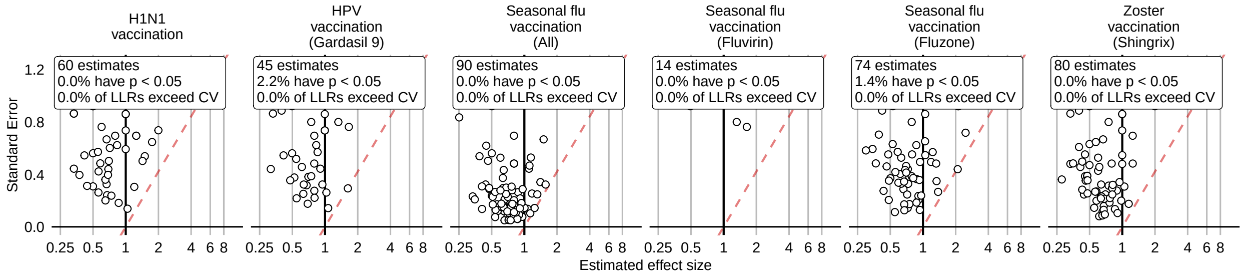

Figure 123: Negative control effect-size estimates and standard errors at the end of the study period using the CohortMethod method (PS matching, using outpatient visits as comparator), in the Optum EHR database.

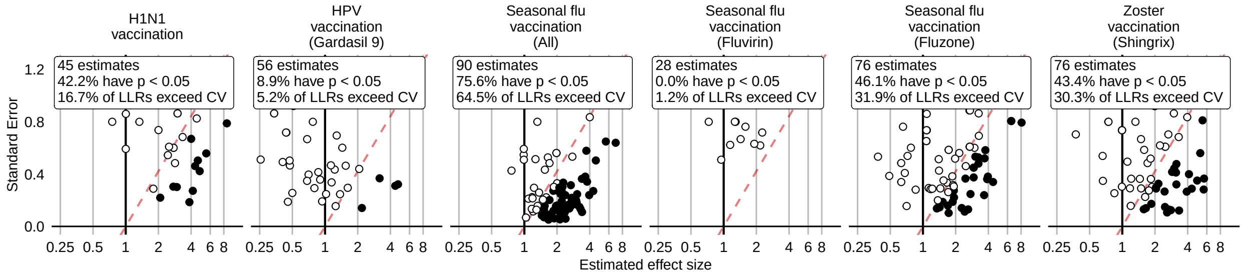

Figure 124: Negative control effect-size estimates and standard errors at the end of the study period using the CohortMethod method (Unadjusted, using random days as comparator), in the Optum EHR database.

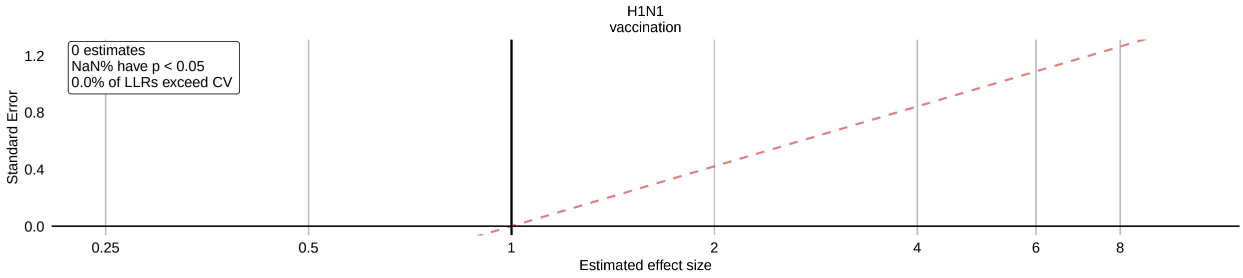

Figure 125: Negative control effect-size estimates and standard errors at the end of the study period using the CohortMethod method (PS matching, using random days as comparator), in the Optum EHR database.

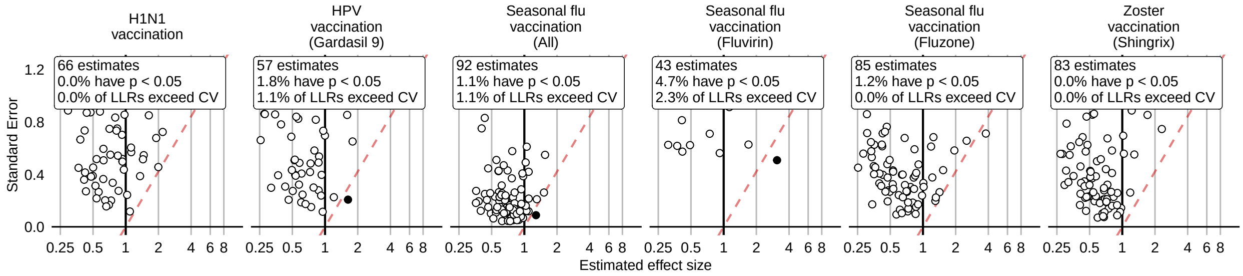

Figure 126: Negative control effect-size estimates and standard errors at the end of the study period using the CohortMethod method (PS stratification, using outpatient visits as comparator), in the Optum EHR database.

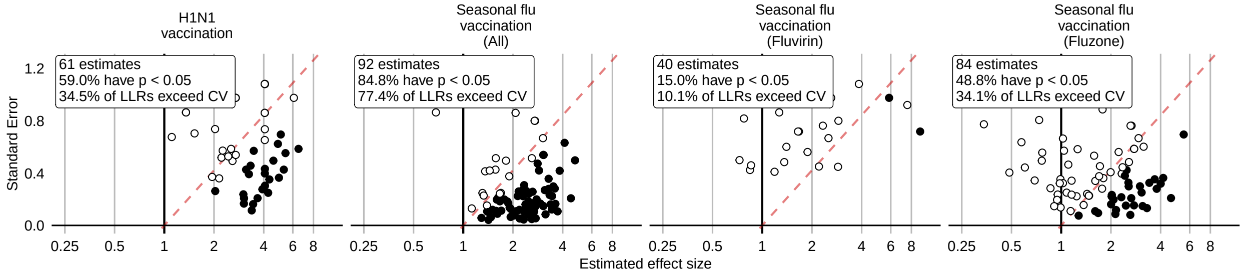

Figure 127: Negative control effect-size estimates and standard errors at the end of the study period using the CohortMethod method (PS stratification, using random days as comparator), in the Optum EHR database.

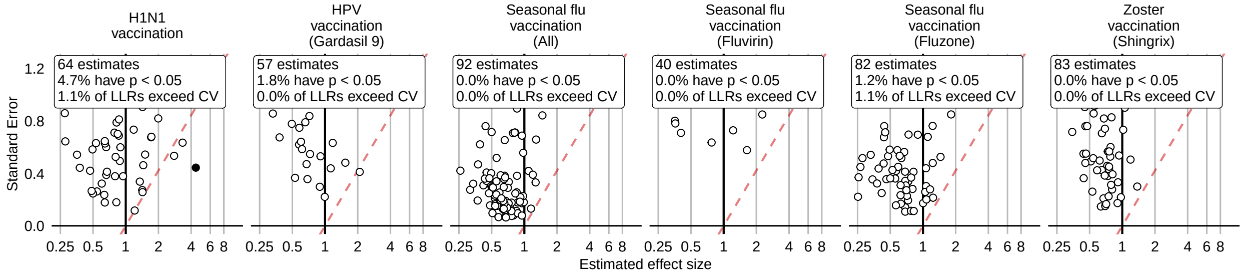

Figure 128: Negative control effect-size estimates and standard errors at the end of the study period using the CohortMethod method (PS weighting, using outpatient visits as comparator), in the Optum EHR database.

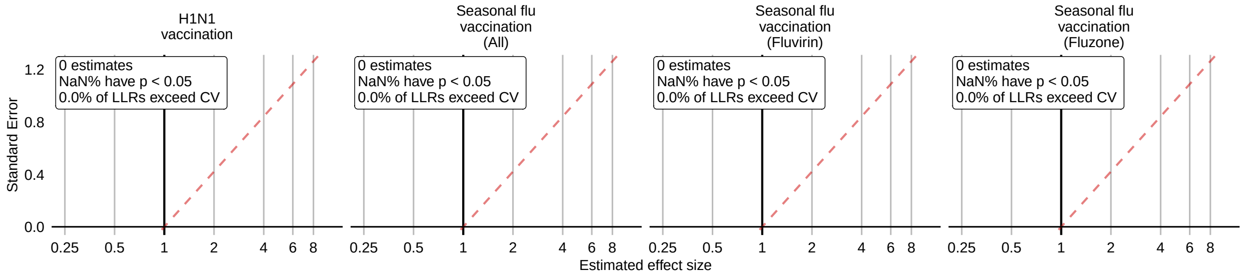

Figure 129: Negative control effect-size estimates and standard errors at the end of the study period using the CohortMethod method (PS weighting, using random days as comparator), in the Optum EHR database.

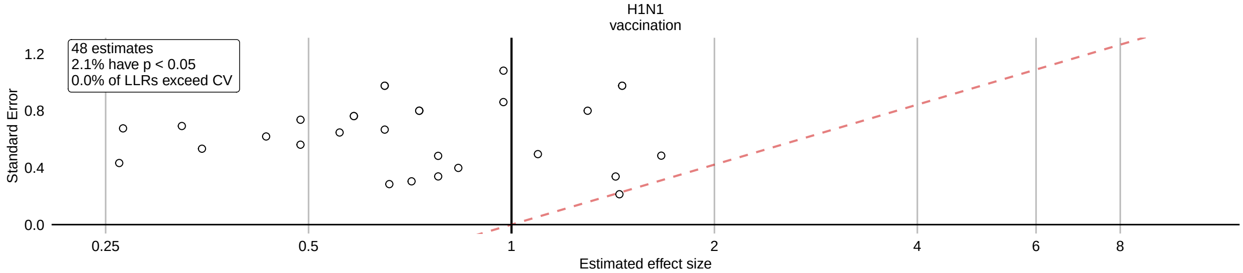

Figure 130: Negative control effect-size estimates and standard errors at the end of the study period using the CohortMethod method (Per-month PS matching, using outpatient visits as comparator), in the Optum EHR database.

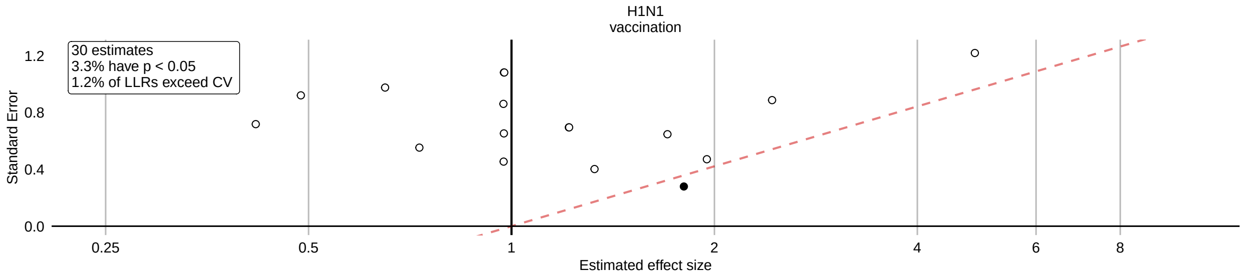

Figure 131: Negative control effect-size estimates and standard errors at the end of the study period using the CohortMethod method (Per-month PS matching, using random days as comparator), in the Optum EHR database.

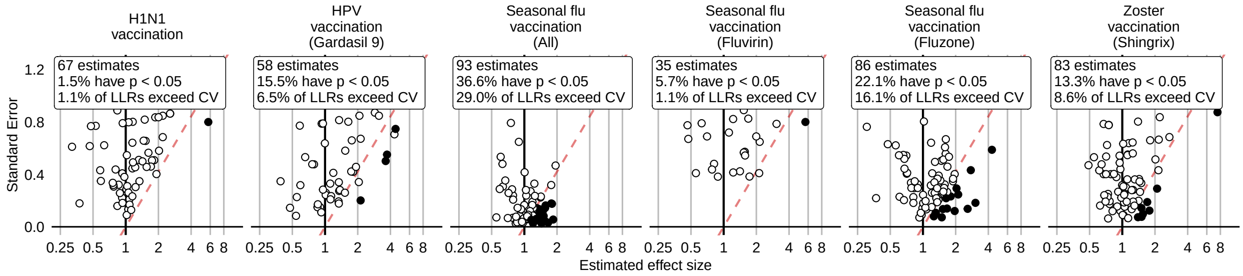

Figure 132: Negative control effect-size estimates and standard errors at the end of the study period using the SCCS method (Unadjusted SCCS excluding pre-vaccination window), in the Optum EHR database.

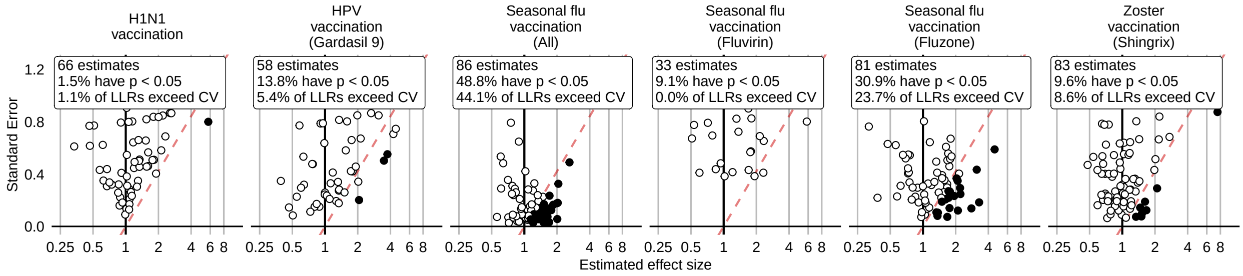

Figure 133: Negative control effect-size estimates and standard errors at the end of the study period using the SCCS method (Age & season adjusted SCCS excluding pre-vaccination window), in the Optum EHR database.

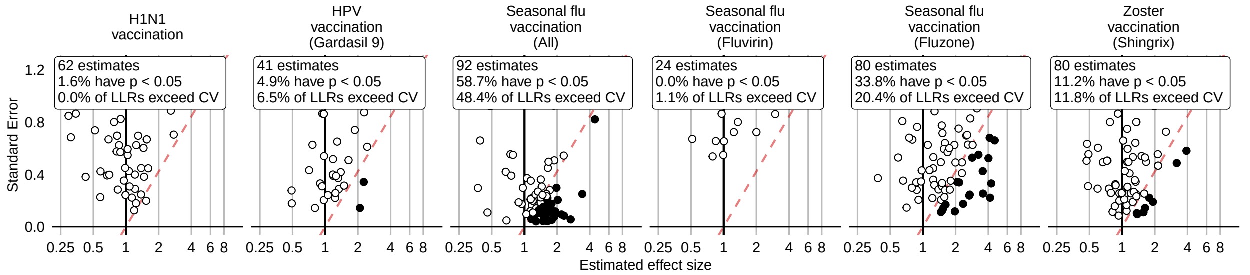

Figure 134: Negative control effect-size estimates and standard errors at the end of the study period using the SCCS method (SCRI with prior control interval), in the Optum EHR database.

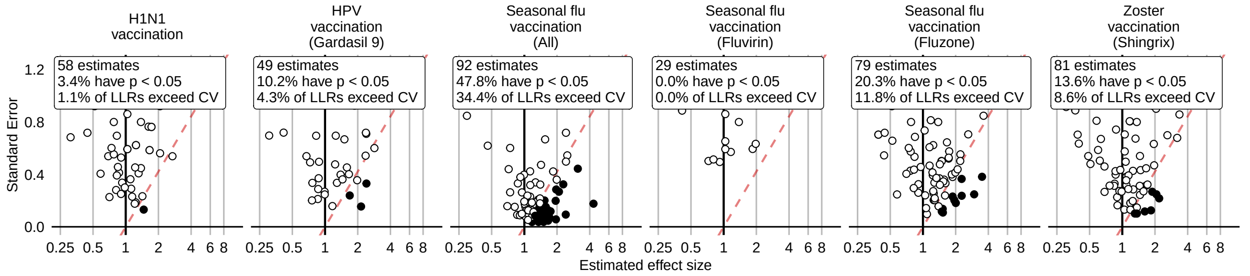

Figure 135: Negative control effect-size estimates and standard errors at the end of the study period using the SCCS method (SCRI with posterior control interval), in the Optum EHR database.

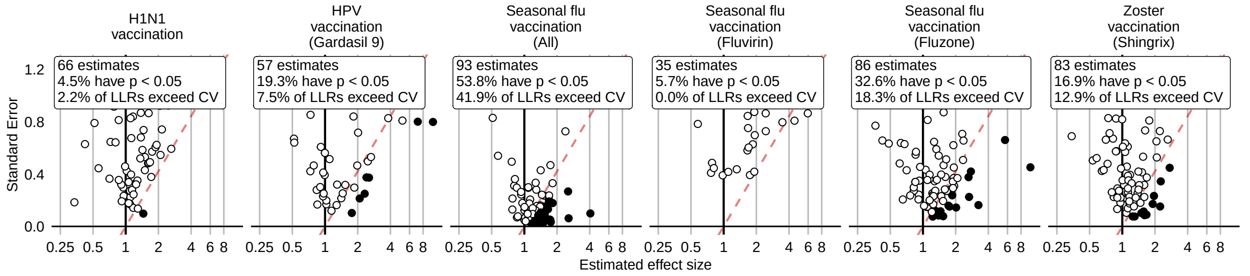

Figure 136: Negative control effect-size estimates and standard errors at the end of the study period using the SCCS method (Unadjusted SCCS excluding all pre-vaccination time), in the Optum EHR database.

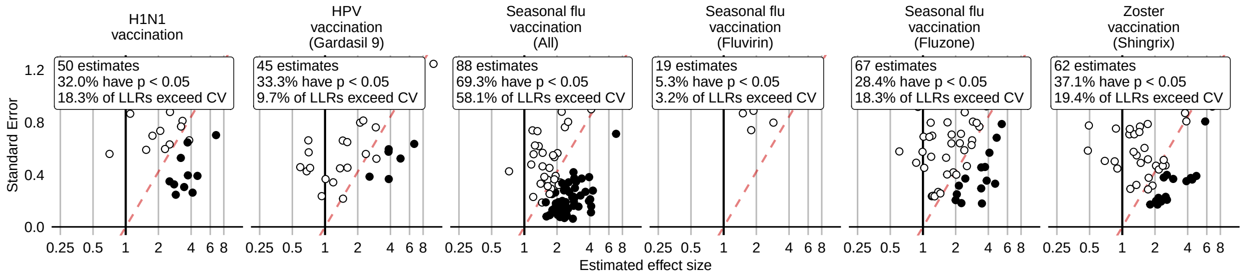

Figure 137: Negative control effect-size estimates and standard errors at the end of the study period using the CaseControl method (Age & sex adjusted, using random controls), in the Optum EHR database.

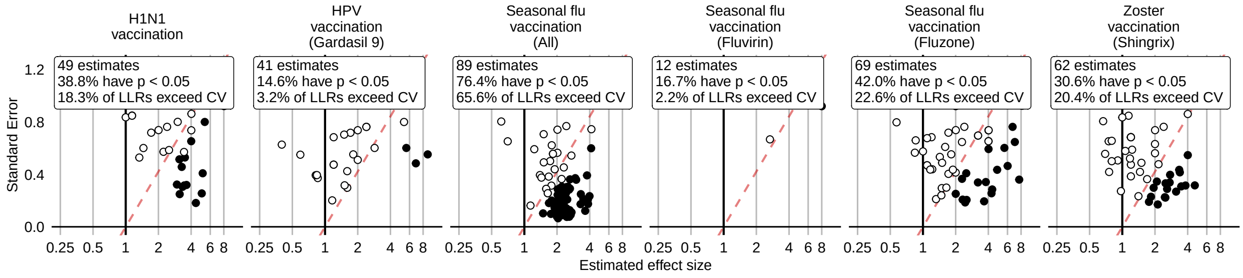

Figure 138: Negative control effect-size estimates and standard errors at the end of the study period using the CaseControl method (Age & sex matched controls), in the Optum EHR database.

14.2 Negative controls effect size estimates in MDCD

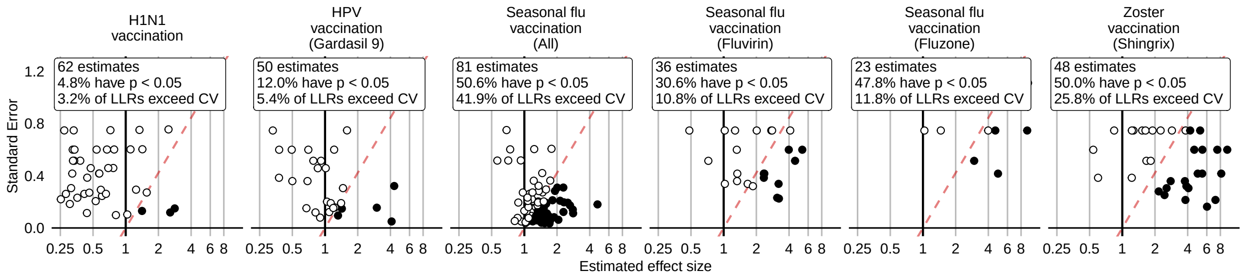

Figure 139: Negative control effect-size estimates and standard errors at the end of the study period using the HistoricalComparator method (Unadjusted, using entire historic period), in the MDCD database.

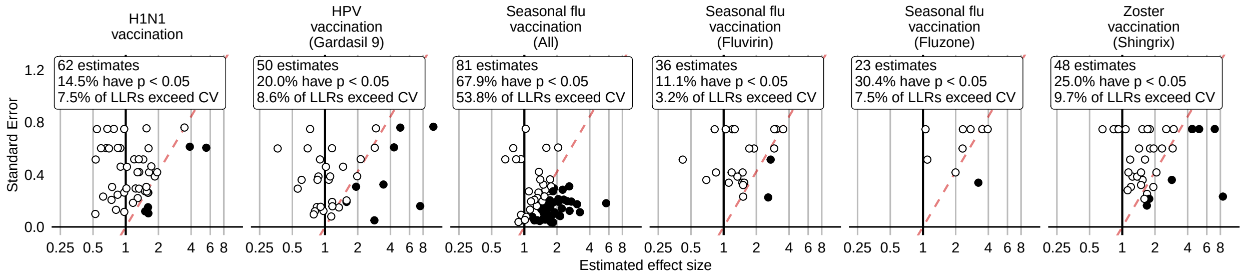

Figure 140: Negative control effect-size estimates and standard errors at the end of the study period using the HistoricalComparator method (Age & sex adjusted, using entire historic period), in the MDCD database.

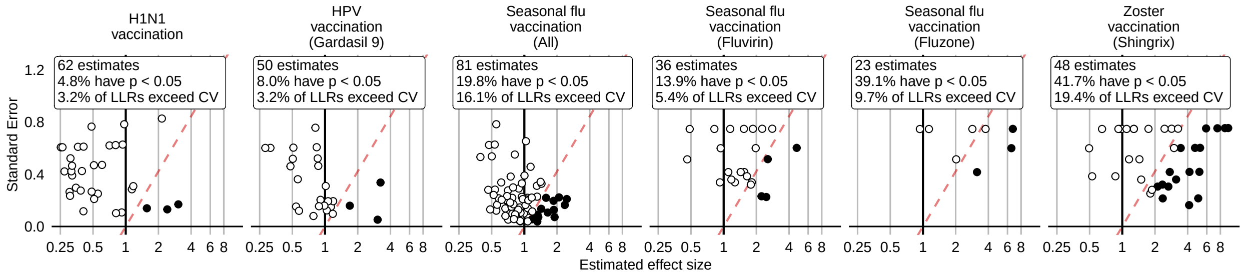

Figure 141: Negative control effect-size estimates and standard errors at the end of the study period using the HistoricalComparator method (Unadjusted, using TaR after historic visit), in the MDCD database.

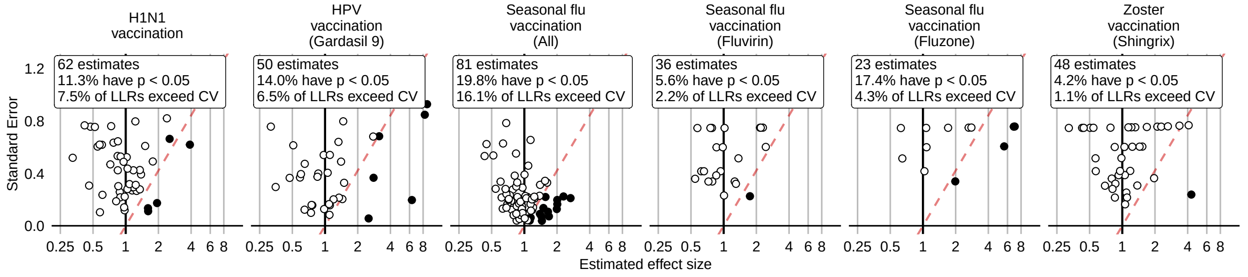

Figure 142: Negative control effect-size estimates and standard errors at the end of the study period using the HistoricalComparator method (Age & sex adjusted, using TaR after historic visit), in the MDCD database.

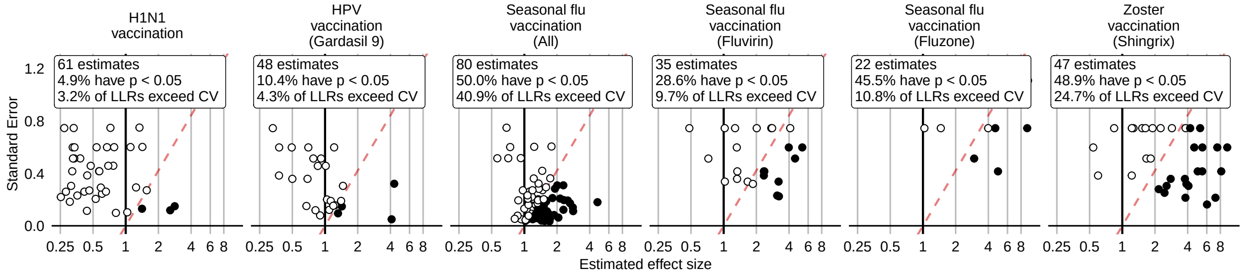

Figure 143: Negative control effect-size estimates and standard errors at the end of the study period using the HistoricalComparator method (Unadjusted, using entire historic period, filtered), in the MDCD database.

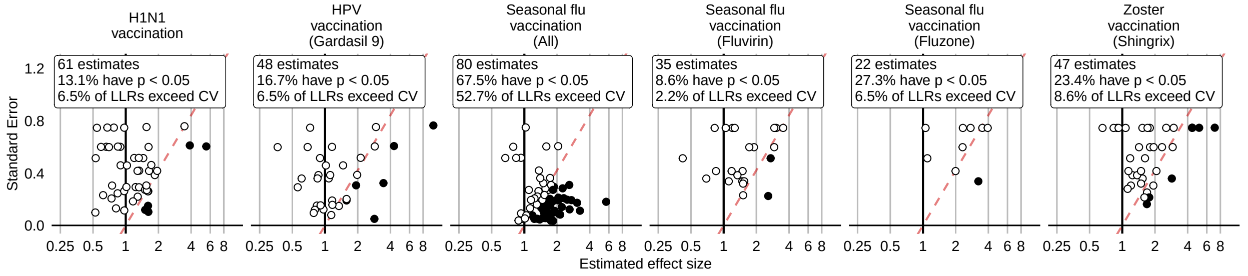

Figure 144: Negative control effect-size estimates and standard errors at the end of the study period using the HistoricalComparator method (Age & sex adjusted, using entire historic period, filtered), in the MDCD database.

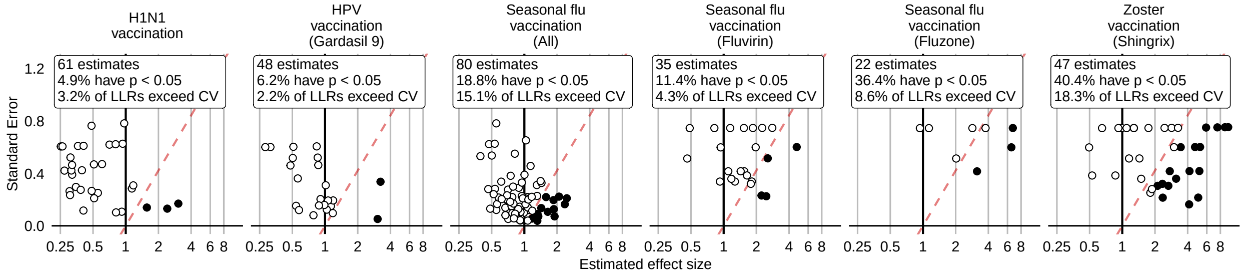

Figure 145: Negative control effect-size estimates and standard errors at the end of the study period using the HistoricalComparator method (Unadjusted, using TaR after historic visit, filtered), in the MDCD database.

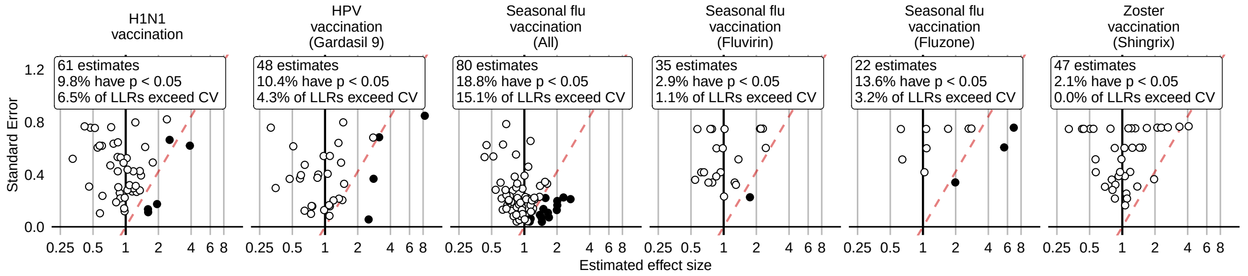

Figure 146: Negative control effect-size estimates and standard errors at the end of the study period using the HistoricalComparator method (Age & sex adjusted, using TaR after historic visit, filtered), in the MDCD database.

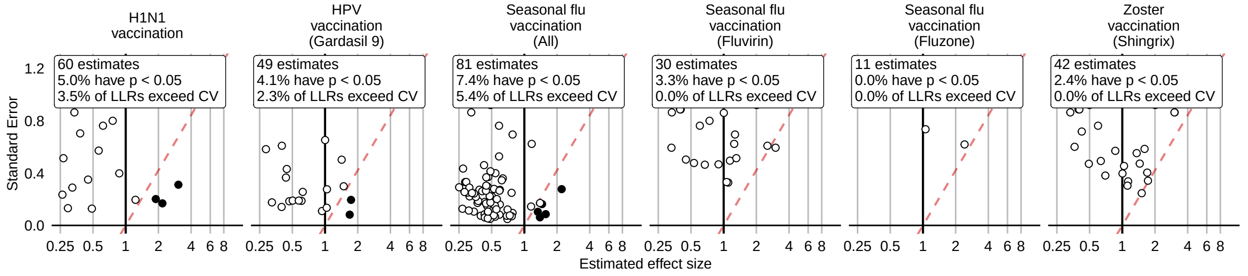

Figure 147: Negative control effect-size estimates and standard errors at the end of the study period using the CohortMethod method (Unadjusted, using outpatient visits as comparator), in the MDCD database.

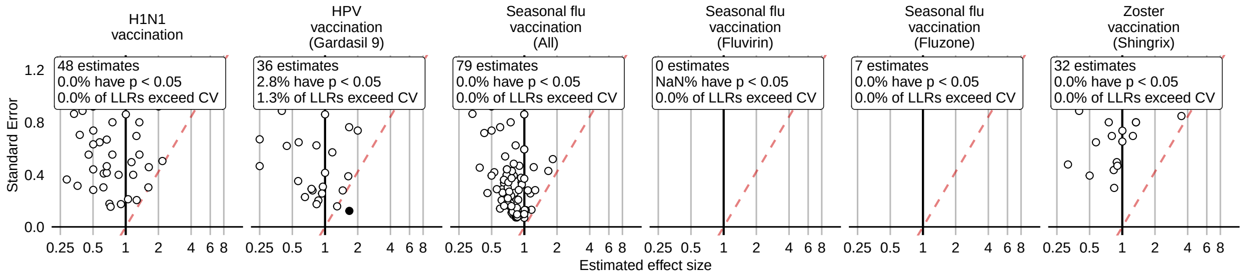

Figure 148: Negative control effect-size estimates and standard errors at the end of the study period using the CohortMethod method (PS matching, using outpatient visits as comparator), in the MDCD database.

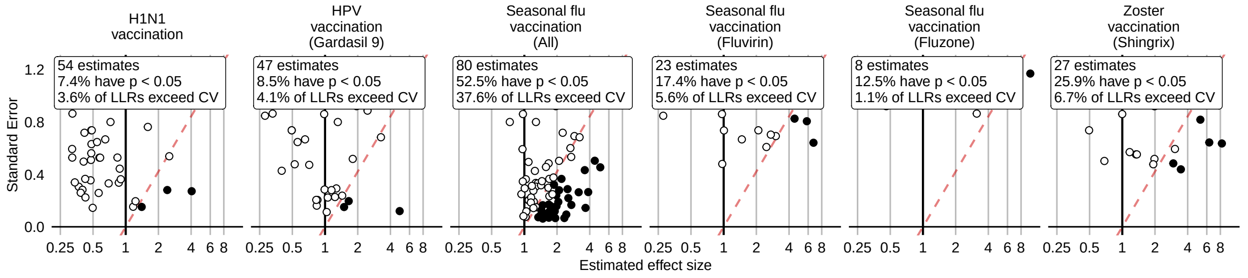

Figure 149: Negative control effect-size estimates and standard errors at the end of the study period using the CohortMethod method (Unadjusted, using random days as comparator), in the MDCD database.

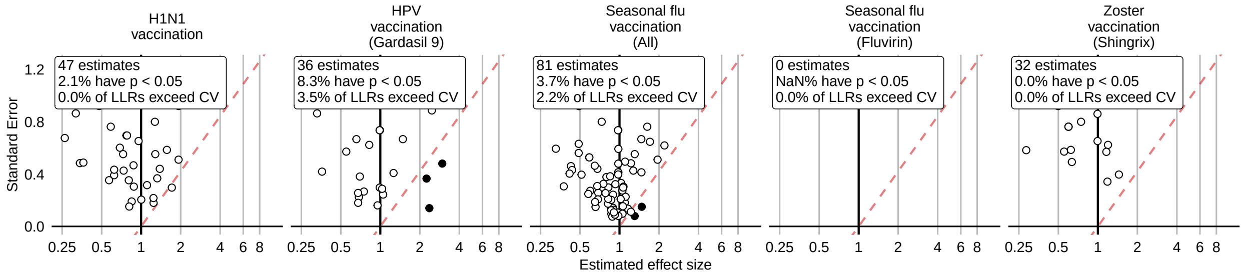

Figure 150: Negative control effect-size estimates and standard errors at the end of the study period using the CohortMethod method (PS matching, using random days as comparator), in the MDCD database.

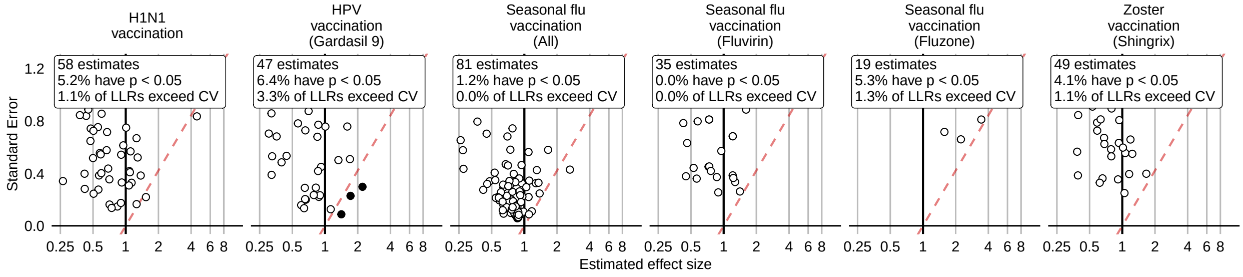

Figure 151: Negative control effect-size estimates and standard errors at the end of the study period using the CohortMethod method (PS stratification, using outpatient visits as comparator), in the MDCD database.

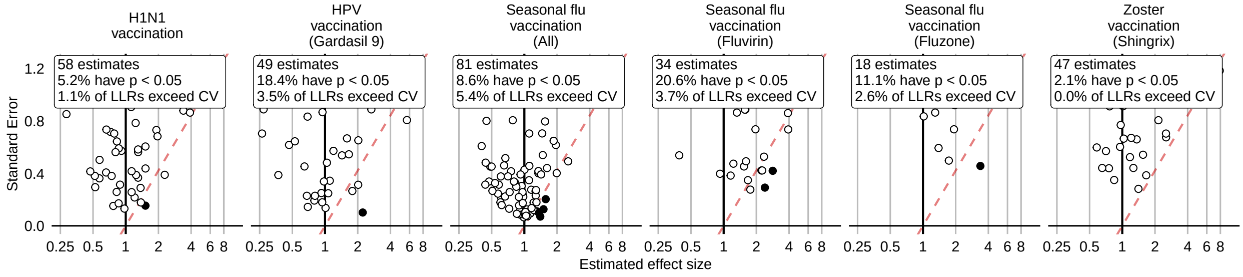

Figure 152: Negative control effect-size estimates and standard errors at the end of the study period using the CohortMethod method (PS stratification, using random days as comparator), in the MDCD database.

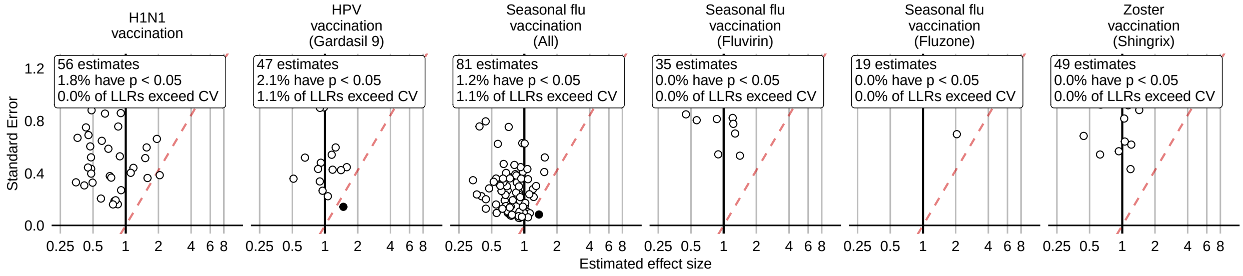

Figure 153: Negative control effect-size estimates and standard errors at the end of the study period using the CohortMethod method (PS weighting, using outpatient visits as comparator), in the MDCD database.

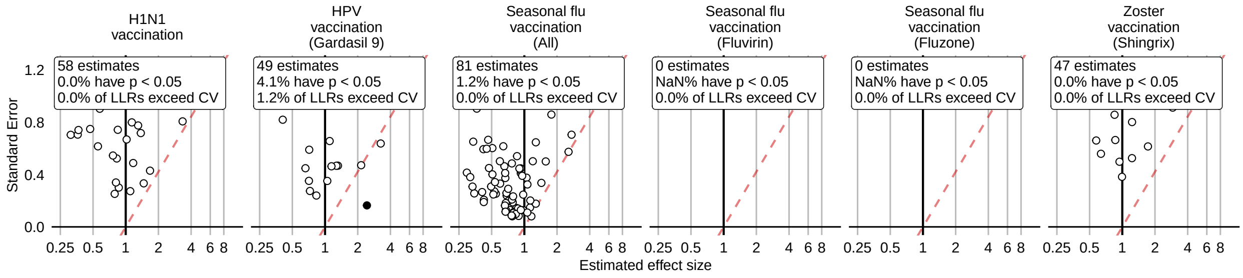

Figure 154: Negative control effect-size estimates and standard errors at the end of the study period using the CohortMethod method (PS weighting, using random days as comparator), in the MDCD database.

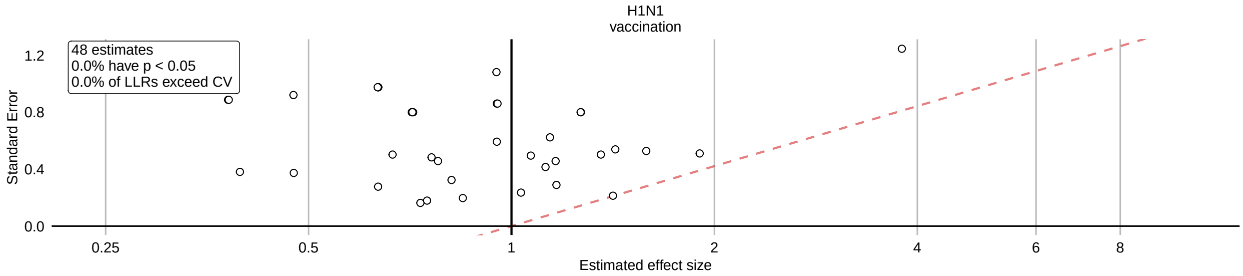

Figure 155: Negative control effect-size estimates and standard errors at the end of the study period using the CohortMethod method (Per-month PS matching, using outpatient visits as comparator), in the MDCD database.

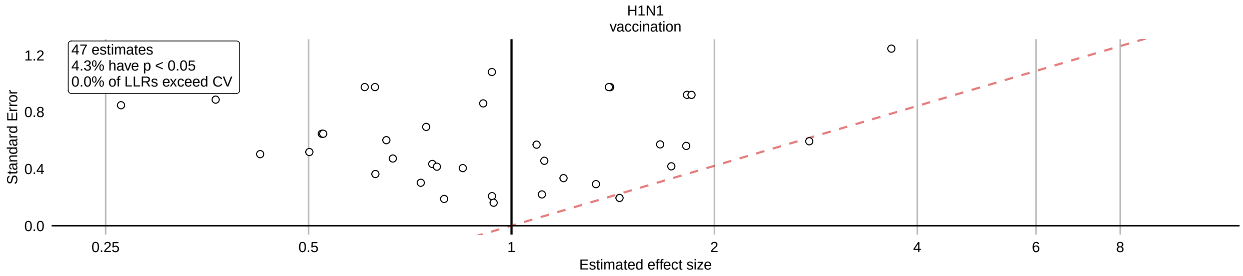

Figure 156: Negative control effect-size estimates and standard errors at the end of the study period using the CohortMethod method (Per-month PS matching, using random days as comparator), in the MDCD database.

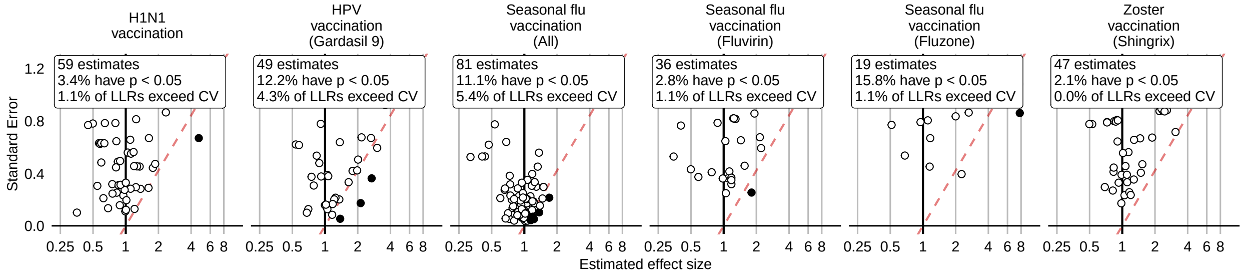

Figure 157: Negative control effect-size estimates and standard errors at the end of the study period using the SCCS method (Unadjusted SCCS excluding pre-vaccination window), in the MDCD database.

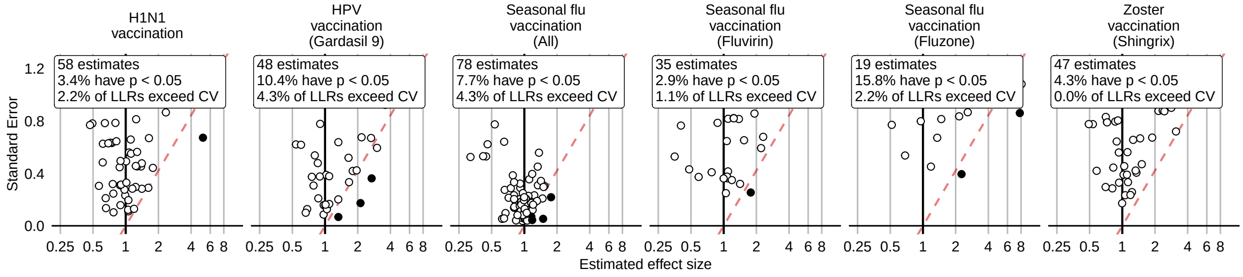

Figure 158: Negative control effect-size estimates and standard errors at the end of the study period using the SCCS method (Age & season adjusted SCCS excluding pre-vaccination window), in the MDCD database.

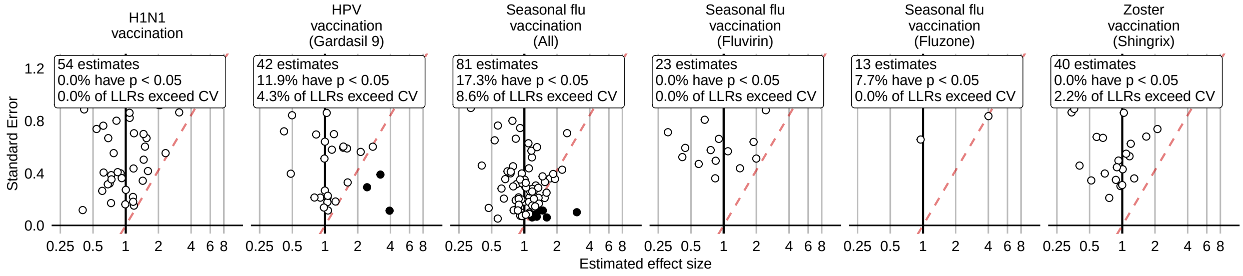

Figure 159: Negative control effect-size estimates and standard errors at the end of the study period using the SCCS method (SCRI with prior control interval), in the MDCD database.

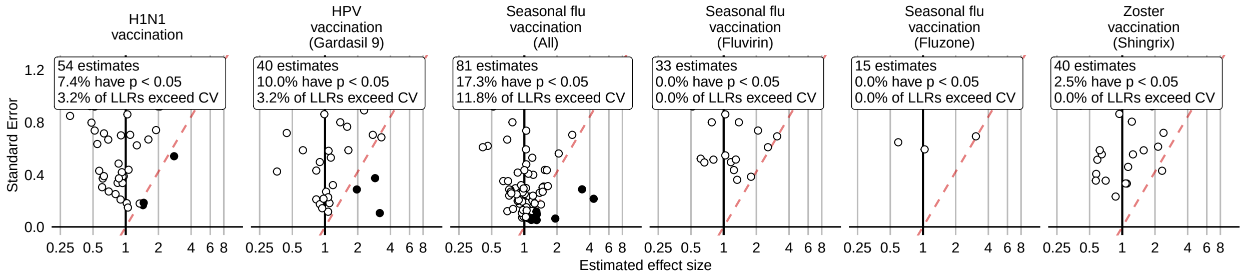

Figure 160: Negative control effect-size estimates and standard errors at the end of the study period using the SCCS method (SCRI with posterior control interval), in the MDCD database.

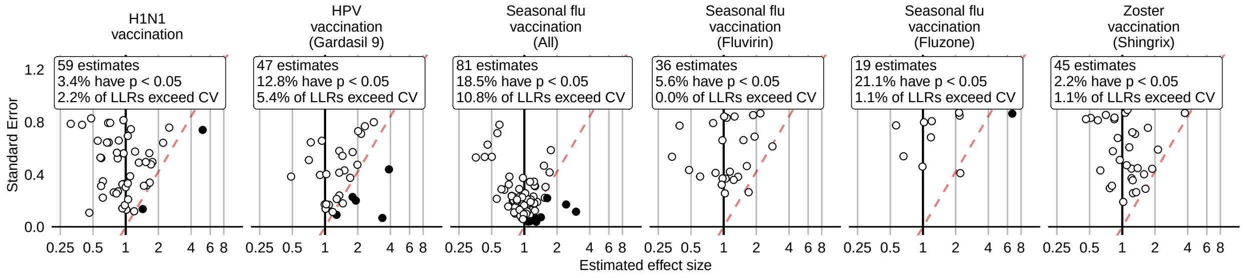

Figure 161: Negative control effect-size estimates and standard errors at the end of the study period using the SCCS method (Unadjusted SCCS excluding all pre-vaccination time), in the MDCD database.

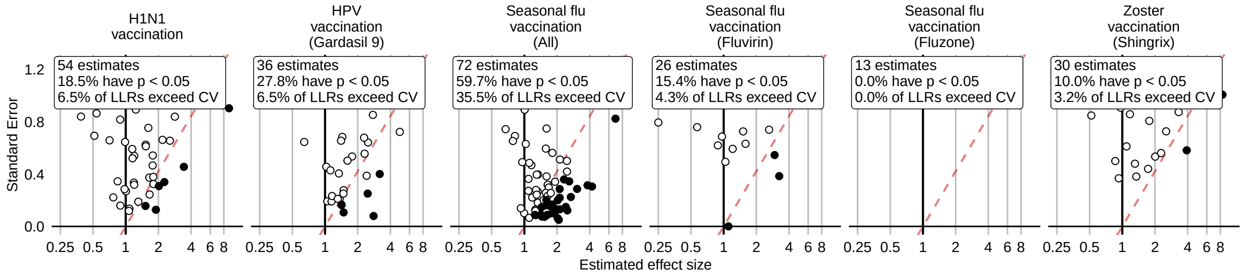

Figure 162: Negative control effect-size estimates and standard errors at the end of the study period using the CaseControl method (Age & sex adjusted, using random controls), in the MDCD database.

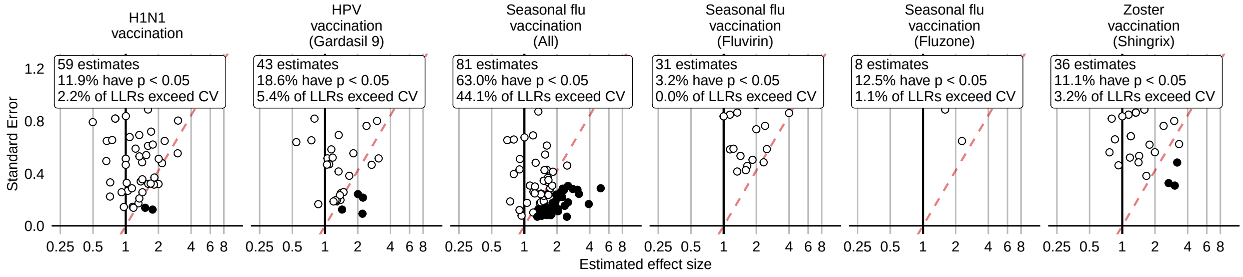

Figure 163: Negative control effect-size estimates and standard errors at the end of the study period using the CaseControl method (Age & sex matched controls), in the MDCD database.

14.3 Negative controls effect size estimates in MDCR

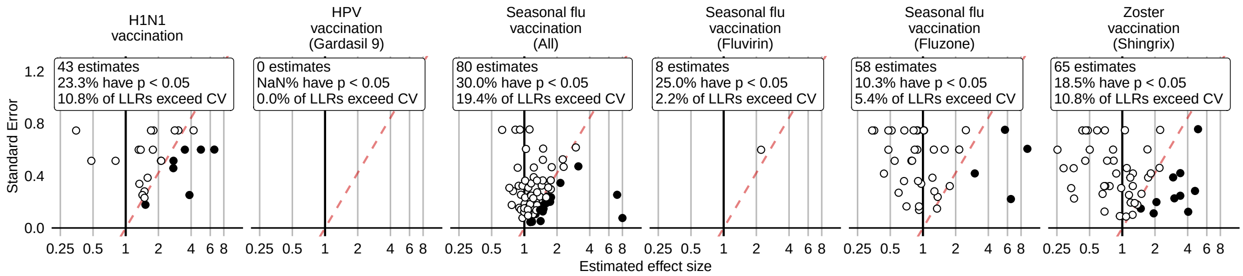

Figure 164: Negative control effect-size estimates and standard errors at the end of the study period using the HistoricalComparator method (Unadjusted, using entire historic period), in the MDCR database.

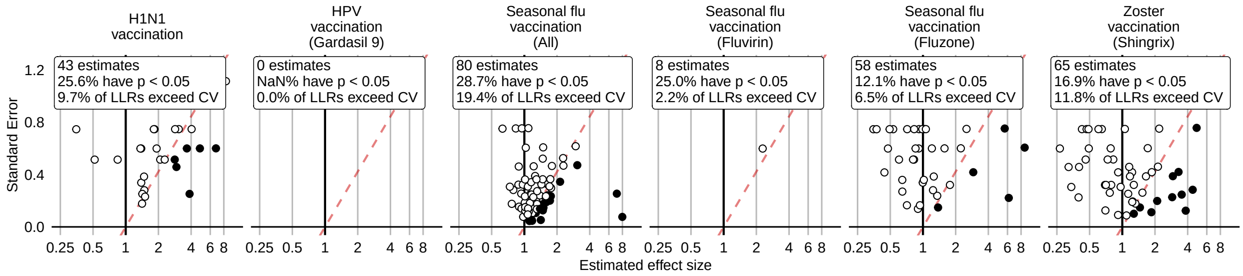

Figure 165: Negative control effect-size estimates and standard errors at the end of the study period using the HistoricalComparator method (Age & sex adjusted, using entire historic period), in the MDCR database.

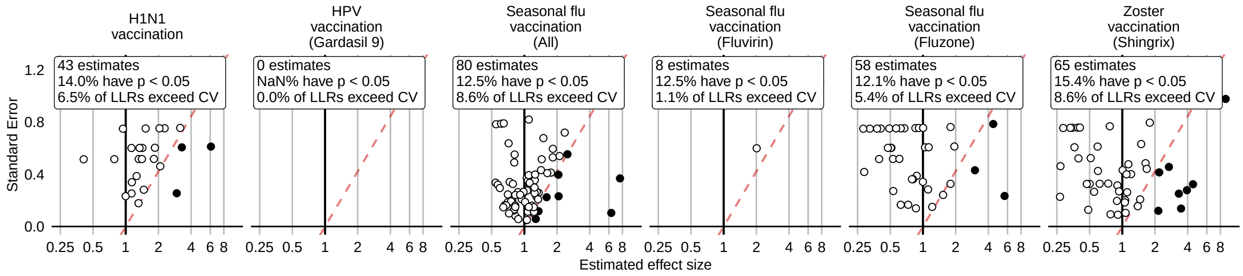

Figure 166: Negative control effect-size estimates and standard errors at the end of the study period using the HistoricalComparator method (Unadjusted, using TaR after historic visit), in the MDCR database.

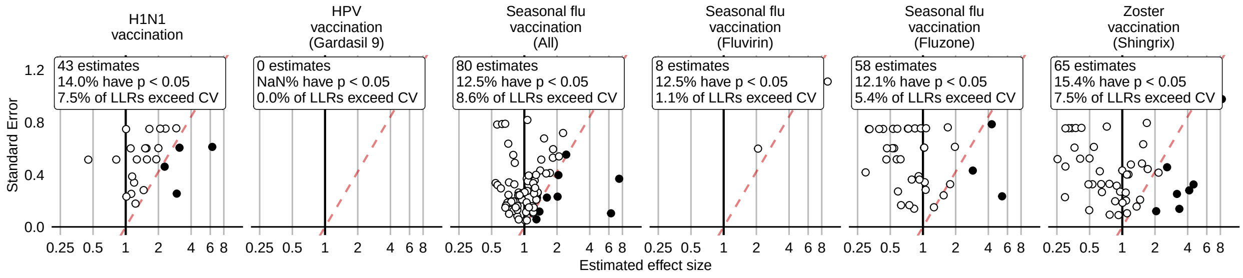

Figure 167: Negative control effect-size estimates and standard errors at the end of the study period using the HistoricalComparator method (Age & sex adjusted, using TaR after historic visit), in the MDCR database.

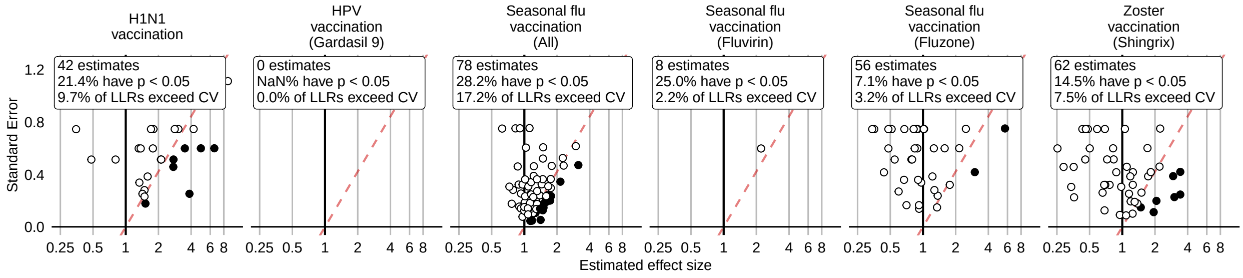

Figure 168: Negative control effect-size estimates and standard errors at the end of the study period using the HistoricalComparator method (Unadjusted, using entire historic period, filtered), in the MDCR database.

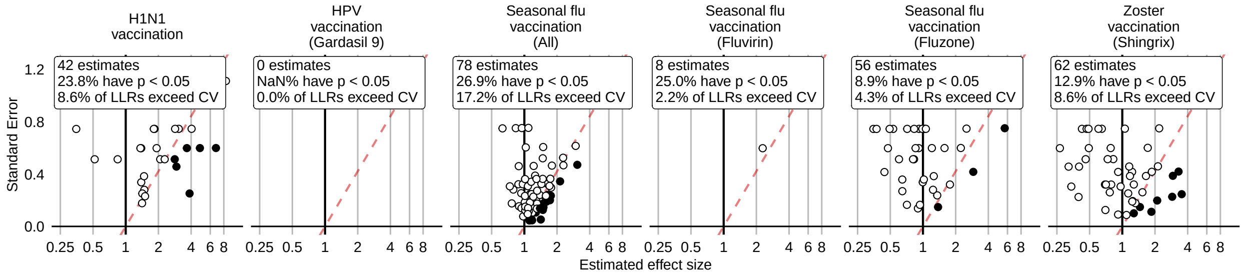

Figure 169: Negative control effect-size estimates and standard errors at the end of the study period using the HistoricalComparator method (Age & sex adjusted, using entire historic period, filtered), in the MDCR database.

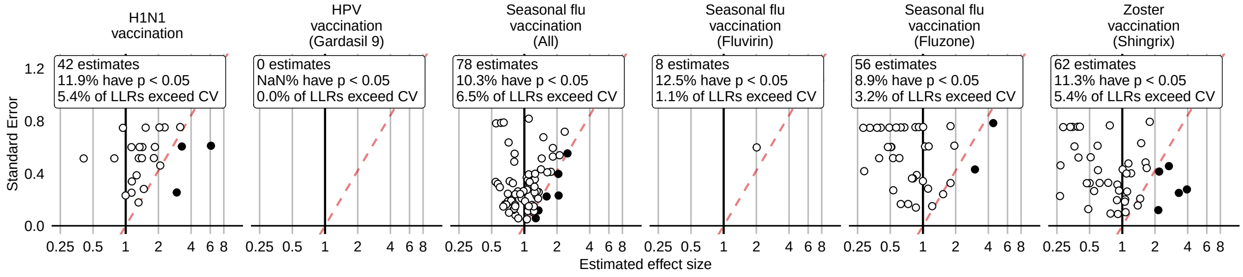

Figure 170: Negative control effect-size estimates and standard errors at the end of the study period using the HistoricalComparator method (Unadjusted, using TaR after historic visit, filtered), in the MDCR database.

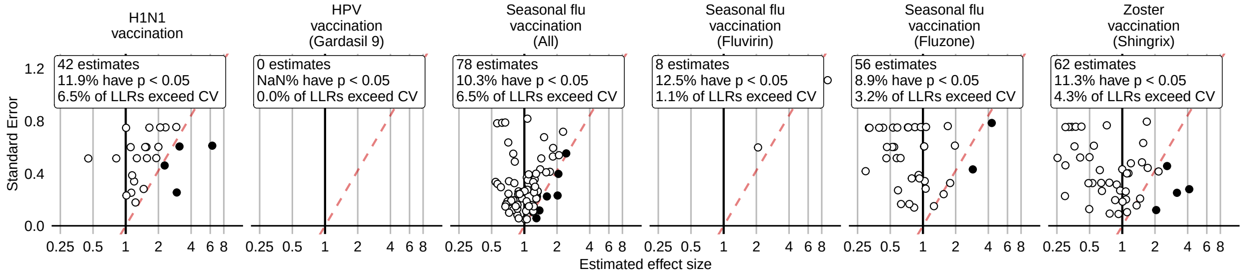

Figure 171: Negative control effect-size estimates and standard errors at the end of the study period using the HistoricalComparator method (Age & sex adjusted, using TaR after historic visit, filtered), in the MDCR database.

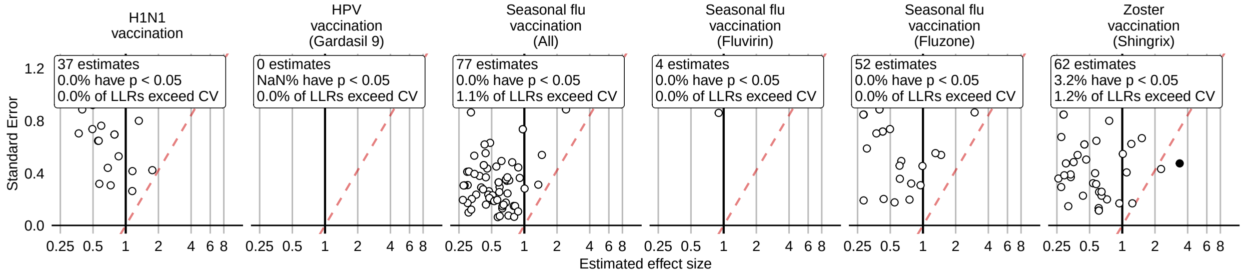

Figure 172: Negative control effect-size estimates and standard errors at the end of the study period using the CohortMethod method (Unadjusted, using outpatient visits as comparator), in the MDCR database.

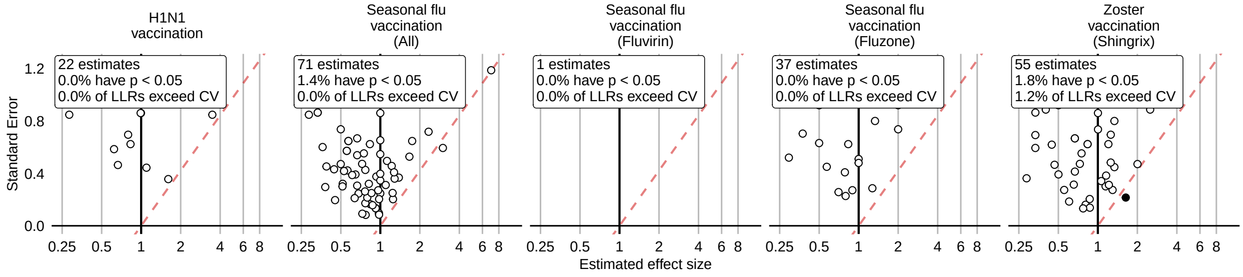

Figure 173: Negative control effect-size estimates and standard errors at the end of the study period using the CohortMethod method (PS matching, using outpatient visits as comparator), in the MDCR database.

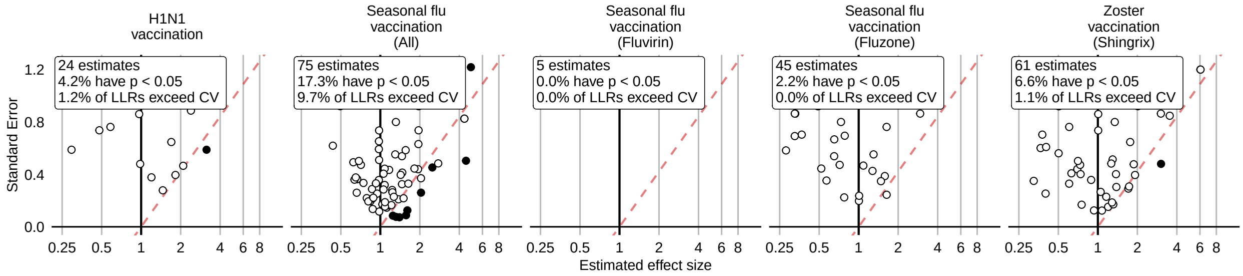

Figure 174: Negative control effect-size estimates and standard errors at the end of the study period using the CohortMethod method (Unadjusted, using random days as comparator), in the MDCR database.

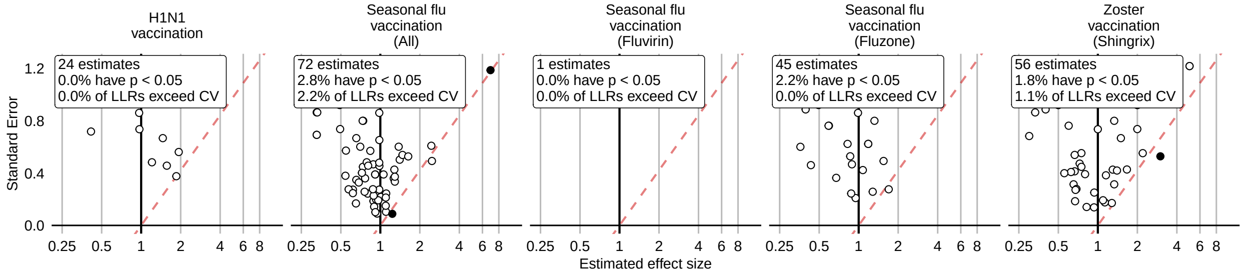

Figure 175: Negative control effect-size estimates and standard errors at the end of the study period using the CohortMethod method (PS matching, using random days as comparator), in the MDCR database.

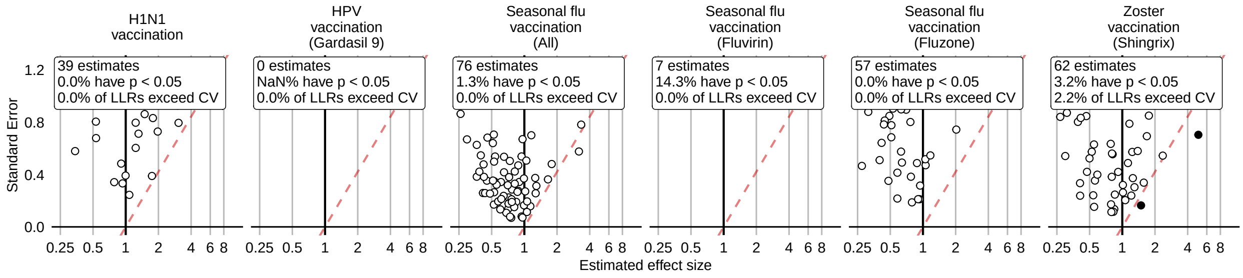

Figure 176: Negative control effect-size estimates and standard errors at the end of the study period using the CohortMethod method (PS stratification, using outpatient visits as comparator), in the MDCR database.

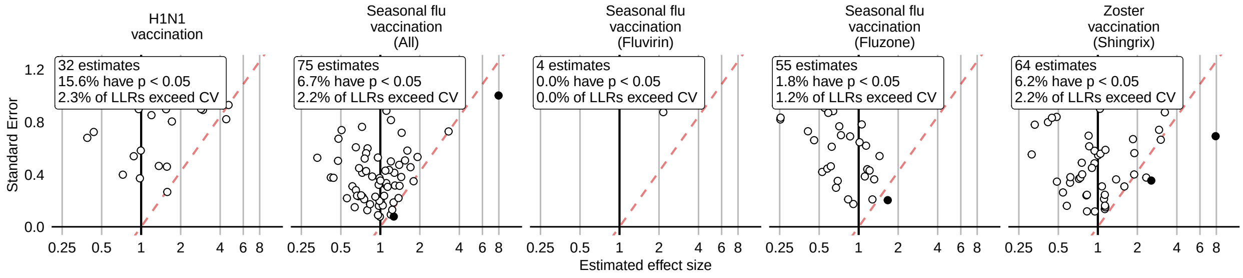

Figure 177: Negative control effect-size estimates and standard errors at the end of the study period using the CohortMethod method (PS stratification, using random days as comparator), in the MDCR database.

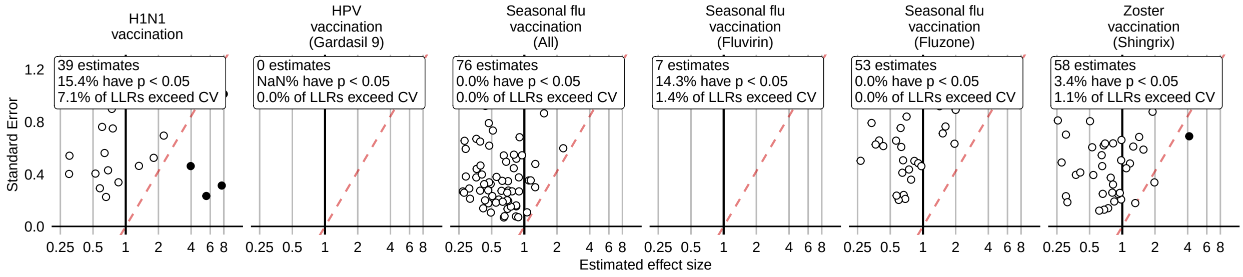

Figure 178: Negative control effect-size estimates and standard errors at the end of the study period using the CohortMethod method (PS weighting, using outpatient visits as comparator), in the MDCR database.

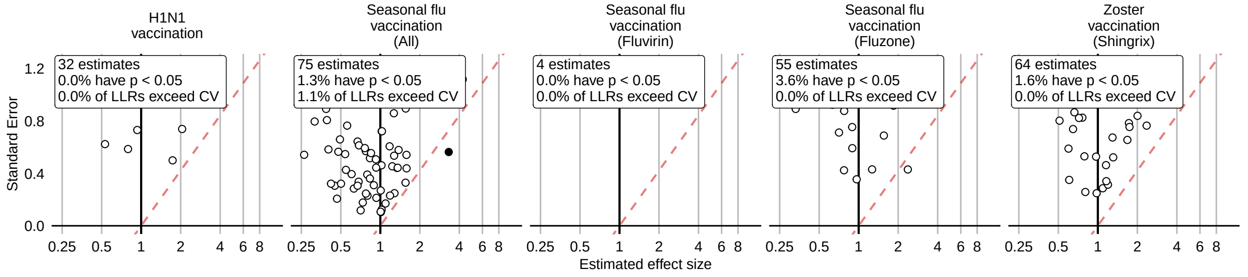

Figure 179: Negative control effect-size estimates and standard errors at the end of the study period using the CohortMethod method (PS weighting, using random days as comparator), in the MDCR database.

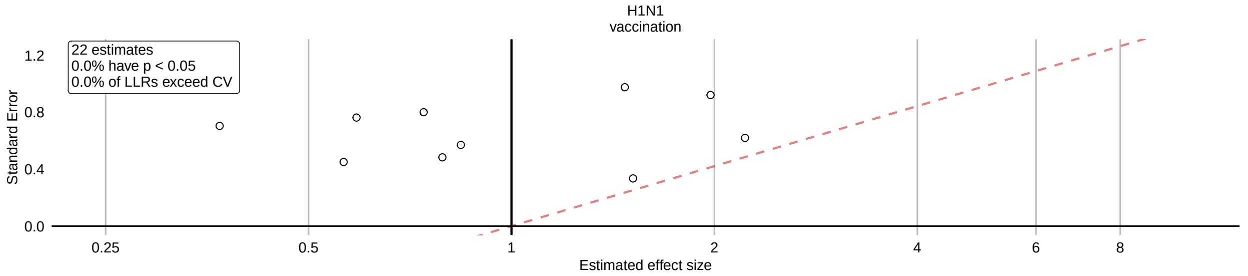

Figure 180: Negative control effect-size estimates and standard errors at the end of the study period using the CohortMethod method (Per-month PS matching, using outpatient visits as comparator), in the MDCR database.

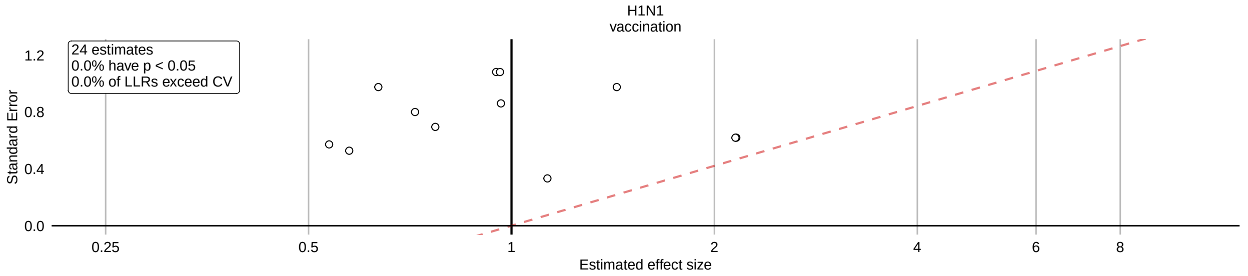

Figure 181: Negative control effect-size estimates and standard errors at the end of the study period using the CohortMethod method (Per-month PS matching, using random days as comparator), in the MDCR database.

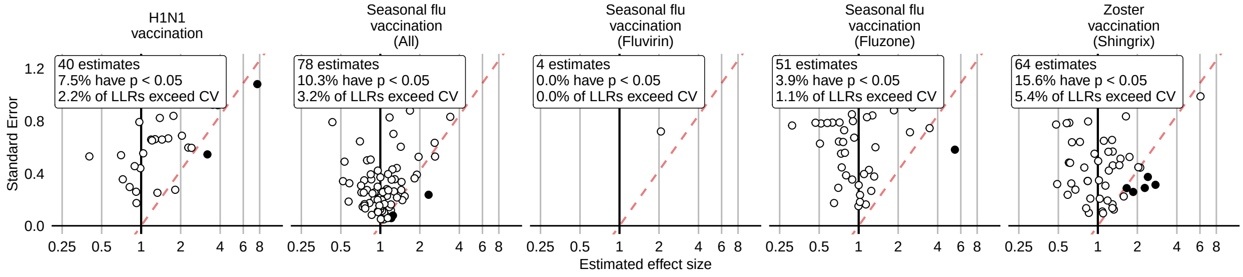

Figure 182: Negative control effect-size estimates and standard errors at the end of the study period using the SCCS method (Unadjusted SCCS excluding pre-vaccination window), in the MDCR database.

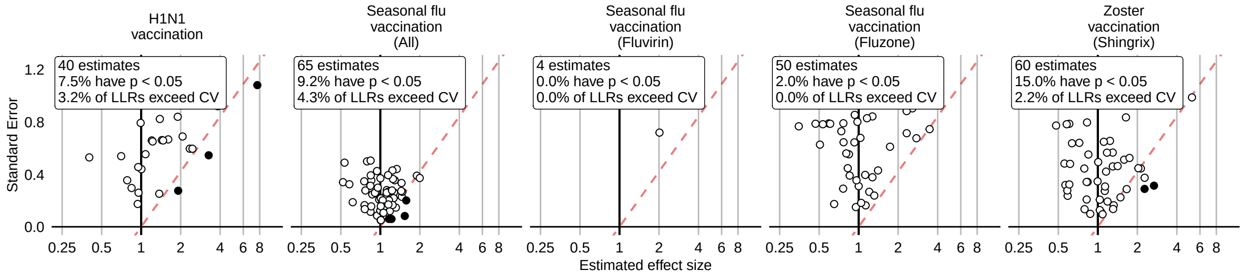

Figure 183: Negative control effect-size estimates and standard errors at the end of the study period using the SCCS method (Age & season adjusted SCCS excluding pre-vaccination window), in the MDCR database.

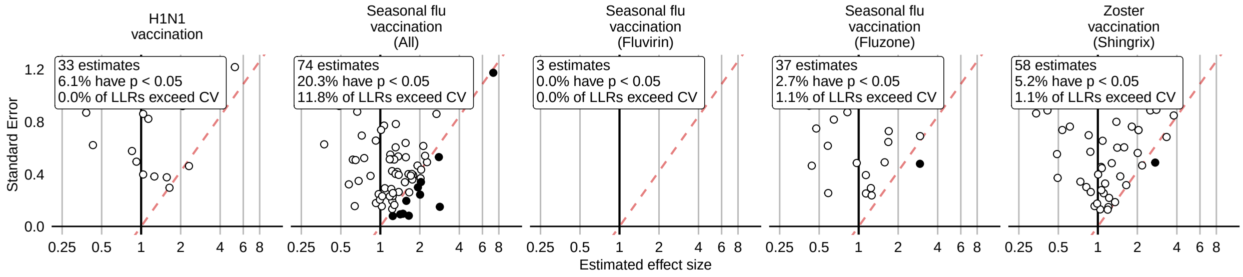

Figure 184: Negative control effect-size estimates and standard errors at the end of the study period using the SCCS method (SCRI with prior control interval), in the MDCR database.

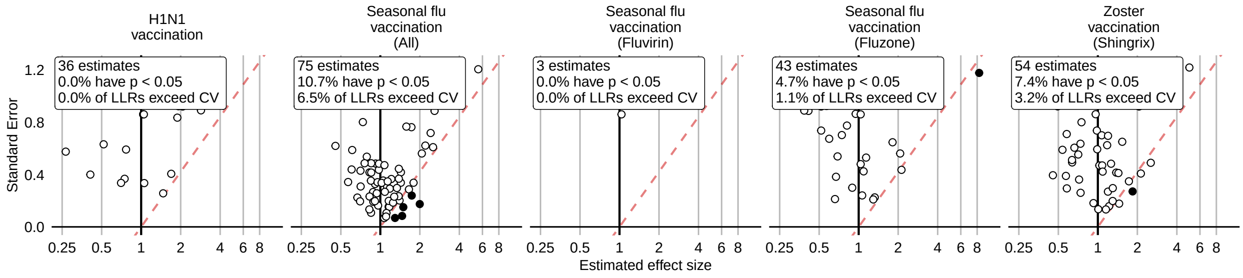

Figure 185: Negative control effect-size estimates and standard errors at the end of the study period using the SCCS method (SCRI with posterior control interval), in the MDCR database.

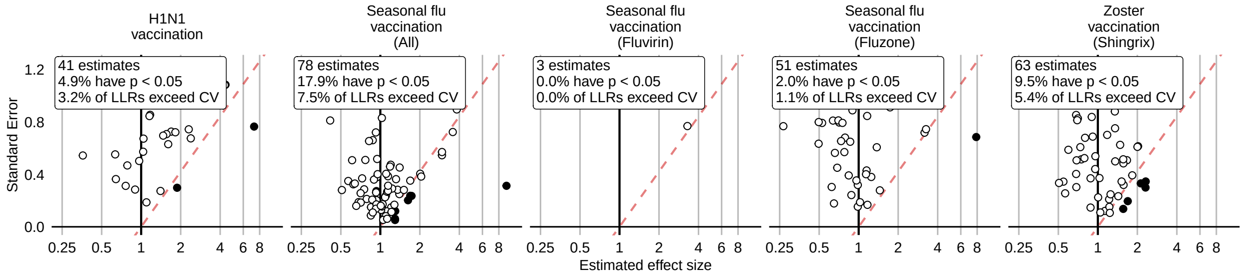

Figure 186: Negative control effect-size estimates and standard errors at the end of the study period using the SCCS method (Unadjusted SCCS excluding all pre-vaccination time), in the MDCR database.

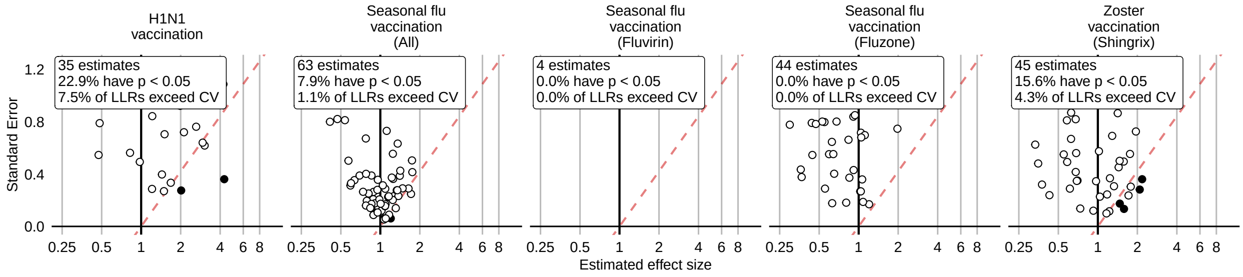

Figure 187: Negative control effect-size estimates and standard errors at the end of the study period using the CaseControl method (Age & sex adjusted, using random controls), in the MDCR database.

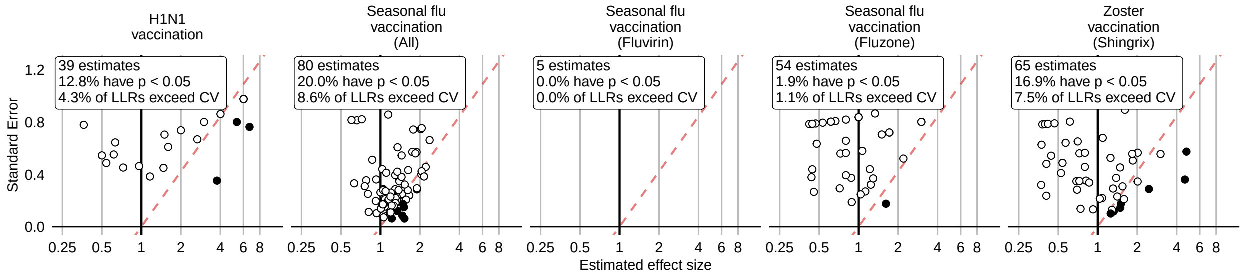

Figure 188: Negative control effect-size estimates and standard errors at the end of the study period using the CaseControl method (Age & sex matched controls), in the MDCR database.

14.4 Negative controls effect size estimates in CCAE

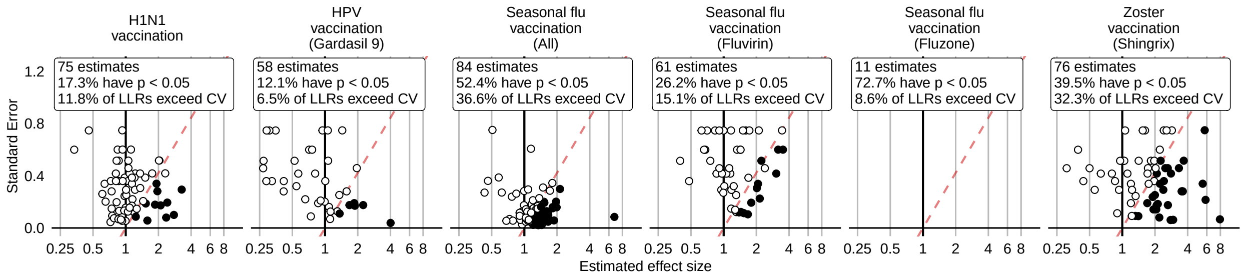

Figure 189: Negative control effect-size estimates and standard errors at the end of the study period using the HistoricalComparator method (Unadjusted, using entire historic period), in the CCAE database.

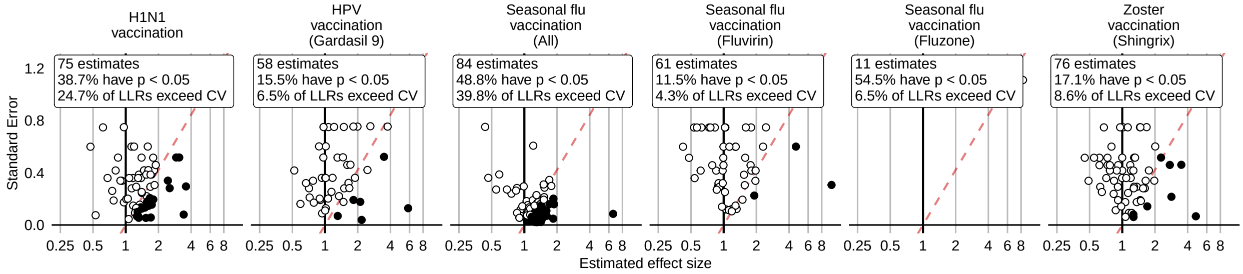

Figure 190: Negative control effect-size estimates and standard errors at the end of the study period using the HistoricalComparator method (Age & sex adjusted, using entire historic period), in the CCAE database.

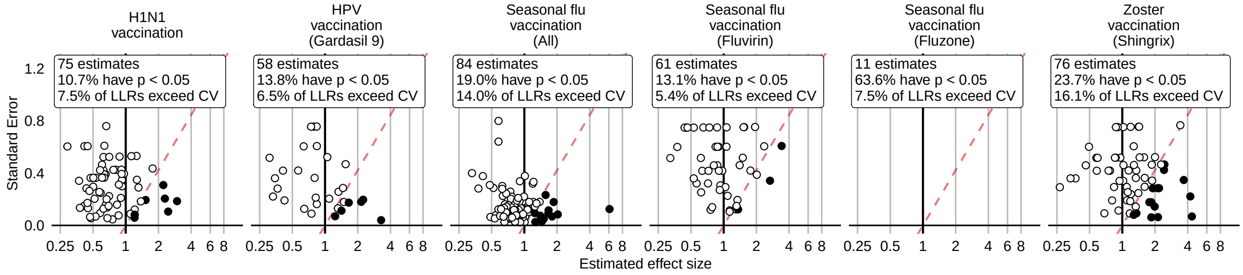

Figure 191: Negative control effect-size estimates and standard errors at the end of the study period using the HistoricalComparator method (Unadjusted, using TaR after historic visit), in the CCAE database.

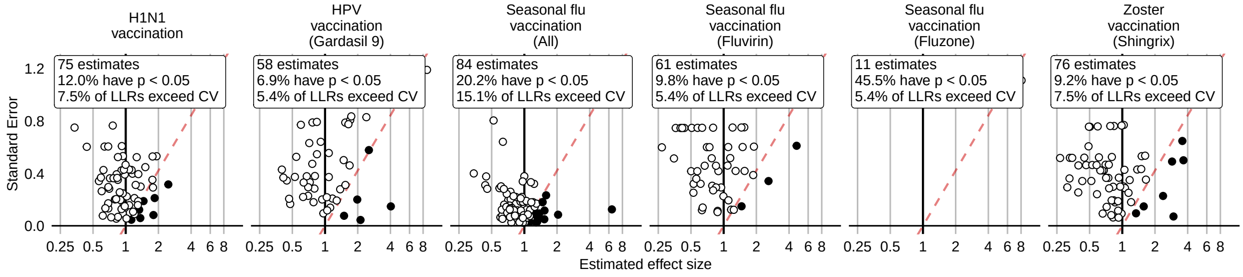

Figure 192: Negative control effect-size estimates and standard errors at the end of the study period using the HistoricalComparator method (Age & sex adjusted, using TaR after historic visit), in the CCAE database.

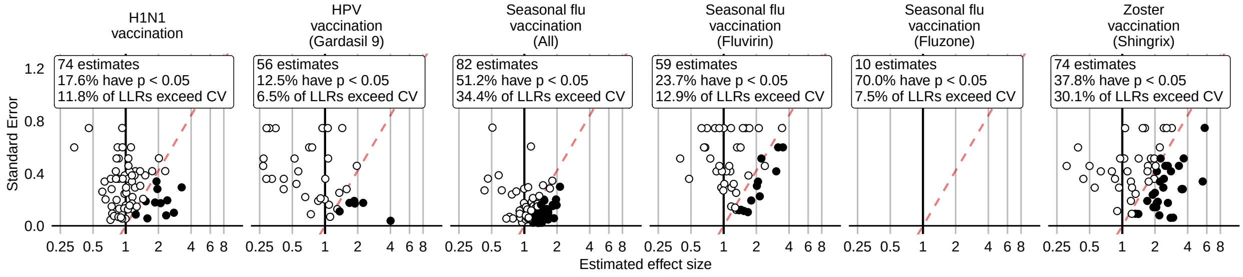

Figure 193: Negative control effect-size estimates and standard errors at the end of the study period using the HistoricalComparator method (Unadjusted, using entire historic period, filtered), in the CCAE database.

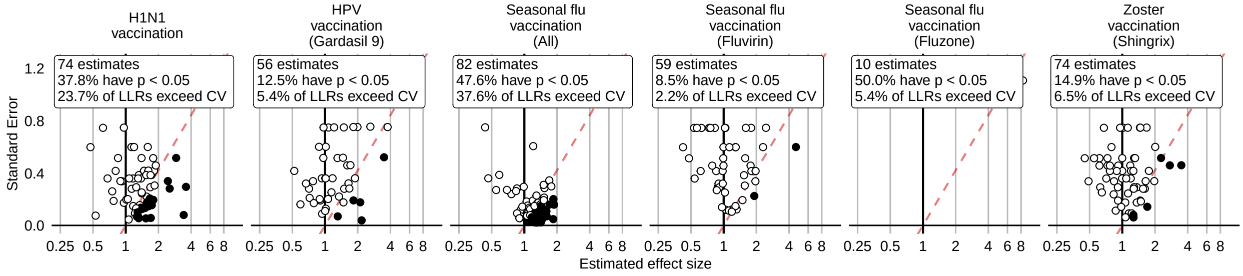

Figure 194: Negative control effect-size estimates and standard errors at the end of the study period using the HistoricalComparator method (Age & sex adjusted, using entire historic period, filtered), in the CCAE database.

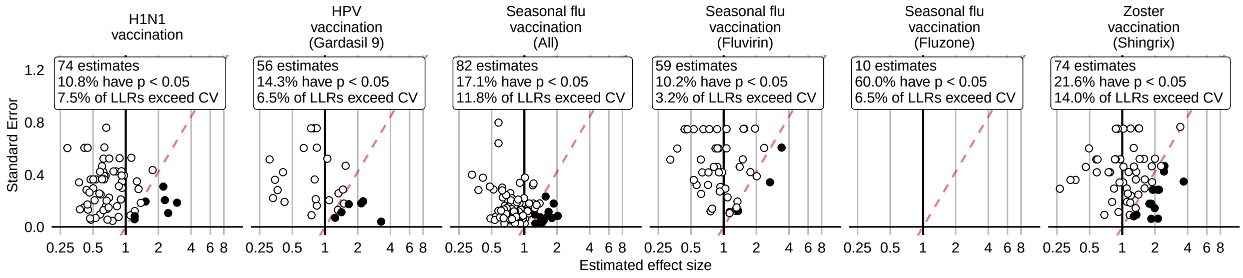

Figure 195: Negative control effect-size estimates and standard errors at the end of the study period using the HistoricalComparator method (Unadjusted, using TaR after historic visit, filtered), in the CCAE database.

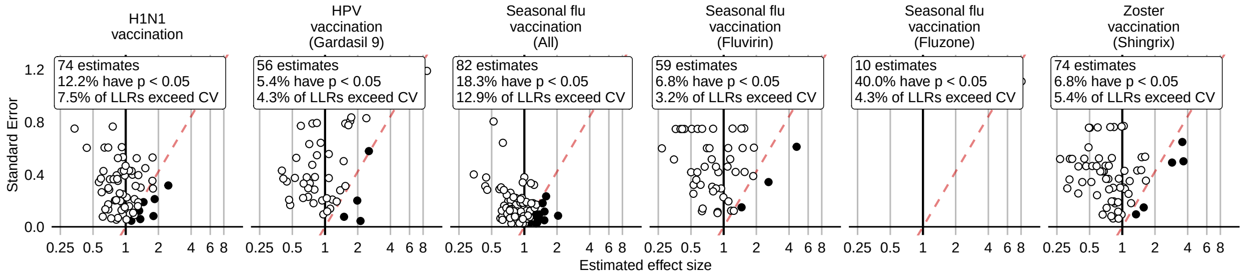

Figure 196: Negative control effect-size estimates and standard errors at the end of the study period using the HistoricalComparator method (Age & sex adjusted, using TaR after historic visit, filtered), in the CCAE database.

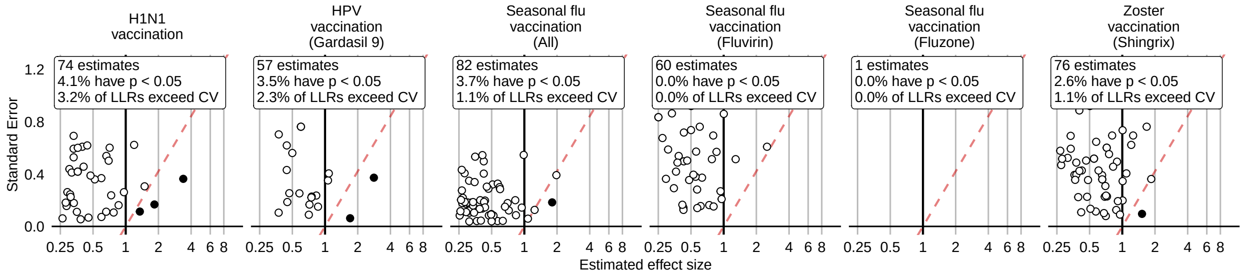

Figure 197: Negative control effect-size estimates and standard errors at the end of the study period using the CohortMethod method (Unadjusted, using outpatient visits as comparator), in the CCAE database.

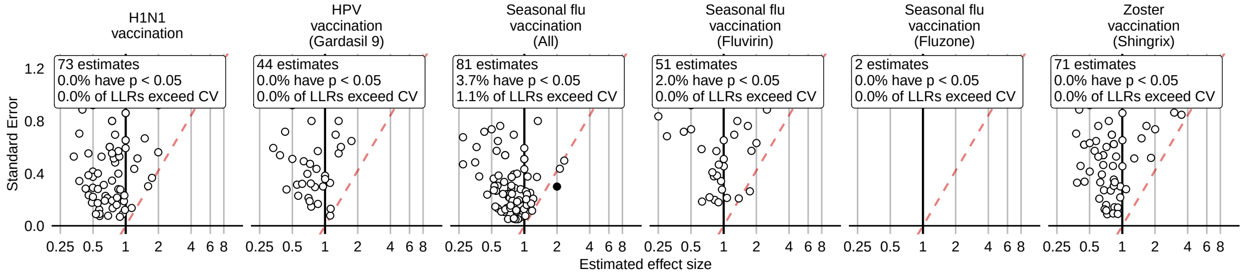

Figure 198: Negative control effect-size estimates and standard errors at the end of the study period using the CohortMethod method (PS matching, using outpatient visits as comparator), in the CCAE database.

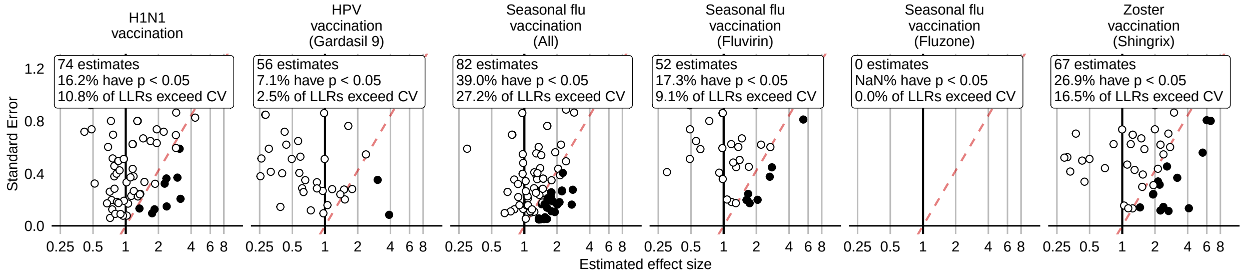

Figure 199: Negative control effect-size estimates and standard errors at the end of the study period using the CohortMethod method (Unadjusted, using random days as comparator), in the CCAE database.

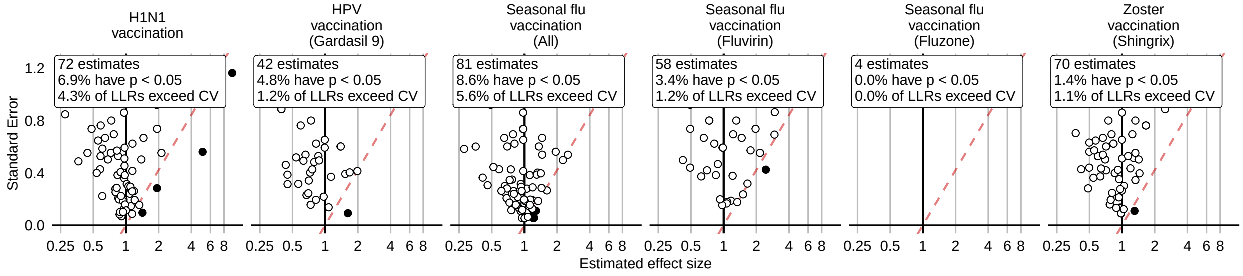

Figure 200: Negative control effect-size estimates and standard errors at the end of the study period using the CohortMethod method (PS matching, using random days as comparator), in the CCAE database.

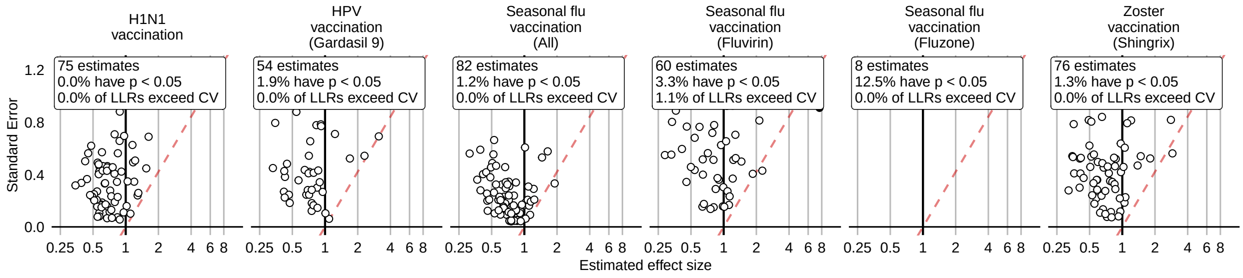

Figure 201: Negative control effect-size estimates and standard errors at the end of the study period using the CohortMethod method (PS stratification, using outpatient visits as comparator), in the CCAE database.

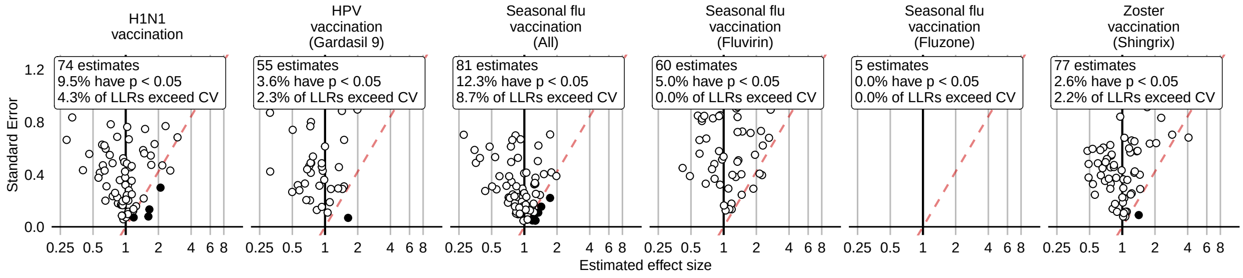

Figure 202: Negative control effect-size estimates and standard errors at the end of the study period using the CohortMethod method (PS stratification, using random days as comparator), in the CCAE database.

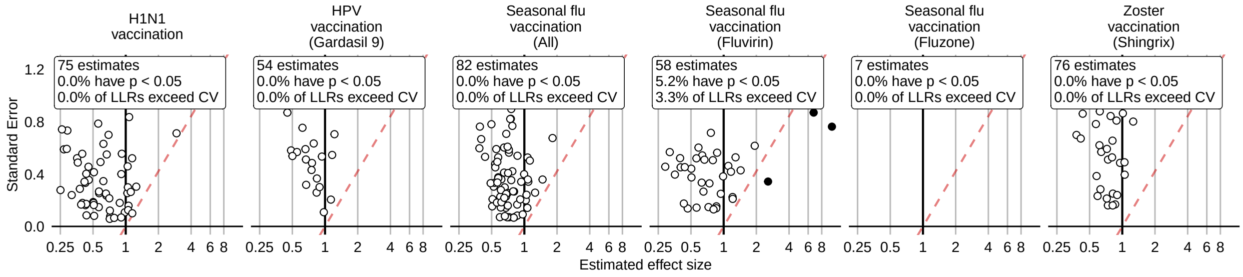

Figure 203: Negative control effect-size estimates and standard errors at the end of the study period using the CohortMethod method (PS weighting, using outpatient visits as comparator), in the CCAE database.

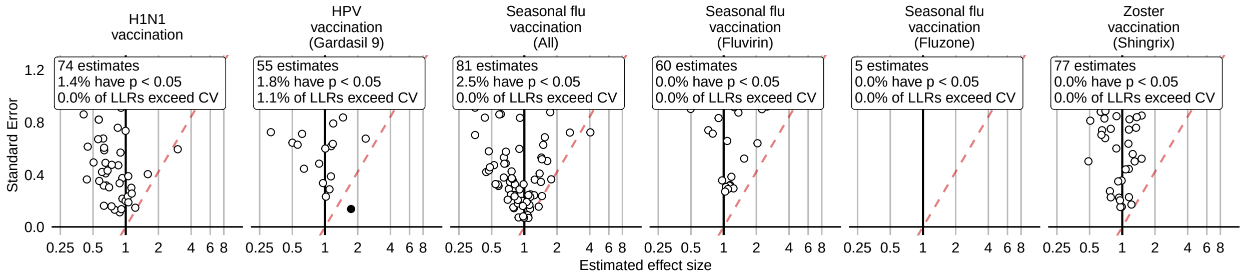

Figure 204: Negative control effect-size estimates and standard errors at the end of the study period using the CohortMethod method (PS weighting, using random days as comparator), in the CCAE database.

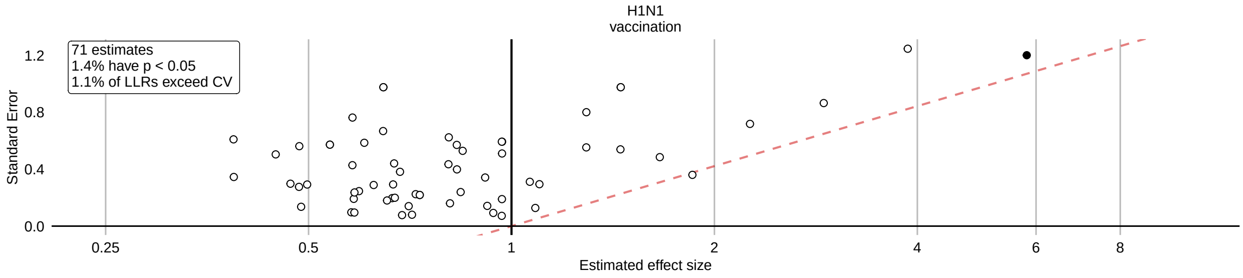

Figure 205: Negative control effect-size estimates and standard errors at the end of the study period using the CohortMethod method (Per-month PS matching, using outpatient visits as comparator), in the CCAE database.

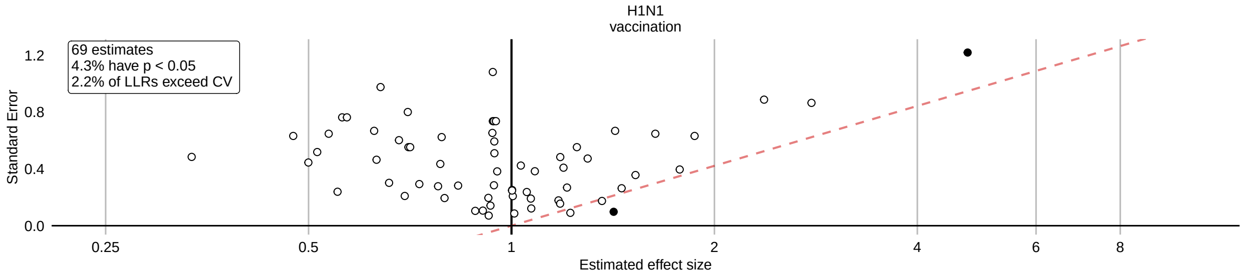

Figure 206: Negative control effect-size estimates and standard errors at the end of the study period using the CohortMethod method (Per-month PS matching, using random days as comparator), in the CCAE database.

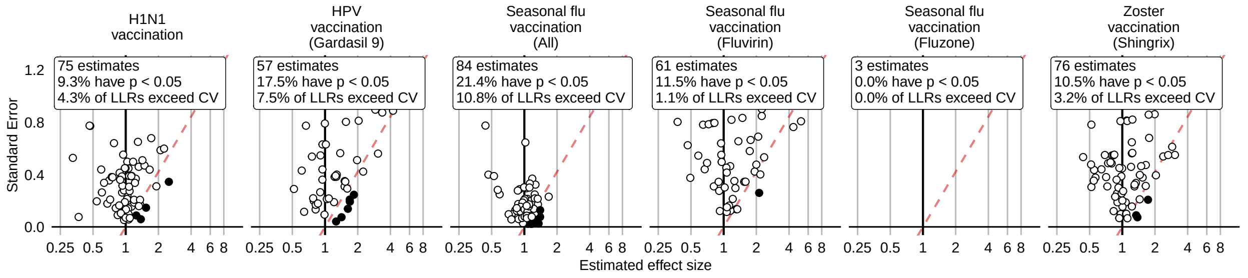

Figure 207: Negative control effect-size estimates and standard errors at the end of the study period using the SCCS method (Unadjusted SCCS excluding pre-vaccination window), in the CCAE database.

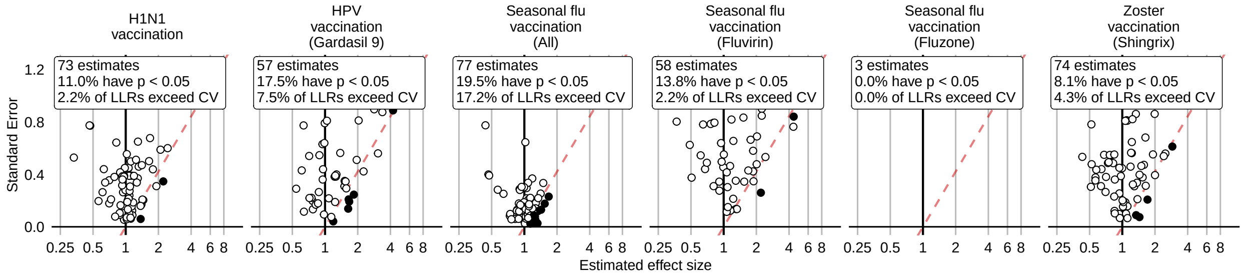

Figure 208: Negative control effect-size estimates and standard errors at the end of the study period using the SCCS method (Age & season adjusted SCCS excluding pre-vaccination window), in the CCAE database.

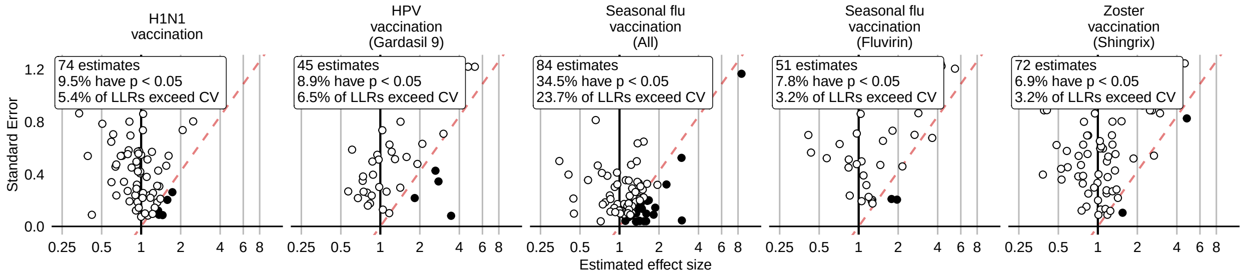

Figure 209: Negative control effect-size estimates and standard errors at the end of the study period using the SCCS method (SCRI with prior control interval), in the CCAE database.

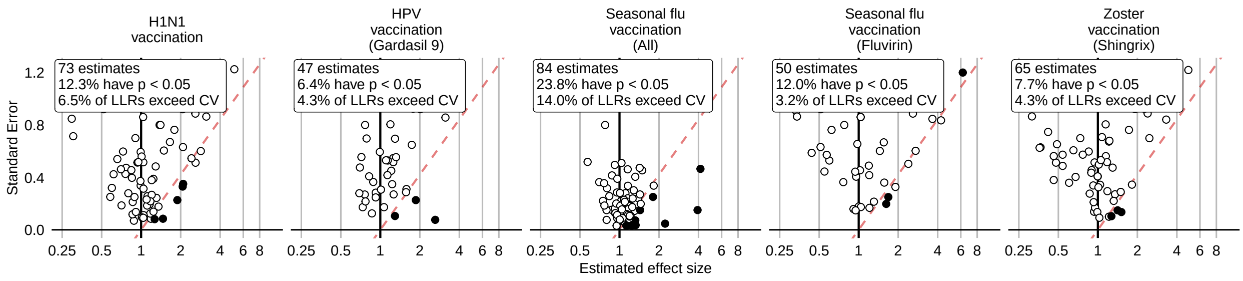

Figure 210: Negative control effect-size estimates and standard errors at the end of the study period using the SCCS method (SCRI with posterior control interval), in the CCAE database.

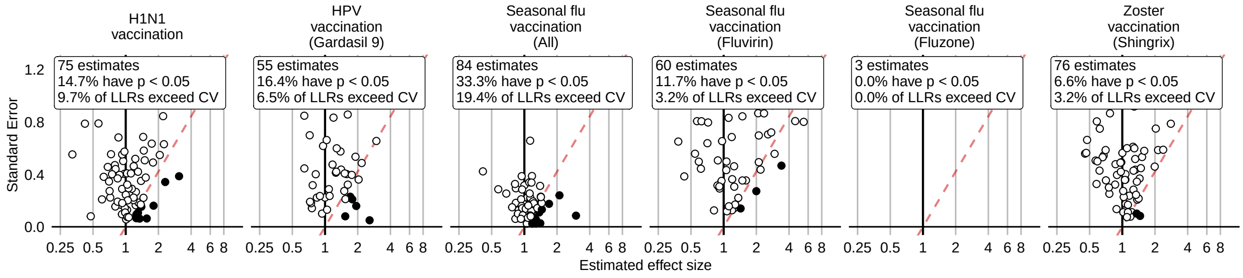

Figure 211: Negative control effect-size estimates and standard errors at the end of the study period using the SCCS method (Unadjusted SCCS excluding all pre-vaccination time), in the CCAE database.

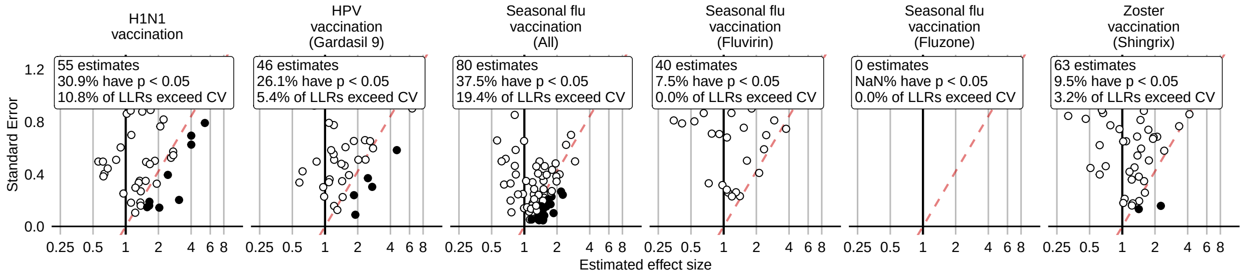

Figure 212: Negative control effect-size estimates and standard errors at the end of the study period using the CaseControl method (Age & sex adjusted, using random controls), in the CCAE database.

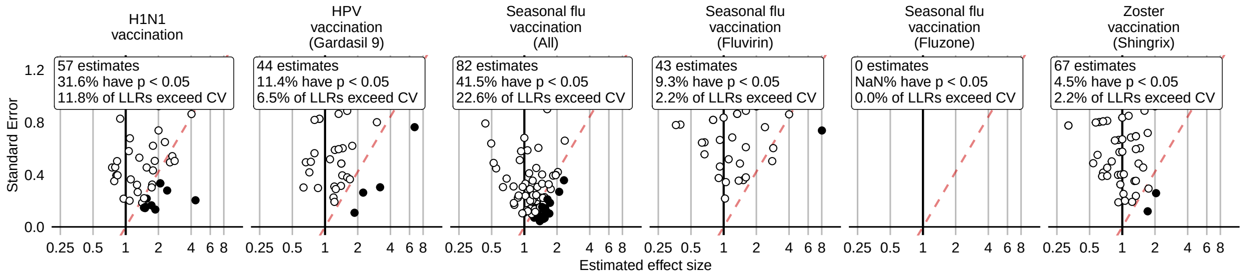

Figure 213: Negative control effect-size estimates and standard errors at the end of the study period using the CaseControl method (Age & sex matched controls), in the CCAE database.

15 Negative controls effect log likelihood ratios

Log likelihood ratios (LLR) for the negative controls at various points in time. Closed dots indicate the LLR in that period exceeded the critical value. The critical value depends on sample size within and across periods, and is therefore different for each control.

15.1 Negative controls log likelihood ratios in Optum EHR

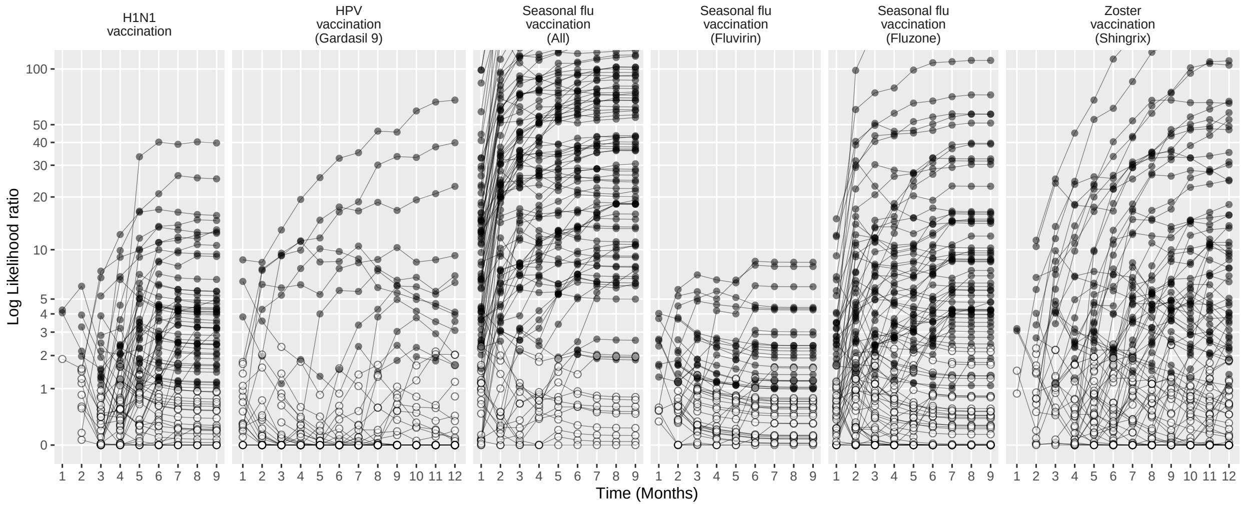

Figure 214: Negative control log likelihood ratios per month using the HistoricalComparator method (Unadjusted, using entire historic period), in the Optum EHR database.

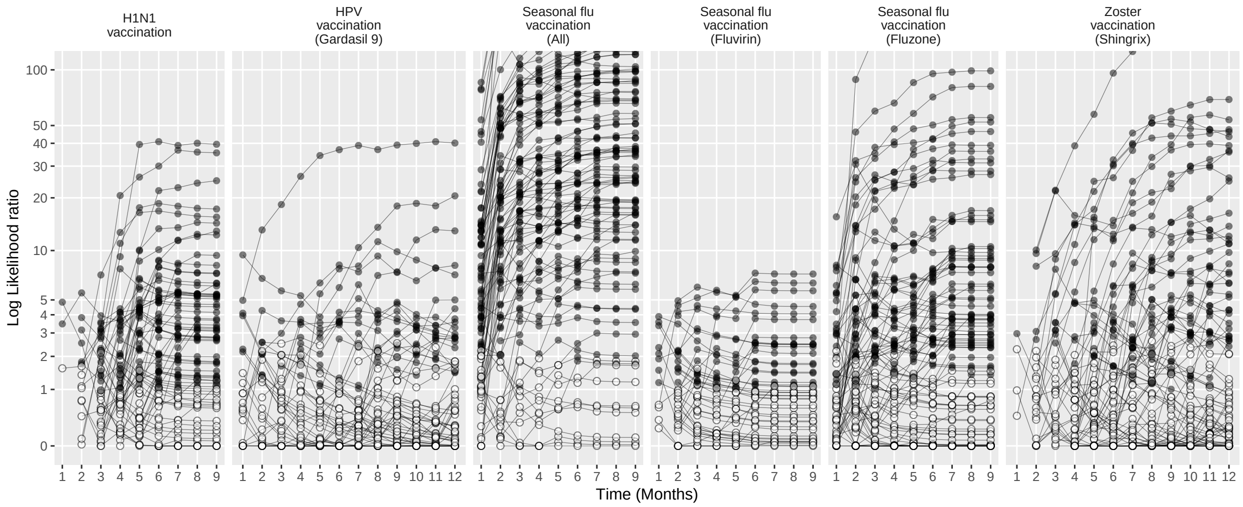

Figure 215: Negative control log likelihood ratios per month using the HistoricalComparator method (Age & sex adjusted, using entire historic period), in the Optum EHR database.

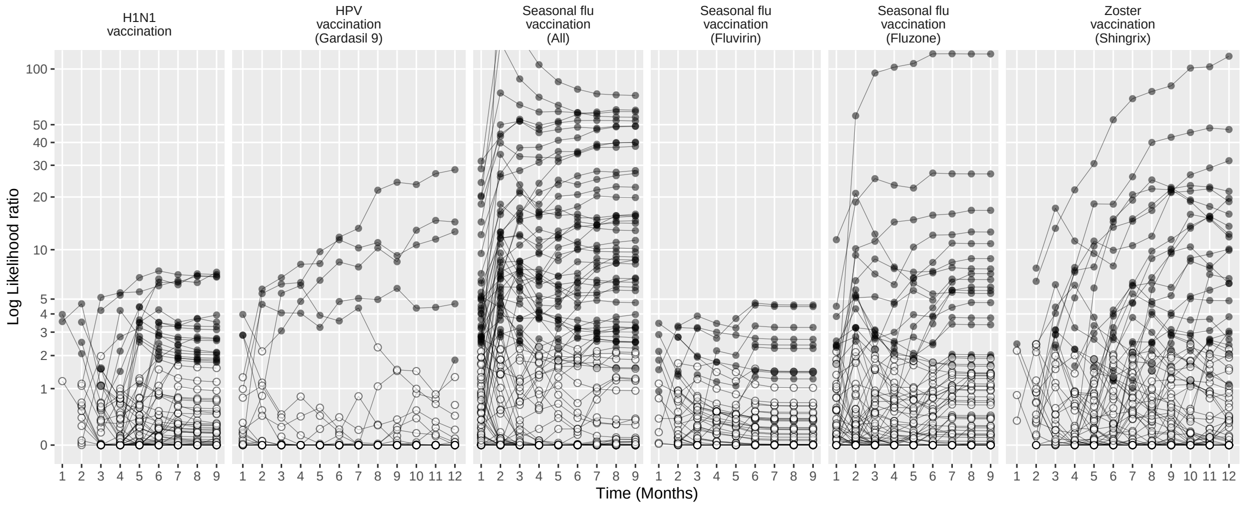

Figure 216: Negative control log likelihood ratios per month using the HistoricalComparator method (Unadjusted, using TaR after historic visit), in the Optum EHR database.

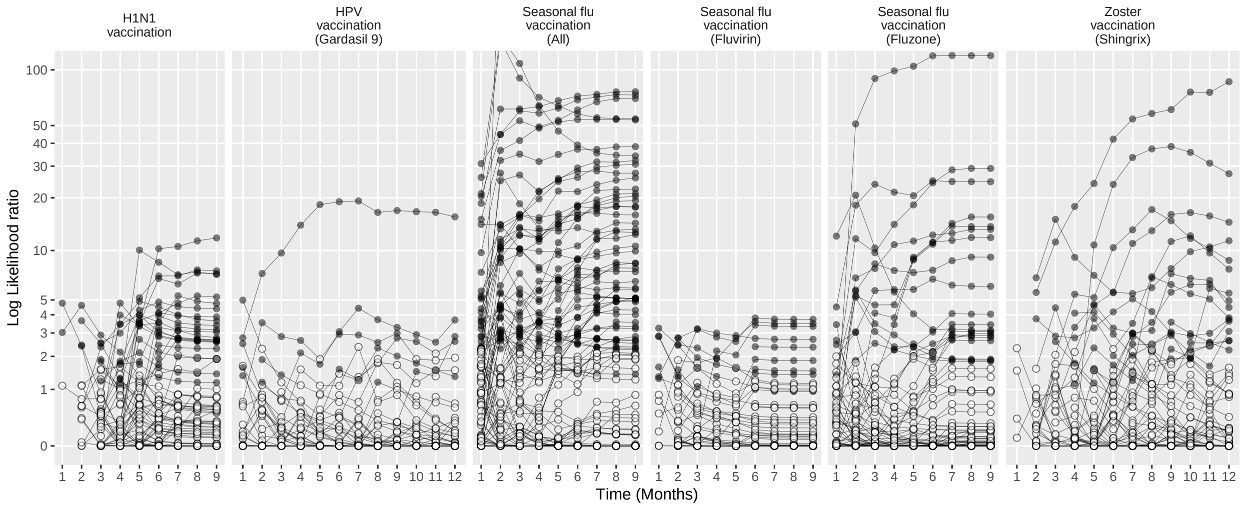

Figure 217: Negative control log likelihood ratios per month using the HistoricalComparator method (Age & sex adjusted, using TaR after historic visit), in the Optum EHR database.

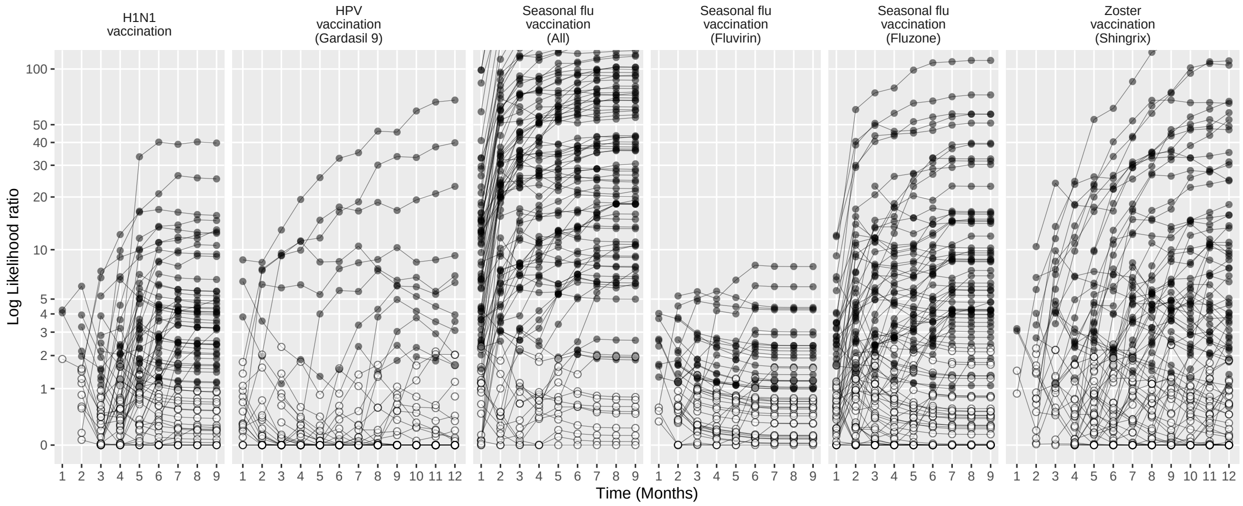

Figure 218: Negative control log likelihood ratios per month using the HistoricalComparator method (Unadjusted, using entire historic period, filtered), in the Optum EHR database.

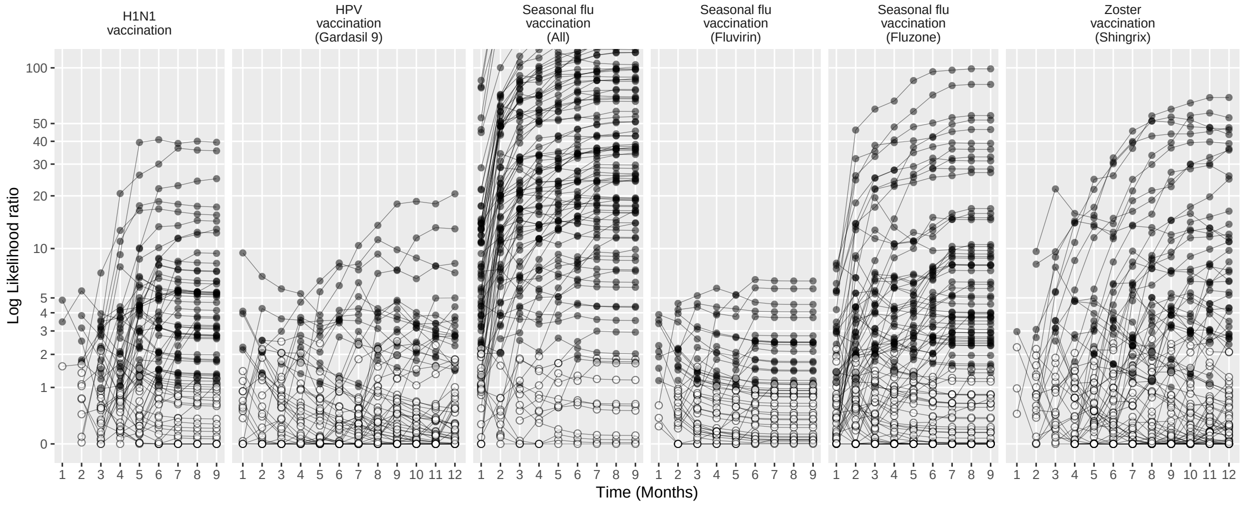

Figure 219: Negative control log likelihood ratios per month using the HistoricalComparator method (Age & sex adjusted, using entire historic period, filtered), in the Optum EHR database.

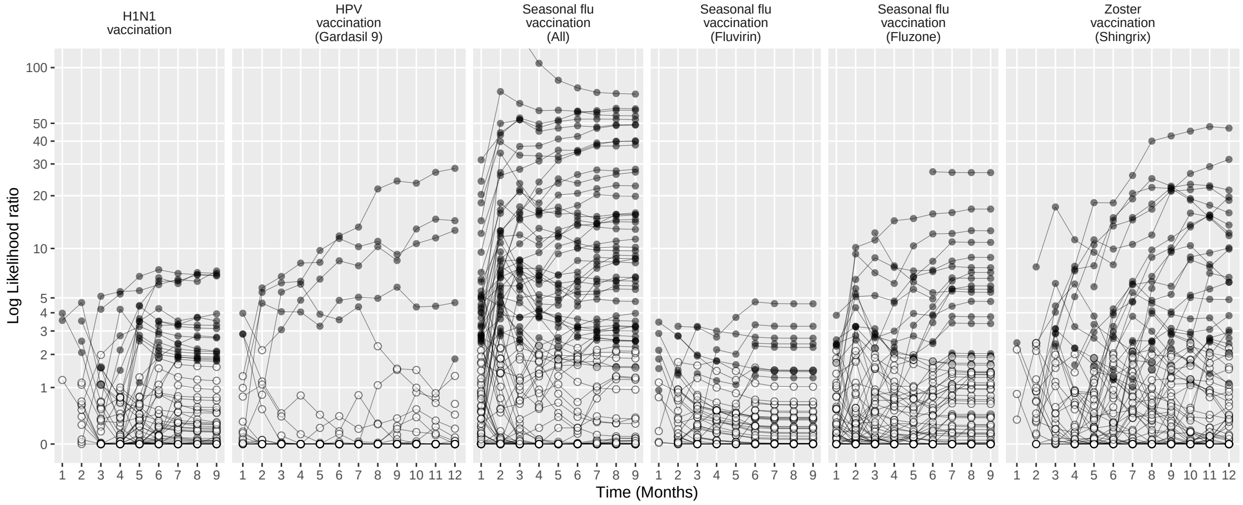

Figure 220: Negative control log likelihood ratios per month using the HistoricalComparator method (Unadjusted, using TaR after historic visit, filtered), in the Optum EHR database.

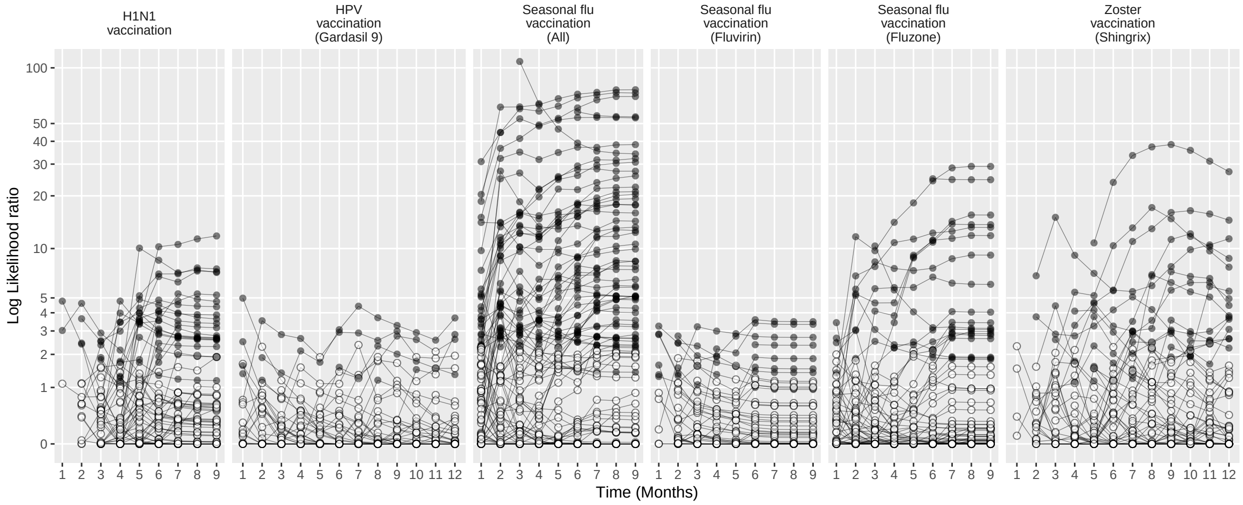

Figure 221: Negative control log likelihood ratios per month using the HistoricalComparator method (Age & sex adjusted, using TaR after historic visit, filtered), in the Optum EHR database.

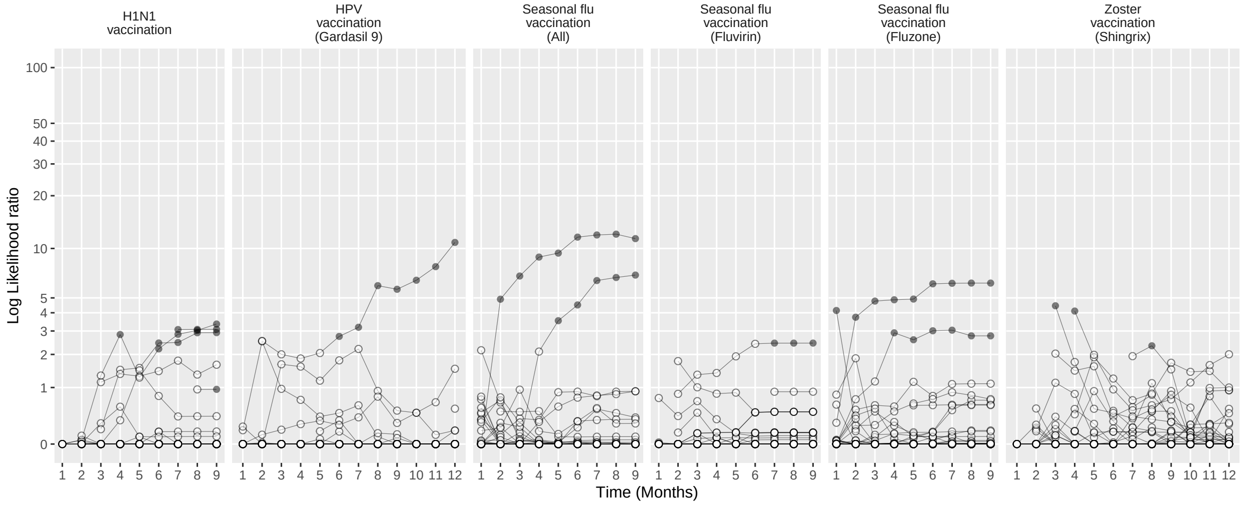

Figure 222: Negative control log likelihood ratios per month using the CohortMethod method (Unadjusted, using outpatient visits as comparator), in the Optum EHR database.

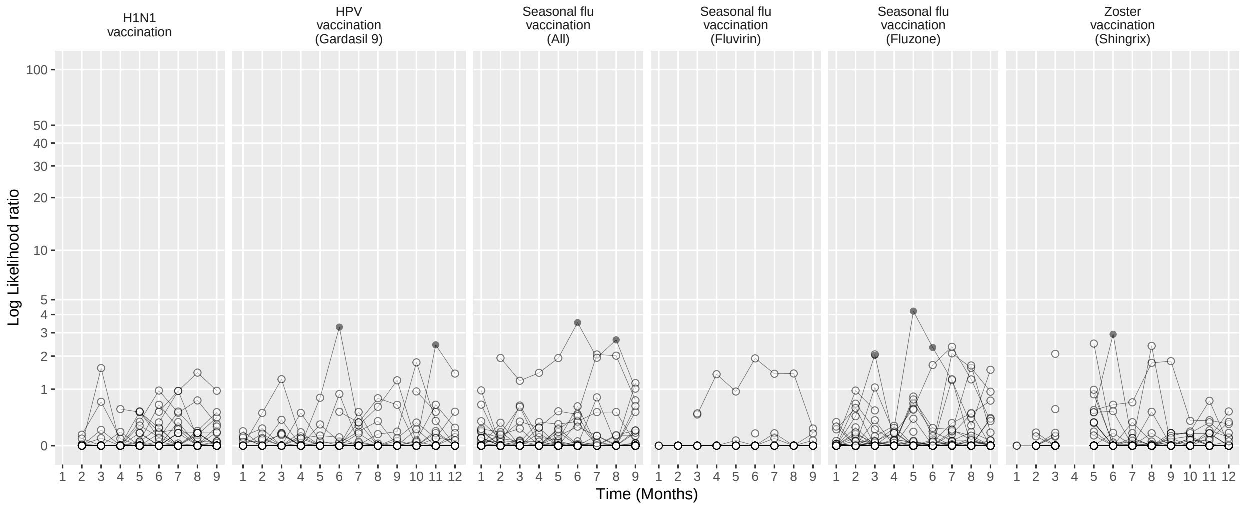

Figure 223: Negative control log likelihood ratios per month using the CohortMethod method (PS matching, using outpatient visits as comparator), in the Optum EHR database.

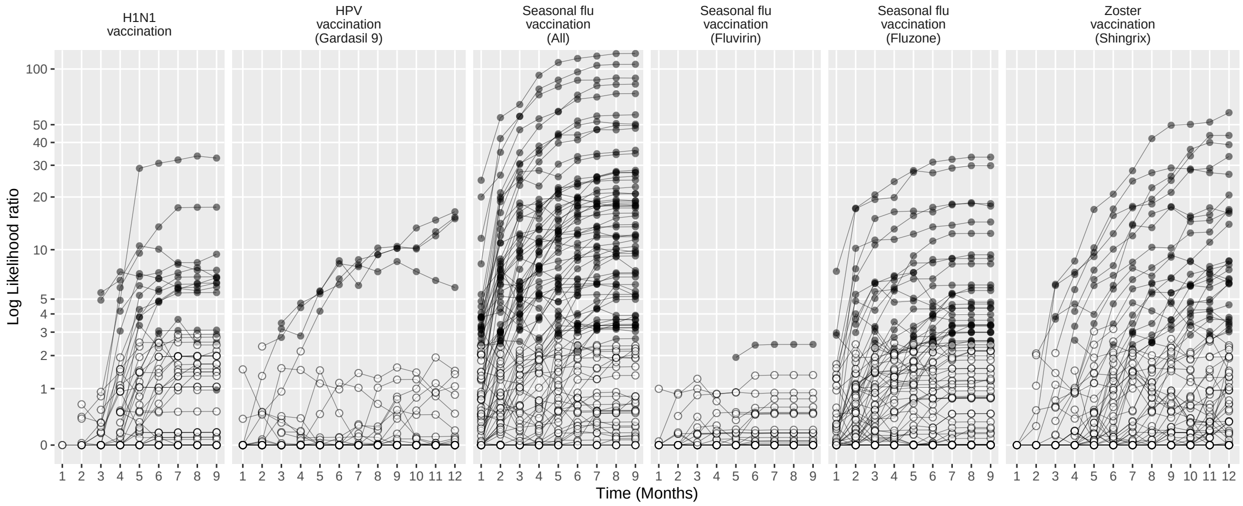

Figure 224: Negative control log likelihood ratios per month using the CohortMethod method (Unadjusted, using random days as comparator), in the Optum EHR database.

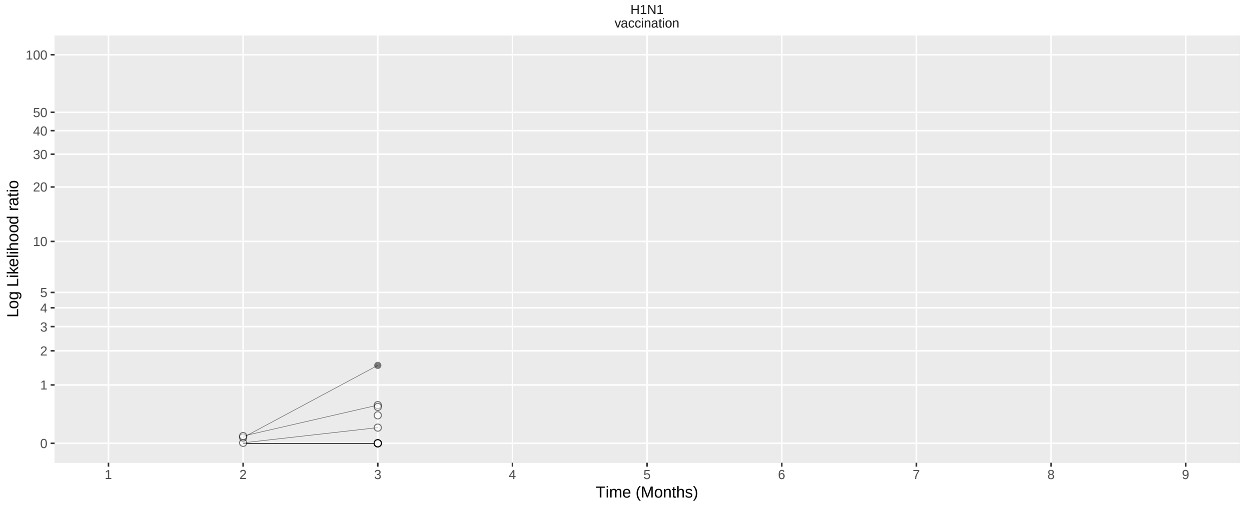

Figure 225: Negative control log likelihood ratios per month using the CohortMethod method (PS matching, using random days as comparator), in the Optum EHR database.

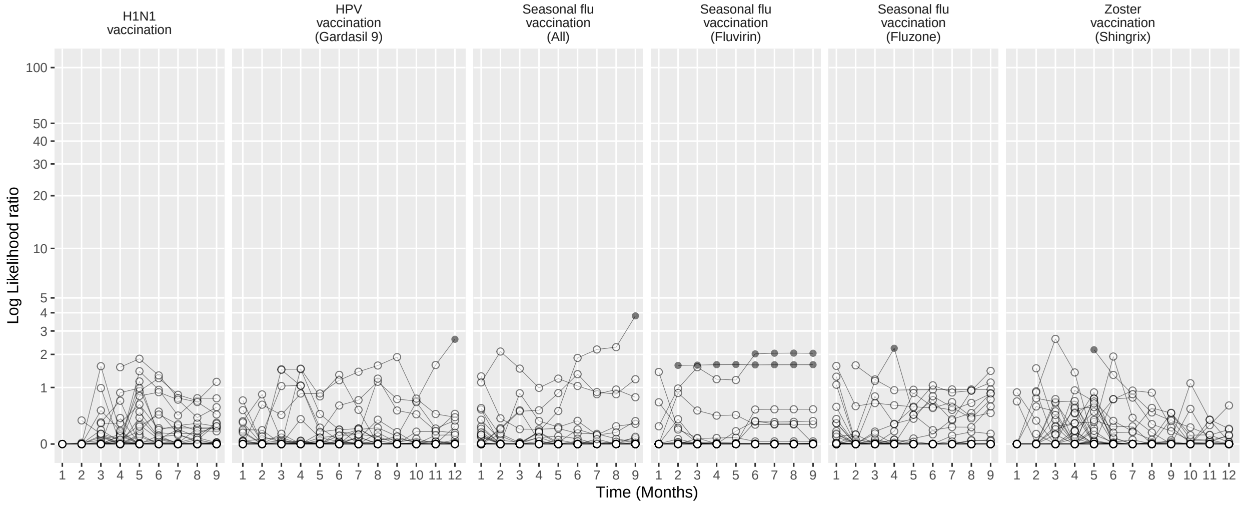

Figure 226: Negative control log likelihood ratios per month using the CohortMethod method (PS stratification, using outpatient visits as comparator), in the Optum EHR database.

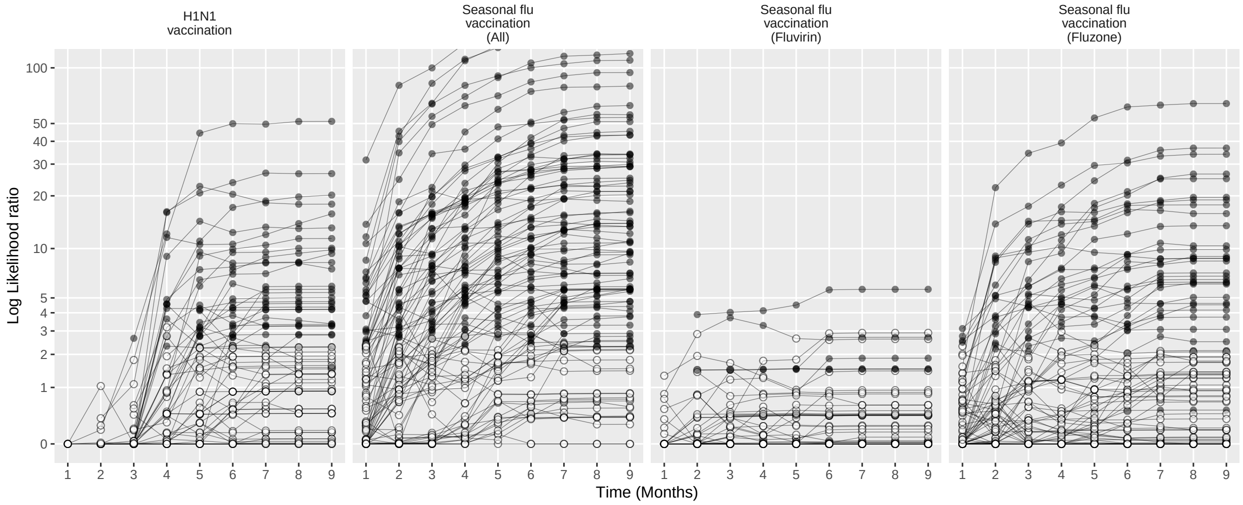

Figure 227: Negative control log likelihood ratios per month using the CohortMethod method (PS stratification, using random days as comparator), in the Optum EHR database.

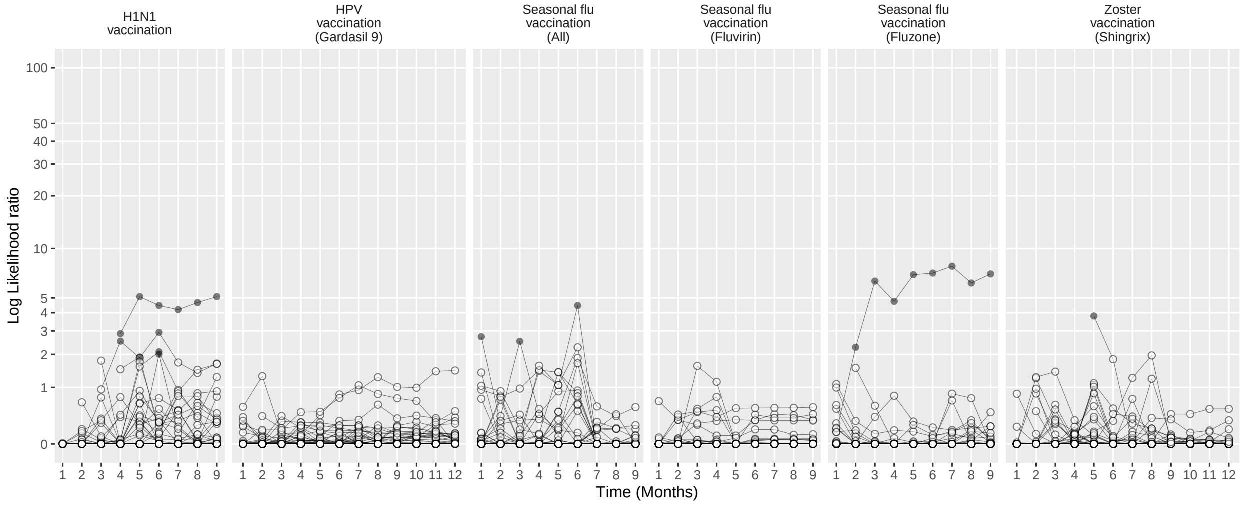

Figure 228: Negative control log likelihood ratios per month using the CohortMethod method (PS weighting, using outpatient visits as comparator), in the Optum EHR database.

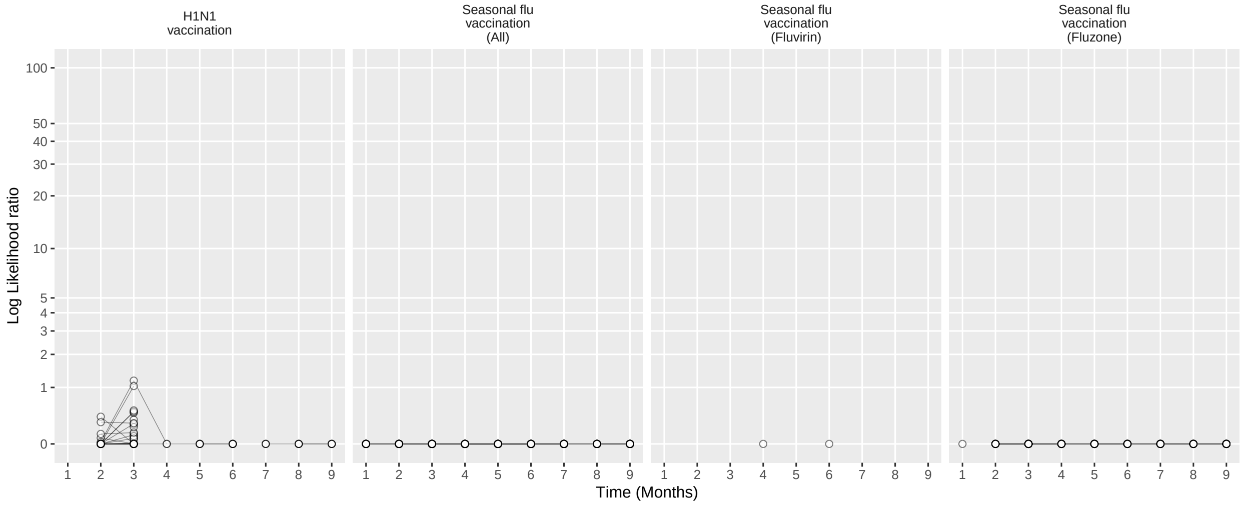

Figure 229: Negative control log likelihood ratios per month using the CohortMethod method (PS weighting, using random days as comparator), in the Optum EHR database.

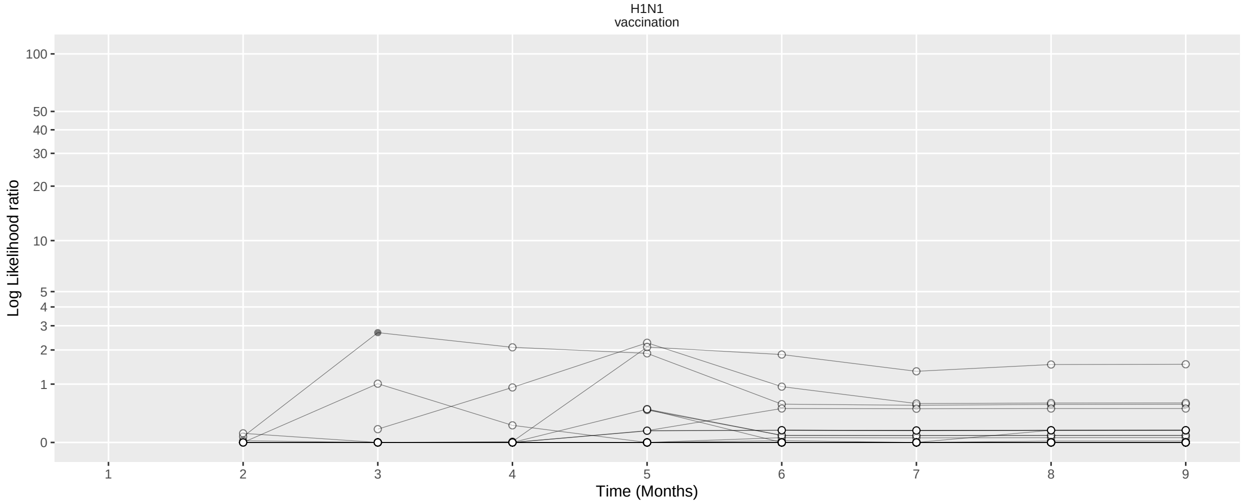

Figure 230: Negative control log likelihood ratios per month using the CohortMethod method (Per-month PS matching, using outpatient visits as comparator), in the Optum EHR database.

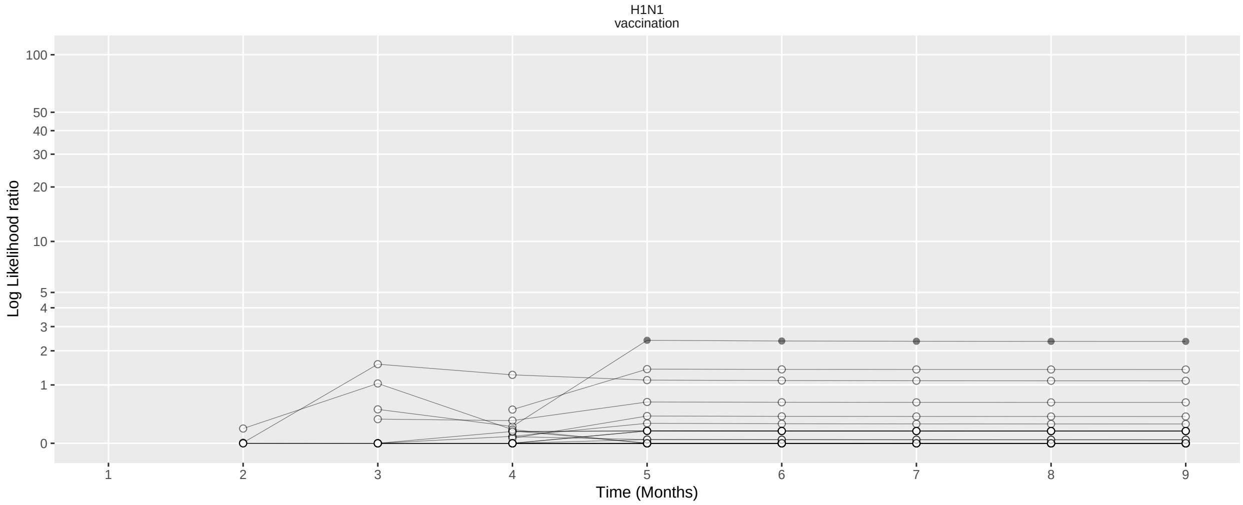

Figure 231: Negative control log likelihood ratios per month using the CohortMethod method (Per-month PS matching, using random days as comparator), in the Optum EHR database.

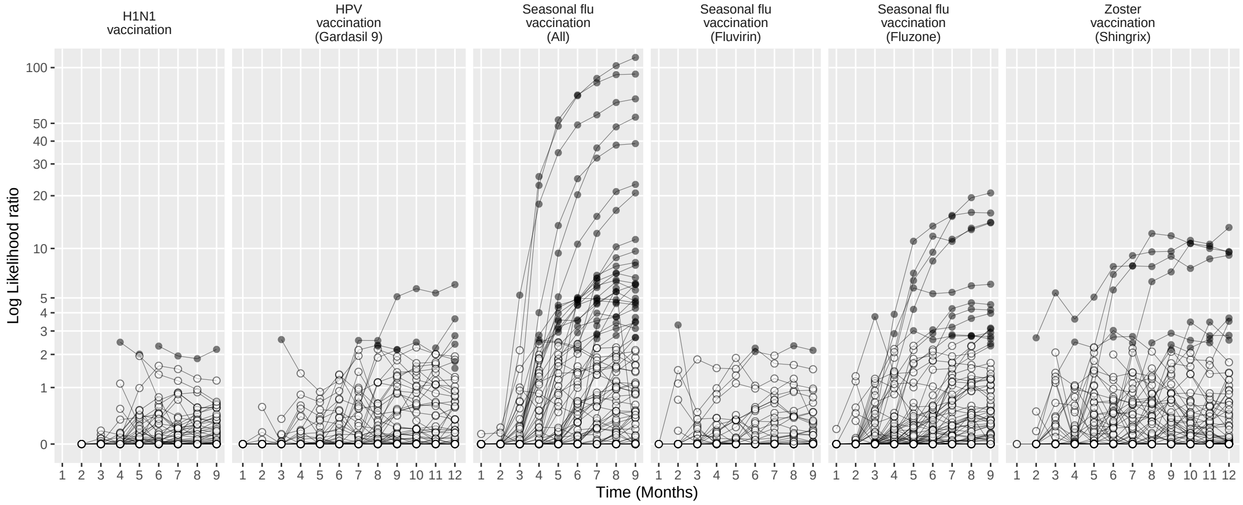

Figure 232: Negative control log likelihood ratios per month using the SCCS method (Unadjusted SCCS excluding pre-vaccination window), in the Optum EHR database.

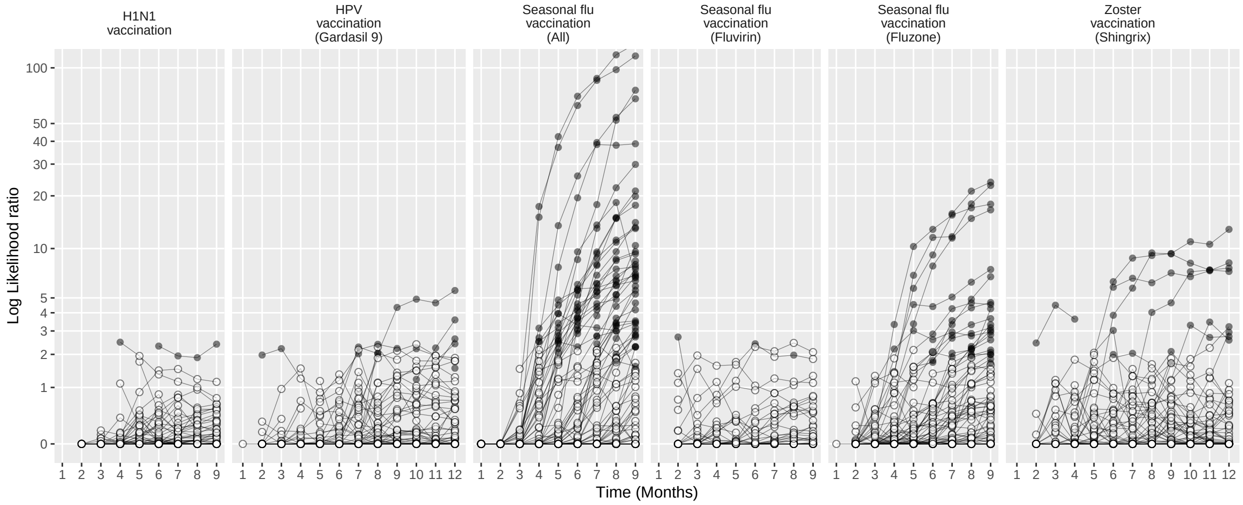

Figure 233: Negative control log likelihood ratios per month using the SCCS method (Age & season adjusted SCCS excluding pre-vaccination window), in the Optum EHR database.

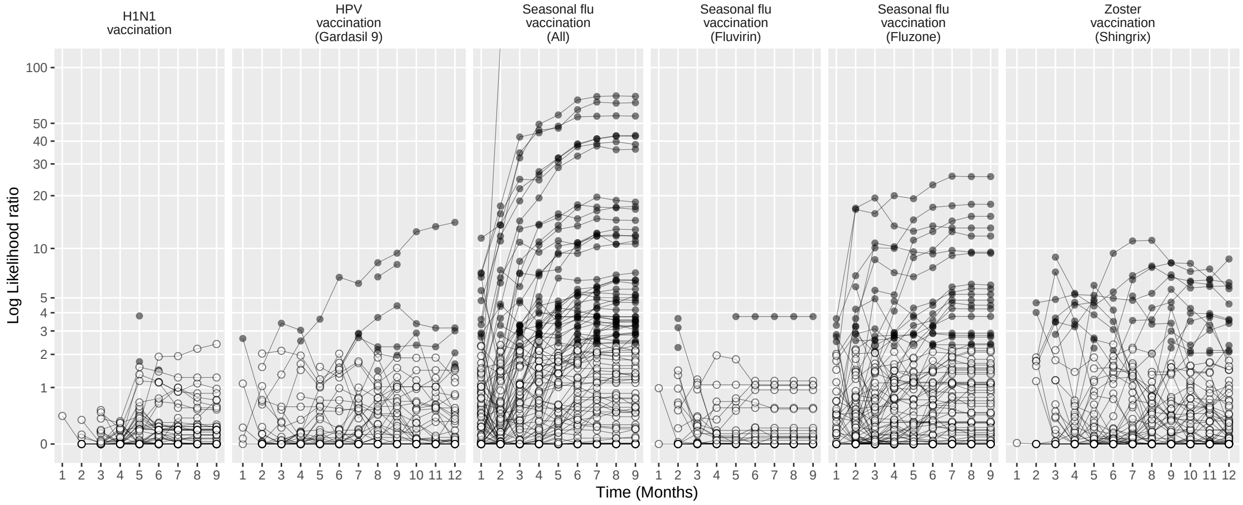

Figure 234: Negative control log likelihood ratios per month using the SCCS method (SCRI with prior control interval), in the Optum EHR database.

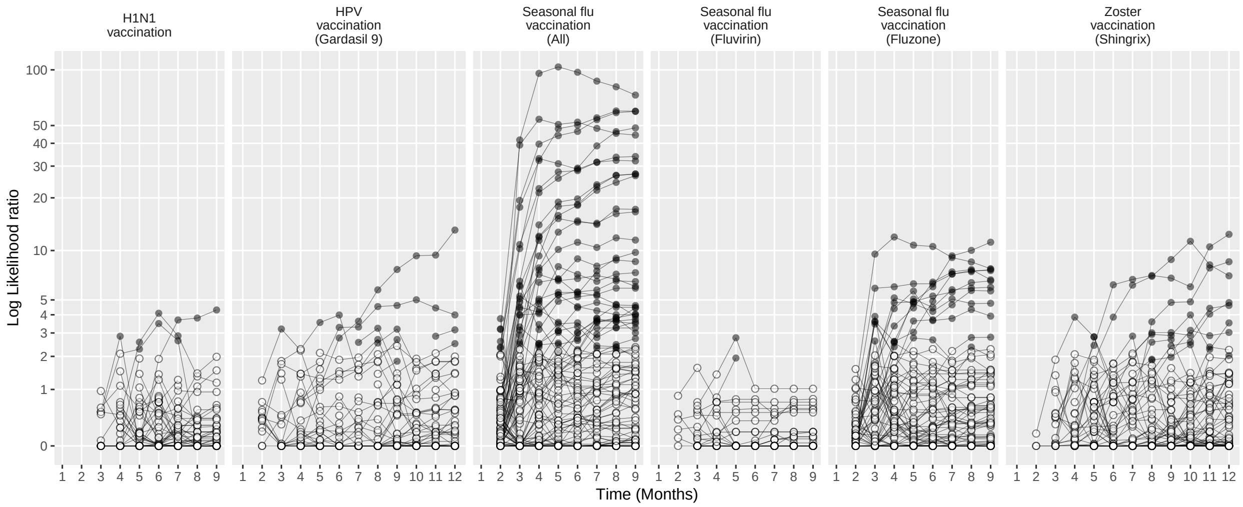

Figure 235: Negative control log likelihood ratios per month using the SCCS method (SCRI with posterior control interval), in the Optum EHR database.

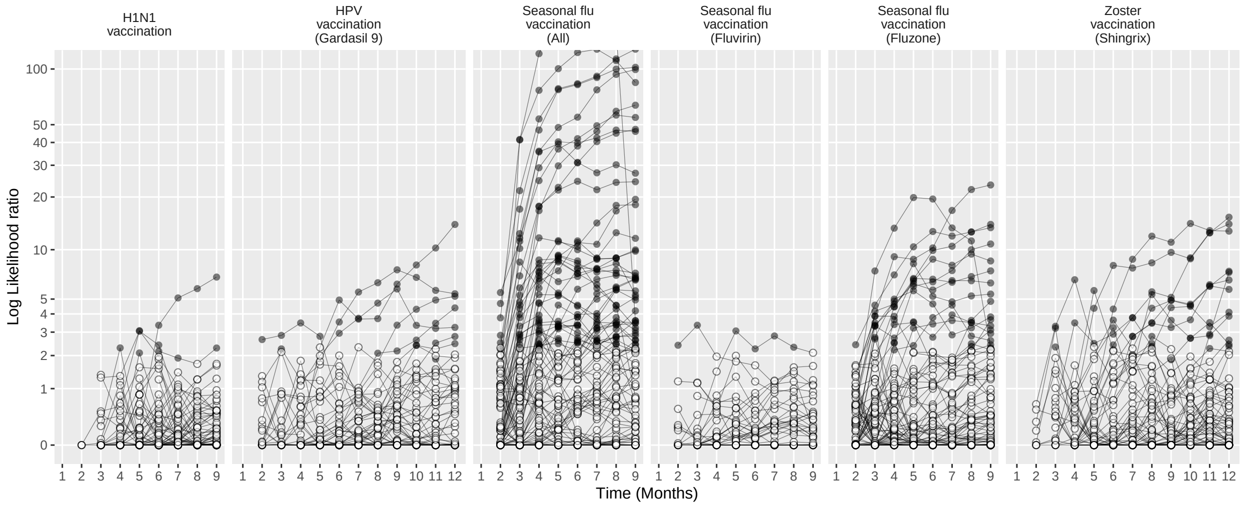

Figure 236: Negative control log likelihood ratios per month using the SCCS method (Unadjusted SCCS excluding all pre-vaccination time), in the Optum EHR database.

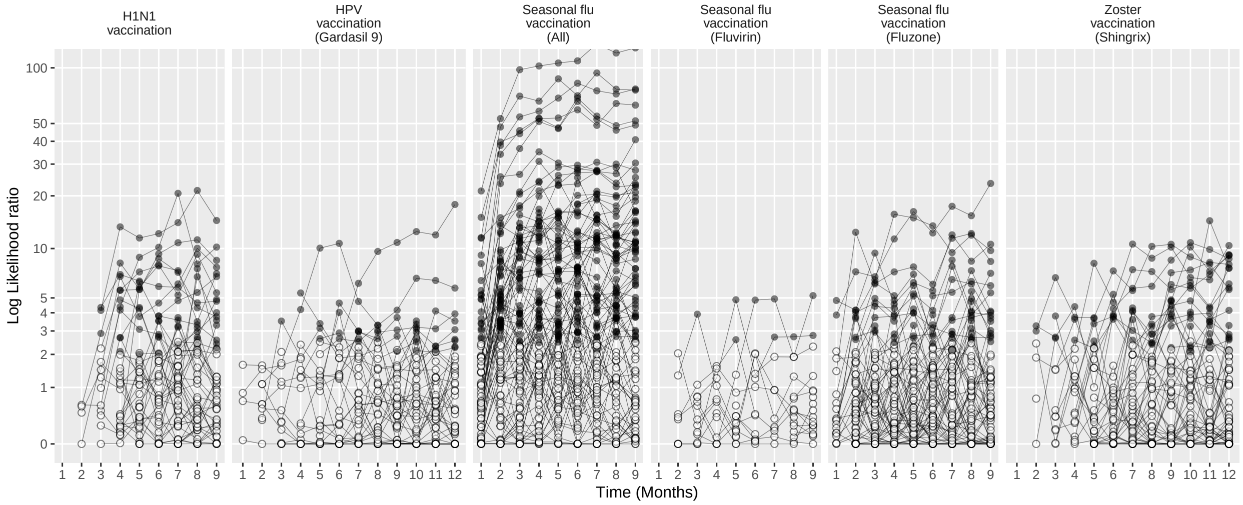

Figure 237: Negative control log likelihood ratios per month using the CaseControl method (Age & sex adjusted, using random controls), in the Optum EHR database.

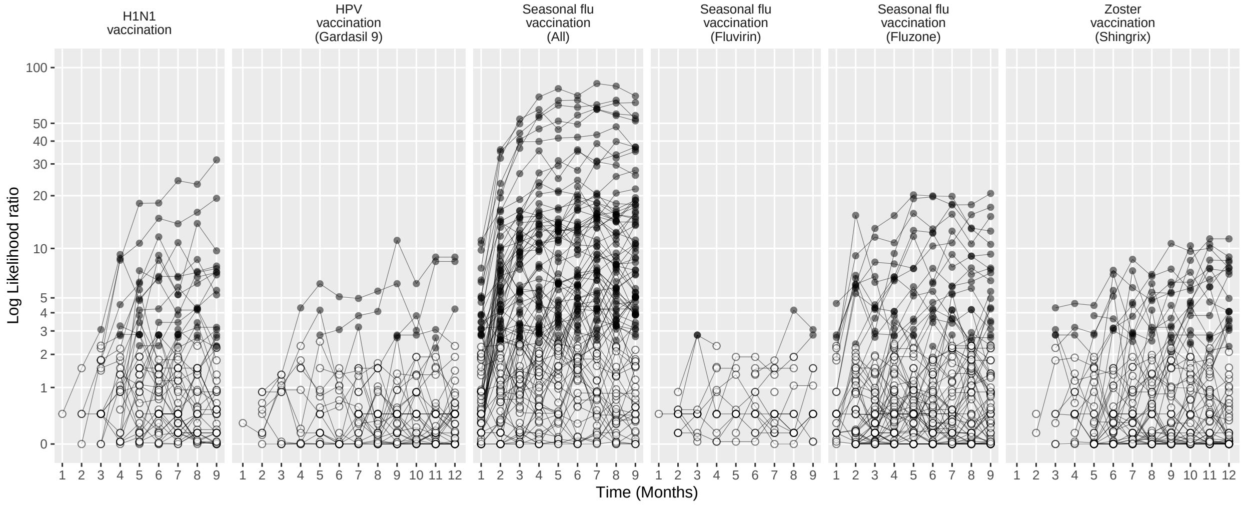

Figure 238: Negative control log likelihood ratios per month using the CaseControl method (Age & sex matched controls), in the Optum EHR database.

15.2 Negative controls log likelihood ratios in MDCD

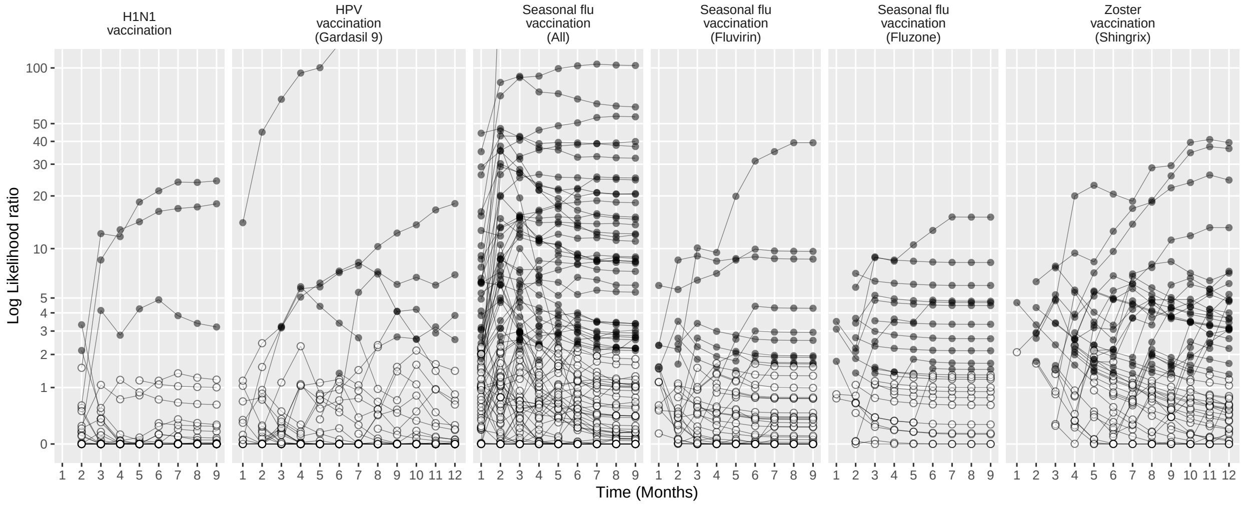

Figure 239: Negative control log likelihood ratios per month using the HistoricalComparator method (Unadjusted, using entire historic period), in the MDCD database.

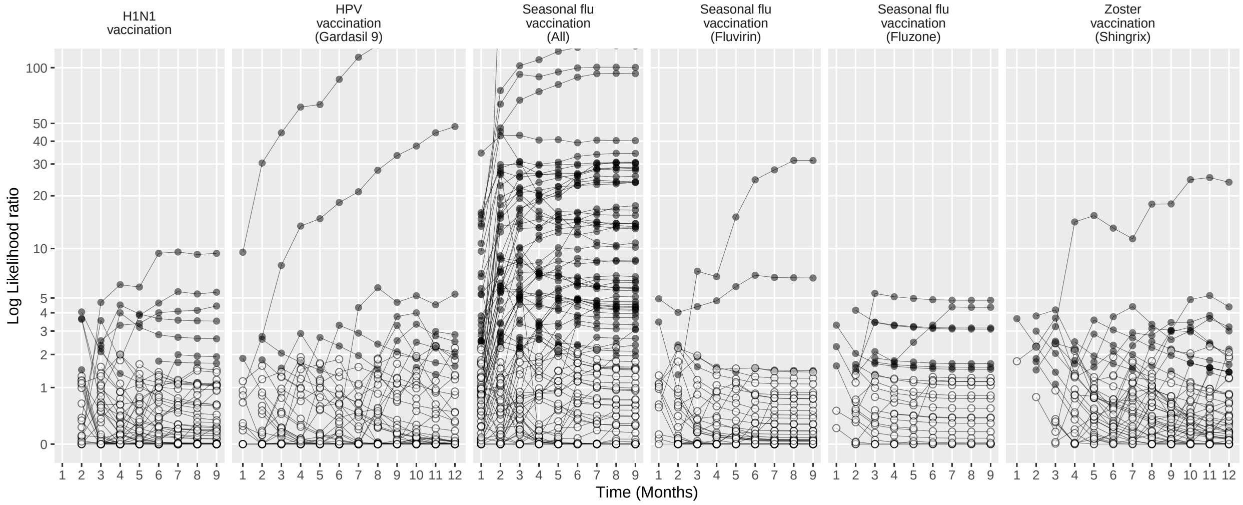

Figure 240: Negative control log likelihood ratios per month using the HistoricalComparator method (Age & sex adjusted, using entire historic period), in the MDCD database.

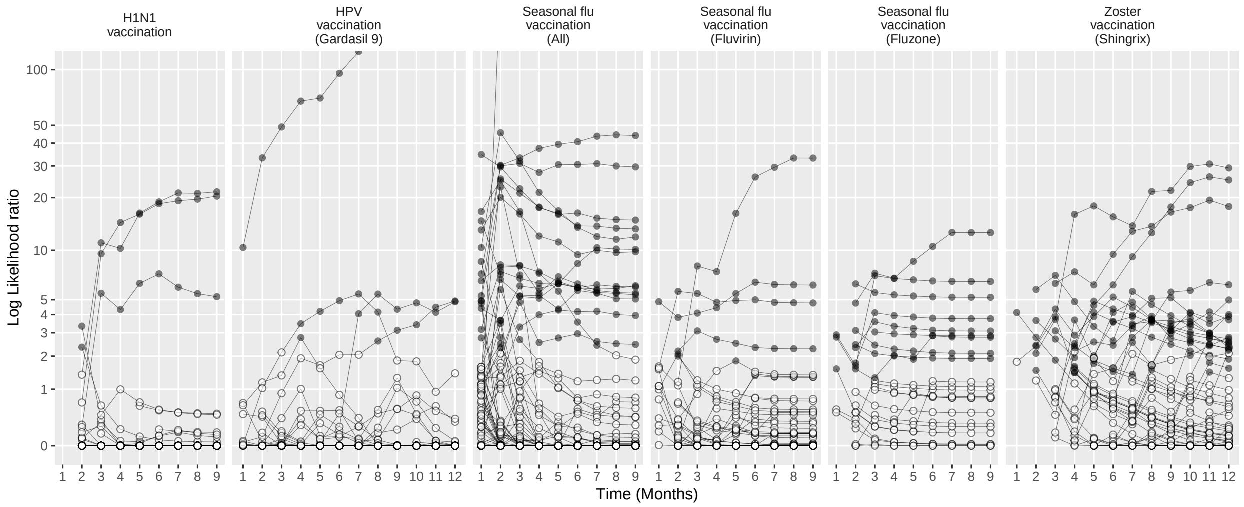

Figure 241: Negative control log likelihood ratios per month using the HistoricalComparator method (Unadjusted, using TaR after historic visit), in the MDCD database.

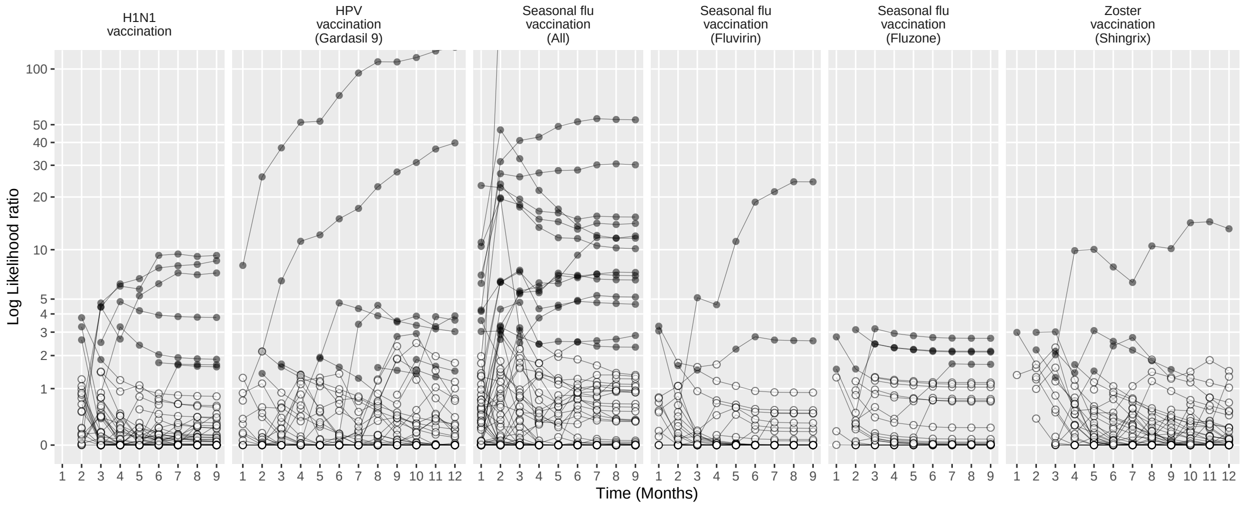

Figure 242: Negative control log likelihood ratios per month using the HistoricalComparator method (Age & sex adjusted, using TaR after historic visit), in the MDCD database.

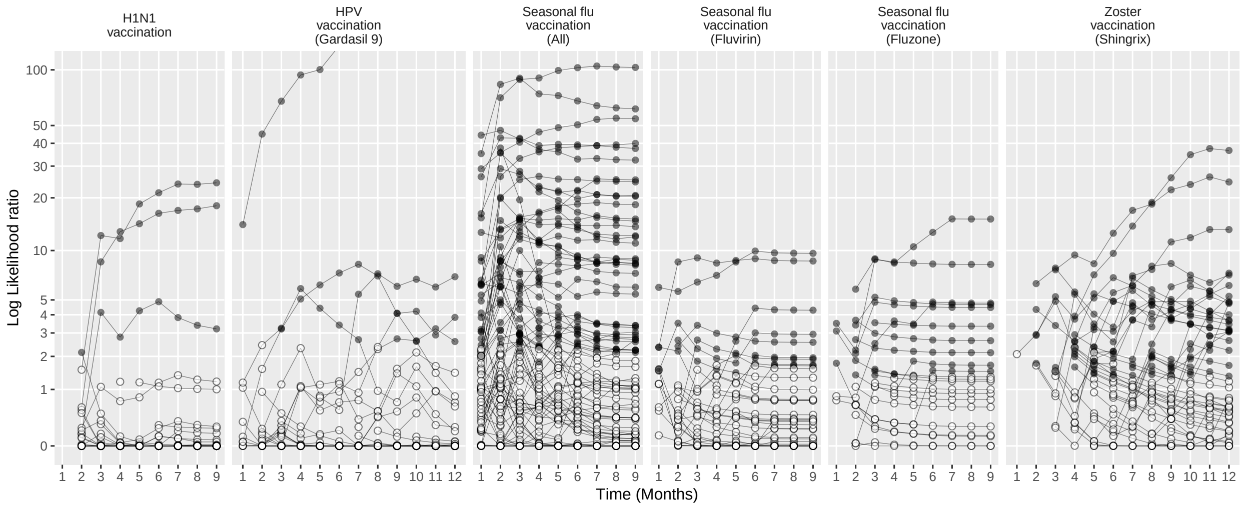

Figure 243: Negative control log likelihood ratios per month using the HistoricalComparator method (Unadjusted, using entire historic period, filtered), in the MDCD database.

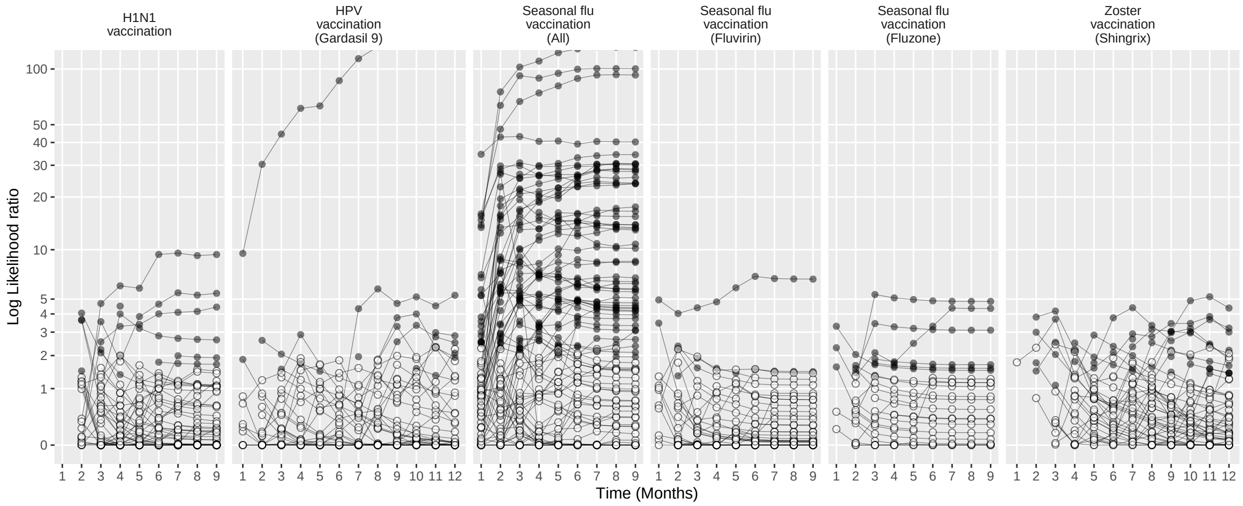

Figure 244: Negative control log likelihood ratios per month using the HistoricalComparator method (Age & sex adjusted, using entire historic period, filtered), in the MDCD database.

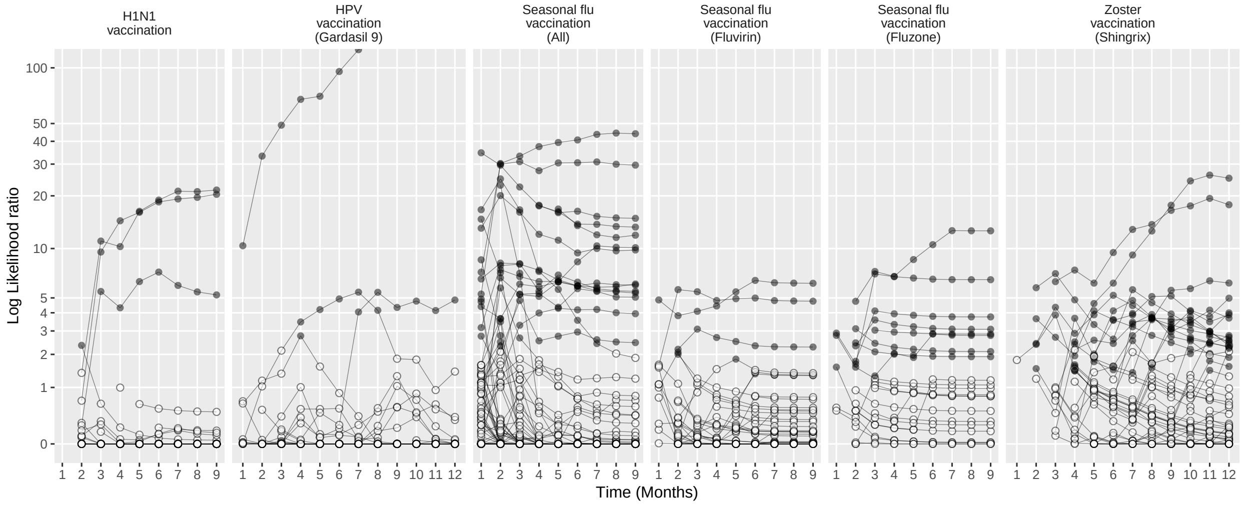

Figure 245: Negative control log likelihood ratios per month using the HistoricalComparator method (Unadjusted, using TaR after historic visit, filtered), in the MDCD database.

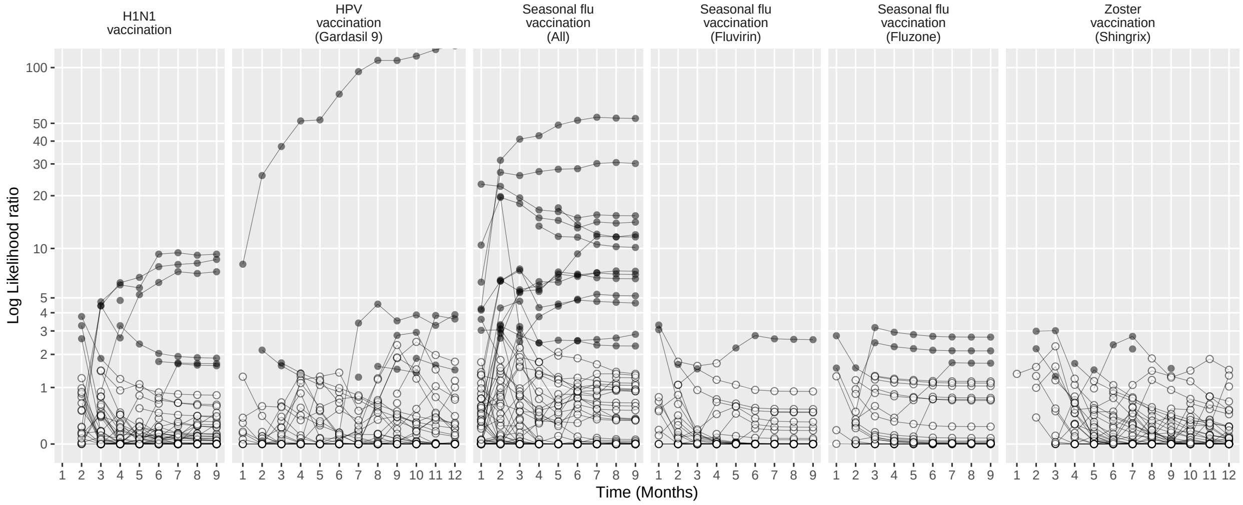

Figure 246: Negative control log likelihood ratios per month using the HistoricalComparator method (Age & sex adjusted, using TaR after historic visit, filtered), in the MDCD database.

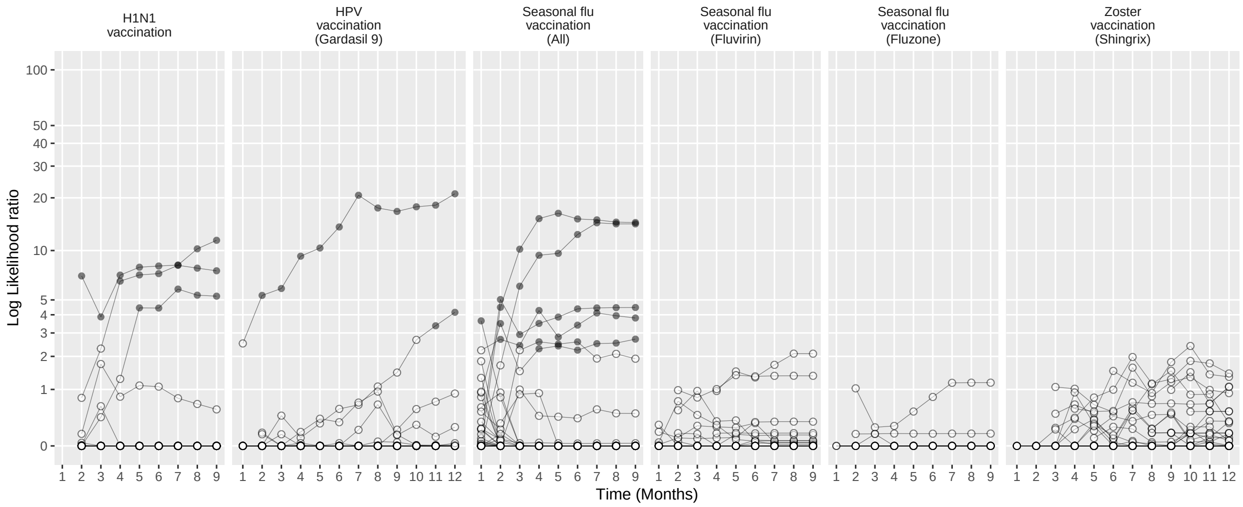

Figure 247: Negative control log likelihood ratios per month using the CohortMethod method (Unadjusted, using outpatient visits as comparator), in the MDCD database.

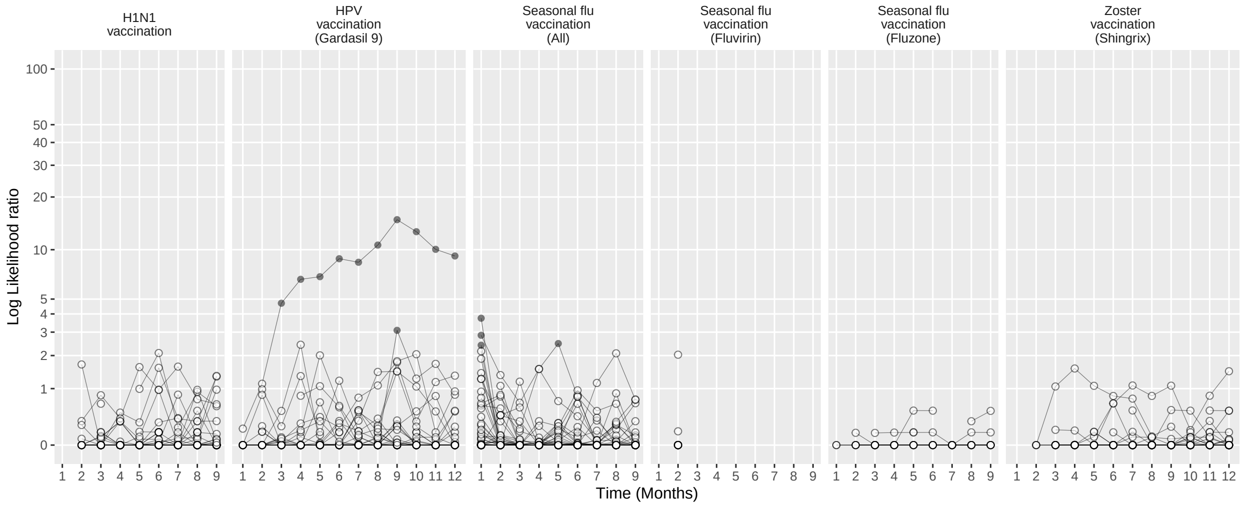

Figure 248: Negative control log likelihood ratios per month using the CohortMethod method (PS matching, using outpatient visits as comparator), in the MDCD database.

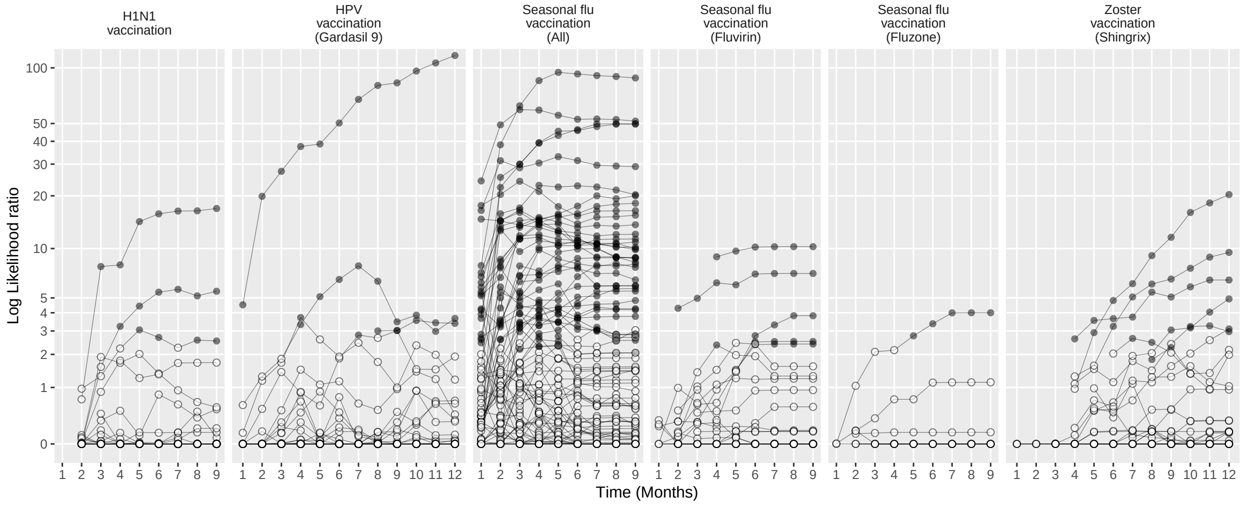

Figure 249: Negative control log likelihood ratios per month using the CohortMethod method (Unadjusted, using random days as comparator), in the MDCD database.

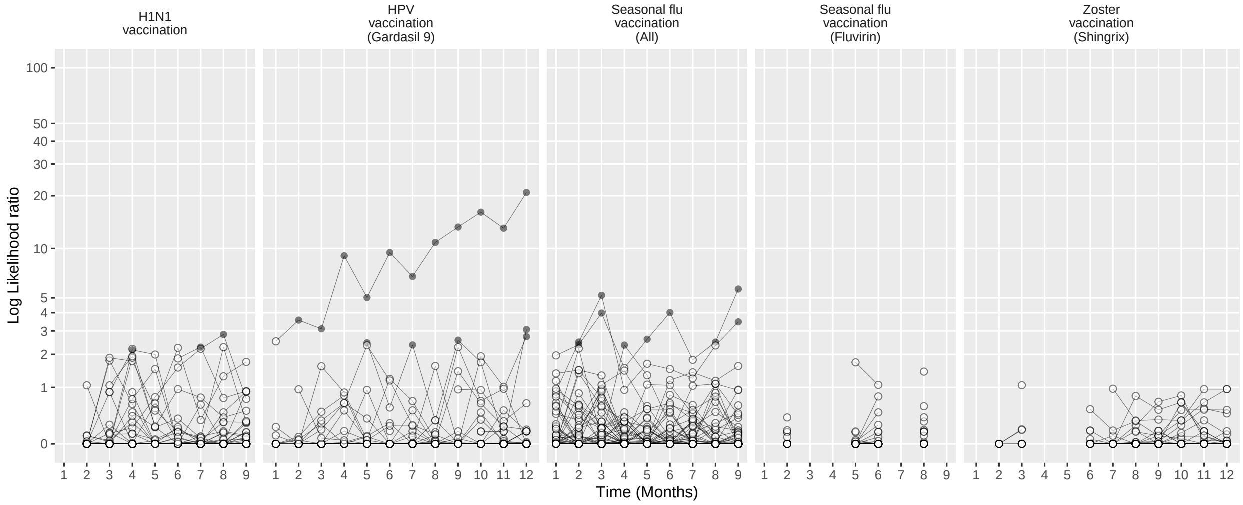

Figure 250: Negative control log likelihood ratios per month using the CohortMethod method (PS matching, using random days as comparator), in the MDCD database.

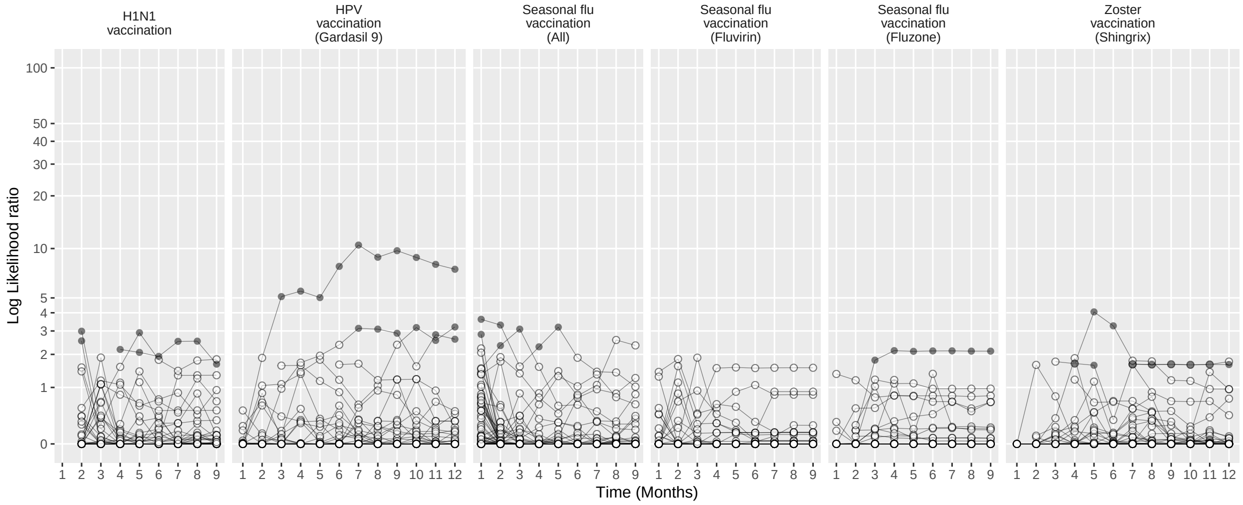

Figure 251: Negative control log likelihood ratios per month using the CohortMethod method (PS stratification, using outpatient visits as comparator), in the MDCD database.

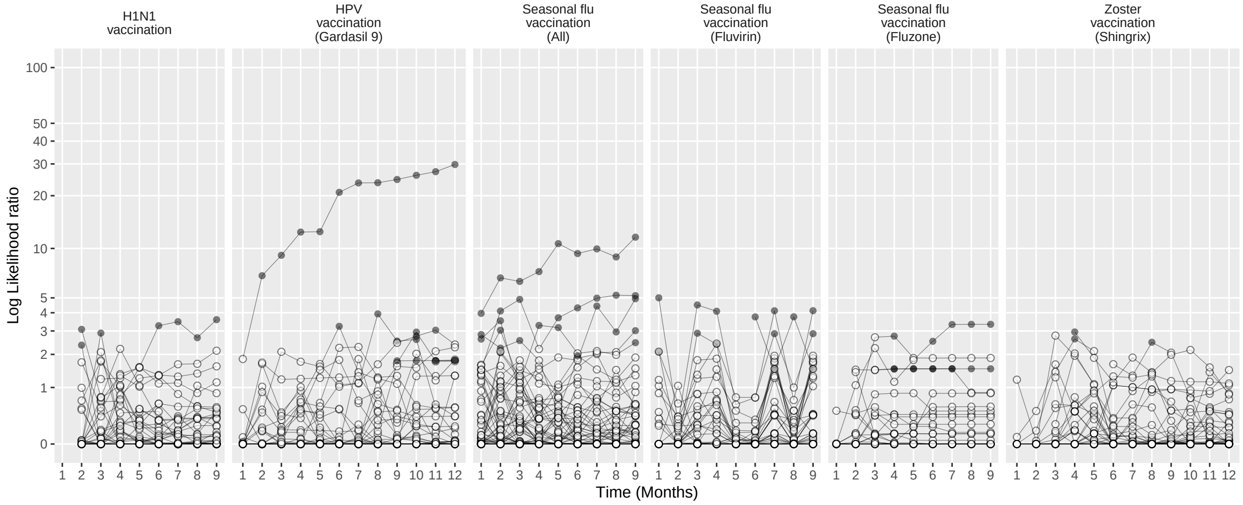

Figure 252: Negative control log likelihood ratios per month using the CohortMethod method (PS stratification, using random days as comparator), in the MDCD database.

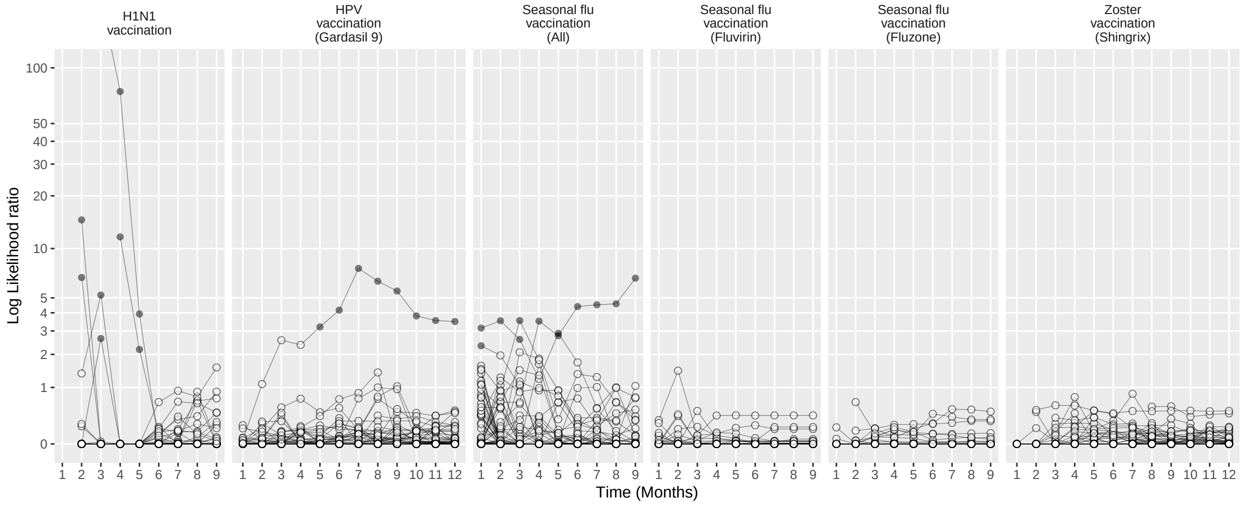

Figure 253: Negative control log likelihood ratios per month using the CohortMethod method (PS weighting, using outpatient visits as comparator), in the MDCD database.

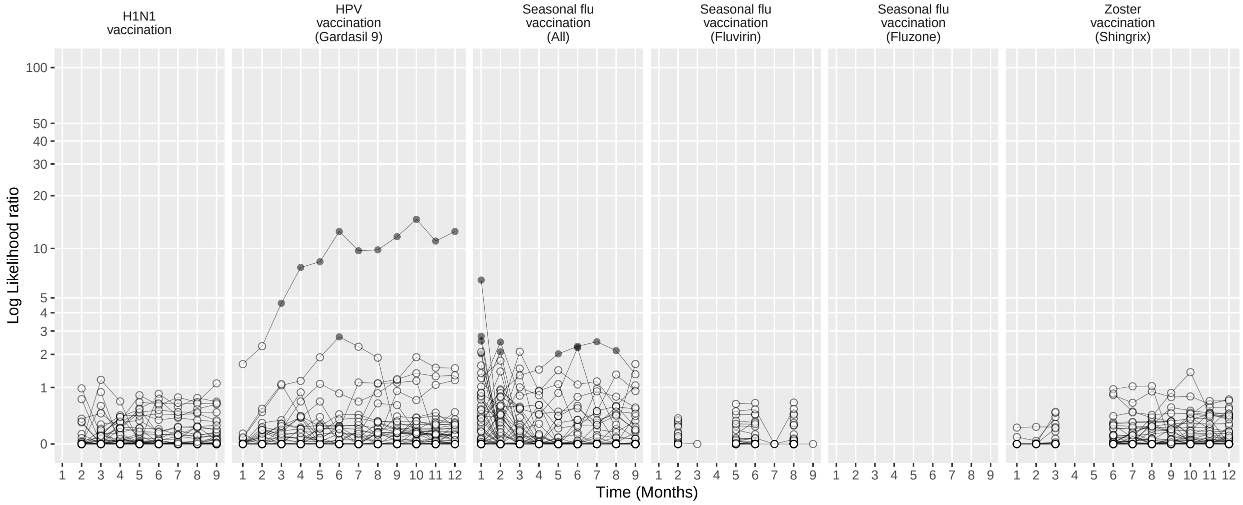

Figure 254: Negative control log likelihood ratios per month using the CohortMethod method (PS weighting, using random days as comparator), in the MDCD database.

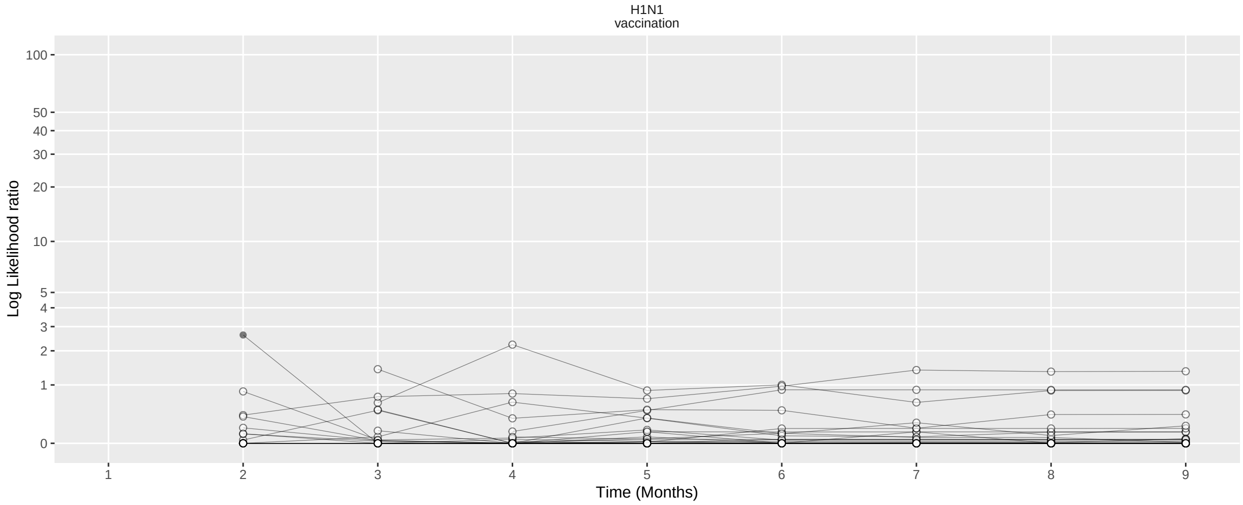

Figure 255: Negative control log likelihood ratios per month using the CohortMethod method (Per-month PS matching, using outpatient visits as comparator), in the MDCD database.

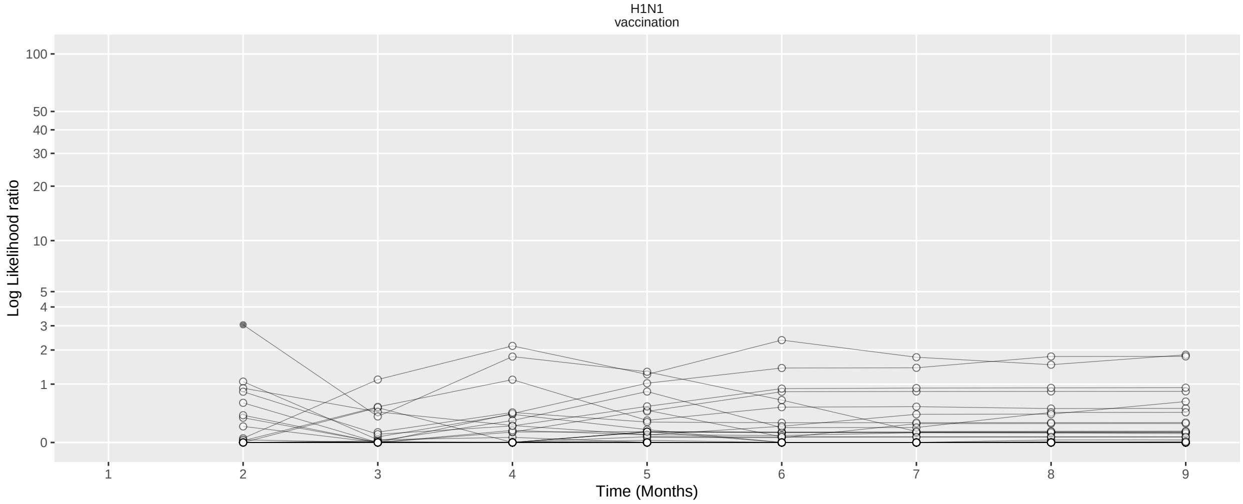

Figure 256: Negative control log likelihood ratios per month using the CohortMethod method (Per-month PS matching, using random days as comparator), in the MDCD database.

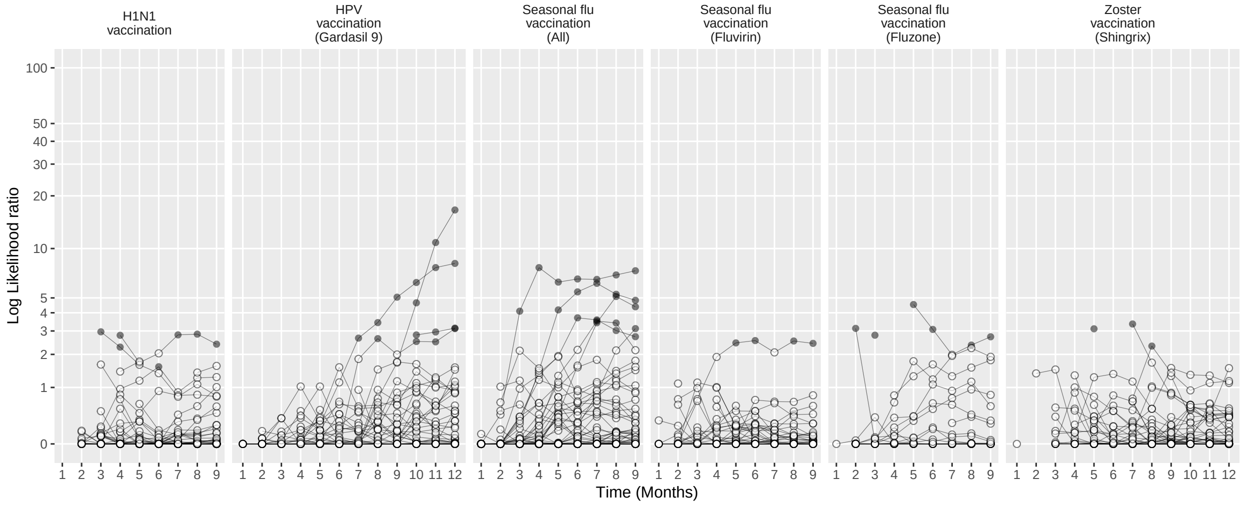

Figure 257: Negative control log likelihood ratios per month using the SCCS method (Unadjusted SCCS excluding pre-vaccination window), in the MDCD database.

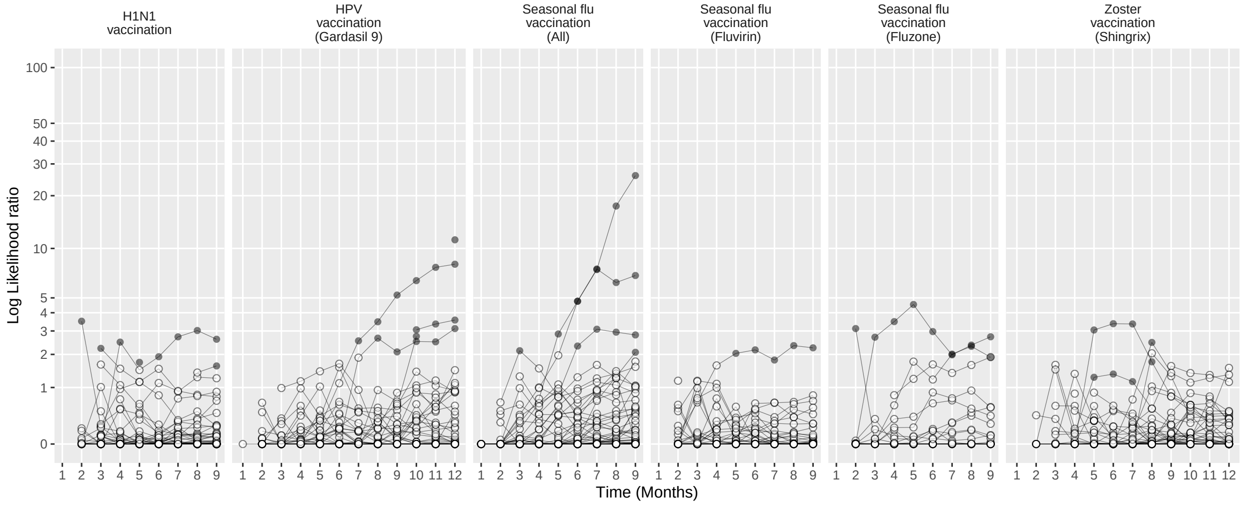

Figure 258: Negative control log likelihood ratios per month using the SCCS method (Age & season adjusted SCCS excluding pre-vaccination window), in the MDCD database.

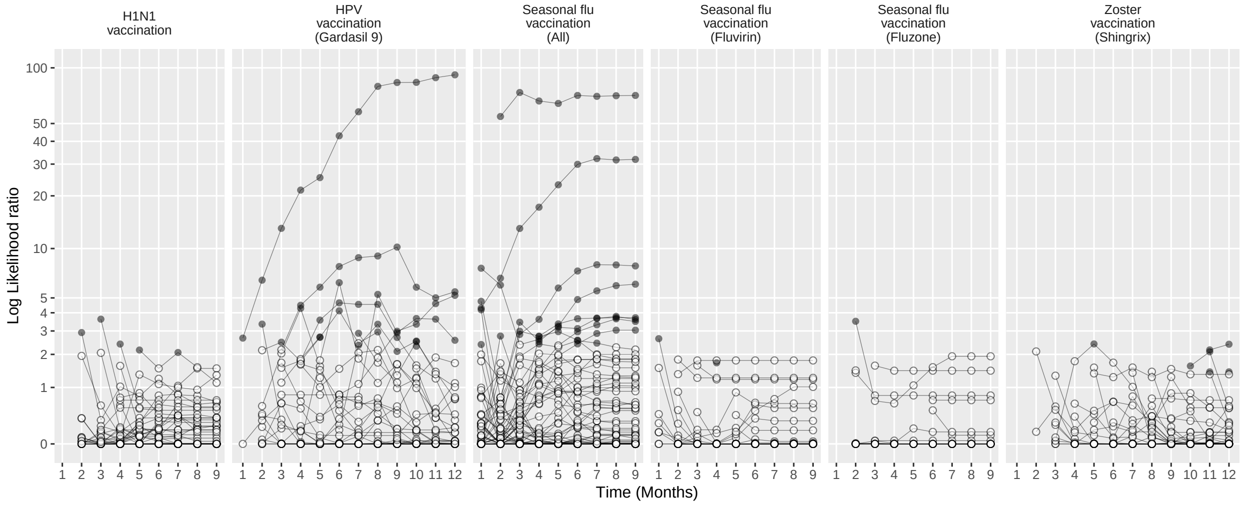

Figure 259: Negative control log likelihood ratios per month using the SCCS method (SCRI with prior control interval), in the MDCD database.

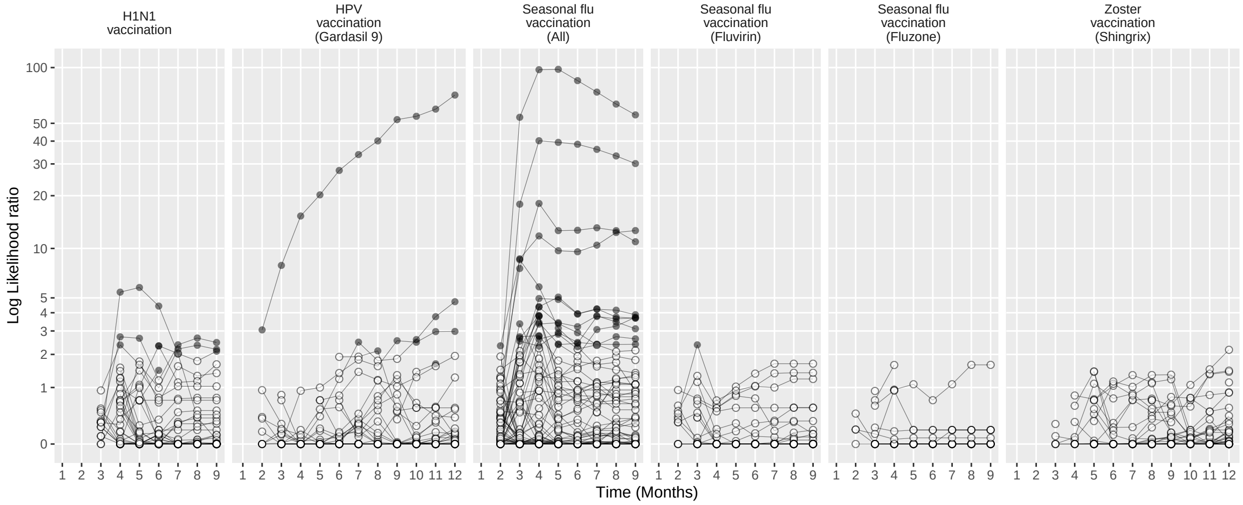

Figure 260: Negative control log likelihood ratios per month using the SCCS method (SCRI with posterior control interval), in the MDCD database.

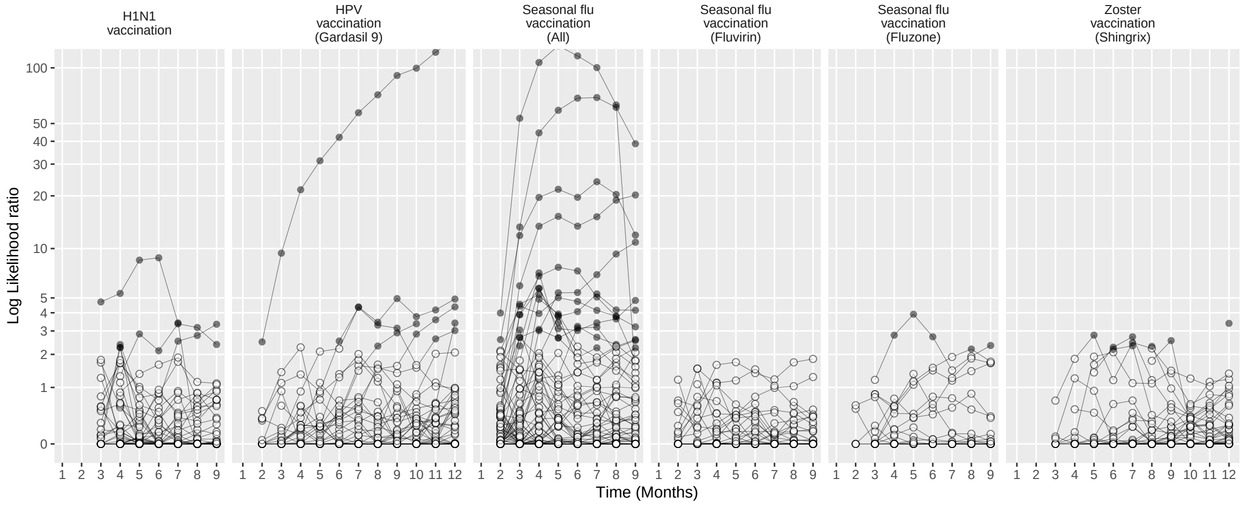

Figure 261: Negative control log likelihood ratios per month using the SCCS method (Unadjusted SCCS excluding all pre-vaccination time), in the MDCD database.

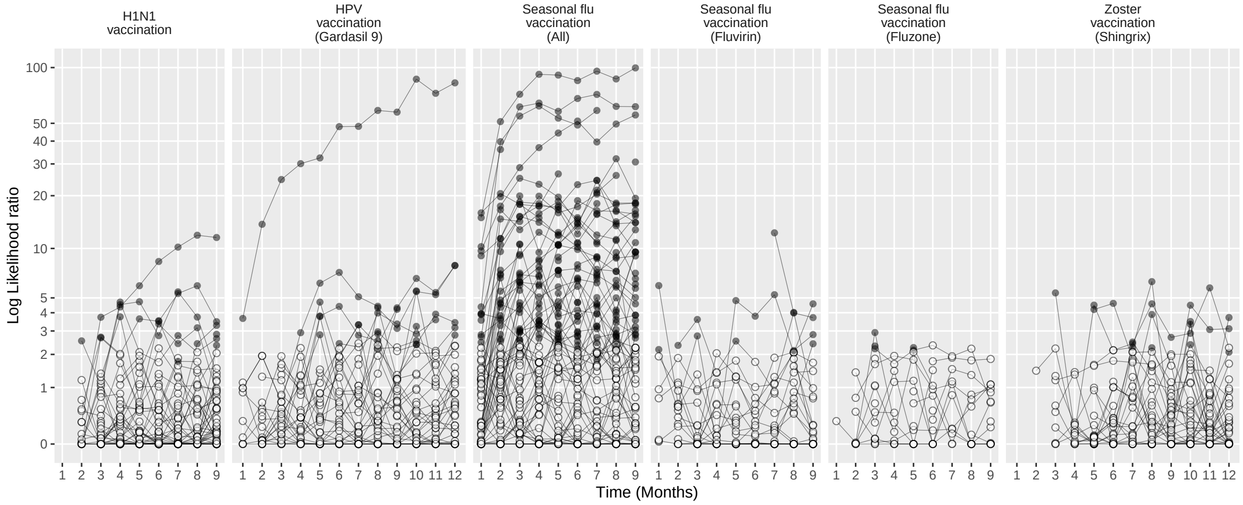

Figure 262: Negative control log likelihood ratios per month using the CaseControl method (Age & sex adjusted, using random controls), in the MDCD database.

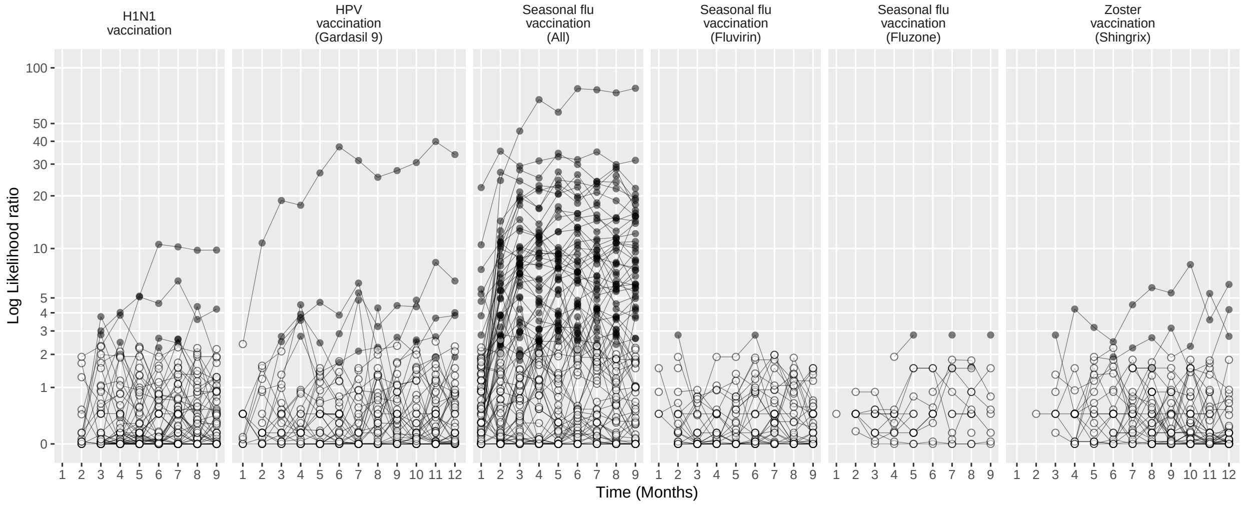

Figure 263: Negative control log likelihood ratios per month using the CaseControl method (Age & sex matched controls), in the MDCD database.

15.3 Negative controls log likelihood ratios in MDCR

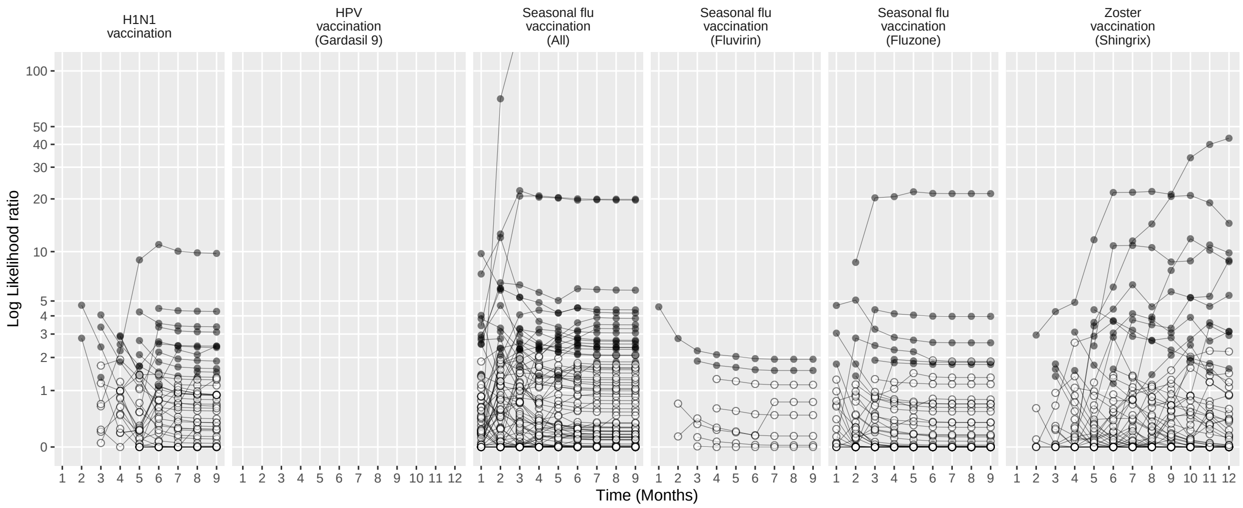

Figure 264: Negative control log likelihood ratios per month using the HistoricalComparator method (Unadjusted, using entire historic period), in the MDCR database.

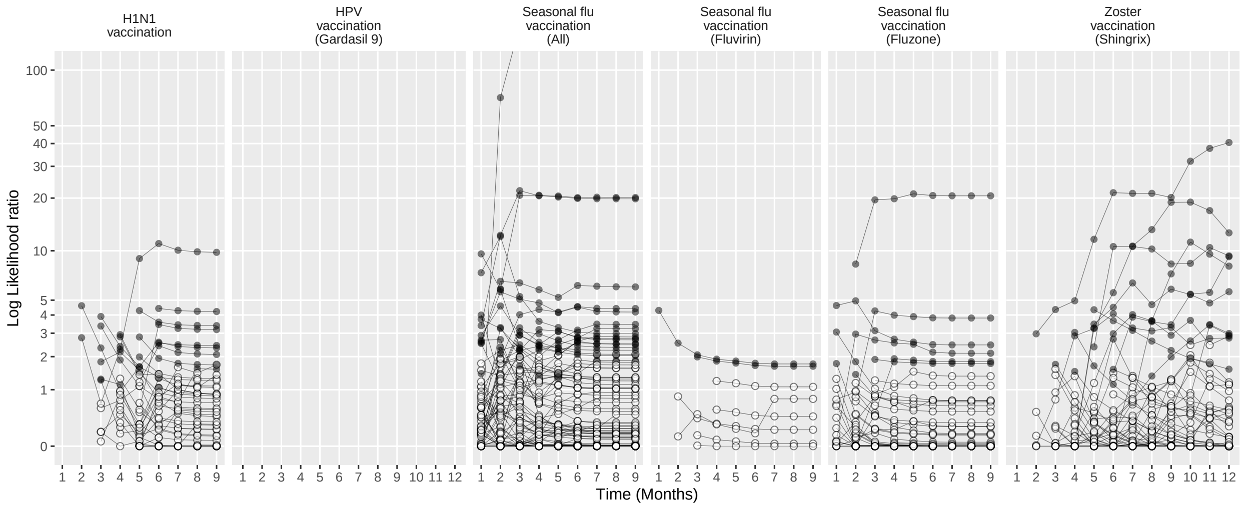

Figure 265: Negative control log likelihood ratios per month using the HistoricalComparator method (Age & sex adjusted, using entire historic period), in the MDCR database.

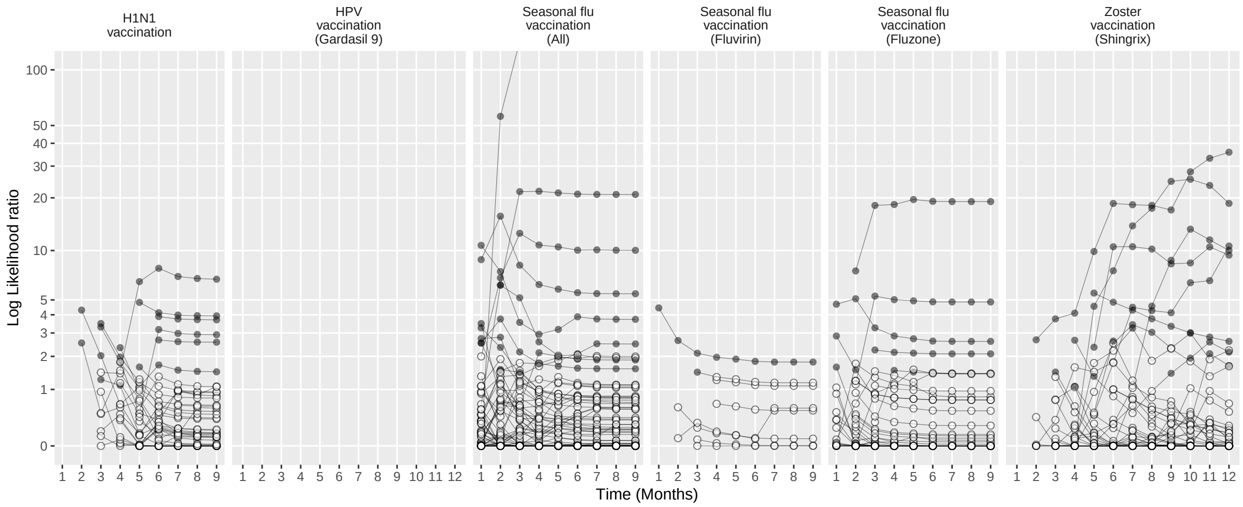

Figure 266: Negative control log likelihood ratios per month using the HistoricalComparator method (Unadjusted, using TaR after historic visit), in the MDCR database.

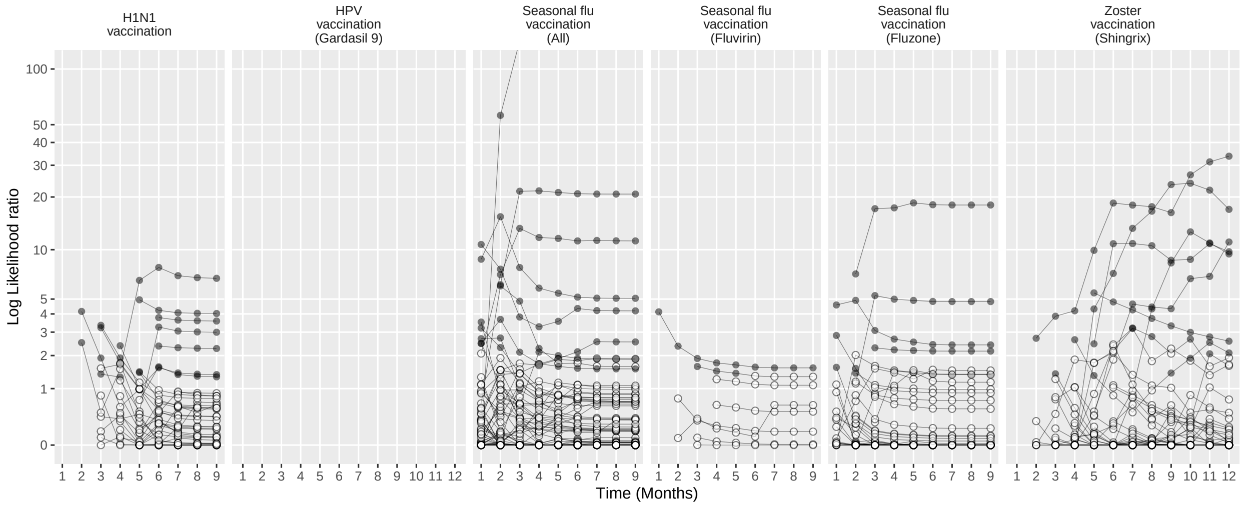

Figure 267: Negative control log likelihood ratios per month using the HistoricalComparator method (Age & sex adjusted, using TaR after historic visit), in the MDCR database.

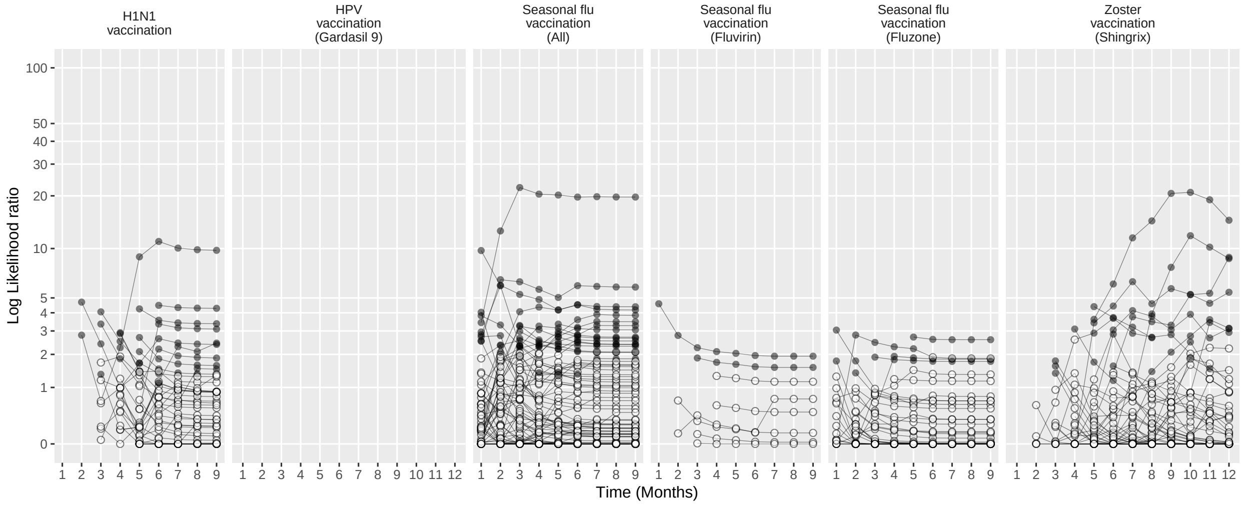

Figure 268: Negative control log likelihood ratios per month using the HistoricalComparator method (Unadjusted, using entire historic period, filtered), in the MDCR database.

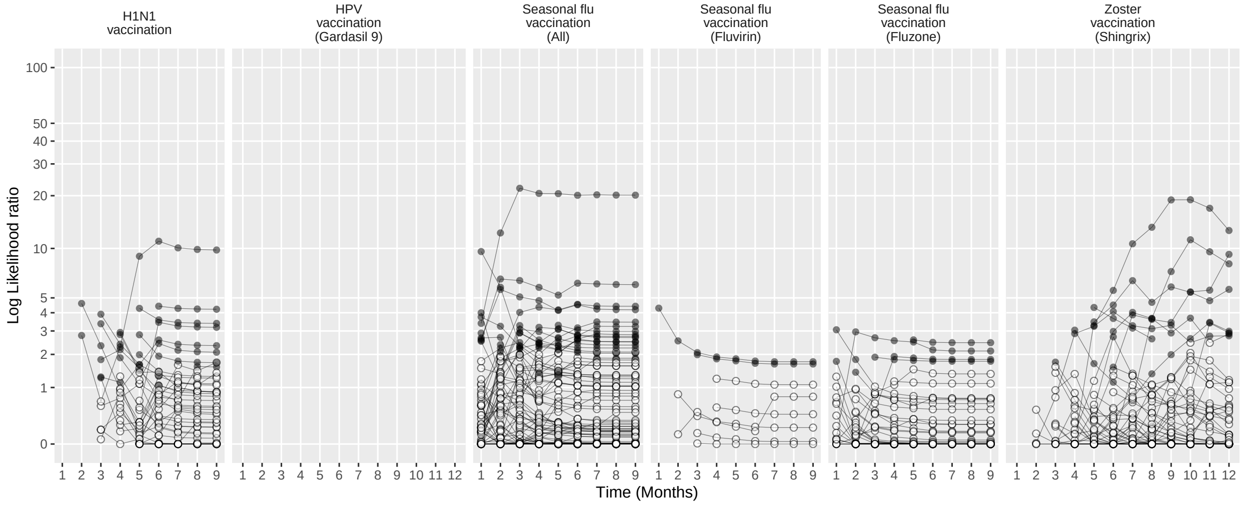

Figure 269: Negative control log likelihood ratios per month using the HistoricalComparator method (Age & sex adjusted, using entire historic period, filtered), in the MDCR database.

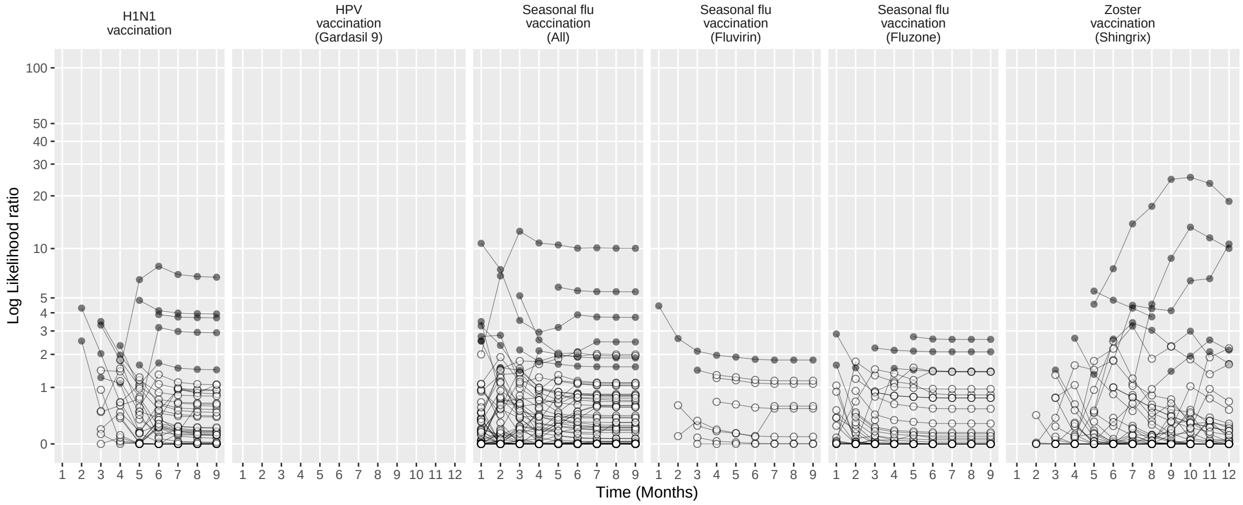

Figure 270: Negative control log likelihood ratios per month using the HistoricalComparator method (Unadjusted, using TaR after historic visit, filtered), in the MDCR database.

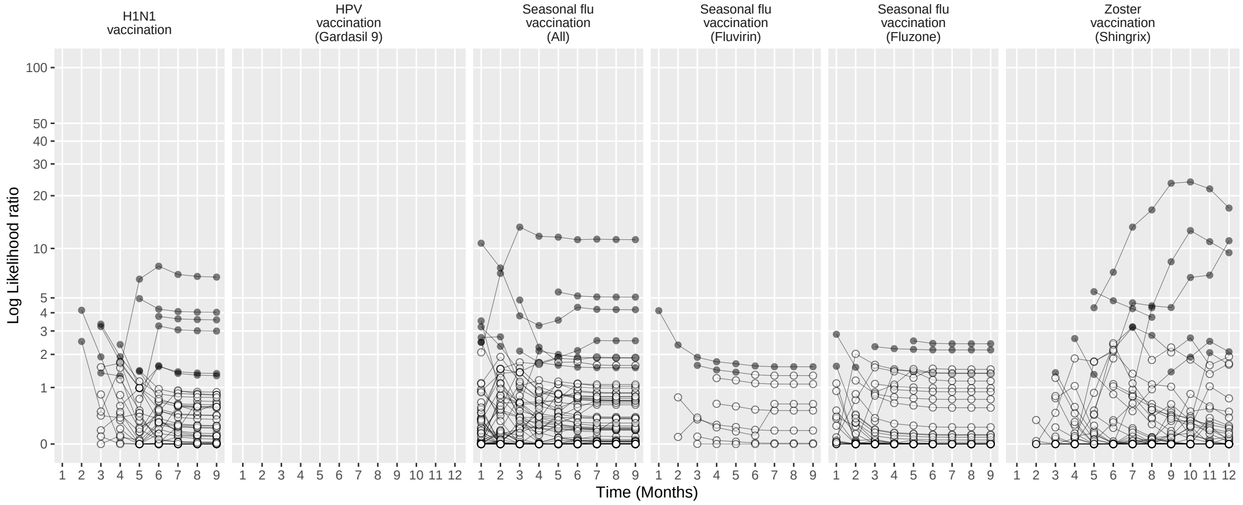

Figure 271: Negative control log likelihood ratios per month using the HistoricalComparator method (Age & sex adjusted, using TaR after historic visit, filtered), in the MDCR database.

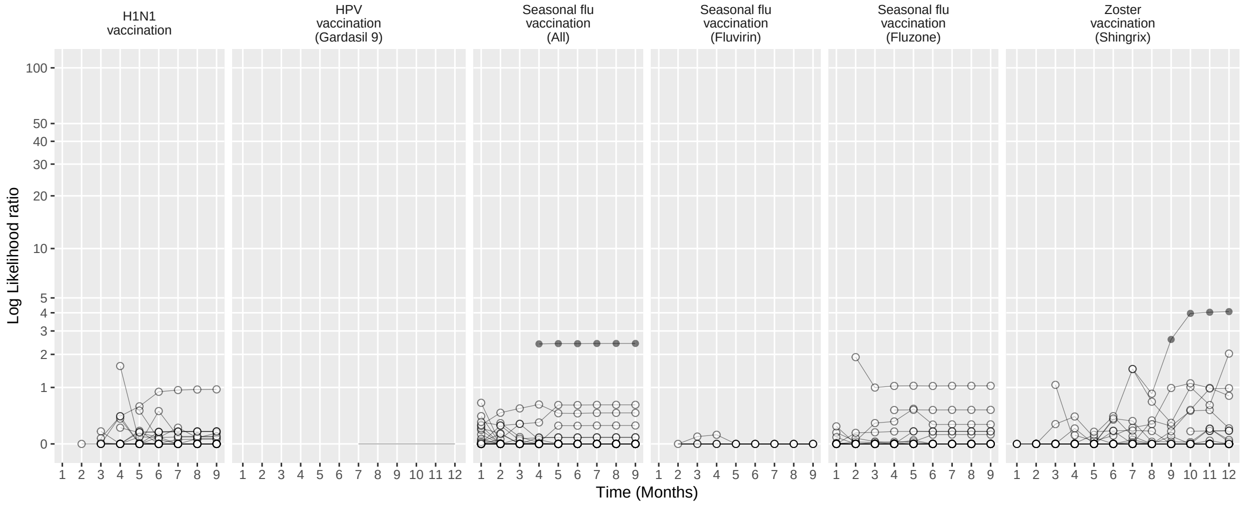

Figure 272: Negative control log likelihood ratios per month using the CohortMethod method (Unadjusted, using outpatient visits as comparator), in the MDCR database.

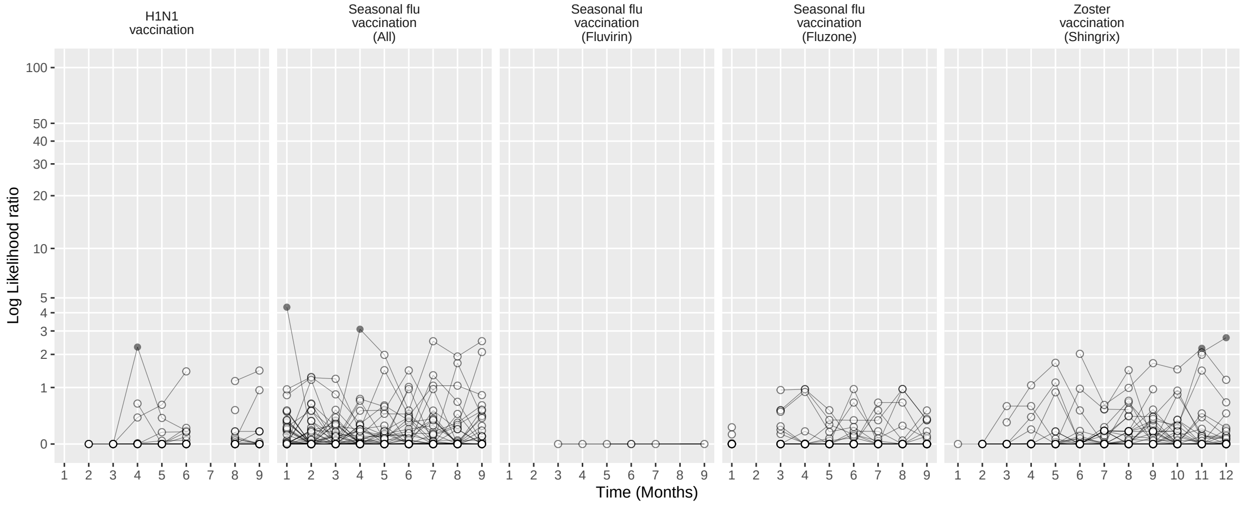

Figure 273: Negative control log likelihood ratios per month using the CohortMethod method (PS matching, using outpatient visits as comparator), in the MDCR database.

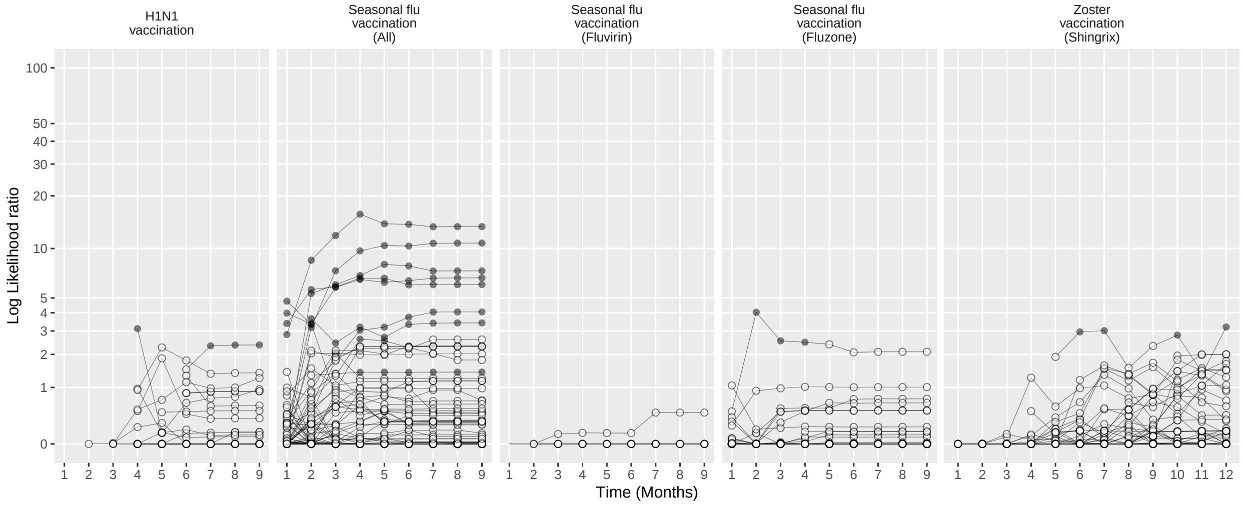

Figure 274: Negative control log likelihood ratios per month using the CohortMethod method (Unadjusted, using random days as comparator), in the MDCR database.

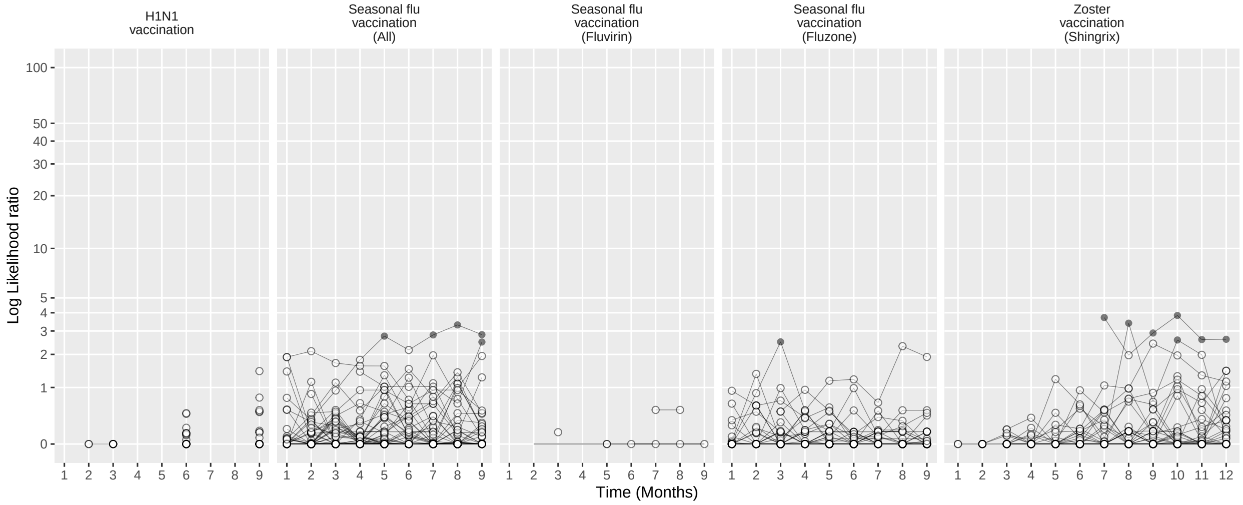

Figure 275: Negative control log likelihood ratios per month using the CohortMethod method (PS matching, using random days as comparator), in the MDCR database.

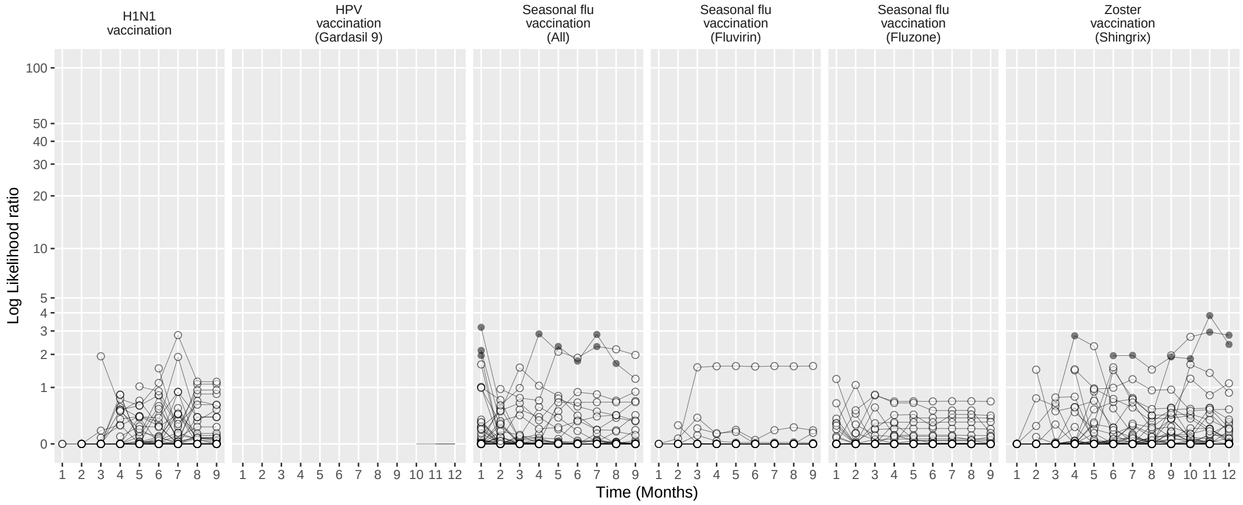

Figure 276: Negative control log likelihood ratios per month using the CohortMethod method (PS stratification, using outpatient visits as comparator), in the MDCR database.

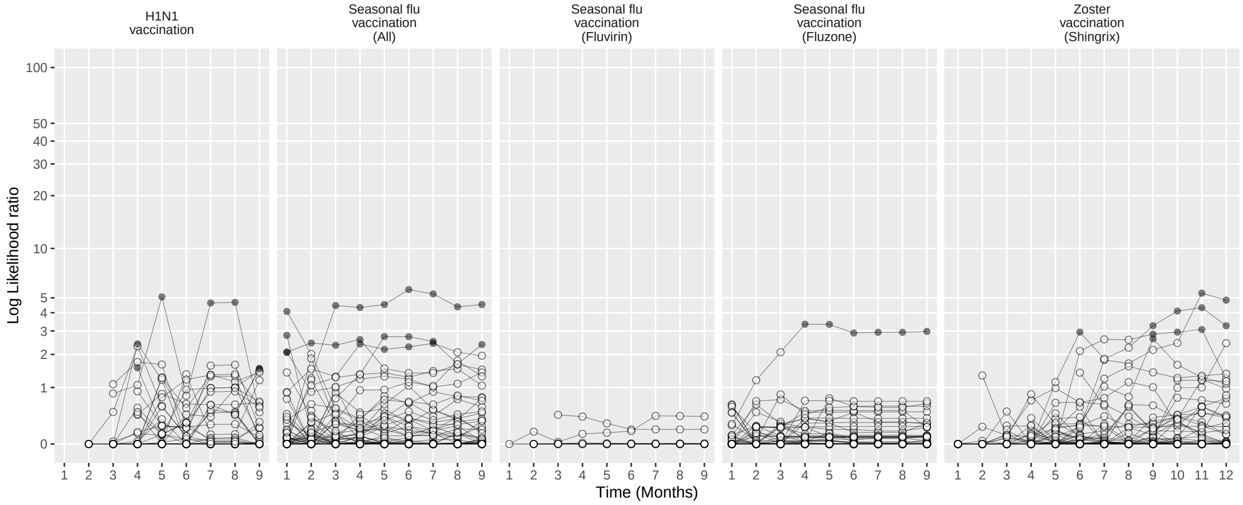

Figure 277: Negative control log likelihood ratios per month using the CohortMethod method (PS stratification, using random days as comparator), in the MDCR database.

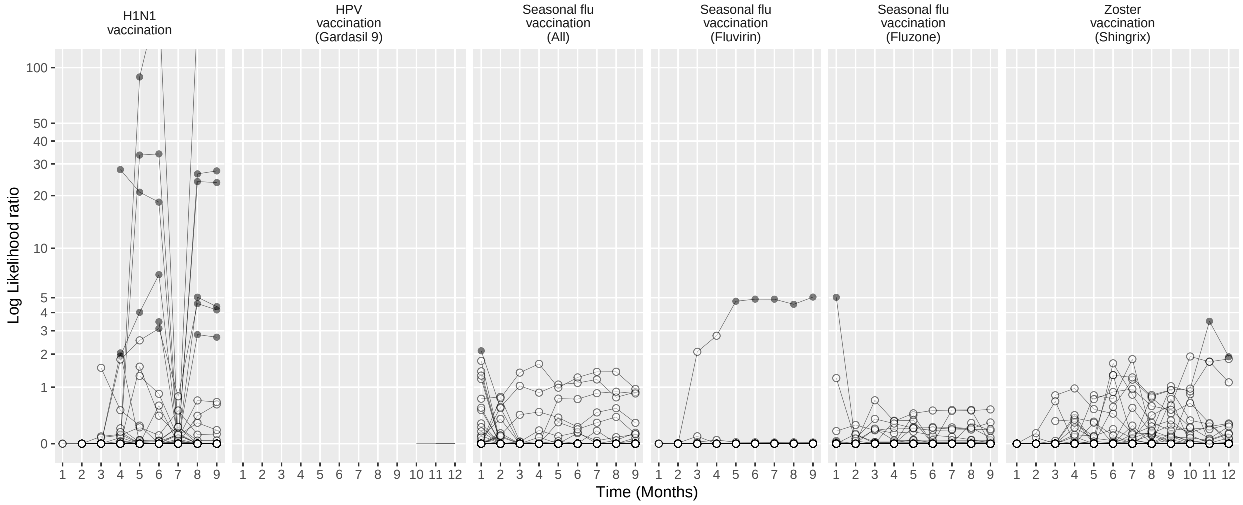

Figure 278: Negative control log likelihood ratios per month using the CohortMethod method (PS weighting, using outpatient visits as comparator), in the MDCR database.

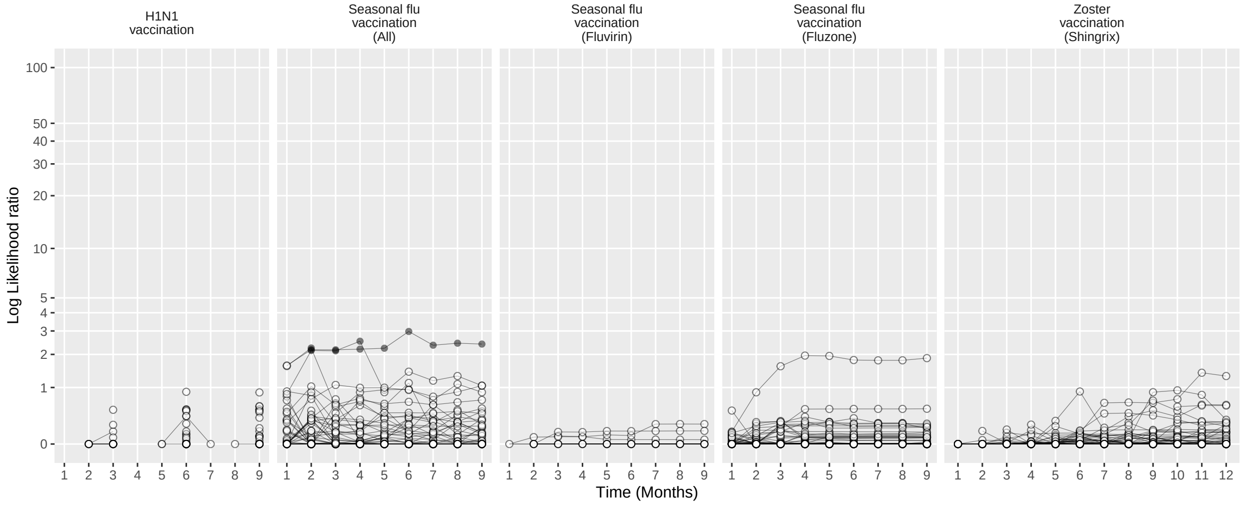

Figure 279: Negative control log likelihood ratios per month using the CohortMethod method (PS weighting, using random days as comparator), in the MDCR database.

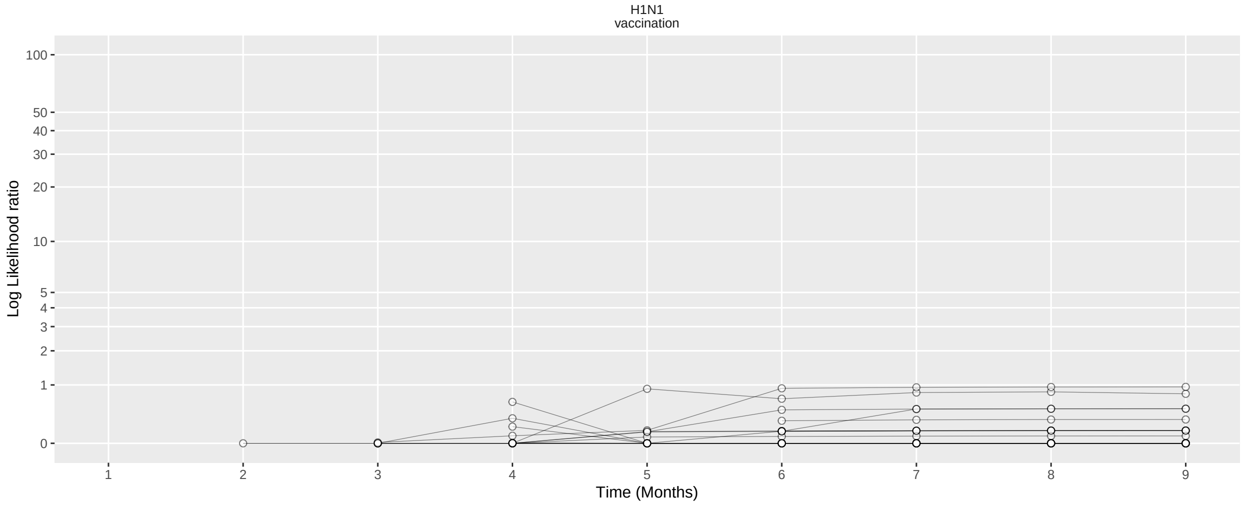

Figure 280: Negative control log likelihood ratios per month using the CohortMethod method (Per-month PS matching, using outpatient visits as comparator), in the MDCR database.

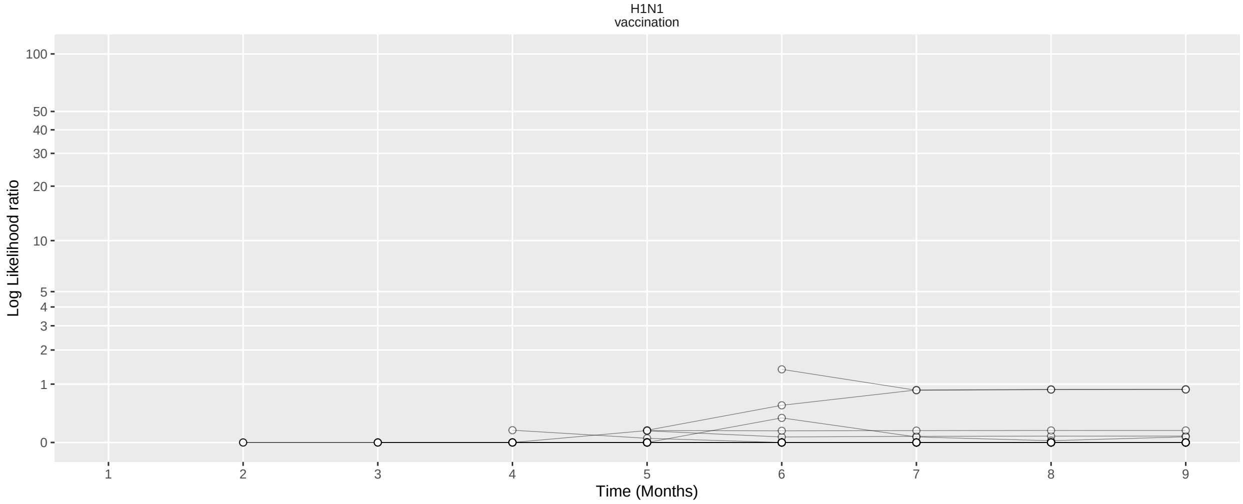

Figure 281: Negative control log likelihood ratios per month using the CohortMethod method (Per-month PS matching, using random days as comparator), in the MDCR database.

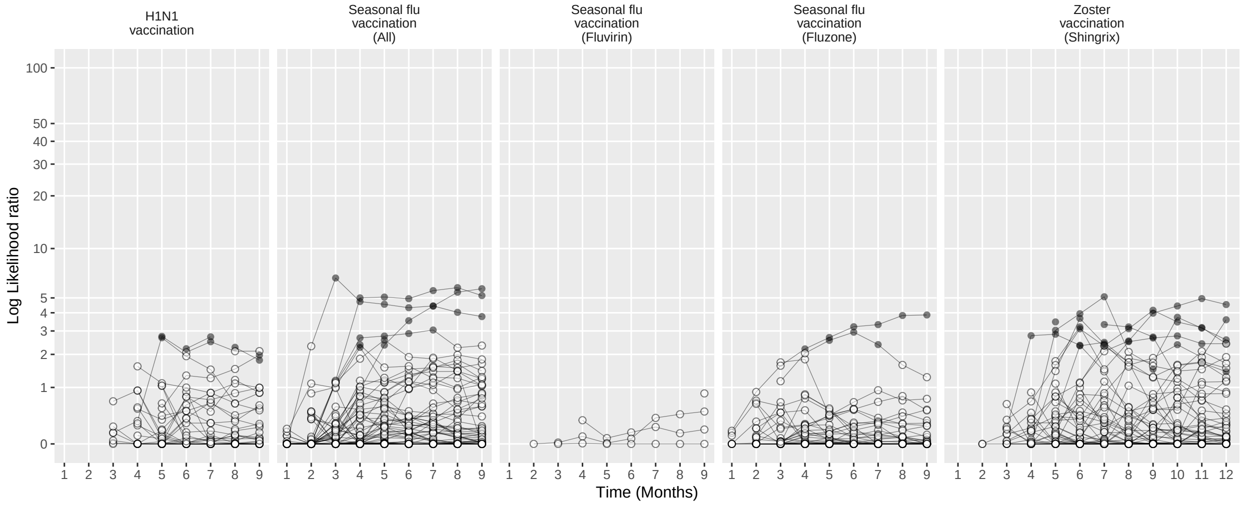

Figure 282: Negative control log likelihood ratios per month using the SCCS method (Unadjusted SCCS excluding pre-vaccination window), in the MDCR database.

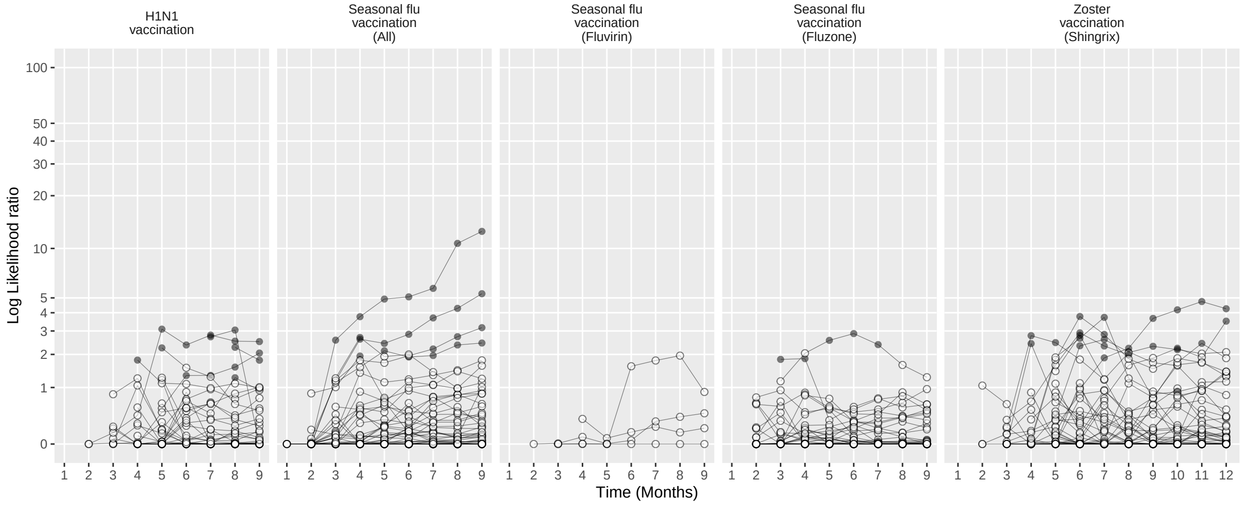

Figure 283: Negative control log likelihood ratios per month using the SCCS method (Age & season adjusted SCCS excluding pre-vaccination window), in the MDCR database.

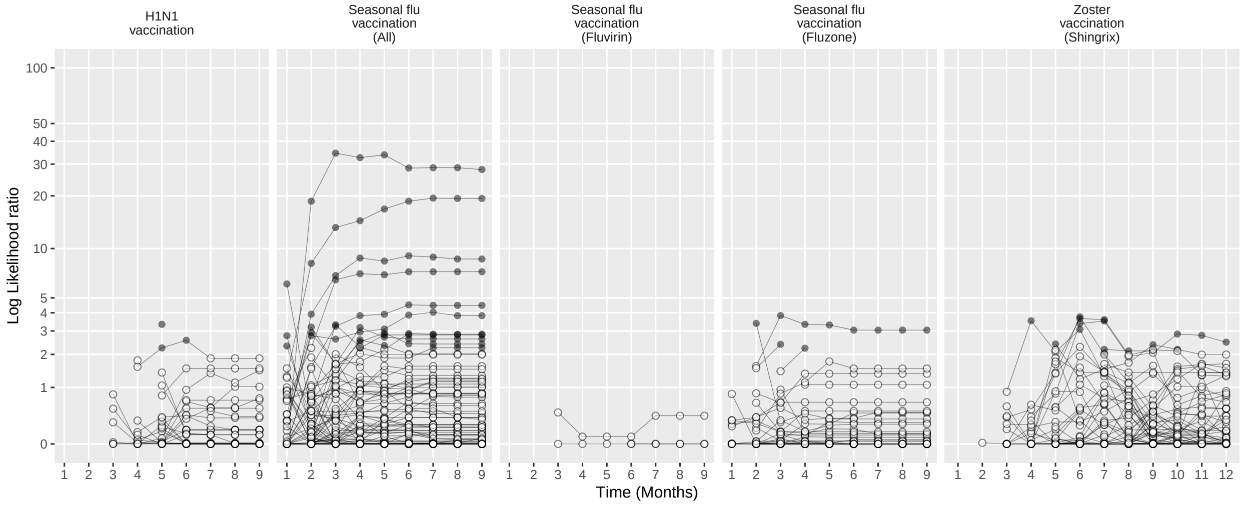

Figure 284: Negative control log likelihood ratios per month using the SCCS method (SCRI with prior control interval), in the MDCR database.

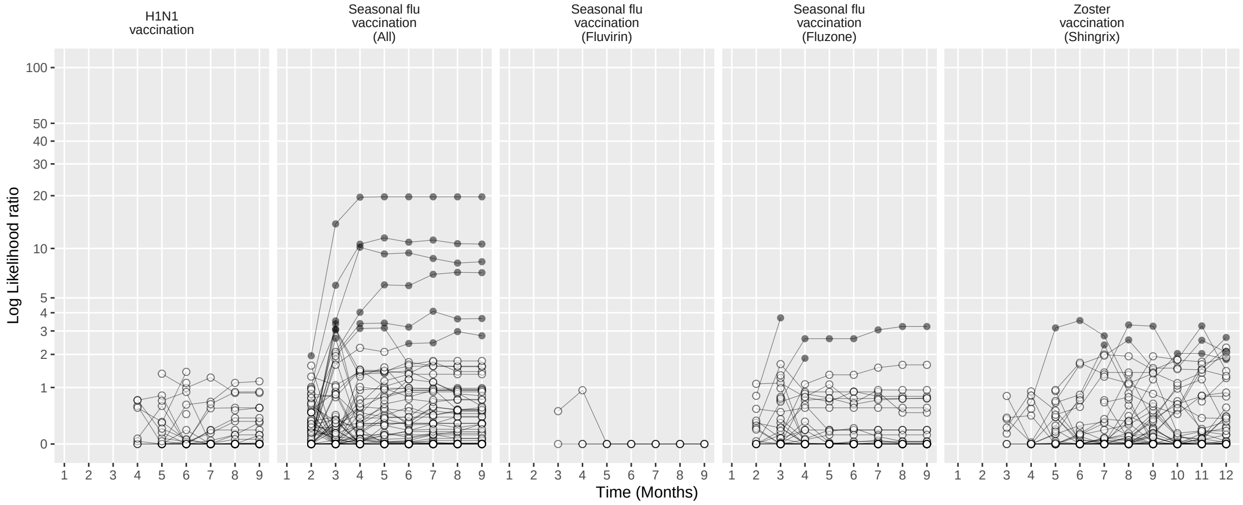

Figure 285: Negative control log likelihood ratios per month using the SCCS method (SCRI with posterior control interval), in the MDCR database.

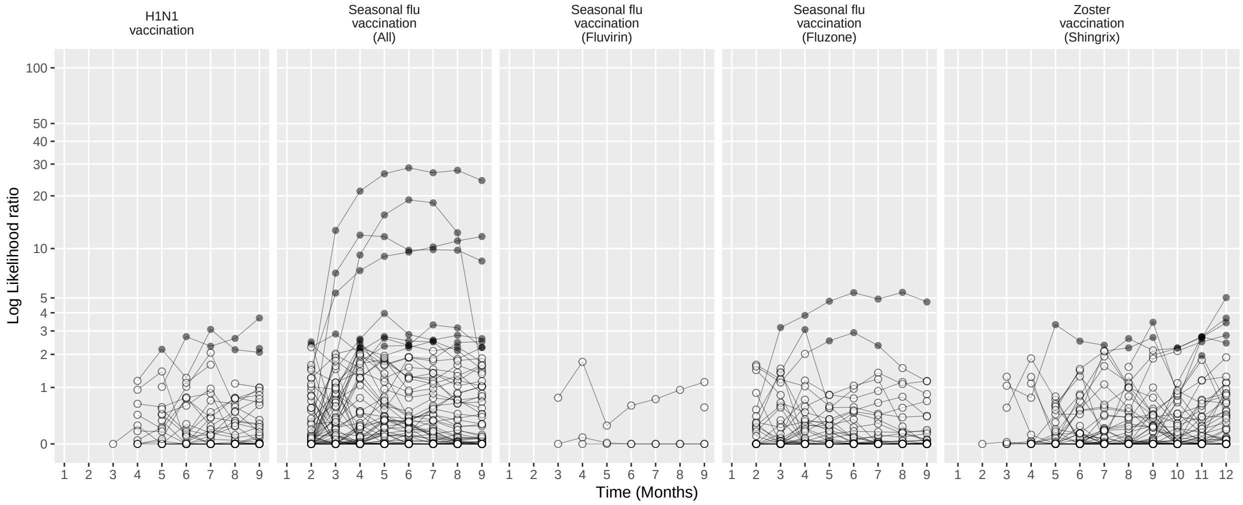

Figure 286: Negative control log likelihood ratios per month using the SCCS method (Unadjusted SCCS excluding all pre-vaccination time), in the MDCR database.

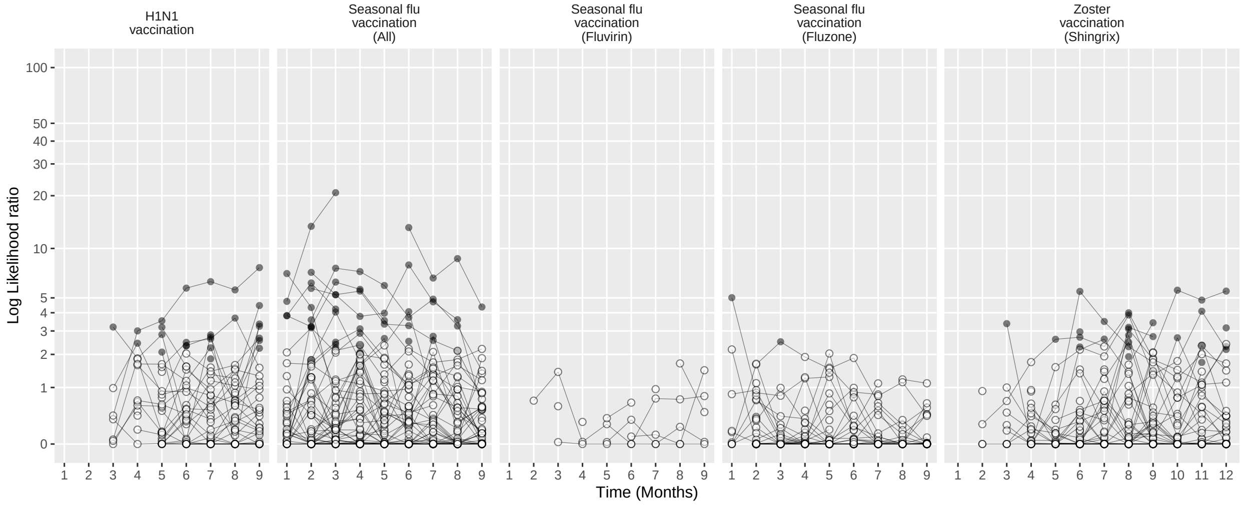

Figure 287: Negative control log likelihood ratios per month using the CaseControl method (Age & sex adjusted, using random controls), in the MDCR database.

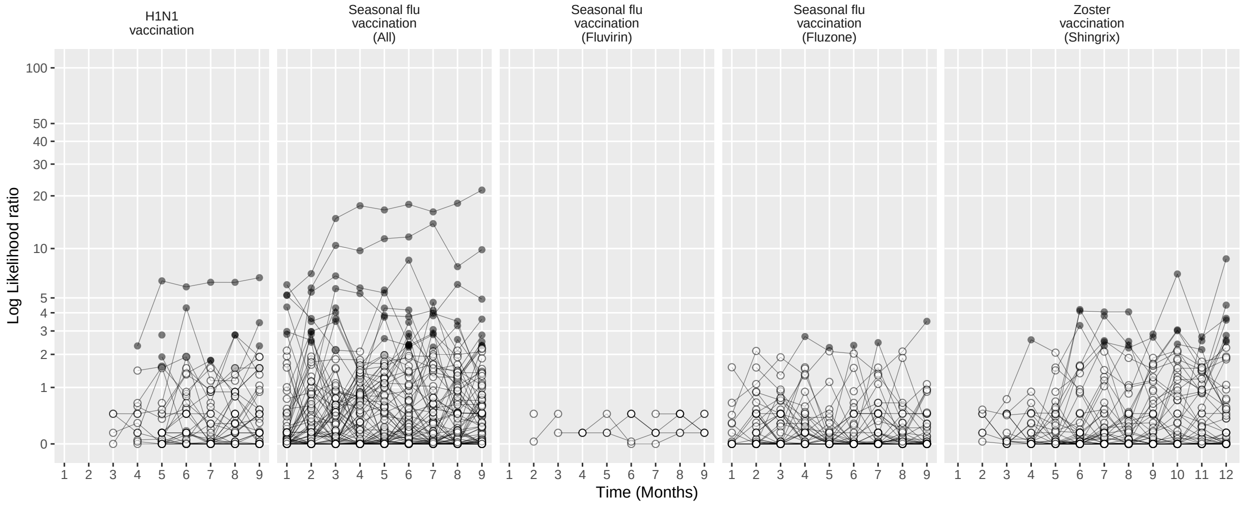

Figure 288: Negative control log likelihood ratios per month using the CaseControl method (Age & sex matched controls), in the MDCR database.

15.4 Negative controls log likelihood ratios in CCAE

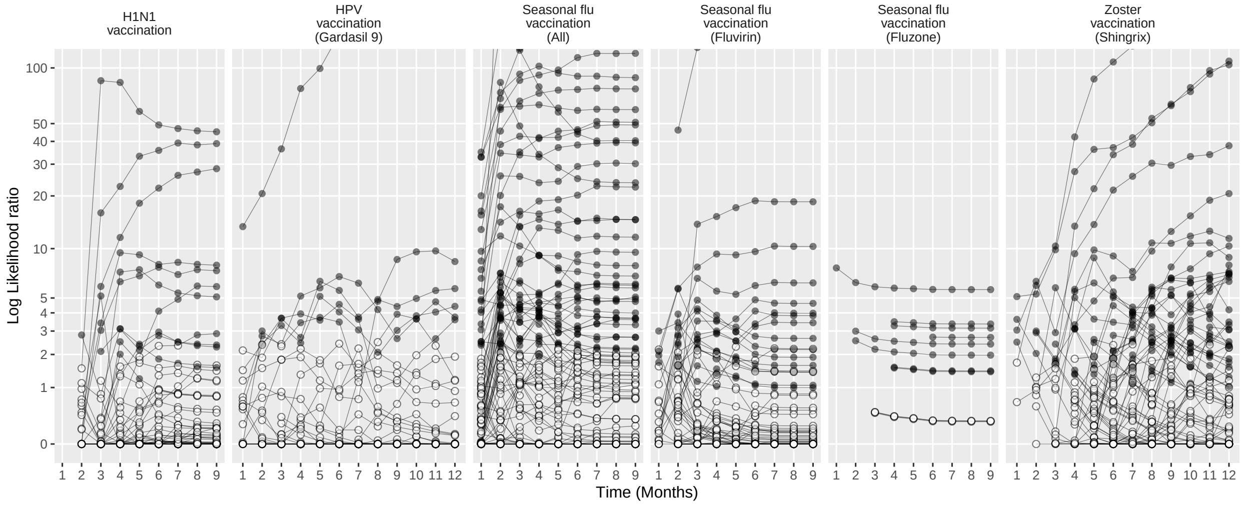

Figure 289: Negative control log likelihood ratios per month using the HistoricalComparator method (Unadjusted, using entire historic period), in the CCAE database.

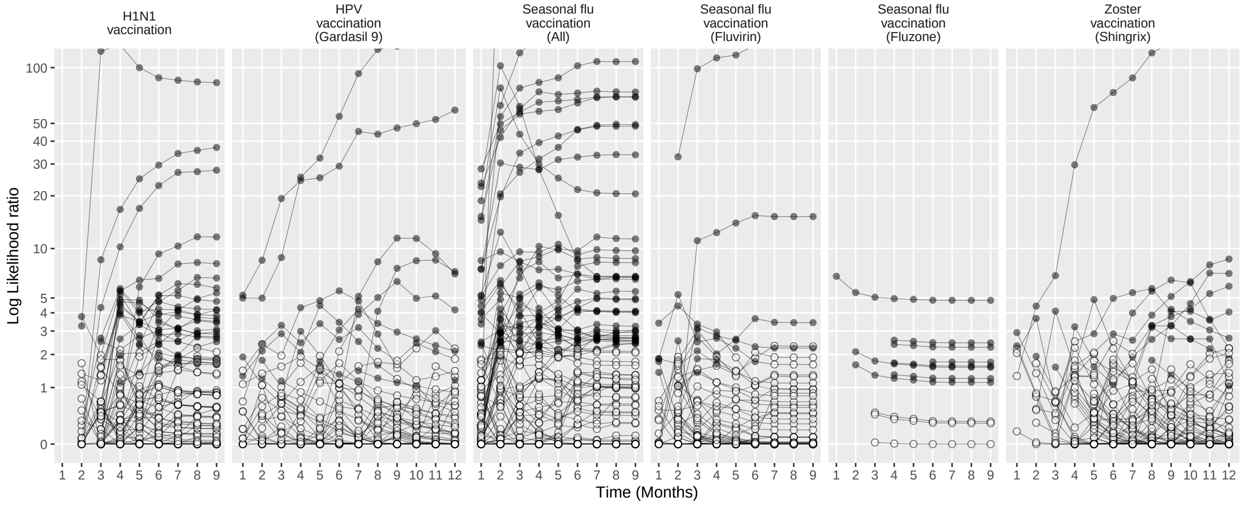

Figure 290: Negative control log likelihood ratios per month using the HistoricalComparator method (Age & sex adjusted, using entire historic period), in the CCAE database.

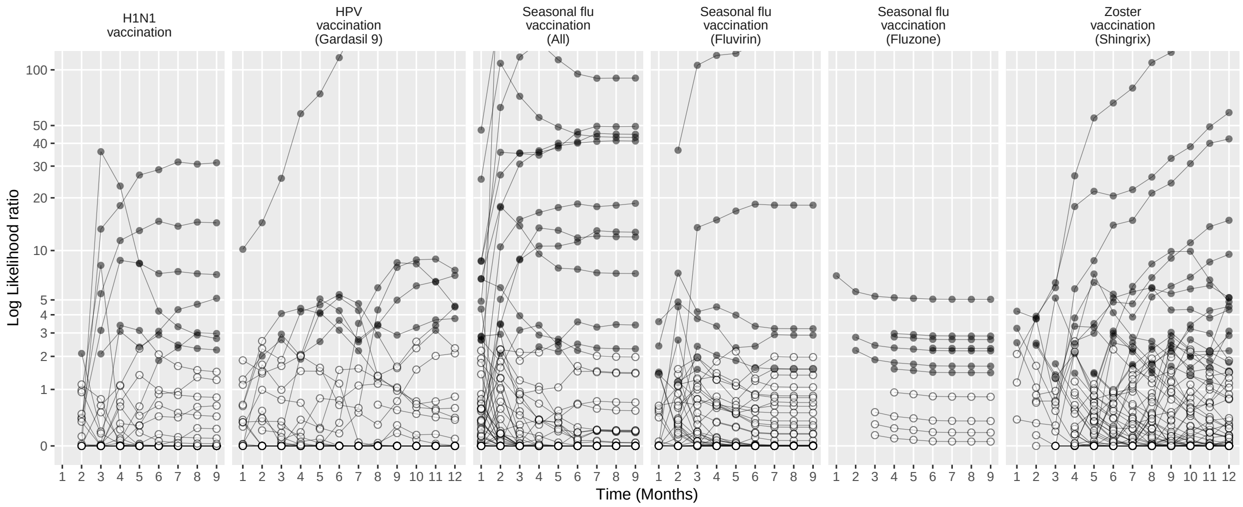

Figure 291: Negative control log likelihood ratios per month using the HistoricalComparator method (Unadjusted, using TaR after historic visit), in the CCAE database.

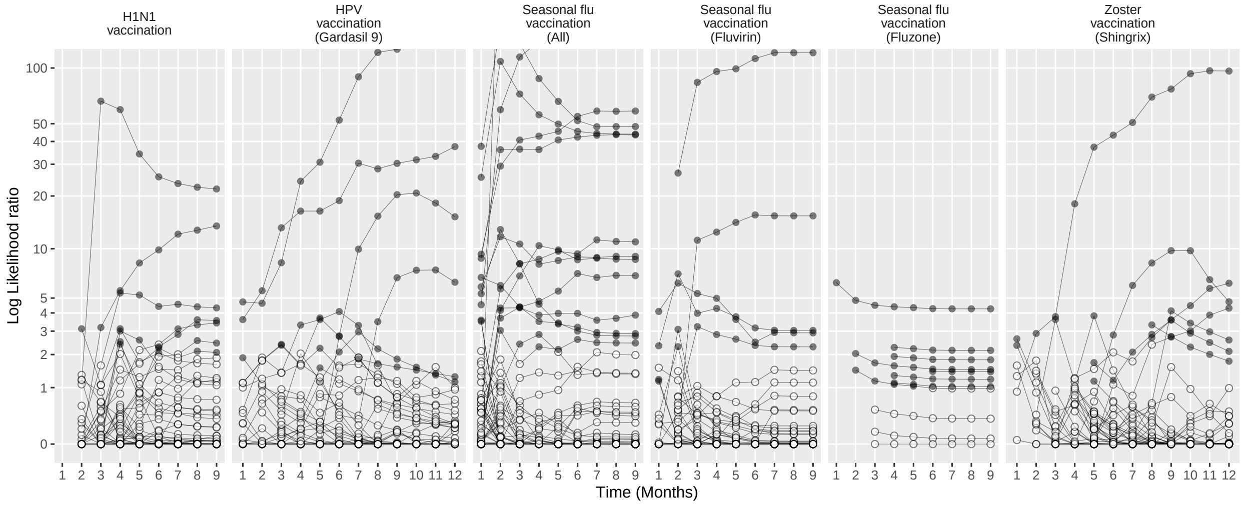

Figure 292: Negative control log likelihood ratios per month using the HistoricalComparator method (Age & sex adjusted, using TaR after historic visit), in the CCAE database.

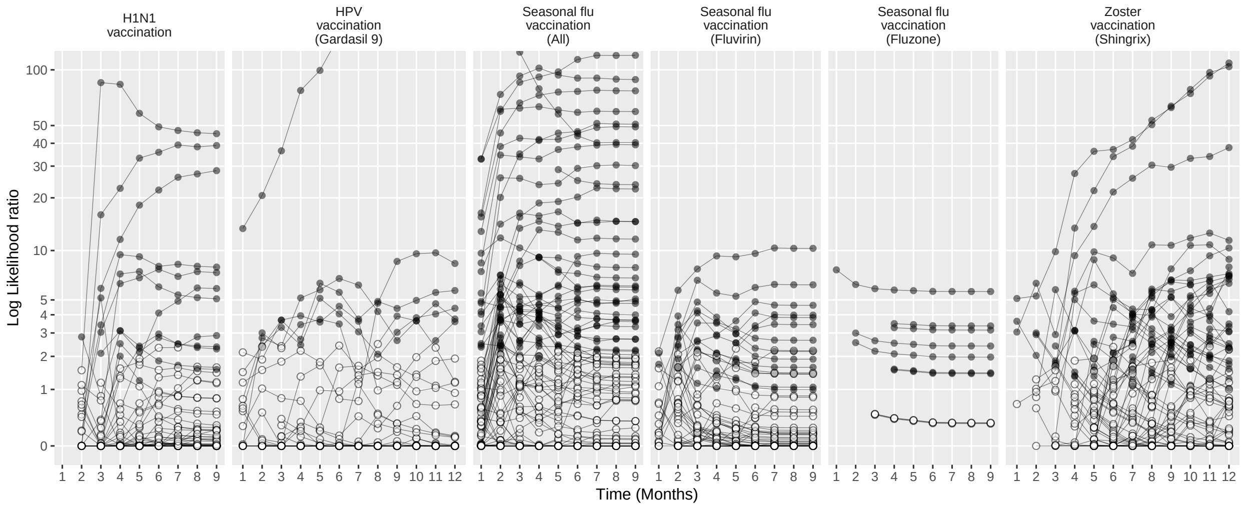

Figure 293: Negative control log likelihood ratios per month using the HistoricalComparator method (Unadjusted, using entire historic period, filtered), in the CCAE database.

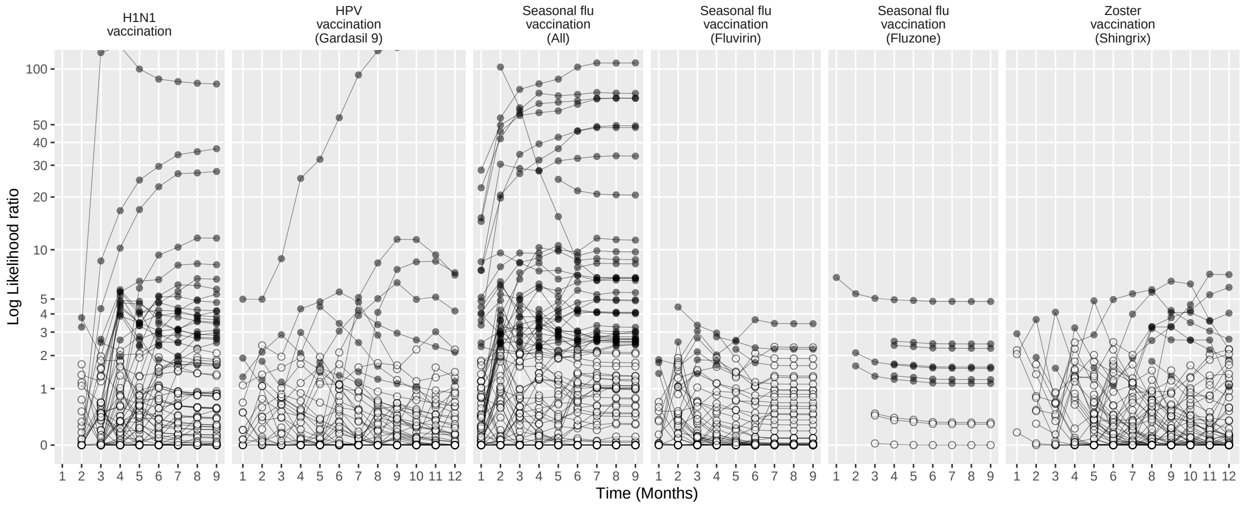

Figure 294: Negative control log likelihood ratios per month using the HistoricalComparator method (Age & sex adjusted, using entire historic period, filtered), in the CCAE database.

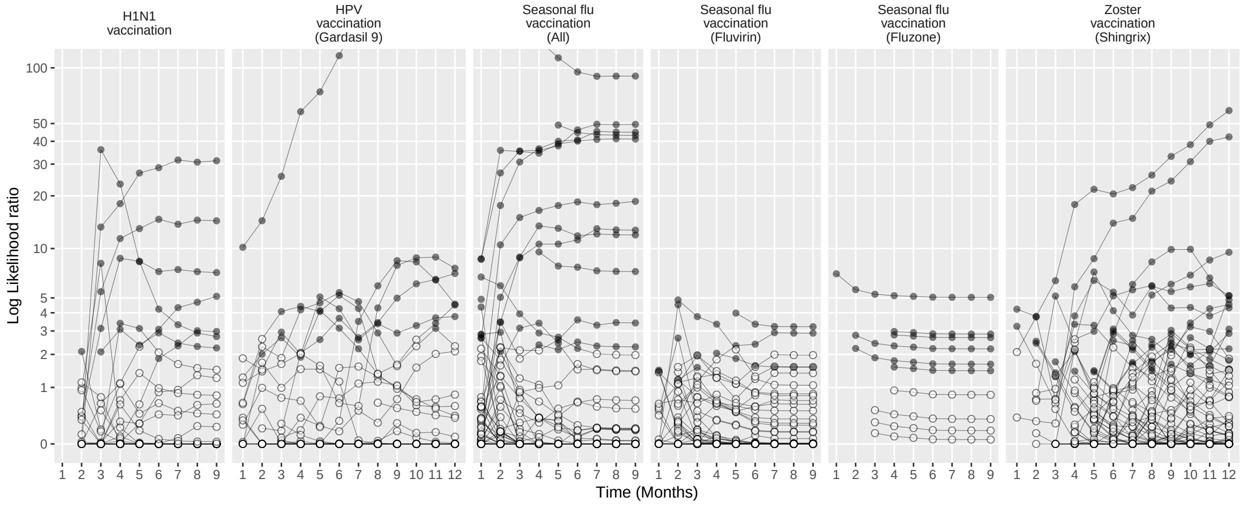

Figure 295: Negative control log likelihood ratios per month using the HistoricalComparator method (Unadjusted, using TaR after historic visit, filtered), in the CCAE database.

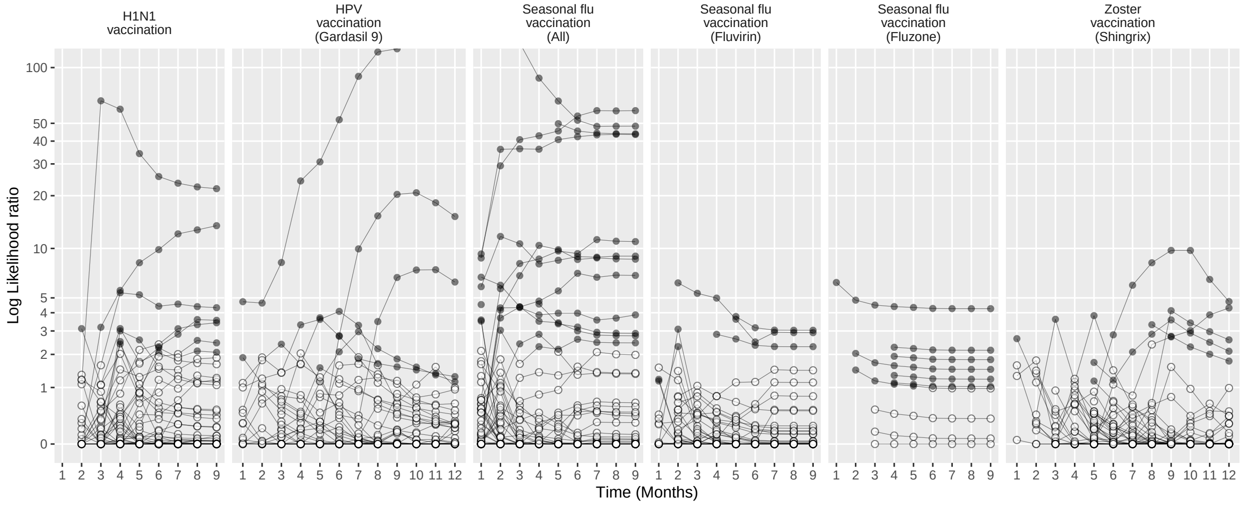

Figure 296: Negative control log likelihood ratios per month using the HistoricalComparator method (Age & sex adjusted, using TaR after historic visit, filtered), in the CCAE database.

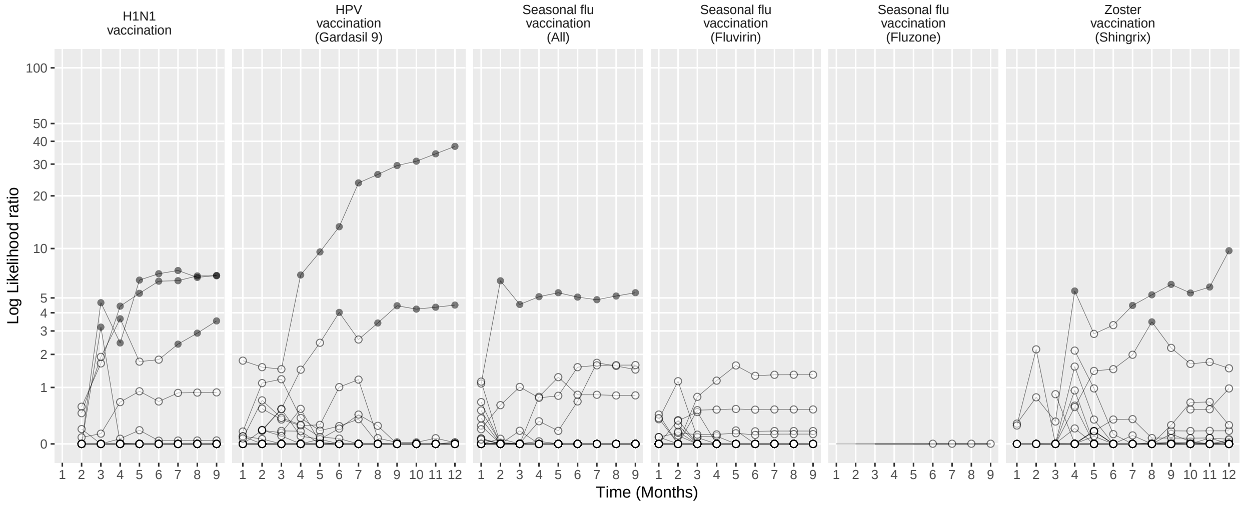

Figure 297: Negative control log likelihood ratios per month using the CohortMethod method (Unadjusted, using outpatient visits as comparator), in the CCAE database.

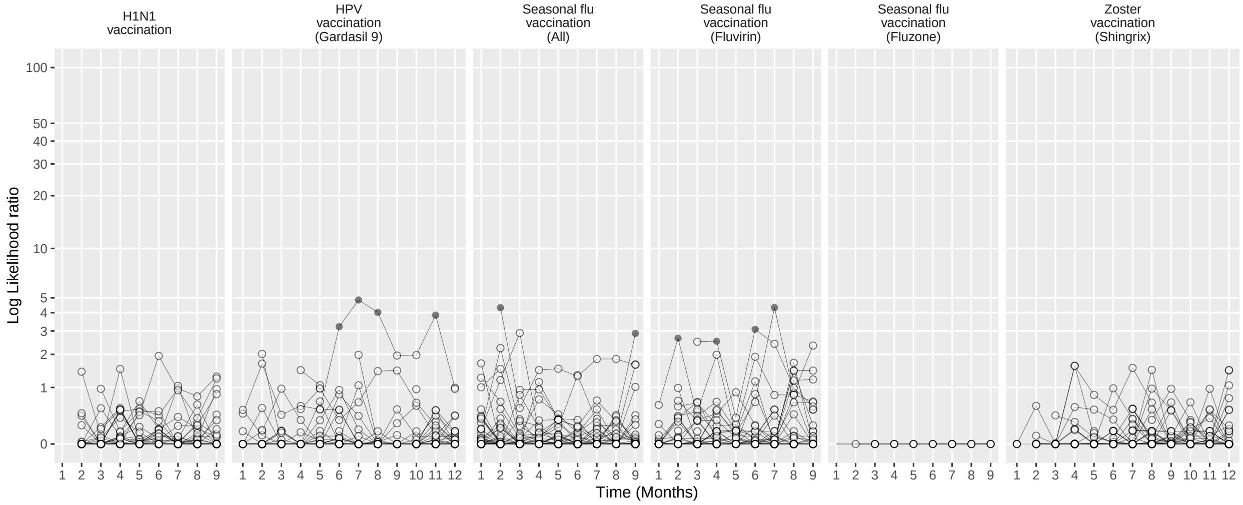

Figure 298: Negative control log likelihood ratios per month using the CohortMethod method (PS matching, using outpatient visits as comparator), in the CCAE database.

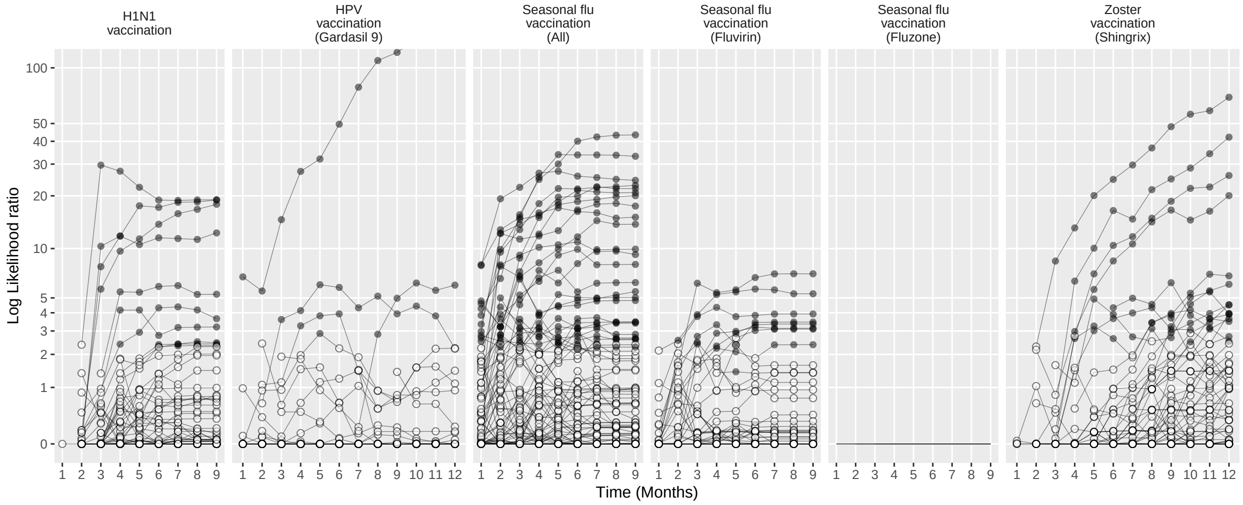

Figure 299: Negative control log likelihood ratios per month using the CohortMethod method (Unadjusted, using random days as comparator), in the CCAE database.

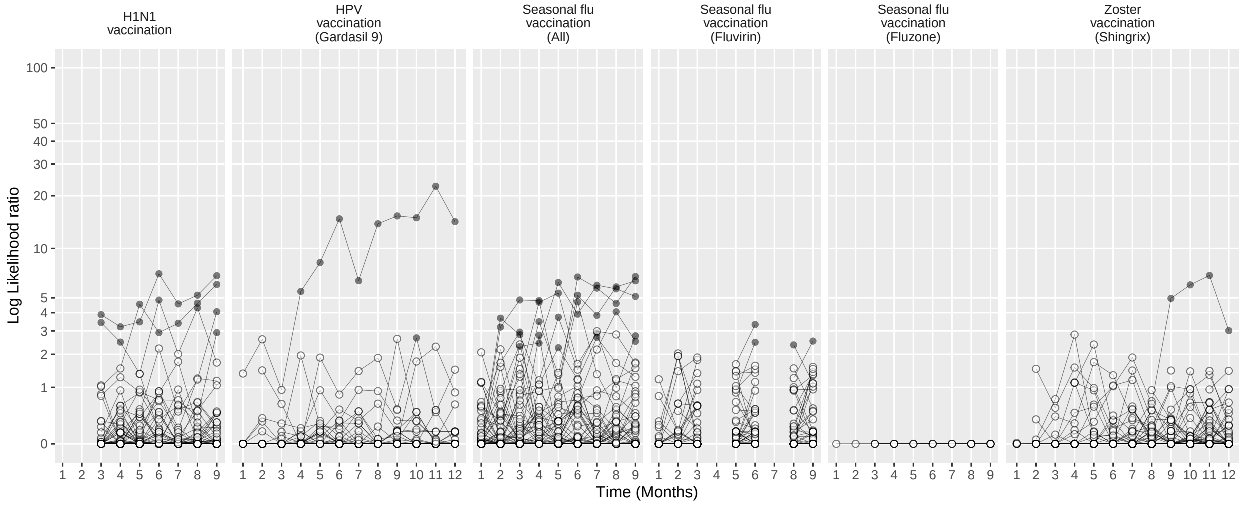

Figure 300: Negative control log likelihood ratios per month using the CohortMethod method (PS matching, using random days as comparator), in the CCAE database.

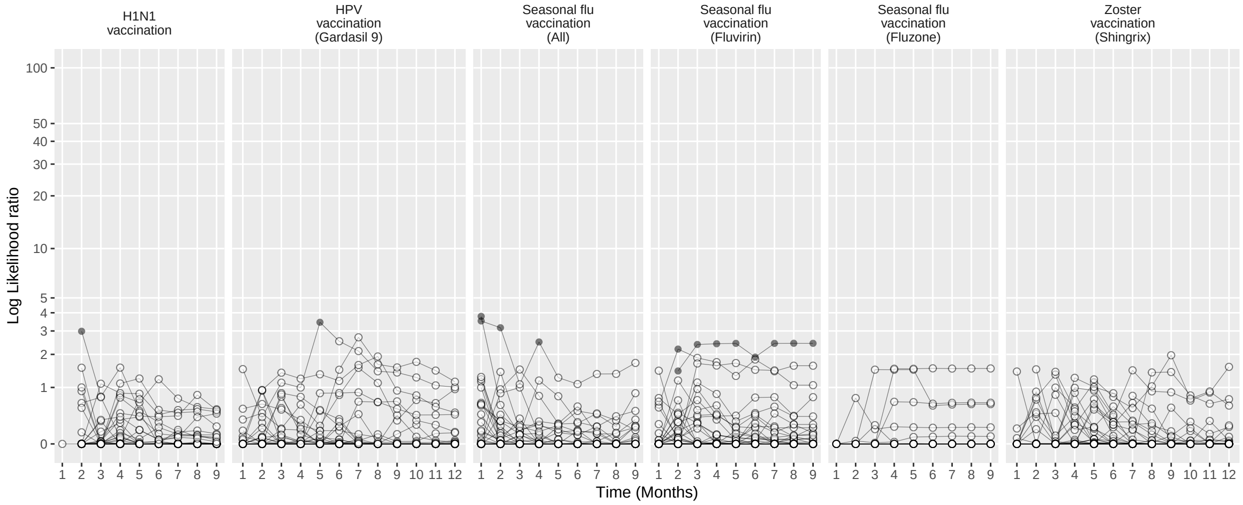

Figure 301: Negative control log likelihood ratios per month using the CohortMethod method (PS stratification, using outpatient visits as comparator), in the CCAE database.

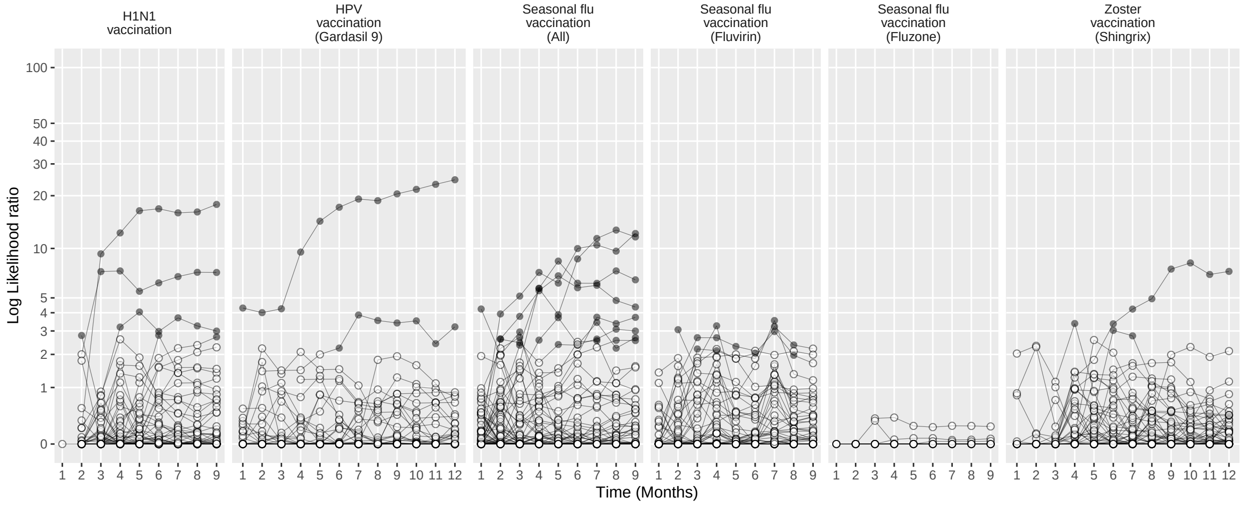

Figure 302: Negative control log likelihood ratios per month using the CohortMethod method (PS stratification, using random days as comparator), in the CCAE database.

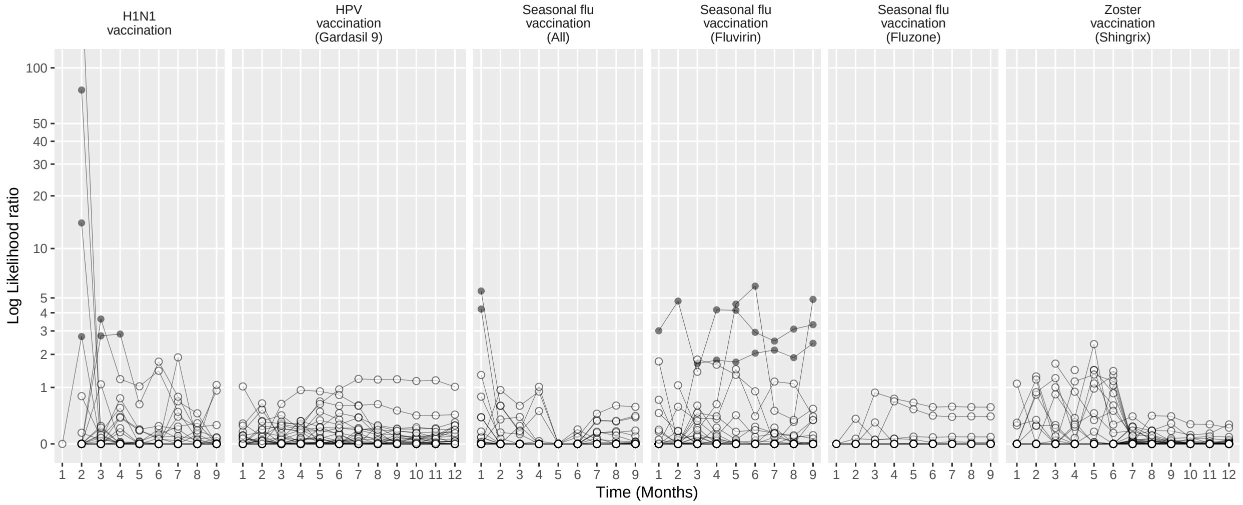

Figure 303: Negative control log likelihood ratios per month using the CohortMethod method (PS weighting, using outpatient visits as comparator), in the CCAE database.

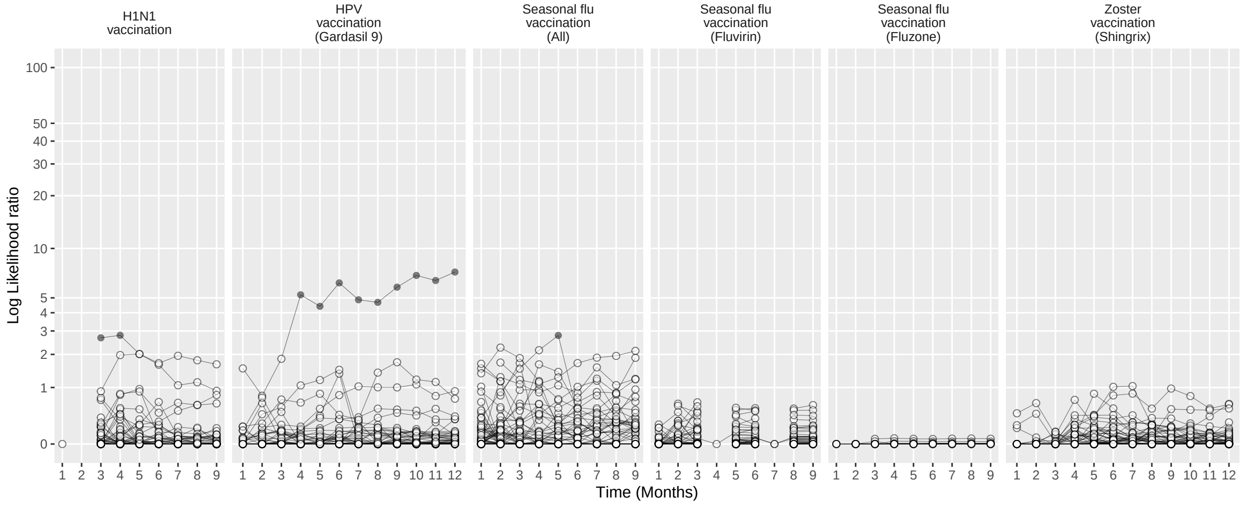

Figure 304: Negative control log likelihood ratios per month using the CohortMethod method (PS weighting, using random days as comparator), in the CCAE database.

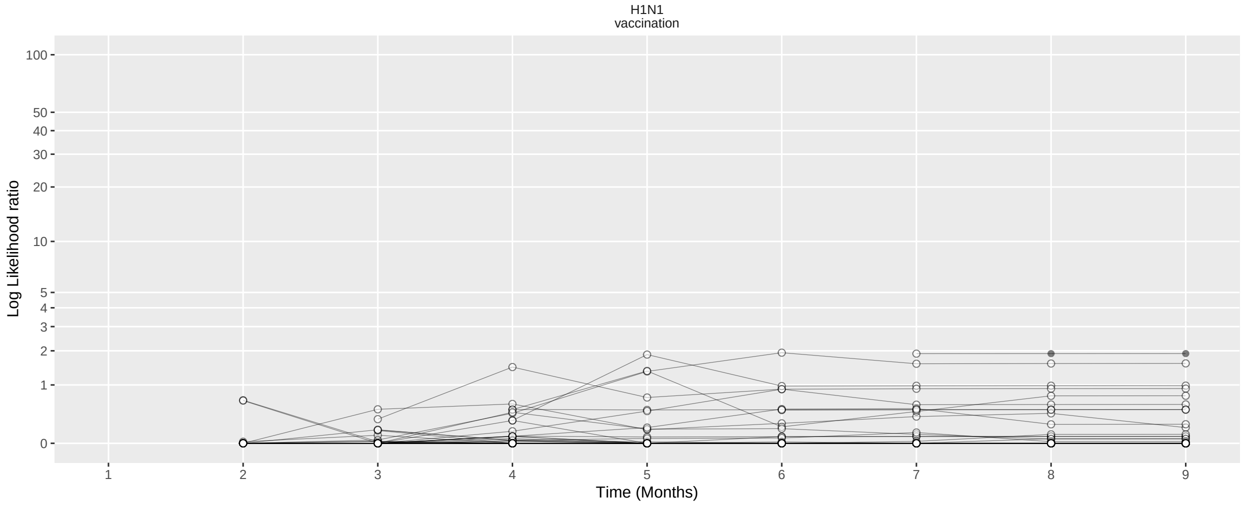

Figure 305: Negative control log likelihood ratios per month using the CohortMethod method (Per-month PS matching, using outpatient visits as comparator), in the CCAE database.

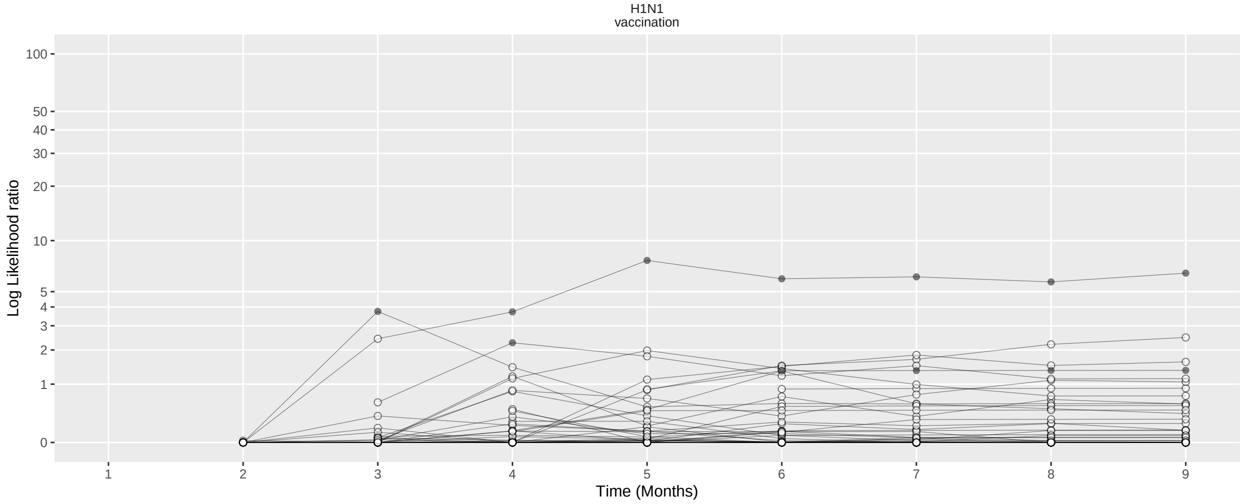

Figure 306: Negative control log likelihood ratios per month using the CohortMethod method (Per-month PS matching, using random days as comparator), in the CCAE database.

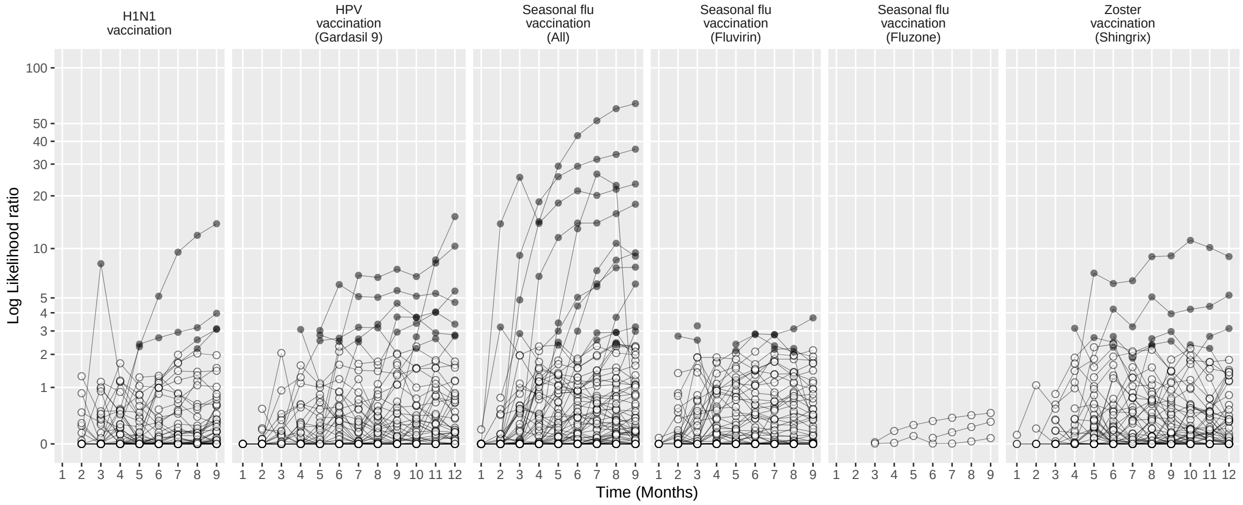

Figure 307: Negative control log likelihood ratios per month using the SCCS method (Unadjusted SCCS excluding pre-vaccination window), in the CCAE database.

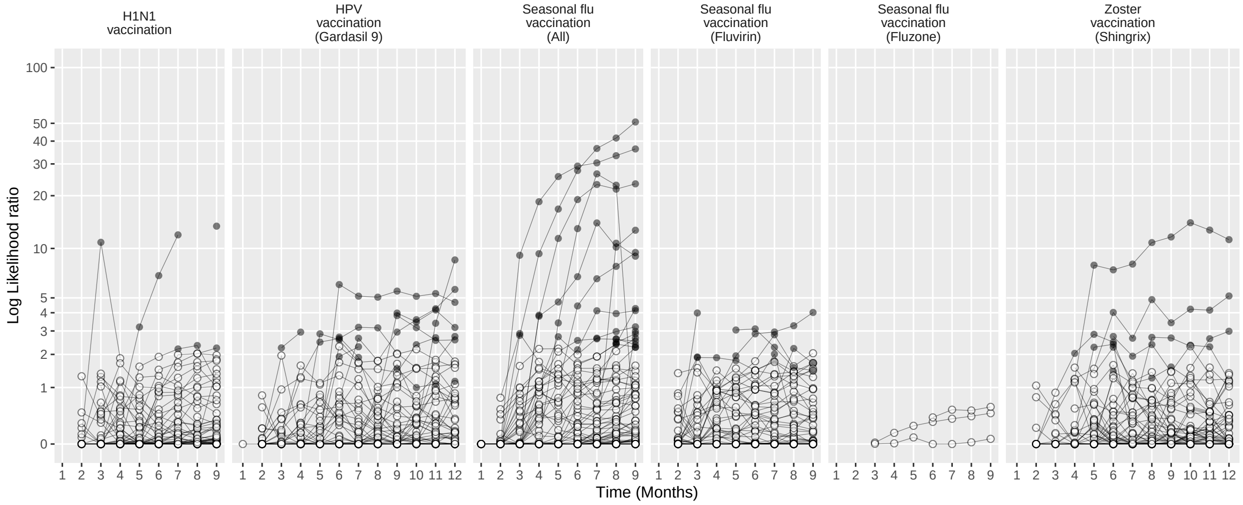

Figure 308: Negative control log likelihood ratios per month using the SCCS method (Age & season adjusted SCCS excluding pre-vaccination window), in the CCAE database.

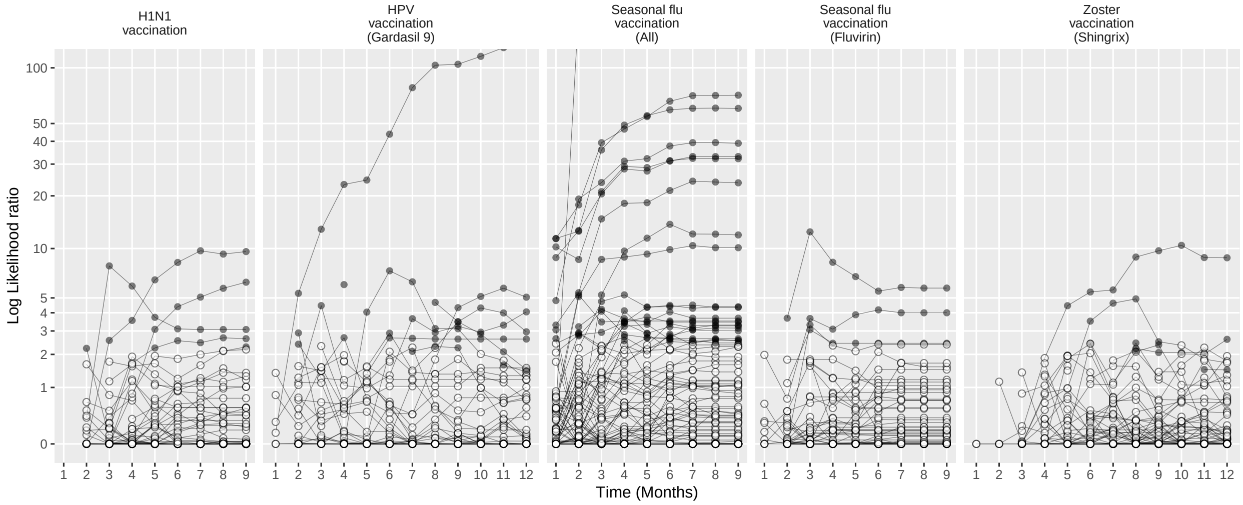

Figure 309: Negative control log likelihood ratios per month using the SCCS method (SCRI with prior control interval), in the CCAE database.

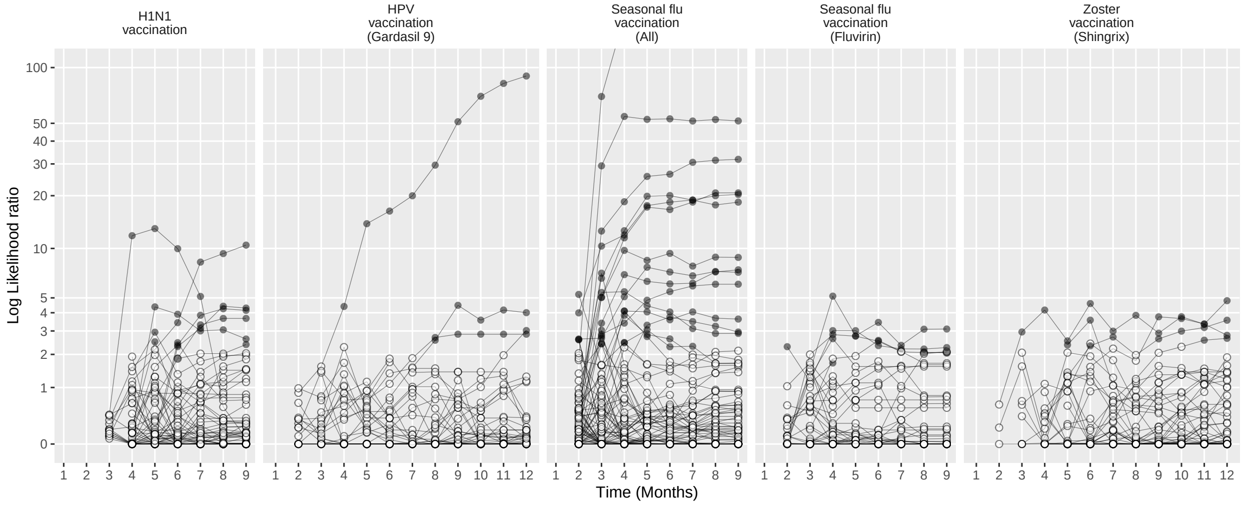

Figure 310: Negative control log likelihood ratios per month using the SCCS method (SCRI with posterior control interval), in the CCAE database.

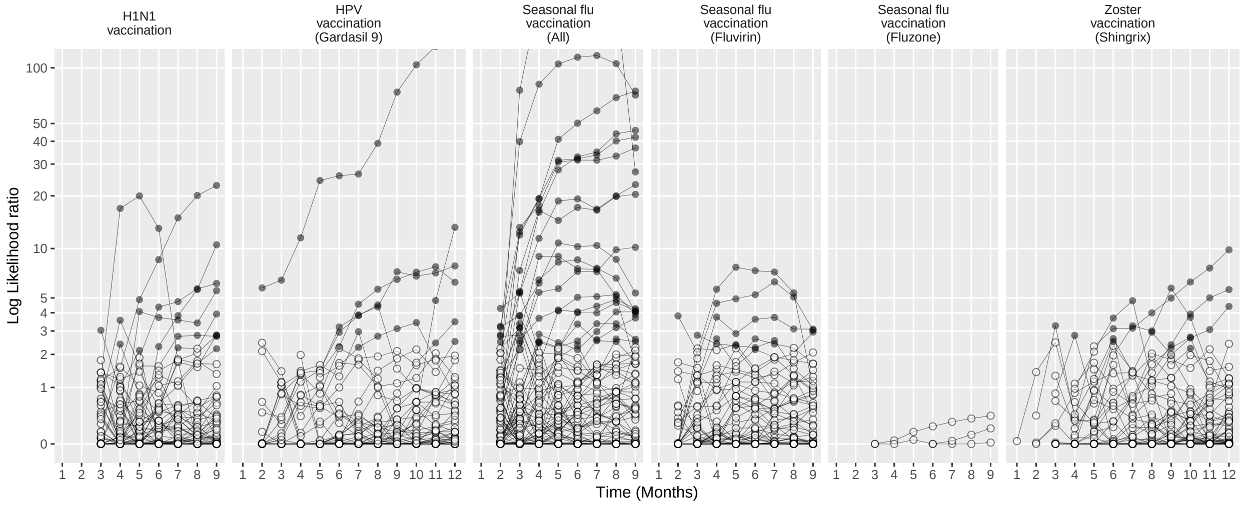

Figure 311: Negative control log likelihood ratios per month using the SCCS method (Unadjusted SCCS excluding all pre-vaccination time), in the CCAE database.

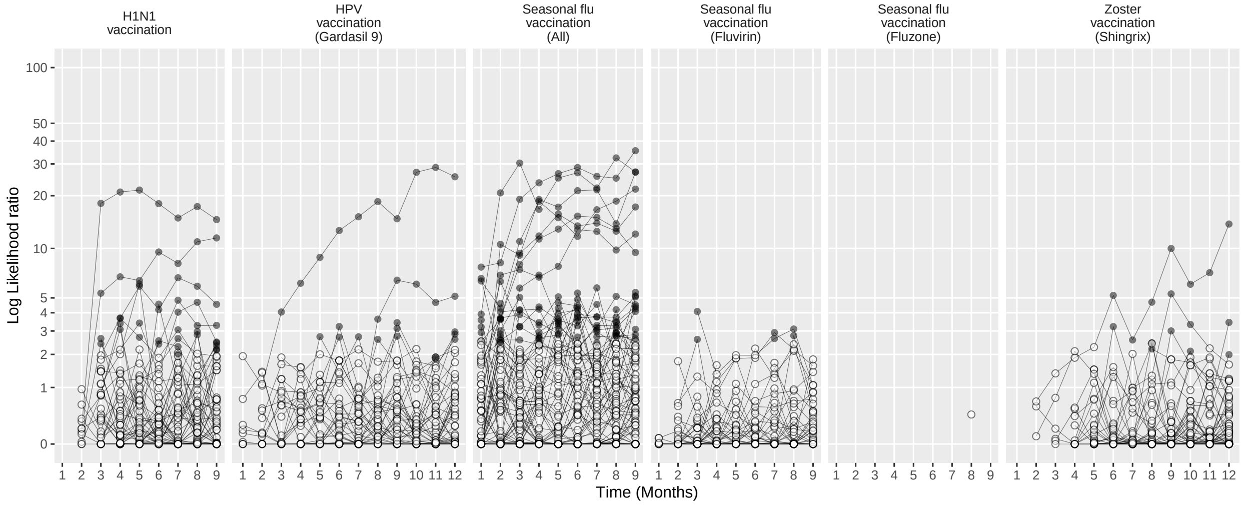

Figure 312: Negative control log likelihood ratios per month using the CaseControl method (Age & sex adjusted, using random controls), in the CCAE database.

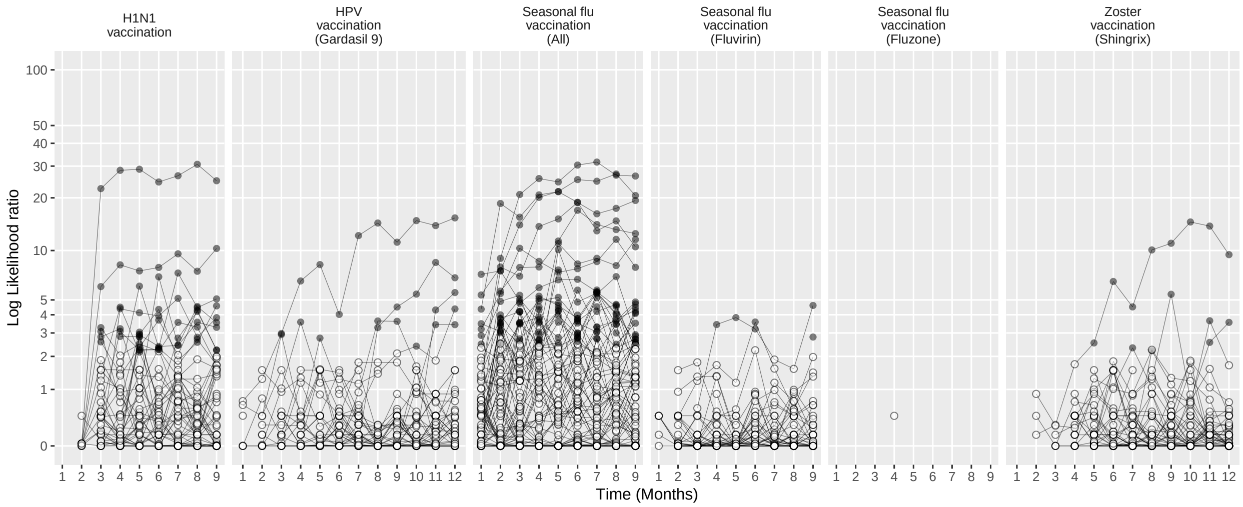

Figure 313: Negative control log likelihood ratios per month using the CaseControl method (Age & sex matched controls), in the CCAE database.

References

1 Tian Y, Schuemie MJ, Suchard MA. Evaluating large-scale propensity score performance through real-world and synthetic data experiments. *International journal of epidemiology* 2018;**47**:2005–14.

2 Izurieta HS, Lu M, Kelman J, *et al.* Comparative effectiveness of influenza vaccines among U.S. Medicare beneficiaries ages 65 years and older during the 2019-20 season. *Clin Infect Dis* 2020.

3 Whitaker HJ, Farrington CP, Spiessens B, *et al.* Tutorial in biostatistics: the self-controlled case series method. *Stat Med* 2006;**25**:1768–97.

4 Glanz JM, McClure DL, Xu S, *et al.* Four different study designs to evaluate vaccine safety were equally validated with contrasting limitations. *J Clin Epidemiol* 2006;**59**:808–18.

5 Kulldorff M, Davis RL, Kolczak† M, *et al.* A maximized sequential probability ratio test for drug and vaccine safety surveillance. *Sequential Analysis* 2011;**30**:58–78. doi:10.1080/07474946.2011.539924

6 Schuemie MJ, Ryan PB, DuMouchel W, *et al.* Interpreting observational studies: Why empirical calibration is needed to correct p-values. *Statistics in medicine* 2014;**33**:209–18.

7 Schuemie MJ, Hripcsak G, Ryan PB, *et al.* Empirical confidence interval calibration for population-level effect estimation studies in observational healthcare data. *Proceedings of the National Academy of Sciences of the United States of America* 2018;**115**:2571–7.
